# New Glycosylated Dihydrochalcones Obtained by Biotransformation of 2′-Hydroxy-2-methylchalcone in Cultures of Entomopathogenic Filamentous Fungi

**DOI:** 10.3390/ijms22179619

**Published:** 2021-09-05

**Authors:** Agnieszka Krawczyk-Łebek, Monika Dymarska, Tomasz Janeczko, Edyta Kostrzewa-Susłow

**Affiliations:** Department of Chemistry, Faculty of Biotechnology and Food Science, Wrocław University of Environmental and Life Sciences, 50-375 Wrocław, Poland; monika.dymarska@upwr.edu.pl (M.D.); tomasz.janeczko@upwr.edu.pl (T.J.)

**Keywords:** flavonoids, biotransformations, glycosylation, methylchalcone, *O*-methylglucosides, *Beauveria bassiana*, *Isaria fumosorosea*, *Isaria farinosa*

## Abstract

Flavonoids, including chalcones, are more stable and bioavailable in the form of glycosylated and methylated derivatives. The combined chemical and biotechnological methods can be applied to obtain such compounds. In the present study, 2′-hydroxy-2-methylchalcone was synthesized and biotransformed in the cultures of entomopathogenic filamentous fungi *Beauveria bassiana* KCH J1.5, *Isaria fumosorosea* KCH J2 and *Isaria farinosa* KCH J2.6, which have been known for their extensive enzymatic system and ability to perform glycosylation of flavonoids. As a result, five new glycosylated dihydrochalcones were obtained. Biotransformation of 2′-hydroxy-2-methylchalcone by *B. bassiana* KCH J1.5 resulted in four glycosylated dihydrochalcones: 2′-hydroxy-2-methyldihydrochalcone 3′-*O*-*β*-d-(4″-*O*-methyl)-glucopyranoside, 2′,3-dihydroxy-2-methyldihydrochalcone 3′-*O*-*β*-d-(4″-*O*-methyl)-glucopyranoside, 2′-hydroxy-2-hydroxymethyldihydrochalcone 3′-*O*-*β*-d-(4″-*O*-methyl)-glucopyranoside, and 2′,4-dihydroxy-2-methyldihydrochalcone 3′-*O*-*β*-d-(4″-*O*-methyl)-glucopyranoside. In the culture of *I. fumosorosea* KCH J2 only one product was formed—3-hydroxy-2-methyldihydrochalcone 2′-*O*-*β*-d-(4″-*O*-methyl)-glucopyranoside. Biotransformation performed by *I. farinosa* KCH J2.6 resulted in the formation of two products: 2′-hydroxy-2-methyldihydrochalcone 3′-*O*-*β*-d-(4″-*O*-methyl)-glucopyranoside and 2′,3-dihydroxy-2-methyldihydrochalcone 3′-*O*-*β*-d-(4″-*O*-methyl)-glucopyranoside. The structures of all obtained products were established based on the NMR spectroscopy. All products mentioned above may be used in further studies as potentially bioactive compounds with improved stability and bioavailability. These compounds can be considered as flavor enhancers and potential sweeteners.

## 1. Introduction

The widespread and well-documented evidence of health-promoting properties of dietary flavonoids results in a significant increase of interest in their application as dietary supplements and medicaments [1,2,3].

Chalcones are one of the subclasses of dietary flavonoids, which consist of two aromatic rings joined by a three-carbon α, β-unsaturated carbonyl system. Their mostly described biological activities can be highlighted as antifungal, antivirus, anti-diabetic, anti-inflammatory, and antitumor [1,4,5,6].

The poor aqueous solubility of flavonoid aglycones limits their oral bioavailability and, as a consequence, pharmacological application. To overcome this obstacle, many attempts have been made, such as shifting the site of the absorption from the large intestine to the small intestine, increasing metabolic stability and aqueous solubility, inventing novel formulations for topical delivery [7,8].

Undoubtedly flavonoid core structure and the attached functional groups affect its bioactivity and bioavailability [2]. Flavonoids with methyl moiety have improved metabolic stability, intestinal absorption and bioavailability [9,10].

Modulated physiochemical and biological properties can also be observed for glycosylated flavonoids, which exhibit improved aqueous solubility and intestinal absorption, higher plasma levels, and longer mean residence time in the blood than analogous aglycones. Flavonoid glycosides act as pro-drugs. The sugar unit increases the bioavailability and improves the delivery efficiency, while the flavonoid aglycone acts as the active substance. However, the impact of the glycosylation has not been sufficiently investigated and probably depends on the position of the sugar moiety attached [8,11,12,13,14]. Despite numerous in vitro studies concerning the biological activity of flavonoids and their glycosides, the effect on glycosylation in vitro may differ from that observed in vivo. In studies with oral flavonoid administration, glycosides showed similar or even higher anti-inflammatory, anti-diabetic, and antiallergic activity than analogous aglycones [13].

Among flavonoid subclasses, worth attention are dihydrochalcones known for their antimicrobial activity and sweet taste. Presently they are increasingly applied as a healthy alternative for traditional sweeteners in the food industry [15]. The best-known compound is neohesperidin dihydrochalcone, which is about 1500 times sweeter than sucrose and is obtained from immature citrus fruits [16]. Another widely used dietary sweetener, naringin dihydrochalcone, shows anti-oxidant activity and have been reported as a potential therapeutic agent for Alzheimer’s Disease, based on its positive effects on the cognitive function of transgenic Alzheimer’s Disease mice [17].

From natural sources, dihydrochalcones are isolated as aglycones and their glycosides.Most extensively described dihydrochalcones are phloretin and its 2′-*O*-glucoside named phloridzin, isolated from a genus of small trees and shrubs *Malus*. Many studies proved numerous biological activities of phloridzin, such as anti-diabetic, anti-inflammatory, anti-oxidant, antihyperglycemic, antimicrobial, cardio- and hepatoprotective, anti-obesity and others [18,19,20,21,22]. Similar activities have been reported for phloretin [23]. Structure-antioxidant capacity relationship studies of dihydrochalcone compounds in *Malus* proved the antioxidant activity of these compounds through various mechanisms. The presence of the hydroxyl moiety at C-2′ increased antioxidant potency of examined dihydrochalcones [24,25].

Others recently isolated from plants dihydrochalcones are, inter alia, phloretin 4-*O*-*β*-d-glucopyranoside from the stems *Homalium stenophyllum* [26], fragnanone C from *Anneslea fragrans* twigs [27], 2′,5′-dimethyl-3′-methoxy-4′,6′-dihydroxyl-dihydrochalcone from *Empetrum nigrum* L. var. *japonicum* K. Koch (E. nigrum) [28], and glucoside derivatives of phloretin, with attached one or more glucosyl moieties at different positions of flavonoid core, from subaerial parts of *Thonningia sanguinea*. The latter were tested for their *in vitro* inhibitory activities against protein tyrosine phosphatase-1B, which increased activity is related to obesity, type 2 diabetes and carcinoma [29]. Another compound, 3′-formyl-2′,4′,6′-trihydroxy-5′-methyldihydrochalcone, exhibited antimicrobial, and anticancer potential [30].

Variously substituted chalcone aglycones and glycosides can be obtained by combined chemical and biotechnological methods with entomopathogenic filamentous fungi as biocatalysts [12,31,32,33,34,35,36,37,38,39] and concerned as new, potentially biologically active compounds with increased bioavailability.

The present study is a continuation of our previous research studies with 2′-hydroxy-5′-methylchalcone, 6-methylflavanone and 6-methylflavone as the biotransformation substrates [33,34]. In this study, we synthesized flavonoid compound with the methyl group at C-2 of the chalcone skeleton. Subsequently, we biotransformed the resulting compound using entomopathogenic filamentous fungi strains *B. bassiana* KCH J1.5, *I. fumosorosea* KCH J2 and *I. farinosa* KCH J2.6 as biocatalysts.

As a result of biotransformation of 2′-hydroxy-2-methylchalcone in *B. bassiana* KCH J1.5 culture we obtained four glycosylated dihydrochalcones. In the case of *I. fumosorosea* KCH J2 as a biocatalyst only one glycosylation product was obtained. *I. farinosa* KCH J2.6 biotransformed 2′-hydroxy-2-methylchalcone into two products formed also by *B. bassiana* KCH J1.5.

According to our best knowledge, all obtained biotransformation products have not been previously known and can be used in biological activity and bioavailability assessment. Moreover, obtained dihydrochalcone glycosides may be concerned as potential flavor enhancers and health-promoting sweeteners.

## 2. Results

2′-Hydroxy-2-methylchalcone was utilized in microbial transformations performed by three entomopathogenic filamentous fungi strains *B. bassiana* KCH J1.5, *I. fumosorosea* KCH J2, and *I. farinosa* KCH J2.6. Microorganisms were isolated from the environment and had been effective in the glycosylation of flavonoids and steroids in our earlier studies [33,34,40,41].

Experiments were performed on a semi-preparative scale to determine the chemical structures of biotransformation products and their isolated yields.

**Scheme 1:**
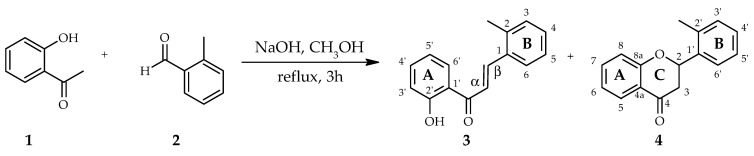
Synthesis of 2′-hydroxy-2-methylchalcone (**3**) in the Claisen–Schmidt condensation reaction.

As a result of 2′-hydroxy-2-methylchalcone biotransformation in the *B. bassiana* KCH J1.5 culture four glycosylated dihydrochalcones were obtained. In the case of *I. fumosorosea* KCH J2 as a biocatalyst, only one product was obtained. The biotransformation in the culture of *I. farinosa* KCH J2.6 resulted in the formation of two products formed also in *B. bassiana* KCH J1.5 culture. In the experiment, 2′-hydroxy-2-methylchalcone (**3**) was synthesized in the Claisen–Schmidt condensation reaction (Scheme 1) (Section 4.1).

The structure of product (**3**) was confirmed based on NMR (Nuclear Magnetic Resonance)spectroscopy (Table 1 and Table 2). In the ^1^H NMR (Proton Nuclear Magnetic Resonance) spectrum were observed two characteristic signals: the first one from the hydroxyl moiety at δ = 12.88 ppm with the corresponding signal from C-2′ in the ^13^C NMR (Carbon-13 Nuclear Magnetic Resonance) spectrum at δ = 164.5 ppm, and the second one from the three protons of the methyl moiety at δ = 2.51 ppm with the corresponding signal from C-2-CH_3_ in the ^13^C NMR spectrum at δ = 19.8 ppm (Supplementary Materials: Figures S2 and S4). In the ^1^H NMR spectrum were also observed two doublets from protons α and β (δ = 8.25 ppm and 7.94 ppm, respectively) and the signals from protons of the A and B rings (Supplementary Materials: Figure S3). In the ^13^C NMR spectrum was also observed the signal from the carbonyl group at δ = 195.0 ppm (Supplementary Materials: Figure S4). The couplings between the three protons of the methyl moiety (δ = 2.51 ppm) and the signals from carbons C-1 (δ = 134.4 ppm), C-2 (δ = 139.5 ppm), and C-3 (δ = 131.8 ppm) confirm substitution with the methyl moiety at position C-2 (Supplementary Materials: Figure S180). Moreover, the protons at C-6′ (δ = 8.28 ppm) and at C-4′ (δ = 7.58 ppm) correlate with the signal from carbon at C-2′ (δ = 164.5 ppm) confirming substitution with the hydroxyl group at position C-2′ (Supplementary Materials: Figure S17).

### 2.1. Biotransformation of 2′-Hydroxy-2-methylchalcone (3) in the Culture of B.bassiana KCH J1.5

**Scheme 2:**
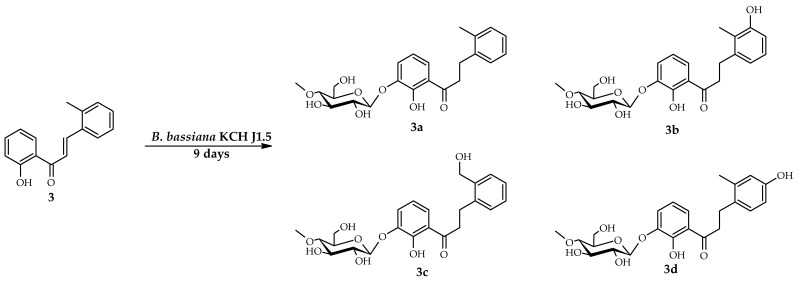
Microbial transformation of 2′-hydroxy-2-methylchalcone (**3**) in B. *bassiana* KCH J1.5 culture.

As a result of 9-day biotransformation of 2′-hydroxy-2-methylchalcone in the culture of *B. bassiana* KCH J1.5 four products were obtained: 2′-hydroxy-2-methyldihydrochalcone 3′-*O*-*β*-d-(4″-*O*-methyl)-glucopyranoside (**3a**) with a 2.9% yield (2.6 mg), 2′,3-dihydroxy-2-methyldihydrochalcone 3′-*O*-*β*-d-(4″-*O*-methyl)-glucopyranoside (**3b**) with a 13.1% yield (12.3 mg), 2′-hydroxy-2-hydroxymethyldihydrochalcone 3′-*O*-*β*-d-(4″-*O*-methyl)-glucopyranoside (**3c**) with a 4.8% yield (4.5 mg), and 2′,4-dihydroxy-2-methyldihydrochalcone 3′-*O*-*β*-d-(4″-*O*-methyl)-glucopyranoside (**3d**) with a 5.0% yield (4.7 mg) (Scheme 2). Biotransformation products were isolated and purified utilizing the preparative Thin Layer Chromatography (TLC) method with a mixture of chloroform and methanol (9:1 *v*/*v*) as eluent.

**Scheme 3:**
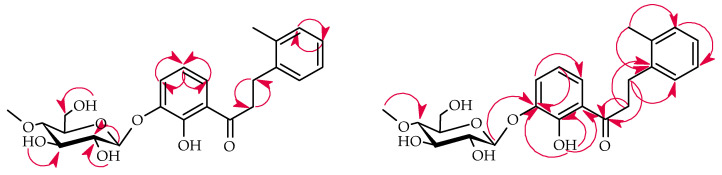
Key COSY (**on the left**) and HMBC (**on the right**) correlations for the structure elucidation of product (**3a**).

**Scheme 4:**
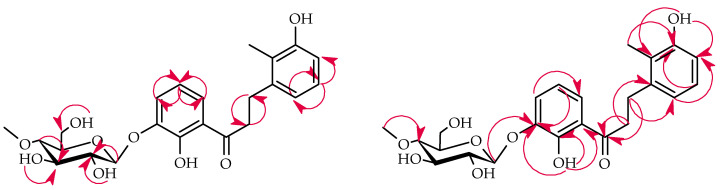
Key COSY (**on the left**) and HMBC (**on the right**) correlations for the structure elucidation of product (**3b**).

**Scheme 5:**
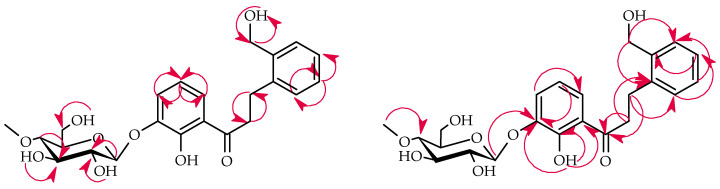
Key COSY (**on the left**) and HMBC (**on the right**) correlations for the structure elucidation of product (**3c**).

**Scheme 6:**
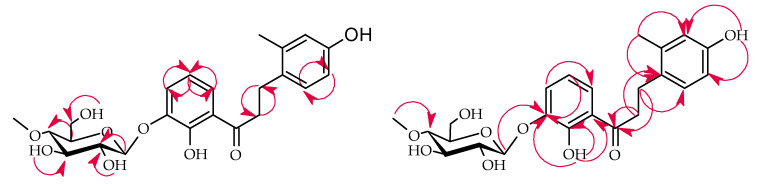
Key COSY (**on the left**) and HMBC (**on the right**) correlations for the structure elucidation of product (**3d**).

The structures of products (**3a**–**3d**) were determined based on NMR spectroscopy (Table 1 and Table 2, Scheme 3, Scheme 4, Scheme 5 and Scheme 6 showing key COSY (Correlation Spectroscopy) and HMBC (Heteronuclear Multiple Bond Coherence) correlations). In all cases, the presence of a glucoside moiety was confirmed by five characteristic carbon signals observed in the region from δ = 80.0 ppm to δ = 62.1 ppm in the ^13^C-NMR spectra (Supplementary Materials: Figures S25, S43, S62 and S80), as well as proton signals of δ_H_ ranging from δ = 3.81 ppm to δ = 3.22 ppm in the ^1^H-NMR spectra (Supplementary Materials: Figures S22, S40, S59 and S77). The attachment of a glycosidic unit to substrate (**3**) was also confirmed by a one-proton doublet from proton at the anomeric carbon atom at δ = 4.91 ppm in the ^1^H-NMR spectra. The coupling constant (*J* = 7.8 Hz) for the anomeric proton was evidence of the glucose *β*-configuration. The sugar unit C-4″ hydroxyl group has been *O*-methylated, because in the ^1^H-NMR spectra a three-proton singlet at δ = 3.56 ppm, with the corresponding signal at δ = 60.5 ppm in the ^13^C-NMR spectra was observed. The C-4″ hydroxyl group methylation was detected based on the HMBC experiment, where the proton signal due to -OCH_3_ was correlated with the signal of C-4″ (δ = 80.0 ppm) in the glucose unit (Supplementary Materials: Figures S35, S54, S72 and S90). In the products (**3a**–**3d**) also occurred reduction of a double bond between C-α and C-β. In the ^1^H NMR spectra the protons at C-α were shifted from 7.94 ppm (**3**) to 3.40 ppm (**3a**), 3.36 ppm (**3b**), 3.46 ppm (**3c**), and 3.33 ppm (**3d**). The protons at C-β were shifted from 8.25 ppm (**3**) to 3.03 ppm (**3a**), 3.02 ppm (**3b**), 3.10 ppm (**3c**), and 2.94 ppm (**3d**). Furthermore, protons at C-α and C-β were correlated with the carbonyl group and the proton at C-α was correlated with C-1 in the HMBC spectra (Supplementary Materials: Figures S35, S54, S55, S72, S73, S91 and S92). The signals from one proton of the hydroxyl group at C-2′ in the products (**3a**–**3d**) and three protons of the methyl group at C-2 in the products (**3a**), (**3b**) and (**3d**) remained intact. In products (**3a**–**3d**) 4″-*O*-methyl-glucopyranoside was attached at C-3′, because in the HMBC spectrum shifted proton at 2′-hydroxyl group (δ = 12.14 ppm—(**3a**), δ = 12.18 ppm—(**3b**), δ = 12.17 ppm—(**3c**), δ = 12.20 ppm—(**3d**)) and the proton at the anomeric carbon atom (δ = 4.91 ppm—(**3a**–**3d**)) were correlated with shifted signal from C-3′ (δ = 147.2 ppm—(**3a**–**3d**)) spectra (Supplementary Materials: Figures S33, S35, S51, S54, S70, S72, S88 and S91). Moreover, the above mentioned hydroxyl group (δ = 12.14 ppm—(**3a**), δ = 12.18 ppm—(**3b**), δ = 12.17 ppm—(**3c**), δ = 12.20 ppm—(**3d**)) couples with the signals from C-1′ (δ = 121.5 ppm—(**3a**–**3d**)), C-2′ (δ = 153.5 ppm—(**3a**), δ = 153.6 ppm—(**3a**) and (**3d**), δ = 153.7 ppm—(**3c**)), and C-3′ (δ = 147.2 ppm—(**3a**–**3d**)), but not with the signal from C-4′, indicating that its position is at C-2′ (not at C-3′) (Supplementary Materials: Figures S33, S51, S70 and S88). The signal from proton at C-5′ (δ = 6.86 ppm—(**3a**–**3b**), δ = 6.85 ppm—(**3c**–**3d**)) also correlates with the shifted signal from C-3′ (δ = 147.2 ppm—(**3a**–**3d**)), confirming 4″-*O*-methylglucosylation occurring at C-3′ (Supplementary Materials: Figure S34, S52, S71 and S89).

The structure of the product (**3a**) was established as 2′-hydroxy-2-methyldihydrochalcone 3′-*O*-*β*-d-(4″-*O*-methyl)-glucopyranoside (Scheme 3).

In the product (**3b**) (2′,3-dihydroxy-2-methyldihydrochalcone 3′-*O*-*β*-d-(4″-*O*-methyl)-glucopyranoside), in the ^1^H NMR spectrum, the singlet from the proton of the hydroxyl group also appeared (δ = 8.16 ppm) and shifted signals from B ring were observed: one triplet from the proton at C-5 (δ = 6.92 ppm) and one doublet of doublets from the protons at C-6 and C-4 (δ = 6.72 ppm) indicating that the attachment of an electronegative hydroxyl group occurred at C-3 (Supplementary Materials: Figure S39). Moreover, in the HMBC experiment hydroxyl group at C-3 (δ = 8.16 ppm) was correlated with C-2 signal (δ = 123.2 ppm) and C-3 signal (δ = 156.3 ppm shifted from δ = 131.8 ppm in substrate (**3**)) (Supplementary Materials: Figure S52). The signal from the methyl group at C-2 (δ = 2.21 ppm) was also correlated with shifted C-3 signal (Supplementary Materials: Figure S54) (Scheme 4).

In the case of product (**3c**) (2′-hydroxy-2-hydroxymethyldihydrochalcone 3′-*O*-*β*-d-(4″-*O*-methyl)-glucopyranoside), in ^1^H NMR spectrum shifted signals from B ring were observed: one signal from the proton at C-3 (δ = 7.40 ppm), which merged with the signal from not shifted proton at C-4′, one multiplet from the proton at C-6 (δ = 7.28 ppm), and one merged signal from the protons at C-4 and C-5 (δ = 7.20 ppm) (Supplementary Materials: Figure S58). In the ^1^H NMR spectrum, two additional signals were observed: one doublet from two protons at the methylene group at C-2 (δ = 4.73 ppm) and one triplet from the proton at hydroxyl group attached to this methylene group (δ = 4.15 ppm). In the COSY experiment can be observed coupling between both signals, confirming hydroxylation of the methyl moiety at C-2 (Supplementary Materials: Figure S65). The two protons from the methylene group (δ = 4.73 ppm) was also correlated with C-1 (δ = 140.0 ppm) and C-3 (δ = 129.1 ppm) in the HMBC experiment, also indicating hydroxylation at C-2-CH_3_(Supplementary Materials: Figure S72) (Scheme 5). Such a reaction was observed previously in the case of the attachment of the hydroxyl moiety to the methyl moiety in the biotransformation of 6-methylflavanone performed also by *B. bassiana* KCH J1.5 [34].

In the product (**3d**), the substitution with the hydroxyl moiety at C-4 was confirmed by the appearance of singlet at δ = 8.02 ppm in the ^1^H NMR spectrum and correlation between this signal and signals from carbon C-3 (δ = 117.9 ppm) and C-5 (δ = 113.7 ppm) in the HMBC experiment (Supplementary Materials: Figures S76 and S88) (Scheme 6).

### 2.2. Biotransformation of 2′-Hydroxy-2-methylchalcone (3) in the Culture of I. fumosorosea KCH J2

**Scheme 7:**
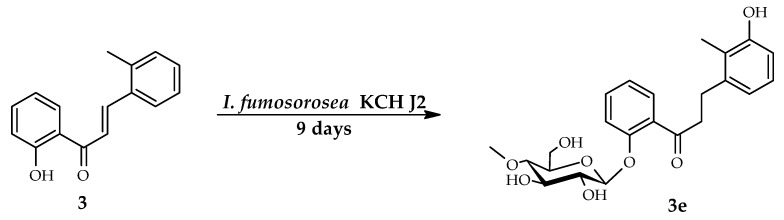
Microbial transformation of 2′-hydroxy-2-methylchalcone (**3**) in *I. fumosorosea* KCH J2 culture.

*I. fumosorosea* KCH J2 was the only one to biotransform 2′-hydroxy-2-methylchalcone (**3**) into 3-hydroxy-2-methyldihydrochalcone 2′-*O*-*β*-D-(4″-O-methyl)-glucopyranoside (**3e**) The isolated yield of (**3e**) was 5.3% (4.8 mg) (Scheme 7).

**Scheme 8:**
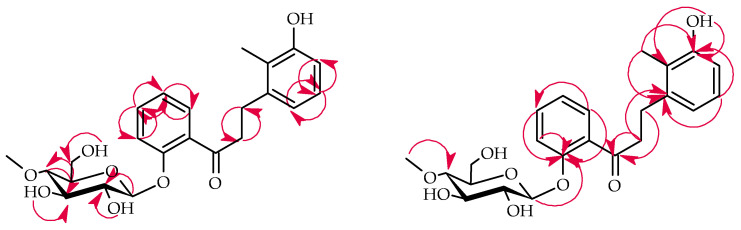
Key COSY (**on the left**) and HMBC (**on the right**) correlations for the structure elucidation of product (**3e**).

The structure of product **3e** was established based on the NMR spectroscopy (Table 1 and Table 2, Scheme 8 (with key COSY and HMBC correlations). The resulting compound was 4″-*O*-methylglycosylated dihydrochalcone, like products (**3a**–**3d**). However, in this case, glycosylation occurred at C-2. In the ^1^H NMR spectrum signal from the hydroxyl moiety at C-2′ (δ = 12.88 ppm) disappeared, indicating substitution at this position. At δ = 8.08 ppm appeared singlet from one proton demonstrating substrate hydroxylation at another position (Supplementary Materials: Figure S95). The three-proton signal of the methyl moiety at C-2 was shifted from δ = 2.51 ppm to δ = 2.19 ppm (like product (**3b**)) indicating an electronegative atom’s attachment in the adjacent position at C-3. Moreover, in the HMBC experiment, the signal from the hydroxyl group at C-3 (δ = 8.08 ppm) was coupled with the shifted signal from the carbon at C-3 (δ = 131.8 ppm in (**3**), δ = 156.1 ppm in (**3e**)) and triplet from one proton at C-5 (δ = 6.90 ppm) was also coupled with the signal from carbon at C-3 (Supplementary Materials: Figure S107). The substitution with the 4″-*O*-methylglucosyl moiety at C-2′ was confirmed by coupling between the signal from proton at the anomeric carbon atom (δ = 5.04 ppm) and the signal from carbon at C-2′, which was shifted from δ = 164.5 ppm to δ = 157.1 ppm (Supplementary Materials: Figure S109). The coupling between proton at 6′ (δ = 7.58 ppm), proton at C-4′ (δ = 7.47 ppm), proton at C-3′ (δ = 7.30 ppm) and carbon at C-2′ also confirmed glycosylation at C-2′ (Supplementary Materials: Figure S107).

### 2.3. Biotransformations of 2′-Hydroxy-2-methylchalcone (3) in the Culture of I. farinosa KCH J2.6

**Scheme 9:**
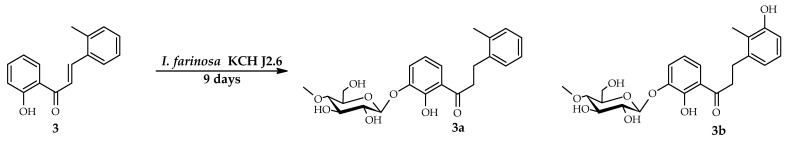
Microbial transformation of 2′-hydroxy-2-methylchalcone(**3**) in *I. farinosa* KCH J2.6 culture.

In the case of biotransformation of 2′-hydroxy-2-methylchalcone (**3**) performed in *I. farinosa* KCH J2.6 culture two products were obtained that were formed also by B. *bassiana* KCH J1.5 2′-hydroxy-2-methyldihydrochalcone 3′-*O*-*β*-d-(4″-O-methyl)-glucopyranoside (**3a**) with 7.9% yield (7.2 mg) and 2′,3-dihydroxy-2-methyldihydrochalcone 3′-*O*-*β*-d-(4″-O-methyl)-glucopyranoside (**3b**) with 9.6% yield (9.0 mg) (Scheme 9).

## 3. Discussion

The previous studies of enzymatic *O*-glycosylation of chalcones by uridine diphosphate (UDP) glycosyltransferases (UGTs) showed three sites of the attachment of the glucosyl moiety: 2′, 4′and less often 4 [23]. The product obtained in the presented study in the culture of *I. fumosorosea* KCH J2 3-hydroxy-2-methyldihydrochalcone 2′-*O*-*β*-D-(4″-*O*-methyl)-glucopyranoside (**3e**) corresponds to this pattern. However, microbial glycosylation of chalcones performed by filamentous fungi is rarely reported. Biotransformation of xanthohumol in the cultures of *Penicillium chrysogenum* 6933 [42], *Absidia coerulea* AM93 and *Rhizopus nigricans* UPF701 [43] led to the formation of 4′-*O*-*β*-D-glucopyranoside derivative and in the cultures of *B. bassiana* AM278 [44] and *B. bassiana* AM446 [43] to 4′-*O*-*β*-D-(4″-*O*-methyl)-glucopyranoside derivative. In our previous study, we showed results of the glycosylation of 2′-hydroxy-5′-methylchalcone. Both used strains *B. bassiana* KCH J1.5 and *I. fumosorosea* KCH J2 were capable of 4″-*O*-methyl-glycosylation at C-3 [34]. On the other hand, to the best of our knowledge, the attachment of the 4″-*O*-methyl-glucosyl moiety at C-3′ observed in the cultures *B. bassiana* KCH J1.5 and *I. farinosa* KCH J2.6 has not been described in the literature yet and is related to the specific regioselectivity of entomopathogenic filamentous fungi enzymes involved in this reaction.

The attempts have also been made to glycosylate dihydrochalcones. Overwin et al. showed that a non-Leloir glycosyltransferase, amylosucrase from *Neisseria polysaccharea*, performed the stereospecific glycosylation of phloretin at position C-4′ [45].

On the other hand, the reduction of the double bond of chalcones has been described in the literature as the result of biotransformation performed by many microorganisms: bacteria (*Stenotrophomonas maltophilia* KB2 [46], *Rhodococcus sp*. DSM 364, *Lactobacillus* sp. C2 PCM 2851, *Rhodotorula rubra* AM 82 [5]), cyanobacteria (*Spirulina platensis*, *Anabaena* sp., *Anabaena laxa*, *Aphanizomenon klebahnii*, *Nodularia moravica*, *Chroococcus minutes*, *Merismopedia glauca*, *Synechocystis aquatilis* [47], yeast (*Yarrowia lipolytica* KCH 71, *Rhodotorula glutinis* KCH 242, *Rhodotorula rubra* KCH 4, *Saccharomyces cerevisiae* KCH 464 [15], *Y. lipolytica* KCH 71 [48], *S. cerevisiae*—a baker’s yeast (BY) from Fleischmann and two industrial yeast strains (CAT-1 and PE-2) [49]), filamentous fungi (*Syncephalastrum racemosum* KCH 105, *Chaetomium* sp. KCH 6651, *Didymosphaeria igniaria* KCH 6670, *A. coerulea* KCH 93, *Fusarium culmorum* KCH 10) [15].

However, to the best of our knowledge, we are the first team to describe biotransformations leading to the reduction of the double bond together with the glycosylation of the chalcone substrate. All three used strains of entomopathogenic filamentous fungi *B. bassiana* KCH J1.5, *I. fumosorosea* KCH J2, and *I. farinosa* KCH J2.6 were able to carry out such reactions.

## 4. Materials and Methods

### 4.1. Substrate

The biotransformation substrate—2′-hydroxy-2-methylchalcone (**3**) was obtained by the Claisen–Schmidt condensation reaction between 2′-hydroxyacetophenone (**1**) and 2-methylbenzaldehyde (**2**) (purchased from Sigma-Aldrich (St. Louis, MO, USA)), showed in Scheme 1. The compounds were dissolved in methanol and the process was carried out under alkaline conditions at high temperature [34,50,51]. 2′-Hydroxy-2-methylchalcone (**3**) was obtained with a 63.8% yield and was used as the biotransformation substrate. As a result of spontaneous cyclization of 2′-hydroxy-2-methylchalcone (**3**) 2′-methylflavanone (**4**) was obtained with 28.2% yield (Scheme 1).

The physical data, including color and form, melting point (°C), molecular ion mass, molecular formula, retention time t_R_ (min), retardation factor Rf, andNMR spectral data of the resulting compound (**3**)are presented below, in Table 1 and Table 2, and in the Supplementary Materials.

#### 2′-Hydroxy-2-methylchalcone (3)

Yellow crystals, mp = 81–82 °C, ESIMS m/z 239.0 ([M + H]^+^, C_16_H_14_O_2_), t_R_ = 18.47, Rf = 0.95, ^1^H-NMR, see Table 1, ^13^C-NMR, see Table 2, Supplementary Materials: Figures S1–S18.

### 4.2. Microorganisms

The biotransformations were performed using three strains of entomopathogenic filamentous fungi *B. bassiana* KCH J1.5, *I. fumosorosea* KCH J2, and *I. farinosa* KCH J2.6. Microorganisms were assembled from the Department of Chemistry of Wrocław University of Environmental and Life Sciences, Poland.

The description of material collection, fungi propagation, and genetic identification have already been described in our previous papers [33,40]. The filamentous fungi were maintained on potato slants at 4°C and were subcultured to a liquid medium before use in the experiments [33,40].

### 4.3. Analysis

The course of the biotransformation was tracked by chromatographic methods (TLC, HPLC). TLC analysis was carried out using TLC Silica gel 60/Kieselguhr F254 (0.2 mm thick) plates (Merck, Darmstadt, Germany). As the developing system was used a mixture of chloroform (Chempur, Piekary Śląskie, Poland) and methanol (Chempur, Piekary Śląskie, Poland) (9:1 *v/v*). The products were observed under the ultraviolet lamp at λ = 254 nm and λ = 365 nm without additional visualization.

HPLC analyses were performed on a Dionex Ultimate 3000 instrument (Thermo Fisher Scientific, Waltham, MA, USA) with a DAD-3000 diode array detector using analytical octadecyl silica (ODS) 2 column (4.6 mm × 250 mm, Waters, Milford, MA, USA) and pre-column. The gradient program was as follows: initial conditions—32.5% B in A, 4 min—40% B in A, 8 min—40% B in A, 10 min—45% B in A, 15 min—95% B in A, 18 min—95% B in A, 19 min—32.5% B in A, 23 min—32.5% B in A. The flow rate was 1 mL min^−1^, the injection volume was 5 µL, and the detection wavelength was 280 nm [34].

Separation of the products obtained by the scale-up biotransformation was achieved using 500 and 1000 µM preparative TLC silica gel plates (Analtech, Gehrden, Germany). The elution of the post-reaction products from the adsorbent on TLC plate was performed with the use of mixture of chloroform and methanol (9:1 *v*/*v*). Afterward, separate gel fractions were extracted with 20 mL of ethyl acetate (Chempur, Piekary Śląskie, Poland) three times. The extracts from a single fraction were combined. The solvent was evaporated from all fractions under reduced pressure and 0.9 cm^3^ of deuterated acetone was added to each sample prior to the NMR analysis [34].

NMR analyses (^1^H-NMR, ^13^C-NMR, COSY, Heteronuclear Single Quantum Correlation—HSQC), HMBC) were performed using a DRX Avance^TM^ 600 MHz NMR spectrometer (Bruker, Billerica, MA, USA).

Molecular formulas of all products were confirmed by analysis performed on the LC-MS 8045 SHIMADZU Triple Quadrupole Liquid Chromatograph Mass Spectrometer with electrospray ionization (ESI) source (Shimadzu, Kyoto, Japan). Identification of compounds was performed as described previously, with minor modifications [41]. Analyses were conducted using method “MRM event from precursor ion search”. It means that in each analysis, in a sample with a pure compound, only a specific ion with a known molecular mass (determined by previous NMR analysis) was searched. The separation was achieved on the Kinetex column (2.6 µM C18 100 Å, 100 mm × 3 mm, Phenomenex, Torrance, CA, USA) operated at 30 °C. The mobile phase was a mixture of 0.1% aqueous formic acid *v*/*v* (A) and acetonitrile (B). The gradient program was as follows: initial conditions—80% B in A, 6.5 min—100% B, 7 min—80% B in A. The flow rate was 0.4 mL min^−1^ and the injection volume was 5 µL. The principal operating parameters for the LC-MS were set as follows: nebulizing gas flow: 3 L min^−1^, heating gas flow: 10 L min^−1^, interface temperature: 300 °C, drying gas flow: 10 L min^−1^, data acquisition range, *m*/*z* 100–1000 Da; ionization mode, negative and positive. Data were collected with LabSolutions version 5.97 (Shimadzu, Kyoto, Japan) software.

### 4.4. Screening Procedure

The screening procedure was performed to assess time needed for the complete microbial transformation of substrate (**3**). As a growth medium for entomopathogenic filamentous fungi was used a modified Sabouraud medium (10g aminobac (purchased from BTL, Warsaw, Poland), 30 g saccharose (purchased from Chempur, Piekary Śląskie, Poland), 1 L distilled water). Firstly, the culture of filamentous fungi strain was transferred from potato slants to a 300 mL Erlenmeyer flask with 100 mL of liquid medium. This preincubation culture was bred on a rotary shaker (DHN, Warsaw, Poland) (140 rpm) at 25 °C for 72 h. Secondly, the pre-grown culture (0.5 mL) was transferred to another 300 mL Erlenmeyer flask with 100 mL of liquid medium and also incubated for 72 h at 25 °C on a rotary shaker (140 rpm). Afterward, 10 mg of the substrate, 2′-hydroxy-2-methylchalcone (**3**), dissolved in 0.5 mL of dimethyl sulfoxide (Chempur, Piekary Śląskie, Poland), was added to the flask. The molar concentration of substrate (**3**) was 0.42 mM. The samples were collected and extracted (with 30 mL of ethyl acetate) after 3, 6 and 9 days of incubating the substrate. All biotransformations were ended after confirming complete substrate conversion (or lack of further substrate conversion) after 9 days. The extracts were dried for five minutes with anhydrous magnesium sulfate (Chempur, Piekary Śląskie, Poland), concentrated using a rotary evaporator (Heidolph, Schwabach, Germany) at temperature 55 °C, and analyzed by TLC and HPLC methods. Two controls were performed: stability of the substrates under biotransformation conditions and microorganisms cultivation with no substrate added [34].

### 4.5. The Semi-Preparative Biotransformations

The semi-preparative biotransformations were performed in 2 L flasks with 500 mL of the modified Sabouraud medium each. For each biotransformation was used one flask. Firstly, 1 mL of the preincubation culture was transferred to the flask and incubated for 72 h under the same conditions as during the screening procedure. Afterward, 50 mg of the substrate, 2′-hydroxy-2-methylchalcone (**3**), dissolved in 0.5 mL of dimethyl sulfoxide, was added and biotransformation was performed for 9 days. The molar concentration of substrates (**3**) was also 0.42 mM. The biotransformation was ended after confirming the complete substrate conversion (or lack of further substrate conversion). The metabolites were extracted two times (with 300 mL of ethyl acetate each time). The joined extracts were dried for five minutes with anhydrous magnesium sulfate and concentrated using a rotary evaporator. The products of biotransformation were separated using preparative TLC plates, and then analyzed by NMR, HPLC, and LC-MS [34].

The physical data, including color and form, melting point (°C), molecular ion mass, molecular formula, retention time t_R_(min),retardation factor Rf, and NMR spectral data of the resulting compounds (**3a**–**3e**)are presented below, in Table 1 and Table 2, and in the Supplementary Materials.

#### 4.5.1. 2′-Hydroxy-2-methyldihydrochalcone 3′-*O*-*β*-d-(4″-*O*-Methyl)-glucopyranoside (**3a**)

Light-yellow crystals, mp = 80–81 °C, ESIMS m/z 431.3 ([M − H]^−^, C_23_H_28_O_8_), t_R_ = 11.74, Rf = 0.50, ^1^H-NMR, see Table 1, ^13^C-NMR, see Table 2, Supplementary Materials: Figures S19–S36.

#### 4.5.2. 2′,3-Dihydroxy-2-methyldihydrochalcone 3′-*O*-*β*-d-(4″-*O*-Methyl)-glucopyranoside (**3b**)

Light-yellow crystals, mp = 184–186 °C, ESIMS m/z 447.3 ([M − H]^−^, C_23_H_28_O_9_), t_R_ = 5.45, Rf = 0.34, ^1^H-NMR, see Table 1, ^13^C-NMR, see Table 2, Supplementary Materials: Figures S37–S55).

#### 4.5.3. 2′-Hydroxy-2-hydroxymethyldihydrochalcone 3′-*O*-*β*-d-(4″-*O*-Methyl)-glucopyranoside (**3c**)

Light-yellow crystals, mp = 86–87 °C, ESIMS m/z 447.3 ([M − H]^−^, C_23_H_28_O_9_), t_R_ = 4.74, Rf = 0.31, ^1^H-NMR, see Table 1, ^13^C-NMR, see Table 2, Supplementary Materials: Figures S56–S73.

#### 4.5.4. 2′,4-Dihydroxy-2-methyldihydrochalcone 3′-*O*-*β*-d-(4″-*O*-Methyl)-glucopyranoside (**3d**)

Light-yellow crystals, mp = 88–89 °C, ESIMS m/z 447.3 ([M − H]^−^, C_23_H_28_O_9_), t_R_ = 5.09, Rf = 0.33, ^1^H-NMR, see Table 1, ^13^C-NMR, see Table 2, Supplementary Materials: Figures S74–S92.

#### 4.5.5. 3-Hydroxy-2-methyldihydrochalcone 2′-*O*-*β*-D-(4″-*O*-Methyl)-glucopyranoside (**3e**)

Light-yellow crystals, mp = 55–56 °C, ESIMS m/z 431.3 ([M − H]^−^, C_23_H_28_O_8_), t_R_ = 5.58, Rf = 0.38, ^1^H-NMR, see Table 1, ^13^C-NMR, see Table 2, Supplementary Materials: Figures S93–S109.

## 5. Conclusions

In this study,2′-hydroxy-2-methylchalcone was synthesized and biotransformed in the cultures of three entomopathogenic filamentous fungi strains *B. bassiana* KCH J1.5, *I. fumosorosea* KCH J2, and *I. farinosa* KCH J2.6. The strain *B. bassiana* KCH J1.5 was capable of the reduction of the double bond of 2′-hydroxy-2-methylchacone, its glycosylation at position C-3′, and hydroxylation at C-3, C-4 and C2-CH_3_. It can be assumed that the presence of the hydroxyl moiety at C-2′ is responsible for the directing effect on glycosylation at position C-3′. The strain *I. farinosa* KCH J2.6 also formed dihydrochalcones with 4″-*O*-methyl-glycosyl group at C-3′ and hydroxylated at C-3. The last strain *I. fumosorosea* KCH J2 was capable of the reduction of the double bond of 2′-hydroxy-2-methylchacone and its glycosylation at C-2′. In all formed products, the methyl moiety at C-2 remained intact. All the above-mentioned biotransformation products have not been previously described in the literature. Their biological activity and bioavailability can be assessed in further *in vitro* and in vivo studies. Above and beyond, dihydrochalcones may be considered as flavor enhancers and potential sweeteners.

## Figures and Tables

**Table 1:** ^1^H-NMR chemical shifts δ (ppm) and coupling constants *J* (Hz) of 2′-hydroxy-2-methylchalcone (**3**) and products of its biotransformation **3a–3e** in Acetone-d6, 600 MHz (Supplementary Materials: Figures S2, S20, S38, S57, S75 and S94).

Proton		Compound				
	3	3a	3b	3c	3d	3e
H-α	7.94 (d)	3.40 (m)	3.36 (m)	3.46 (m)	3.33 (m)	3.34 (m)
	*J* = 15.3					
H-β	8.25 (d)	3.03 (m)	3.02 (m)	3.10 (m)	2.94 (m)	2.95 (m)
	*J* = 15.4					
H-3	7.30 (m)	7.15 (dd)	-	7.40 (m)	6.65 (d)	-
		*J* = 6.8			*J* = 2.5	
		*J* = 1.7				
H-4	7.36 (m)	7.10 (m)	6.72 (m)	7.20 (m)	-	6.68 (d)
						*J* = 8.0
		
H-5	7.30 (m)	7.10 (m)	6.92 (t)	7.20 (m)	6.59 (dd)	6.90 (t)
			*J* = 7.8		*J* = 8.2	*J* = 7.8
					*J* = 2.6	
H-6	7.97 (d)	7.23 (m)	6.72 (m)	7.28 (m)	7.03 (d)	6.71 (d)
	*J* = 7.8				*J* = 8.2	*J* = 7.5
			
H-3′	7.01 (m)	-	-	-	-	7.30 (dd)
						*J* = 8.4
						*J* = 0.5
H-4′	7.58 (m)	7.40 (dd)	7.40 (dd)	7.40 (m)	7.39 (dd)	7.47 (ddd)
		*J* = 8.0	*J* = 8.0		*J* = 8.0	*J* = 8.5
		*J* = 1.2	*J* = 1.1		*J* = 1.3	*J* = 7.3
						*J* = 1.8
H-5′	7.01 (m)	6.86 (t)	6.86 (t)	6.85 (t)	6.85 (t)	7.11 (td)
		*J* = 8.1	*J* = 8.1	*J* = 8.1	*J* = 81	*J* = 7.6
						*J* = 0.9
H-6′	8.28 (dd)	7.65 (dd)	7.65 (dd)	7.65 (dd)	7.64 (dd)	7.58 (dd)
	*J* = 8.4	*J* = 8.1	*J* = 8.2	*J* = 8.2	*J* = 8.1	*J* = 7.7
	*J* = 1.1	*J* = 1.3	*J* = 1.4	*J* = 1.3	*J* = 1.4	*J* = 1.8
H-1″	-	4.91 (d)	4.91 (d)	4.91 (d)	4.91 (d)	5.04 (d)
		*J* = 7.8	*J* = 7.8	*J* = 7.8	*J* = 7.8	*J* = 7.8
H-2″	-	3.50 (m)	3.50 (ddd)	3.49 (m)	3.50 (m)	3.53 (m)
			*J* = 9.1			
			*J* = 7.9			
			*J* = 3.8			
H-3″	-	3.62 (td)	3.62 (td)	3.62 (m)	3.62 (td)	3.63 (td)
		*J* = 9.1	*J* = 9.0		*J* = 9.0	*J* = 9.0
		*J* = 3.8	*J* = 3.9		*J* = 3.9	*J* = 4.3
H-4″	-	3.22 (m)	3.22 (m)	3.22 (m)	3.22 (m)	3.20 (m)
H-5″	-	3.44 (ddd)	3.44 (ddd)	3.43 (m)	3.44 (ddd)	3.49 (ddd)
		*J* = 9.7	*J* = 9.7		*J* = 9.6	*J* = 9.7
		*J* = 4.8	*J* = 4.8		*J* = 4.7	*J* = 4.6
		*J* = 2.1	*J* = 2.2		*J* = 2.1	*J* = 2.0
H-6″	-	3.81 (ddd)	3.82 (ddd)	3.81 (ddd)	3.81 (ddd)	3.83 (ddd)
		*J* = 11.6	*J* = 11.5	*J* = 11.6	*J* = 11.6	*J* = 7.3
		*J* = 5.1	*J* = 5.1	*J* = 5.2	*J* = 5.2	*J* = 4.7
		*J* = 2.0	*J* = 2.1	*J* = 2.2	*J* = 2.1	*J* = 2.2
		3.68 (ddd)	3.68 (ddd)	3.68 (ddd)	3.68 (ddd)	3.68 (m)
		*J* = 11.6	*J* = 11.6	*J* = 11.8	*J* = 11.7,	
		*J* = 6.9	*J* = 6.8	*J* = 7.3	*J* = 7.0	
		*J* = 4.9	*J* = 4.9	*J* = 4.8	*J* = 4.8	
C4″-OCH_3_	-	3.56 (s)	3.56 (s)	3.56 (s)	3.56 (s)	3.55 (s)
C2′-OH	12.88 (s)	12.14 (s)	12.18 (s)	12.17 (s)	12.20 (s)	-
C3-OH	-	-	8.16 (s)	-	-	8.08 (s)
C4-OH	-	-	-	-	8.02 (s)	-
C2-CH_3_	2.51 (s)	2.35 (s)	2.21 (s)	-	2.27 (s)	2.19 (s)
2″-OH	-	4.68 (d)	4.68 (d)	4.59 (d)	4.63 (d)	4.65 (d)
		*J* = 3.5	*J* = 3.9	*J* = 3.9	*J* = 3.7	*J* = 4.1
3″-OH	-	4.42 (d)	4.41 (d)	4.39 (d)	4.40 (d)	4.48 (d)
		*J* = 3.9	*J* = 4.0	*J* = 4.0	*J* = 4.0	*J* = 4.3
6″-OH	-	3.76 (dd)	3.76 (m)	3.74 (dd)	3.75 (dd)	3.71 (dd)
		*J* = 11.7		*J* = 11.1	*J* = 12.8	*J* = 9.3
		*J* = 4.8		*J* = 5.9	*J* = 5.8	*J* = 4.5
2-CH_2_-	-	-	-	4.73 (d)	-	-
				*J* = 5.5		
2-CH_2_-OH	-	-	-	4.15 (t)	-	-
				*J* = 5.4		

**Table 2:** ^13^C-NMR chemical shifts δ (ppm) and coupling constants *J* (Hz) of 2′-hydroxy-2-methylchalcone (**3**) and products of its biotransformation **3a**–**3e** in Acetone-d6, 151 MHz (Supplementary Materials Figures S4, S23, S41, S60, S78 and S97).

Carbon	Compound					
	3	3a	3b	3c	3d	3e
C-α	122.3	40.2	40.5	41.3	40.6	44.7
C-β	143.4	28.0	28.5	27.3	27.4	28.6
C-1	134.4	140.1	141.5	140.0	130.7	142.1
C-2	139.5	136.8	123.2	140.7	137.9	123.2
C-3	131.8	131.0	156.3	129.1	117.9	156.1
C-4	131.6	127.1	113.7	127.0	156.6	113.4
C-5	127.3	126.9	126.9	128.2	113.7	126.8
C-6	127.8	129.6	121.1	130.0	130.6	121.0
C-1′	120.9	121.5	121.5	121.5	121.5	131.0
C-2′	164.5	153.5	153.6	153.7	153.6	157.1
C-3′	119.0	147.2	147.2	147.2	147.2	117.3
C-4′	137.5	123.9	123.9	124.0	123.9	133.9
C-5′	119.9	119.3	119.3	119.3	119.3	123.1
C-6′	131.4	124.8	124.8	124.8	124.8	130.4
C-1″	-	102.6	102.6	102.6	102.6	102.4
C-2″	-	74.9	74.9	74.9	74.9	75.0
C-3″	-	77.9	77.9	77.9	77.9	78.1
C-4″	-	80.0	80.0	80.0	80.0	80.1
C-5″	-	77.2	77.1	77.2	77.2	77.3
C-6″	-	62.1	62.1	62.1	62.1	62.1
4″-OCH_3_	-	60.6	60.5	60.5	60.5	60.6
C=O	195.0	206.8	206.9	207.0	207.2	202.6
2-CH_3_	19.8	19.4	11.4	-	19.5	11.5
2-CH_2_	-	-	-	62.9	-	-

## Data Availability

Samples of the compounds **3**, **3a**–**3e** are available from the authors.

## Supplementary Materials

The following are available online at https://www.mdpi.com/article/10.3390/ijms22179619/s1.
