# Supplementary material for: New Glycosylated Dihydrochalcones Obtained by Biotransformation of 2′-Hydroxy-2-methylchalcone in Cultures of Entomopathogenic Filamentous Fungi
Source: Int J Mol Sci. 2021 Sep 5;22(17):9619. doi: 10.3390/ijms22179619 (PMC8431761; doi:10.3390/ijms22179619)
Supplement: Supplementary file 1 [file ijms-22-09619-s001.zip › ijms-1340122-supplementary.pdf]

Supplementary materials

New glycosylated dihydrochalcones obtained by biotransformation of 2′-hydroxy-2-methylchalcone in cultures of entomopathogenic filamentous fungi

Agnieszka Krawczyk-Łebek*, Monika Dymarska, Tomasz Janeczko and Edyta Kostrzewa-Susłow*

Department of Chemistry, Faculty of Biotechnology and Food Science, Wrocław University of Environmental and Life Sciences, Wrocław, Poland
*Correspondence: agnieszka.krawczyk-lebek@upwr.edu.pl, edyta.kostrzewa-suslow@upwr.edu.pl

Content

Figure S1.MS analysis of 2′-hydroxy-2-methylchalcone (**3**)
Figure S2.^{1}H NMR spectrum (*δ*, acetone-d6, 600 MHz) of 2′-hydroxy-2-methylchalcone (**3**)
Figure S3. ^{1}H NMR spectrum expansion (*δ*, acetone-d6, 600 MHz) of 2′-hydroxy-2-methylchalcone (**3**)
Figure S4. ^{13}C NMR spectrum (*δ*, acetone-d6, 151 MHz) of 2′-hydroxy-2-methylchalcone (**3**)
Figure S5. ^{13}C NMR spectrum expansion (*δ*, acetone-d6, 151 MHz) of 2′-hydroxy-2-methylchalcone (**3**)
Figure S6. ^{13}C NMR spectrum expansion (*δ*, acetone-d6, 151 MHz) of 2′-hydroxy-2-methylchalcone (**3**)
Figure S7. COSYcontour map – ^{1}H x ^{1}Hof 2′-hydroxy-2-methylchalcone (**3**)
Figure S8. COSY contour map – ^{1}H x ^{1}Hexpansion of 2′-hydroxy-2-methylchalcone (**3**)
Figure S9. COSY contour map – ^{1}H x ^{1}Hexpansion of 2′-hydroxy-2-methylchalcone (**3**)
Figure S10. HSQC contour map– ^{1}H x ^{13}C of 2′-hydroxy-2-methylchalcone (**3**)
Figure S11. HSQC contour map– ^{1}H x ^{13}C expansion of 2′-hydroxy-2-methylchalcone (**3**)
Figure S12. HSQC contour map– ^{1}H x ^{13}C expansion of 2′-hydroxy-2-methylchalcone (**3**)
Figure S13. HSQC contour map– ^{1}H x ^{13}C expansion of 2′-hydroxy-2-methylchalcone (**3**)
Figure S14. HMBC contour map– ^{1}H x ^{13}C of 2′-hydroxy-2-methylchalcone (**3**)
Figure S15. HMBC contour map– ^{1}H x ^{13}C expansion of 2′-hydroxy-2-methylchalcone (**3**)
Figure S16. HMBC contour map– ^{1}H x ^{13}C expansion of 2′-hydroxy-2-methylchalcone (**3**)
Figure S17. HMBC contour map– ^{1}H x ^{13}C expansion of 2′-hydroxy-2-methylchalcone (**3**)
Figure S18. HMBC contour map– ^{1}H x ^{13}C expansion of 2′-hydroxy-2-methylchalcone (**3**)
Figure S19.MS analysis of 2′-hydroxy-2-methyldihydrochalcone 3′-*O*-*β*-D-(4′′-*O*-methyl)-glucopyranoside (**3a**)
Figure S20. ^{1}H NMR spectrum (*δ*, acetone-d6, 600 MHz) of 2′-hydroxy-2-methyldihydrochalcone 3′-*O*-*β*-D-(4′′-*O*-methyl)-glucopyranoside (**3a**)
Figure S21. ^{1}H NMR spectrum expansion (*δ*, acetone-d6, 600 MHz) of 2′-hydroxy-2-methyldihydrochalcone 3′-*O*-*β*-D-(4′′-*O*-methyl)-glucopyranoside (**3a**)
Figure S22. ^{1}H NMR spectrum expansion (*δ*, acetone-d6, 600 MHz) of 2′-hydroxy-2-methyldihydrochalcone 3′-*O*-*β*-D-(4′′-*O*-methyl)-glucopyranoside (**3a**)
Figure S23. ^{13}C NMR spectrum (*δ*, acetone-d6, 151 MHz) of 2′-hydroxy-2-methyldihydrochalcone 3′-*O*-*β*-D-(4′′-*O*-methyl)-glucopyranoside (**3a**)

Figure S24. ^{13}C NMR spectrum expansion (*δ,* acetone-d6, 151 MHz) of 2′-hydroxy-2-methyldihydrochalcone 3′-*O*-*β*-D-(4′′-*O*-methyl)-glucopyranoside (**3a**)
Figure S25. ^{13}C NMR spectrum expansion (*δ,* acetone-d6, 600 MHz) of 2′-hydroxy-2-methyldihydrochalcone 3′-*O*-*β*-D-(4′′-*O*-methyl)-glucopyranoside (**3a**)
Figure S26. COSY contour map – ^{1}H x ^{1}Hof 2′-hydroxy-2-methyldihydrochalcone 3′-*O*-*β*-D-(4′′-*O*-methyl)-glucopyranoside (**3a**)
Figure S27. COSY contour map – ^{1}H x ^{1}Hexpansion of 2′-hydroxy-2-methyldihydrochalcone 3′-*O*-*β*-D-(4′′-*O*-methyl)-glucopyranoside (**3a**)
Figure S28. COSY contour map – ^{1}H x ^{1}Hexpansion of 2′-hydroxy-2-methyldihydrochalcone 3′-*O*-*β*-D-(4′′-*O*-methyl)-glucopyranoside (**3a**)
Figure S29. HSQC contour map– ^{1}H x ^{13}C of 2′-hydroxy-2-methyldihydrochalcone 3′-*O*-*β*-D-(4′′-*O*-methyl)-glucopyranoside (**3a**)
Figure S30. HSQC contour map– ^{1}H x ^{13}C expansion of 2′-hydroxy-2-methyldihydrochalcone 3′-*O*-*β*-D-(4′′-*O*-methyl)-glucopyranoside (**3a**)
Figure S31. HSQC contour map– ^{1}H x ^{13}C expansion of 2′-hydroxy-2-methyldihydrochalcone 3′-*O*-*β*-D-(4′′-*O*-methyl)-glucopyranoside (**3a**)
Figure S32. HMBC contour map– ^{1}H x ^{13}C of 2′-hydroxy-2-methyldihydrochalcone 3′-*O*-*β*-D-(4′′-*O*-methyl)-glucopyranoside (**3a**)
Figure S33. HMBC contour map– ^{1}H x ^{13}C expansion of 2′-hydroxy-2-methyldihydrochalcone 3′-*O*-*β*-D-(4′′-*O*-methyl)-glucopyranoside (**3a**)
Figure S34. HMBC contour map– ^{1}H x ^{13}C expansion of 2′-hydroxy-2-methyldihydrochalcone 3′-*O*-*β*-D-(4′′-*O*-methyl)-glucopyranoside (**3a**)
Figure S35. HMBC contour map– ^{1}H x ^{13}C expansion of 2′-hydroxy-2-methyldihydrochalcone 3′-*O*-*β*-D-(4′′-*O*-methyl)-glucopyranoside (**3a**)
Figure S36. HMBC contour map– ^{1}H x ^{13}C expansion of 2′-hydroxy-2-methyldihydrochalcone 3′-*O*-*β*-D-(4′′-*O*-methyl)-glucopyranoside (**3a**)
Figure S37.MS analysis of 2′, 3-dihydroxy-2-methyldihydrochalcone 3′-*O*-*β*-D-(4′′-*O*-methyl)-glucopyranoside (**3b**)
Figure S38. ^{1}H NMR spectrum (*δ,* acetone-d6, 600 MHz) of 2′, 3-dihydroxy-2-methyldihydrochalcone 3′-*O*-*β*-D-(4′′-*O*-methyl)-glucopyranoside (**3b**)
Figure S39. ^{1}H NMR spectrum expansion (*δ,* acetone-d6, 600 MHz) of 2′, 3-dihydroxy-2-methyldihydrochalcone 3′-*O*-*β*-D-(4′′-*O*-methyl)-glucopyranoside (**3b**)
Figure S40. ^{1}H NMR spectrum expansion (*δ,* acetone-d6, 600 MHz) of 2′, 3-dihydroxy-2-methyldihydrochalcone 3′-*O*-*β*-D-(4′′-*O*-methyl)-glucopyranoside (**3b**)
Figure S41. ^{13}C NMR spectrum (*δ,* acetone-d6, 151 MHz) of 2′, 3-dihydroxy-2-methyldihydrochalcone 3′-*O*-*β*-D-(4′′-*O*-methyl)-glucopyranoside (**3b**)
Figure S42. ^{13}C NMR spectrum expansion (*δ,* acetone-d6, 151 MHz) of 2′, 3-dihydroxy-2-methyldihydrochalcone 3′-*O*-*β*-D-(4′′-*O*-methyl)-glucopyranoside (**3b**)
Figure S43. ^{13}C NMR spectrum expansion (*δ,* acetone-d6, 151 MHz) of 2′, 3-dihydroxy-2-methyldihydrochalcone 3′-*O*-*β*-D-(4′′-*O*-methyl)-glucopyranoside (**3b**)
Figure S44. COSY contour map – ^{1}H x ^{1}Hof 2′, 3-dihydroxy-2-methyldihydrochalcone 3′-*O*-*β*-D-(4′′-*O*-methyl)-glucopyranoside (**3b**)
Figure S45.COSY contour map – ^{1}H x ^{1}Hexpansion of 2′, 3-dihydroxy-2-methyldihydrochalcone 3′-*O*-*β*-D-(4′′-*O*-methyl)-glucopyranoside (**3b**)
Figure S46. COSY contour map – ^{1}H x ^{1}Hexpansion of 2′, 3-dihydroxy-2-methyldihydrochalcone 3′-*O*-*β*-D-(4′′-*O*-methyl)-glucopyranoside (**3b**)

Figure S47. HSQC contour map– ^{1}H x ^{13}C of 2′, 3-dihydroxy-2-methyldihydrochalcone 3′-*O*-*β*-D-(4′′-*O*-methyl)-glucopyranoside (**3b**)
Figure S48. HSQC contour map– ^{1}H x ^{13}C expansion of 2′, 3-dihydroxy-2-methyldihydrochalcone 3′-*O*-*β*-D-(4′′-*O*-methyl)-glucopyranoside (**3b**)
Figure S49. HSQC contour map– ^{1}H x ^{13}C expansion of 2′, 3-dihydroxy-2-methyldihydrochalcone 3′-*O*-*β*-D-(4′′-*O*-methyl)-glucopyranoside (**3b**)
Figure S50. HMBC contour map– ^{1}H x ^{13}C of 2′, 3-dihydroxy-2-methyldihydrochalcone 3′-*O*-*β*-D-(4′′-*O*-methyl)-glucopyranoside (**3b**)
Figure S51. HMBC contour map– ^{1}H x ^{13}C expansion of 2′, 3-dihydroxy-2-methyldihydrochalcone 3′-*O*-*β*-D-(4′′-*O*-methyl)-glucopyranoside (**3b**)
Figure S52. HMBC contour map– ^{1}H x ^{13}C expansion of 2′, 3-dihydroxy-2-methyldihydrochalcone 3′-*O*-*β*-D-(4′′-*O*-methyl)-glucopyranoside (**3b**)
Figure S53. HMBC contour map– ^{1}H x ^{13}C expansion of 2′, 3-dihydroxy-2-methyldihydrochalcone 3′-*O*-*β*-D-(4′′-*O*-methyl)-glucopyranoside (**3b**)
Figure S54. HMBC contour map– ^{1}H x ^{13}C expansion of 2′, 3-dihydroxy-2-methyldihydrochalcone 3′-*O*-*β*-D-(4′′-*O*-methyl)-glucopyranoside (**3b**)
Figure S55. HMBC contour map– ^{1}H x ^{13}C expansion of 2′, 3-dihydroxy-2-methyldihydrochalcone 3′-*O*-*β*-D-(4′′-*O*-methyl)-glucopyranoside (**3b**)
Figure S56.MS analysis of 2′-hydroxy-2-hydroxymethyldihydrochalcone 3′-*O*-*β*-D-(4′′-*O*-methyl)-glucopyranoside (**3c**)
Figure S57. ^{1}H NMR spectrum (*δ,* acetone-d6, 600 MHz) of 2′-hydroxy-2-hydroxymethyldihydrochalcone 3′-*O*-*β*-D-(4′′-*O*-methyl)-glucopyranoside (**3c**)
Figure S58. ^{1}H NMR spectrum expansion (*δ,* acetone-d6, 600 MHz) of 2′-hydroxy-2-hydroxymethyldihydrochalcone 3′-*O*-*β*-D-(4′′-*O*-methyl)-glucopyranoside (**3c**)
Figure S59. ^{1}H NMR spectrum expansion (*δ,* acetone-d6, 600 MHz) of 2′-hydroxy-2-hydroxymethyldihydrochalcone 3′-*O*-*β*-D-(4′′-*O*-methyl)-glucopyranoside (**3c**)
Figure S60. ^{13}C NMR spectrum (*δ,* acetone-d6, 151 MHz) of 2′-hydroxy-2-hydroxymethyldihydrochalcone 3′-*O*-*β*-D-(4′′-*O*-methyl)-glucopyranoside (**3c**)
Figure S61. ^{13}C NMR spectrum expansion (*δ,* acetone-d6, 151 MHz) of 2′-hydroxy-2-hydroxymethyldihydrochalcone 3′-*O*-*β*-D-(4′′-*O*-methyl)-glucopyranoside (**3c**)
Figure S62. ^{13}C NMR spectrum expansion (*δ,* acetone-d6, 151 MHz) of 2′-hydroxy-2-hydroxymethyldihydrochalcone 3′-*O*-*β*-D-(4′′-*O*-methyl)-glucopyranoside (**3c**)
Figure S63. COSYcontour map – ^{1}H x ^{1}Hof 2′-hydroxy-2-hydroxymethyldihydrochalcone 3′-*O*-*β*-D-(4′′-*O*-methyl)-glucopyranoside (**3c**)
Figure S64. COSY contour map – ^{1}H x ^{1}Hexpansion of 2′-hydroxy-2-hydroxymethyldihydrochalcone 3′-*O*-*β*-D-(4′′-*O*-methyl)-glucopyranoside (**3c**)
Figure S65. COSY contour map – ^{1}H x ^{1}Hexpansion of 2′-hydroxy-2-hydroxymethyldihydrochalcone 3′-*O*-*β*-D-(4′′-*O*-methyl)-glucopyranoside (**3c**)
Figure S66. HSQC contour map– ^{1}H x ^{13}C of 2′-hydroxy-2-hydroxymethyldihydrochalcone 3′-*O*-*β*-D-(4′′-*O*-methyl)-glucopyranoside (**3c**)
Figure S67. HSQC contour map– ^{1}H x ^{13}C expansion of 2′-hydroxy-2-hydroxymethyldihydrochalcone 3′-*O*-*β*-D-(4′′-*O*-methyl)-glucopyranoside (**3c**)
Figure S68. HSQC contour map– ^{1}H x ^{13}C expansion of 2′-hydroxy-2-hydroxymethyldihydrochalcone 3′-*O*-*β*-D-(4′′-*O*-methyl)-glucopyranoside (**3c**)
Figure S69. HMBC contour map– ^{1}H x ^{13}C of 2′-hydroxy-2-hydroxymethyldihydrochalcone 3′-*O*-*β*-D-(4′′-*O*-methyl)-glucopyranoside (**3c**)

Figure S70. HMBC contour map– ^{1}H x ^{13}C expansion of 2′-hydroxy-2-hydroxymethyldihydrochalcone 3′-*O*-*β*-D-(4′′-*O*-methyl)-glucopyranoside (**3c**)
Figure S71. HMBC contour map– ^{1}H x ^{13}C expansion of 2′-hydroxy-2-hydroxymethyldihydrochalcone 3′-*O*-*β*-D-(4′′-*O*-methyl)-glucopyranoside (**3c**)
Figure S72. HMBC contour map– ^{1}H x ^{13}C expansion of 2′-hydroxy-2-hydroxymethyldihydrochalcone 3′-*O*-*β*-D-(4′′-*O*-methyl)-glucopyranoside (**3c**)
Figure S73. HMBC contour map– ^{1}H x ^{13}C expansion of 2′-hydroxy-2-hydroxymethyldihydrochalcone 3′-*O*-*β*-D-(4′′-*O*-methyl)-glucopyranoside (**3c**)
Figure S74.MS analysis of 2′,4-dihydroxy-2-methyldihydrochalcone 3′-*O*-*β*-D-(4′′-*O*-methyl)-glucopyranoside (**3d**)
Figure S75. ^{1}H NMR spectrum (*δ*, acetone-d6, 600 MHz) of 2′,4-dihydroxy-2-methyldihydrochalcone 3′-*O*-*β*-D-(4′′-*O*-methyl)-glucopyranoside (**3d**)
Figure S76. ^{1}H NMR spectrum expansion (*δ*, acetone-d6, 600 MHz) of 2′,4-dihydroxy-2-methyldihydrochalcone 3′-*O*-*β*-D-(4′′-*O*-methyl)-glucopyranoside (**3d**)
Figure S77. ^{1}H NMR spectrum expansion (*δ*, acetone-d6, 600 MHz) of 2′,4-dihydroxy-2-methyldihydrochalcone 3′-*O*-*β*-D-(4′′-*O*-methyl)-glucopyranoside (**3d**)
Figure S78. ^{13}C NMR spectrum (*δ*, acetone-d6, 151 MHz) of 2′,4-dihydroxy-2-methyldihydrochalcone 3′-*O*-*β*-D-(4′′-*O*-methyl)-glucopyranoside (**3d**)
Figure S79. ^{13}C NMR spectrum expansion (*δ*, acetone-d6, 151 MHz) of 2′,4-dihydroxy-2-methyldihydrochalcone 3′-*O*-*β*-D-(4′′-*O*-methyl)-glucopyranoside (**3d**)
Figure S80. ^{13}C NMR spectrum expansion (*δ*, acetone-d6, 151 MHz) of 2′,4-dihydroxy-2-methyldihydrochalcone 3′-*O*-*β*-D-(4′′-*O*-methyl)-glucopyranoside (**3d**)
Figure S81. COSYcontour map – ^{1}H x ^{1}Hof 2′,4-dihydroxy-2-methyldihydrochalcone 3′-*O*-*β*-D-(4′′-*O*-methyl)-glucopyranoside (**3d**)
Figure S82. COSY contour map – ^{1}H x ^{1}Hexpansion of 2′,4-dihydroxy-2-methyldihydrochalcone 3′-*O*-*β*-D-(4′′-*O*-methyl)-glucopyranoside (**3d**)
Figure S83. COSY contour map – ^{1}H x ^{1}Hexpansion of 2′,4-dihydroxy-2-methyldihydrochalcone 3′-*O*-*β*-D-(4′′-*O*-methyl)-glucopyranoside (**3d**)
Figure S84. HSQC contour map– ^{1}H x ^{13}C of 2′,4-dihydroxy-2-methyldihydrochalcone 3′-*O*-*β*-D-(4′′-*O*-methyl)-glucopyranoside (**3d**)
Figure S85. HSQC contour map– ^{1}H x ^{13}C expansion of 2′,4-dihydroxy-2-methyldihydrochalcone 3′-*O*-*β*-D-(4′′-*O*-methyl)-glucopyranoside (**3d**)
Figure S86. HSQC contour map– ^{1}H x ^{13}C expansion of 2′,4-dihydroxy-2-methyldihydrochalcone 3′-*O*-*β*-D-(4′′-*O*-methyl)-glucopyranoside (**3d**)
Figure S87. HMBC contour map– ^{1}H x ^{13}C of 2′,4-dihydroxy-2-methyldihydrochalcone 3′-*O*-*β*-D-(4′′-*O*-methyl)-glucopyranoside (**3d**)
Figure S88. HMBC contour map– ^{1}H x ^{13}C expansion of 2′,4-dihydroxy-2-methyldihydrochalcone 3′-*O*-*β*-D-(4′′-*O*-methyl)-glucopyranoside (**3d**)
Figure S89. HMBC contour map– ^{1}H x ^{13}C expansion of 2′,4-dihydroxy-2-methyldihydrochalcone 3′-*O*-*β*-D-(4′′-*O*-methyl)-glucopyranoside (**3d**)
Figure S90. HMBC contour map– ^{1}H x ^{13}C expansion of 2′,4-dihydroxy-2-methyldihydrochalcone 3′-*O*-*β*-D-(4′′-*O*-methyl)-glucopyranoside (**3d**)
Figure S91. HMBC contour map– ^{1}H x ^{13}C expansion of 2′,4-dihydroxy-2-methyldihydrochalcone 3′-*O*-*β*-D-(4′′-*O*-methyl)-glucopyranoside (**3d**)
Figure S92. HMBC contour map– ^{1}H x ^{13}C expansion of 2′,4-dihydroxy-2-methyldihydrochalcone 3′-*O*-*β*-D-(4′′-*O*-methyl)-glucopyranoside (**3d**)

Figure S93.MS analysis of 3-hydroxy-2-methyldihydrochalcone 2′-*O*-*β*-D-(4′′-*O*-methyl)-glucopyranoside (**3e**)
Figure S94. ^{1}H NMR spectrum (*δ*, acetone-d6, 600 MHz) of 3-hydroxy-2-methyldihydrochalcone 2′-*O*-*β*-D-(4′′-*O*-methyl)-glucopyranoside (**3e**)
Figure S95. ^{1}H NMR spectrum expansion (*δ*, acetone-d6, 600 MHz) of 3-hydroxy-2-methyldihydrochalcone 2′-*O*-*β*-D-(4′′-*O*-methyl)-glucopyranoside (**3e**)
Figure S96. ^{1}H NMR spectrum expansion (*δ*, acetone-d6, 600 MHz) of 3-hydroxy-2-methyldihydrochalcone 2′-*O*-*β*-D-(4′′-*O*-methyl)-glucopyranoside (**3e**)
Figure S97. ^{13}C NMR spectrum (*δ*, acetone-d6, 151 MHz) of 3-hydroxy-2-methyldihydrochalcone 2′-*O*-*β*-D-(4′′-*O*-methyl)-glucopyranoside (**3e**)
Figure S98. ^{13}C NMR spectrum expansion (*δ*, acetone-d6, 151 MHz) of 3-hydroxy-2-methyldihydrochalcone 2′-*O*-*β*-D-(4′′-*O*-methyl)-glucopyranoside (**3e**)
Figure S99. ^{13}C NMR spectrum expansion (*δ*, acetone-d6, 151 MHz) of 3-hydroxy-2-methyldihydrochalcone 2′-*O*-*β*-D-(4′′-*O*-methyl)-glucopyranoside (**3e**)
Figure S100. COSY contour map – ^{1}H x ^{1}Hof 3-hydroxy-2-methyldihydrochalcone 2′-*O*-*β*-D-(4′′-*O*-methyl)-glucopyranoside (**3e**)
Figure S101. COSY contour map – ^{1}H x ^{1}Hexpansion of 3-hydroxy-2-methyldihydrochalcone 2′-*O*-*β*-D-(4′′-*O*-methyl)-glucopyranoside (**3e**)
Figure S102. COSY contour map – ^{1}H x ^{1}Hexpansion of 3-hydroxy-2-methyldihydrochalcone 2′-*O*-*β*-D-(4′′-*O*-methyl)-glucopyranoside (**3e**)
Figure S103. HSQC contour map– ^{1}H x ^{13}C of 3-hydroxy-2-methyldihydrochalcone 2′-*O*-*β*-D-(4′′-*O*-methyl)-glucopyranoside (**3e**)
Figure S104. HSQC contour map– ^{1}H x ^{13}C expansion of 3-hydroxy-2-methyldihydrochalcone 2′-*O*-*β*-D-(4′′-*O*-methyl)-glucopyranoside (**3e**)
Figure S105. HSQC contour map– ^{1}H x ^{13}C expansion of 3-hydroxy-2-methyldihydrochalcone 2′-*O*-*β*-D-(4′′-*O*-methyl)-glucopyranoside (**3e**)
Figure S106. HMBC contour map– ^{1}H x ^{13}C of 3-hydroxy-2-methyldihydrochalcone 2′-*O*-*β*-D-(4′′-*O*-methyl)-glucopyranoside (**3e**)
Figure S107. HMBC contour map– ^{1}H x ^{13}C expansion of 3-hydroxy-2-methyldihydrochalcone 2′-*O*-*β*-D-(4′′-*O*-methyl)-glucopyranoside (**3e**)
Figure S108. HMBC contour map– ^{1}H x ^{13}C expansion of 3-hydroxy-2-methyldihydrochalcone 2′-*O*-*β*-D-(4′′-*O*-methyl)-glucopyranoside (**3e**)
Figure S109. HMBC contour map– ^{1}H x ^{13}C expansion of 3-hydroxy-2-methyldihydrochalcone 2′-*O*-*β*-D-(4′′-*O*-methyl)-glucopyranoside (**3e**)

Molecular formula: $C_{16}H_{14}O_2$
Formula weight: 238.10
Ionization mode: positive
Precursor: $[M + H]^+$ 239.00
239.0000>121.0500 CE (Collision Energy): -22.0
239.0000>224.1000 CE: -17.0
239.0000>65.0500 CE: -45.0

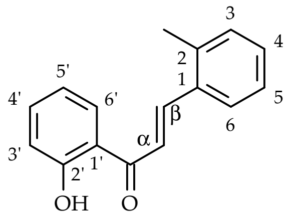

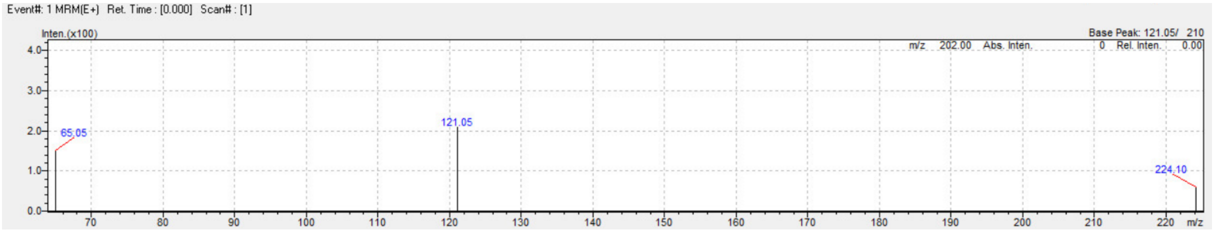

Figure S1.MS analysis of 2′-hydroxy-2-methylchalcone (**3**)

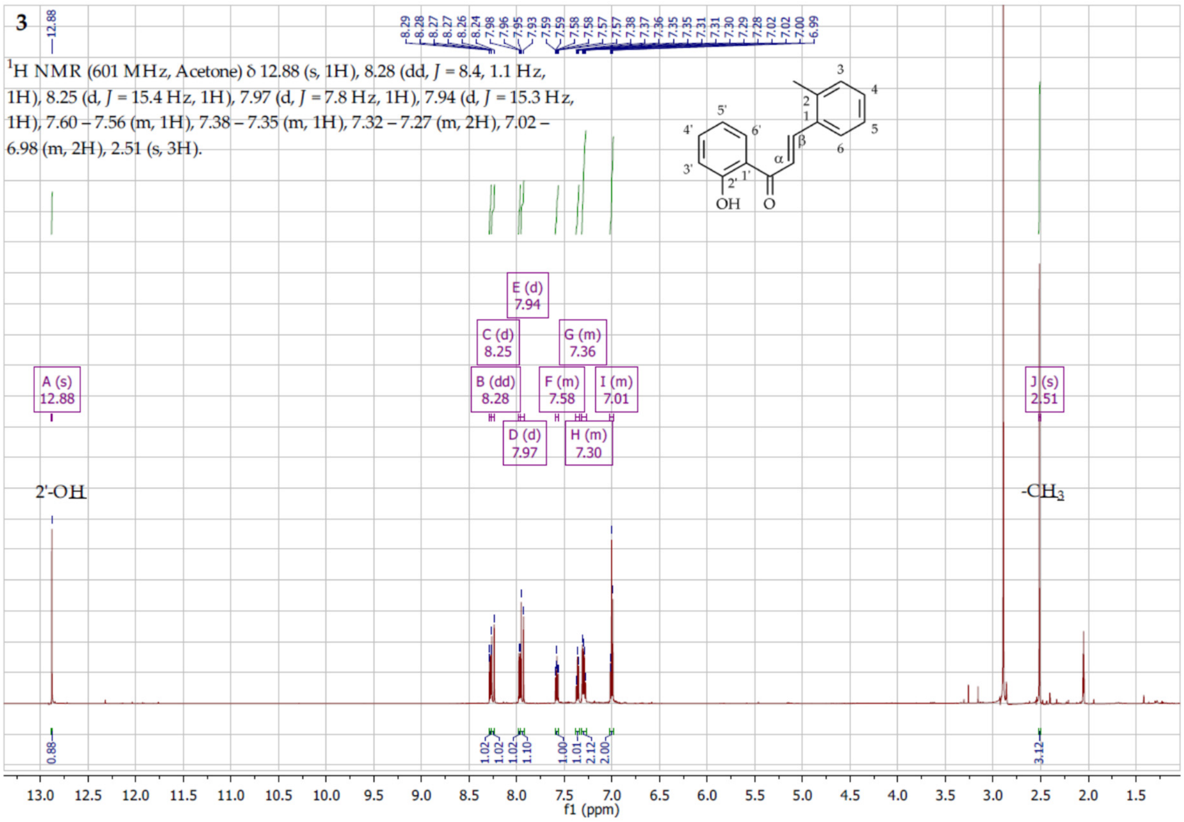

Figure S2.^{1}H NMR spectrum (δ, acetone-d6, 600 MHz) of 2′-hydroxy-2-methylchalcone (**3**)

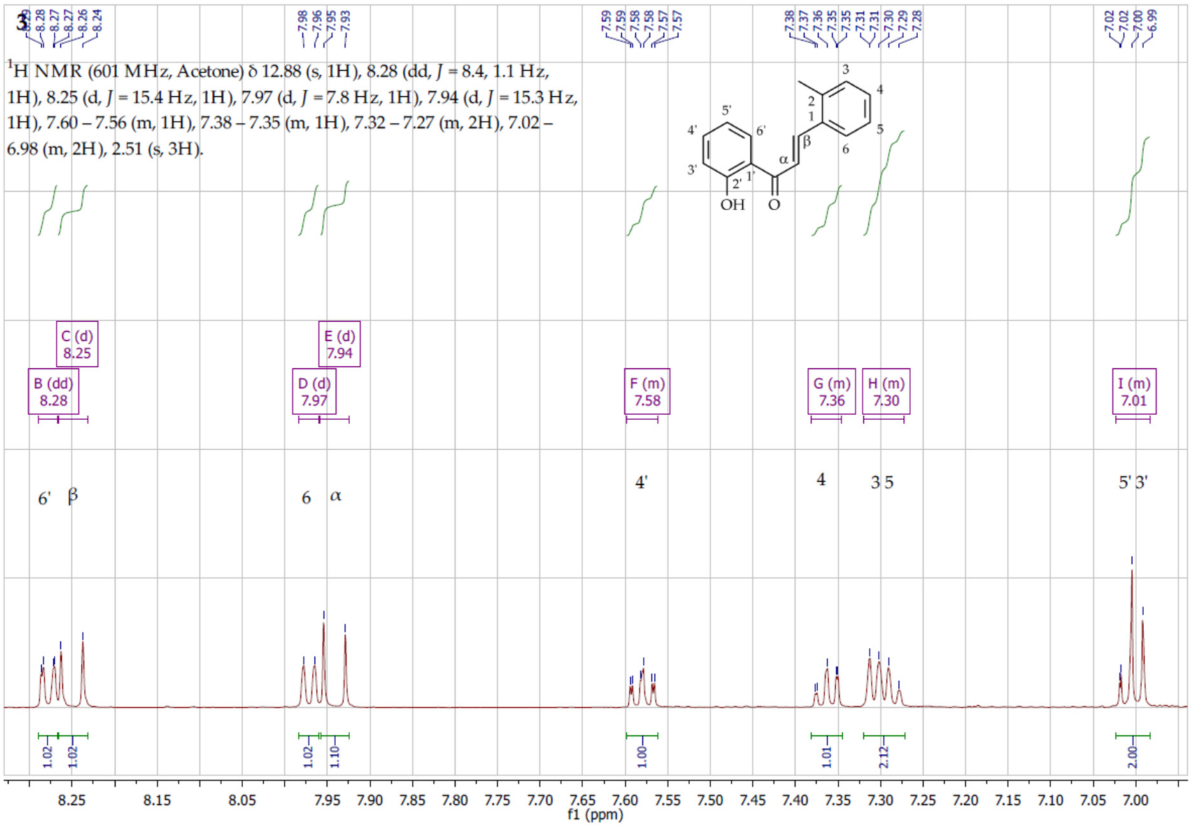

Figure S3. ^{1}H NMR spectrum expansion (*δ,* acetone-d6, 600 MHz) of 2′-hydroxy-2-methylchalcone (**3**)

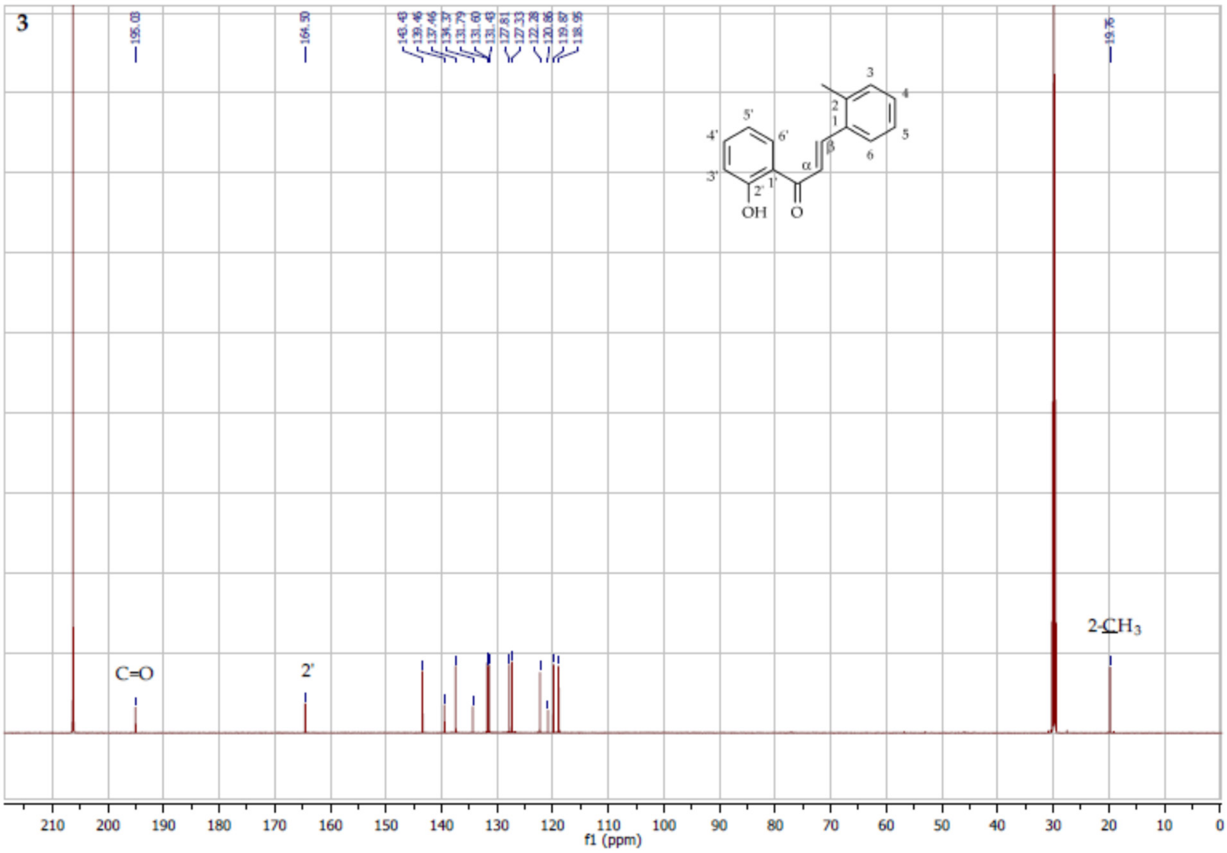

Figure S4. ^{13}C NMR spectrum (*δ,* acetone-d6, 151 MHz) of 2′-hydroxy-2-methylchalcone (**3**)

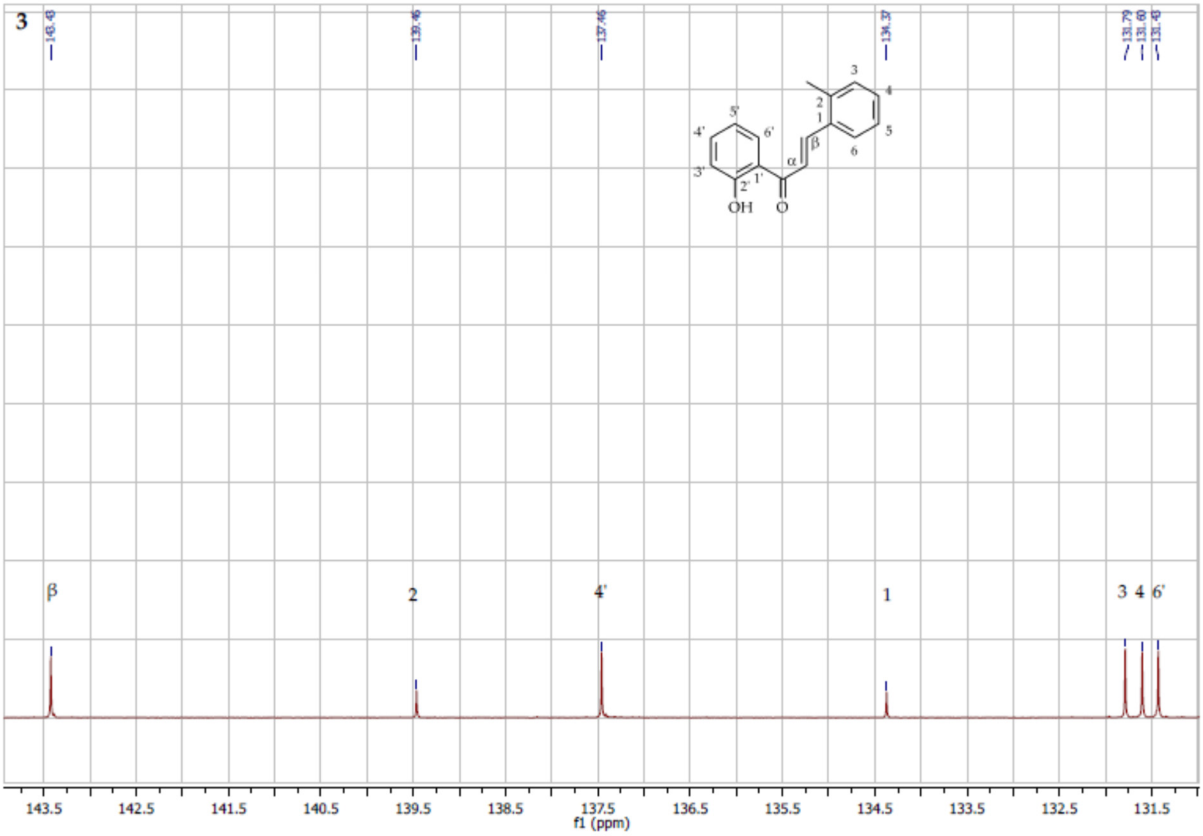

Figure S5. ^{13}C NMR spectrum expansion (δ, acetone-d6, 151 MHz) of 2′-hydroxy-2-methylchalcone (**3**)

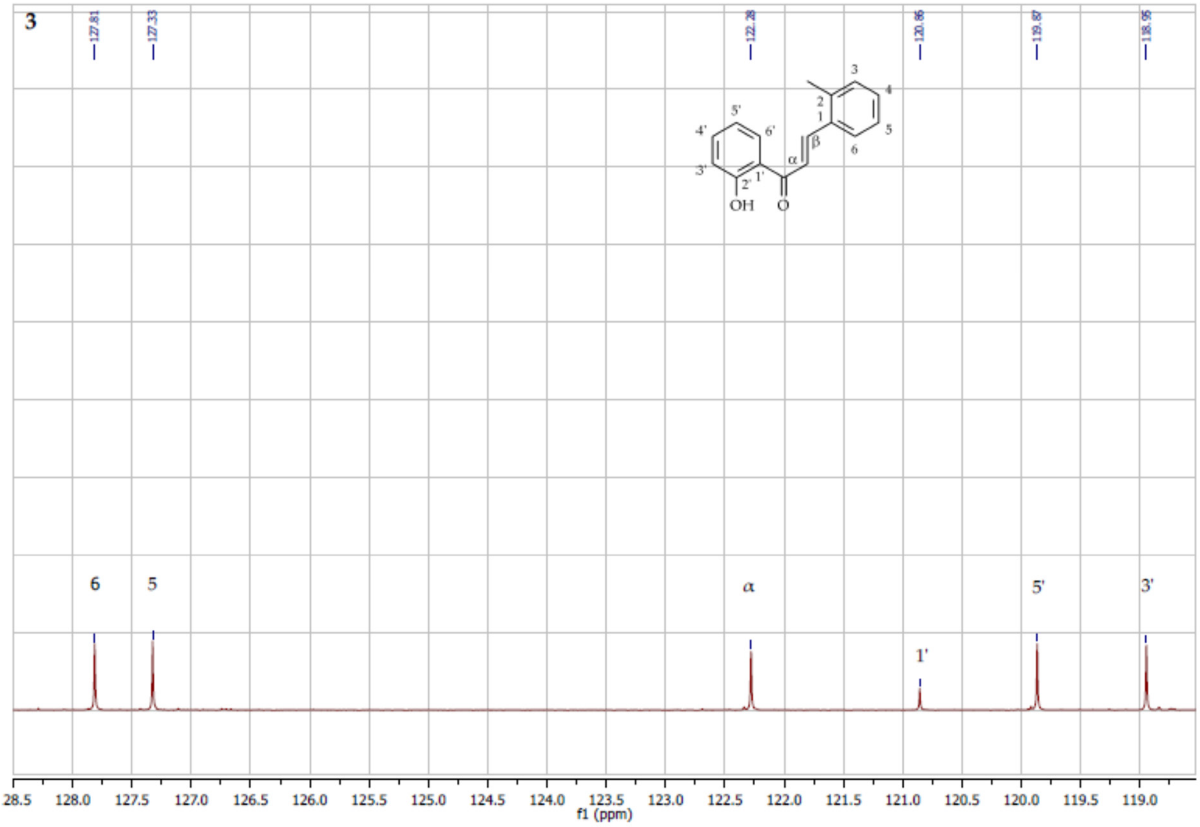

Figure S6. ^{13}C NMR spectrum expansion (δ, acetone-d6, 151 MHz) of 2′-hydroxy-2-methylchalcone (**3**)

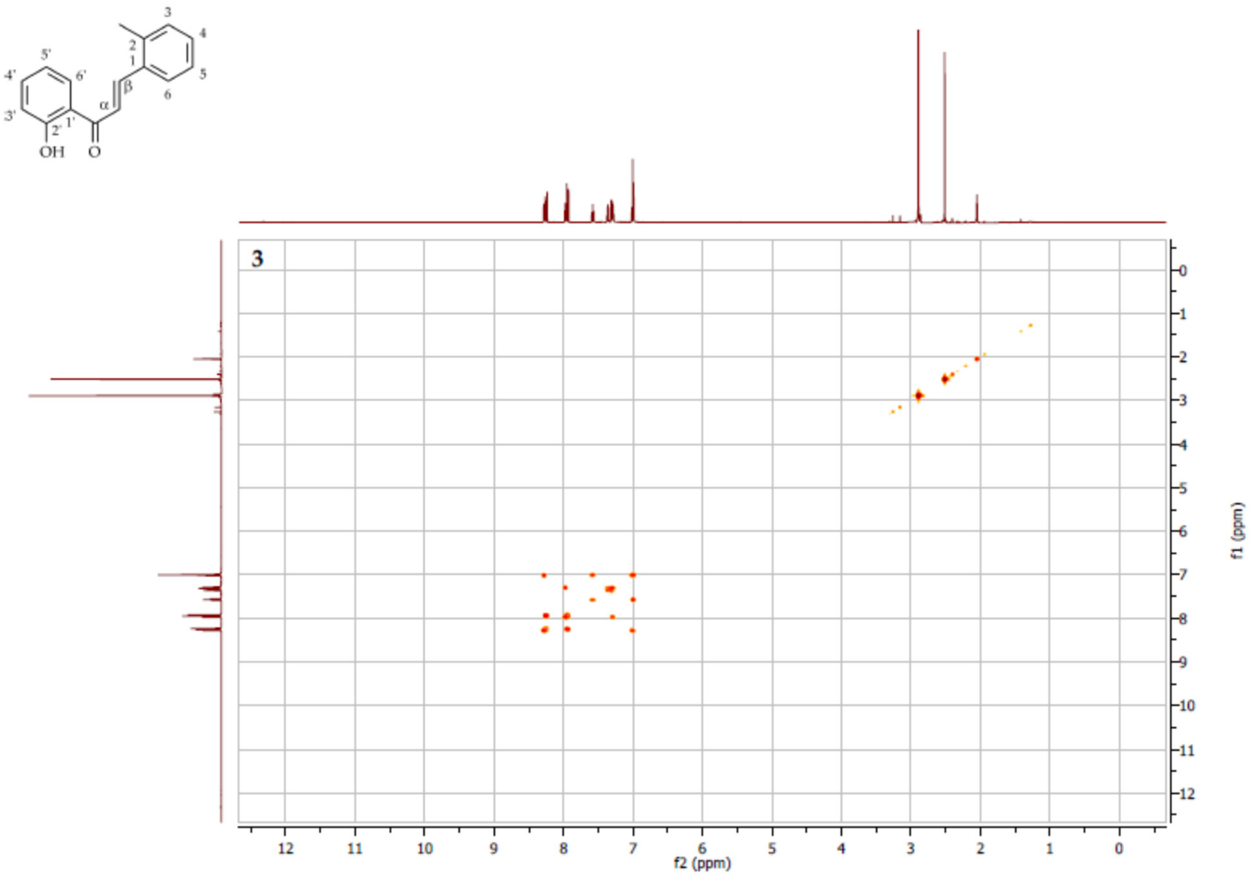

Figure S7. COSY contour map – ^{1}H x ^{1}Hof 2′-hydroxy-2-methylchalcone (**3**)

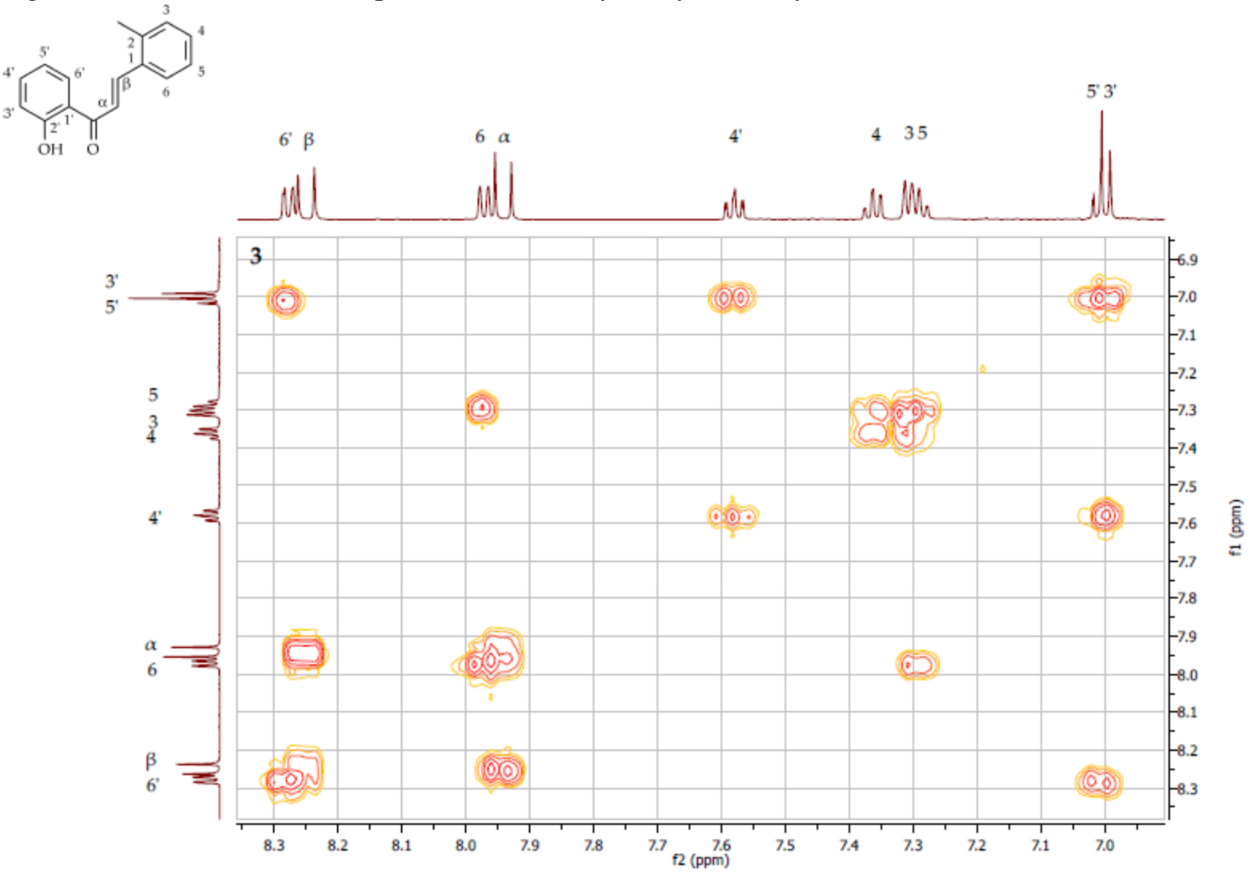

Figure S8. COSY contour map – ^{1}H x ^{1}Hexpansion of 2′-hydroxy-2-methylchalcone (**3**)

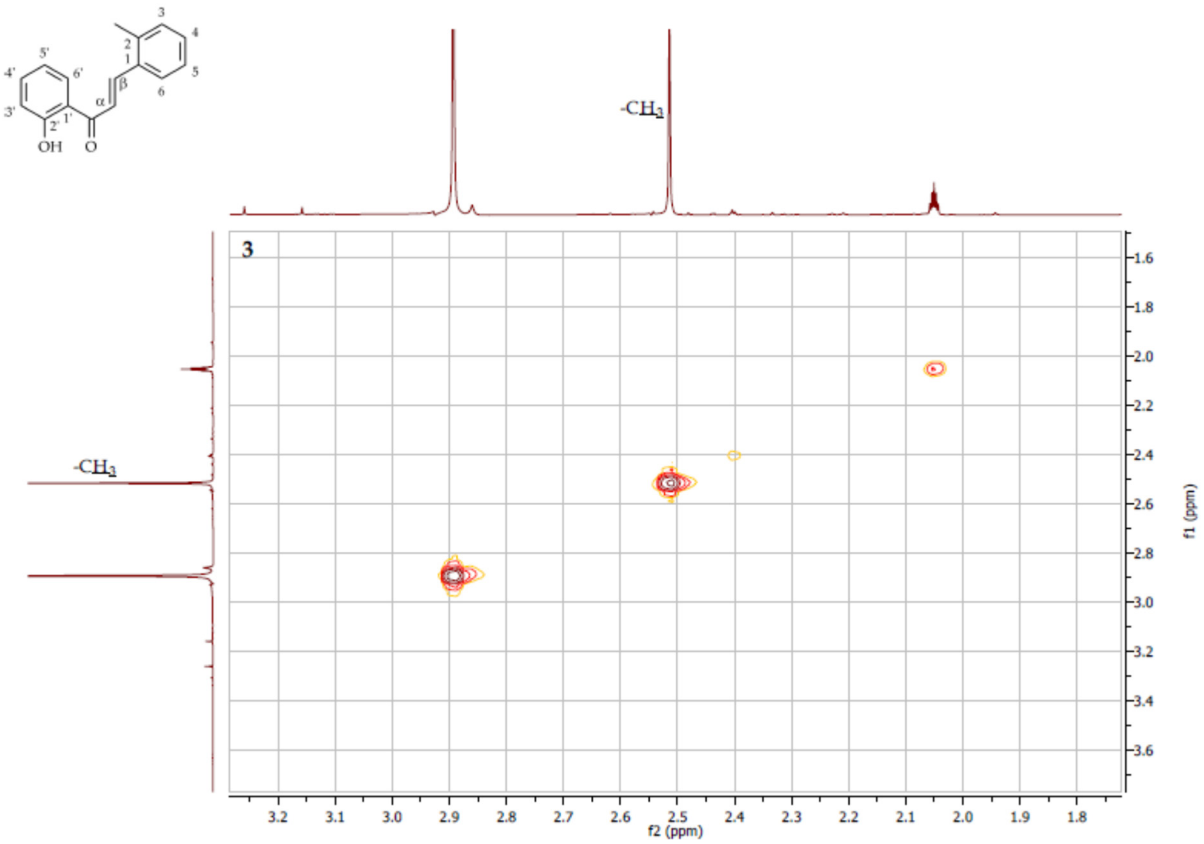

Figure S9. COSY contour map – ¹H x ¹Hexpansion of 2′-hydroxy-2-methylchalcone (**3**)

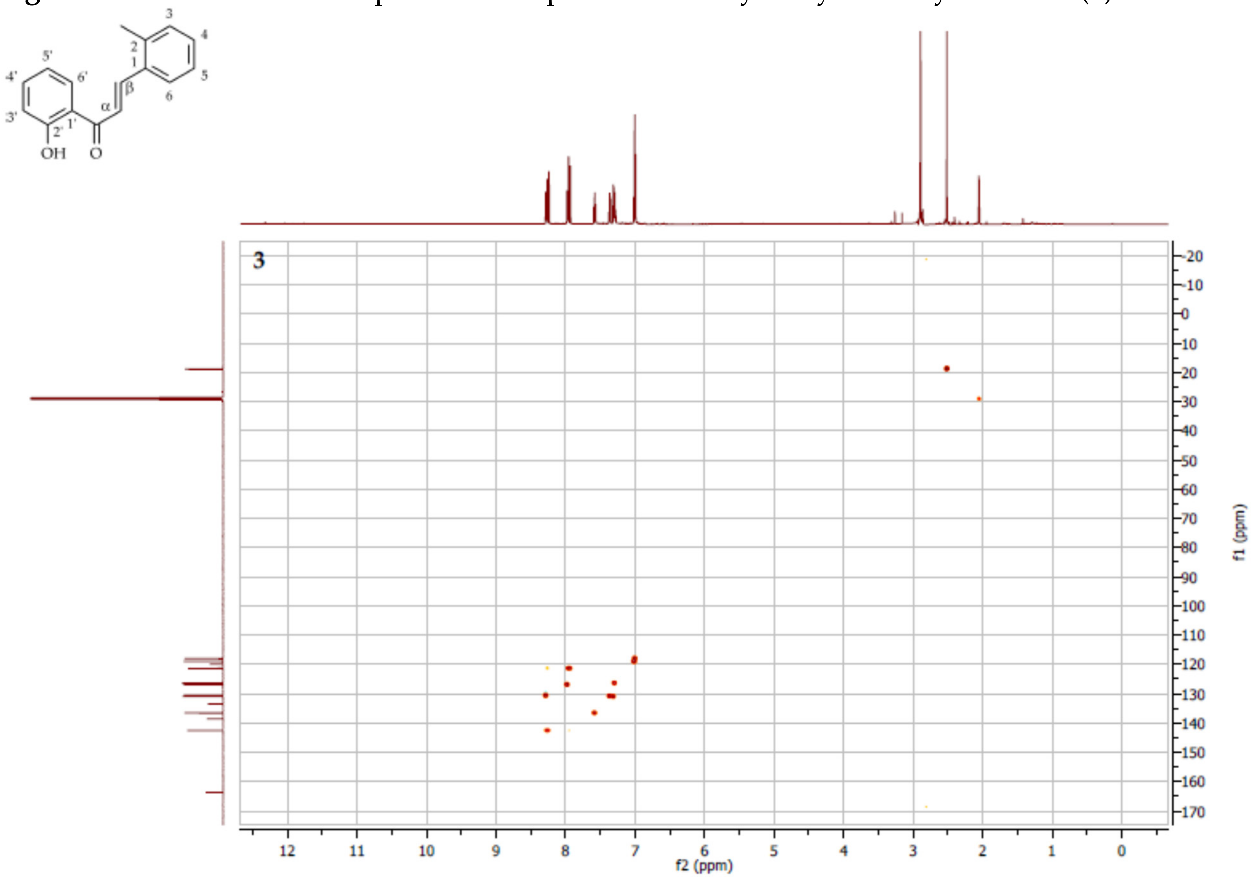

Figure S10. HSQC contour map– ¹H x ¹³C of 2′-hydroxy-2-methylchalcone (**3**)

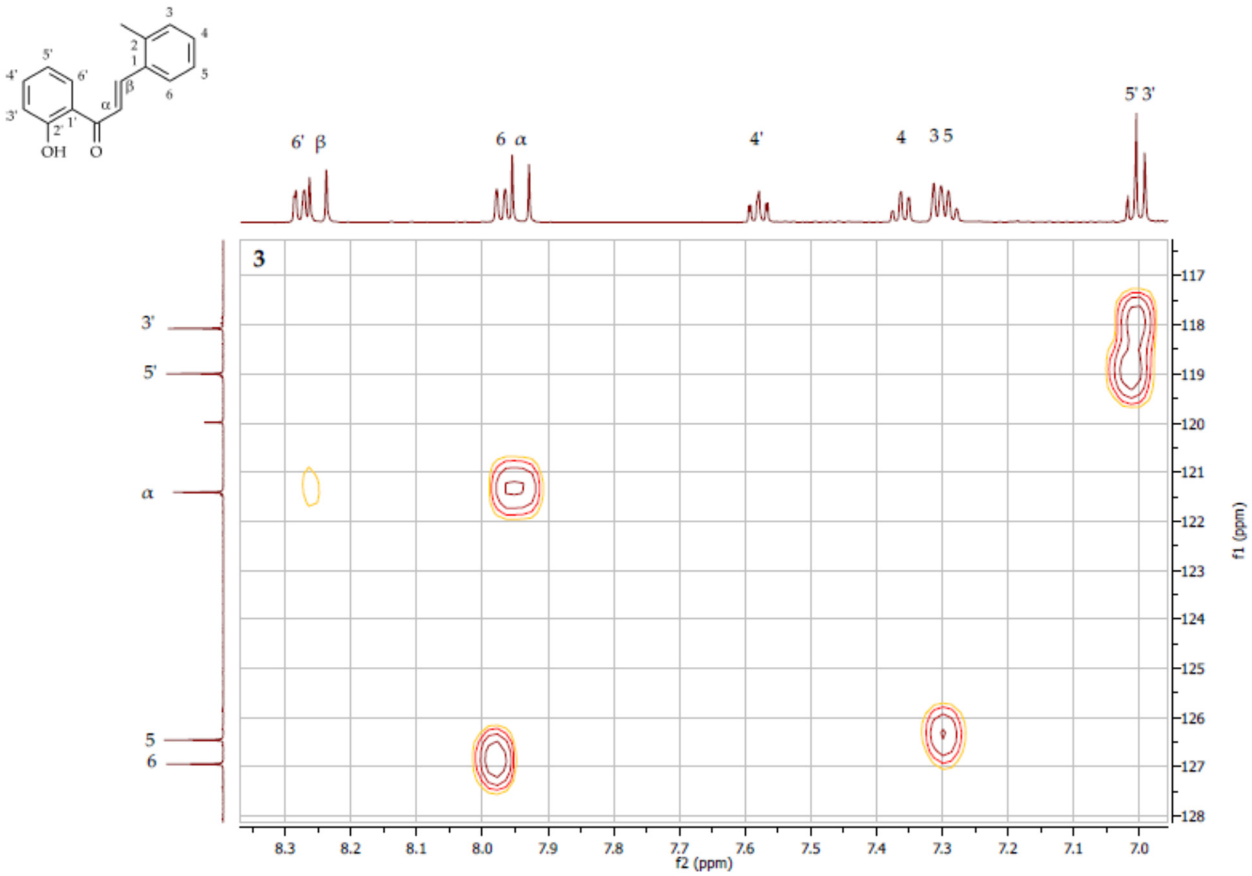

Figure S11. HSQC contour map– $^{1}H \times {}^{13}C$ expansion of 2′-hydroxy-2-methylchalcone (**3**)

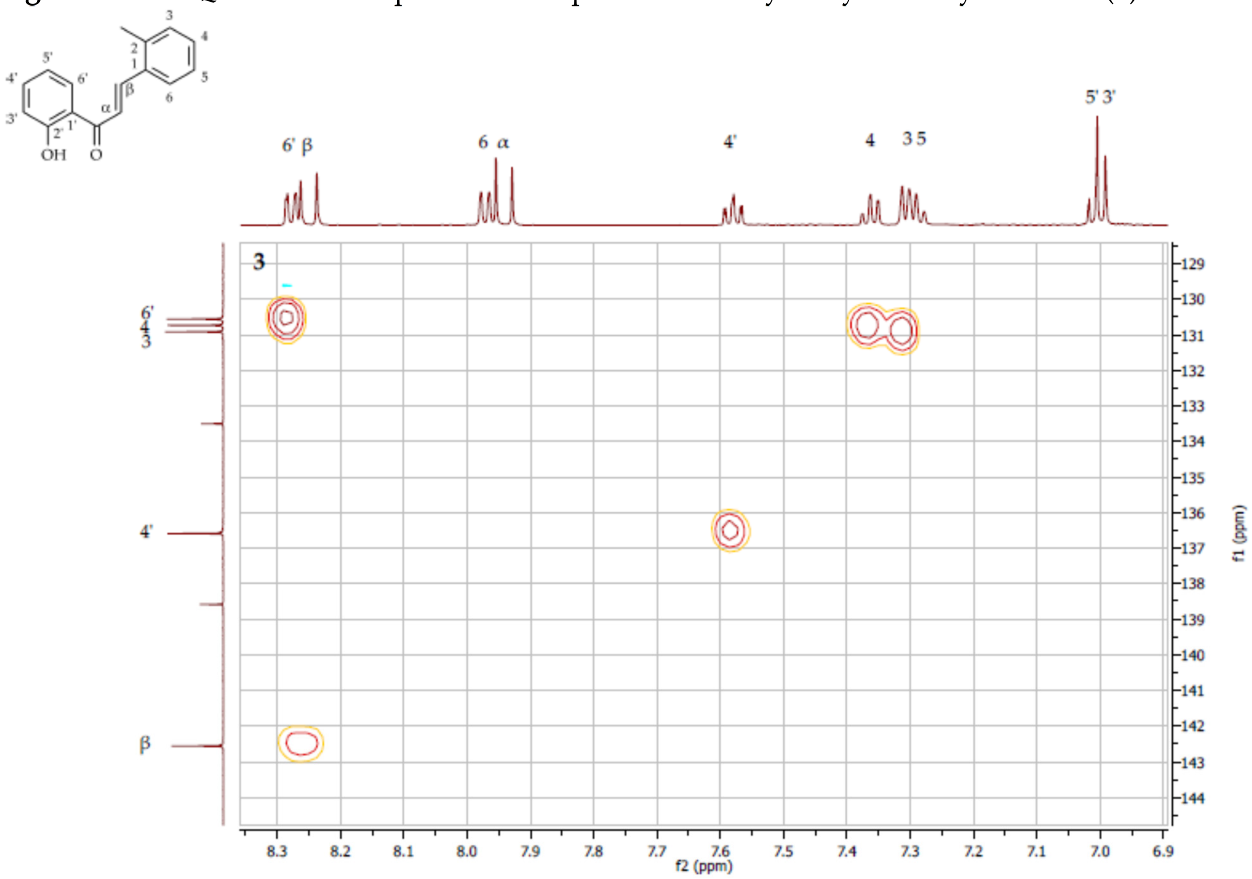

Figure S12. HSQC contour map– $^{1}H \times {}^{13}C$ expansion of 2′-hydroxy-2-methylchalcone (**3**)

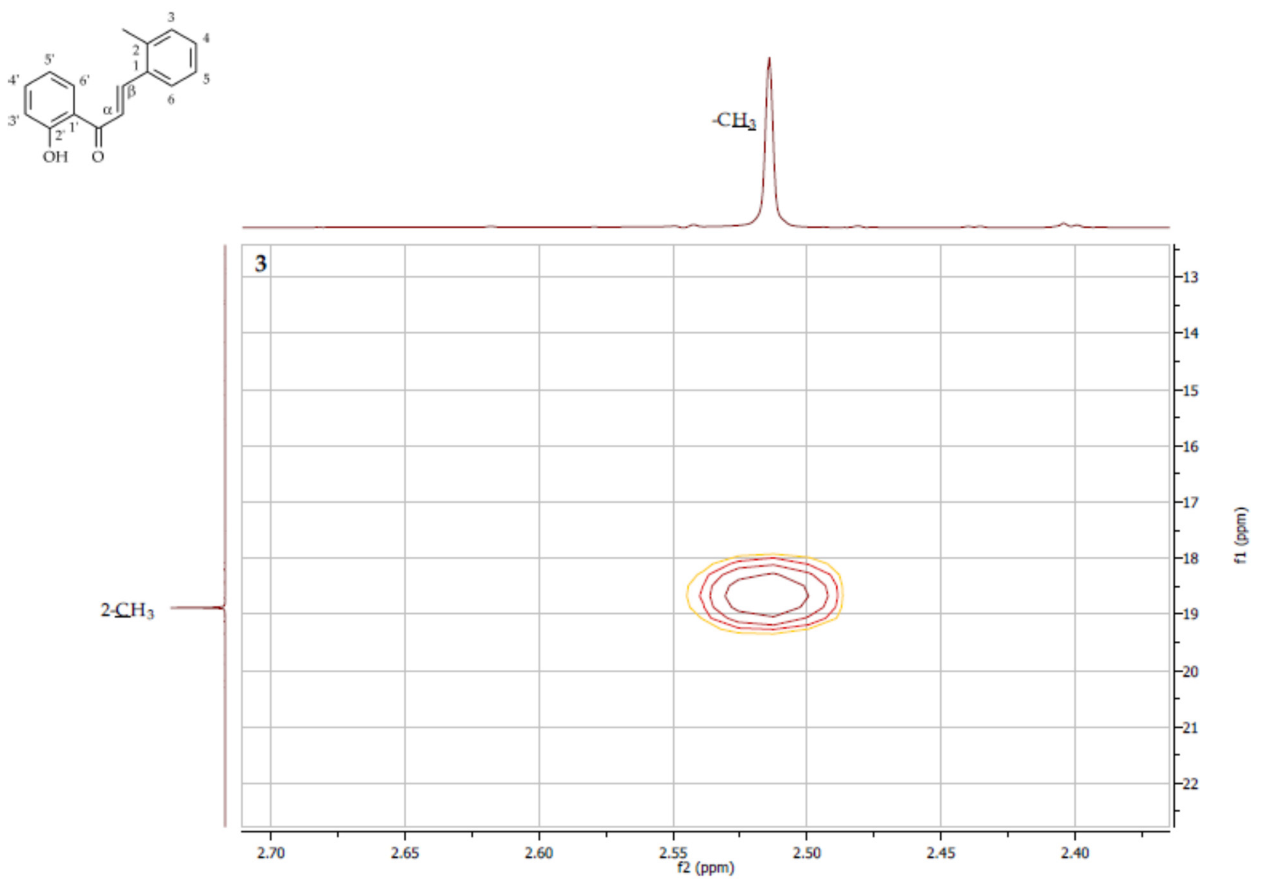

Figure S13. HSQC contour map– ^1H x ^{13}C expansion of 2′-hydroxy-2-methylchalcone (**3**)

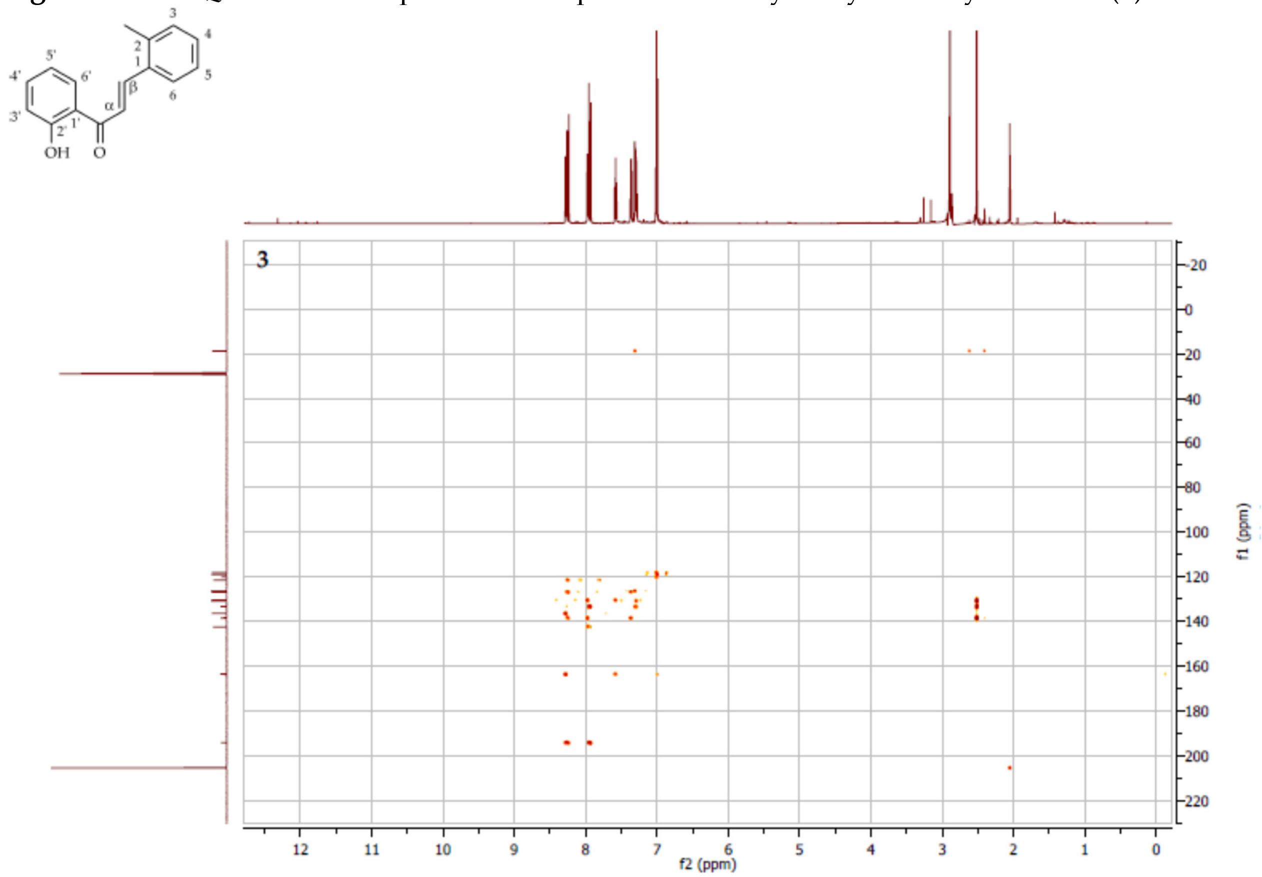

Figure S14. HMBC contour map– ^1H x ^{13}C of 2′-hydroxy-2-methylchalcone (**3**)

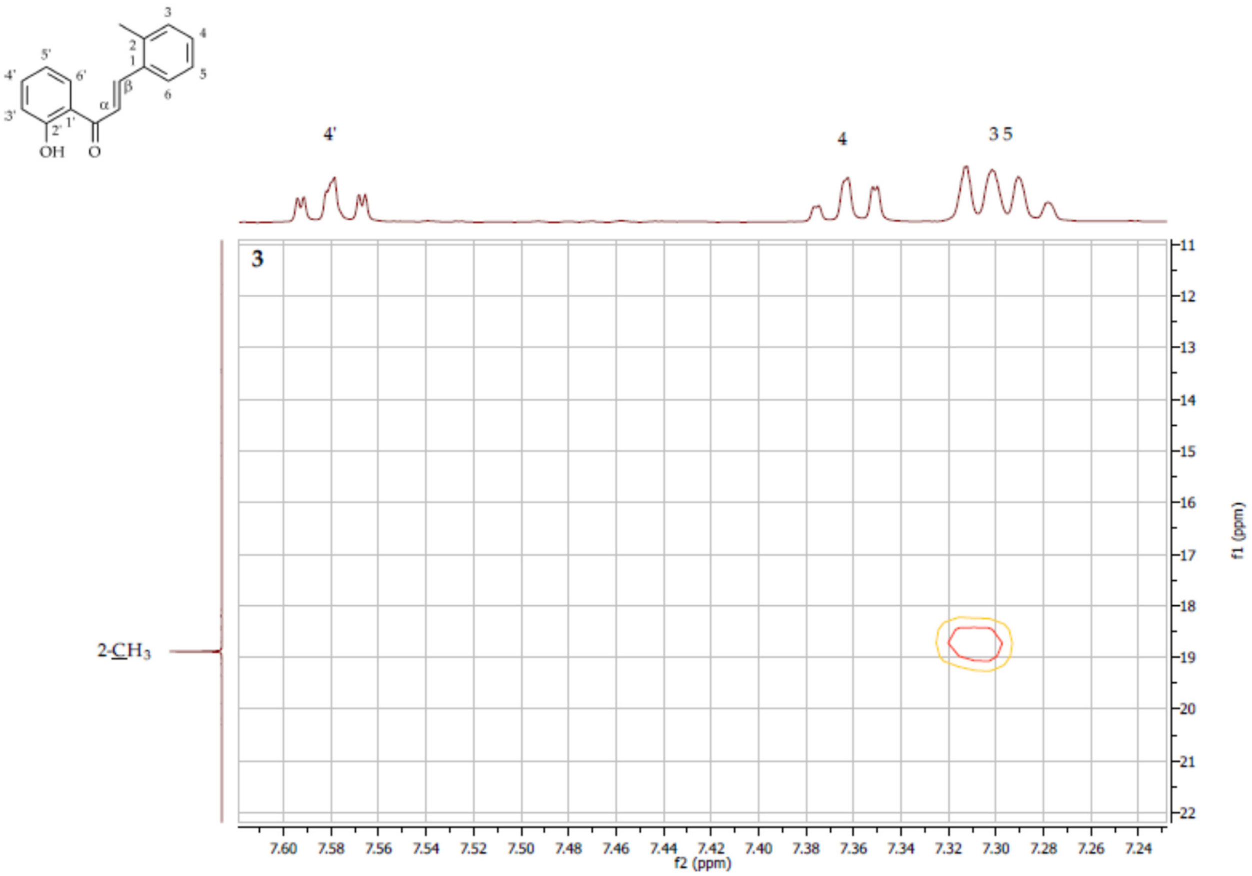

Figure S15. HMBC contour map– ^1H x ^{13}C expansion of 2′-hydroxy-2-methylchalcone (**3**)

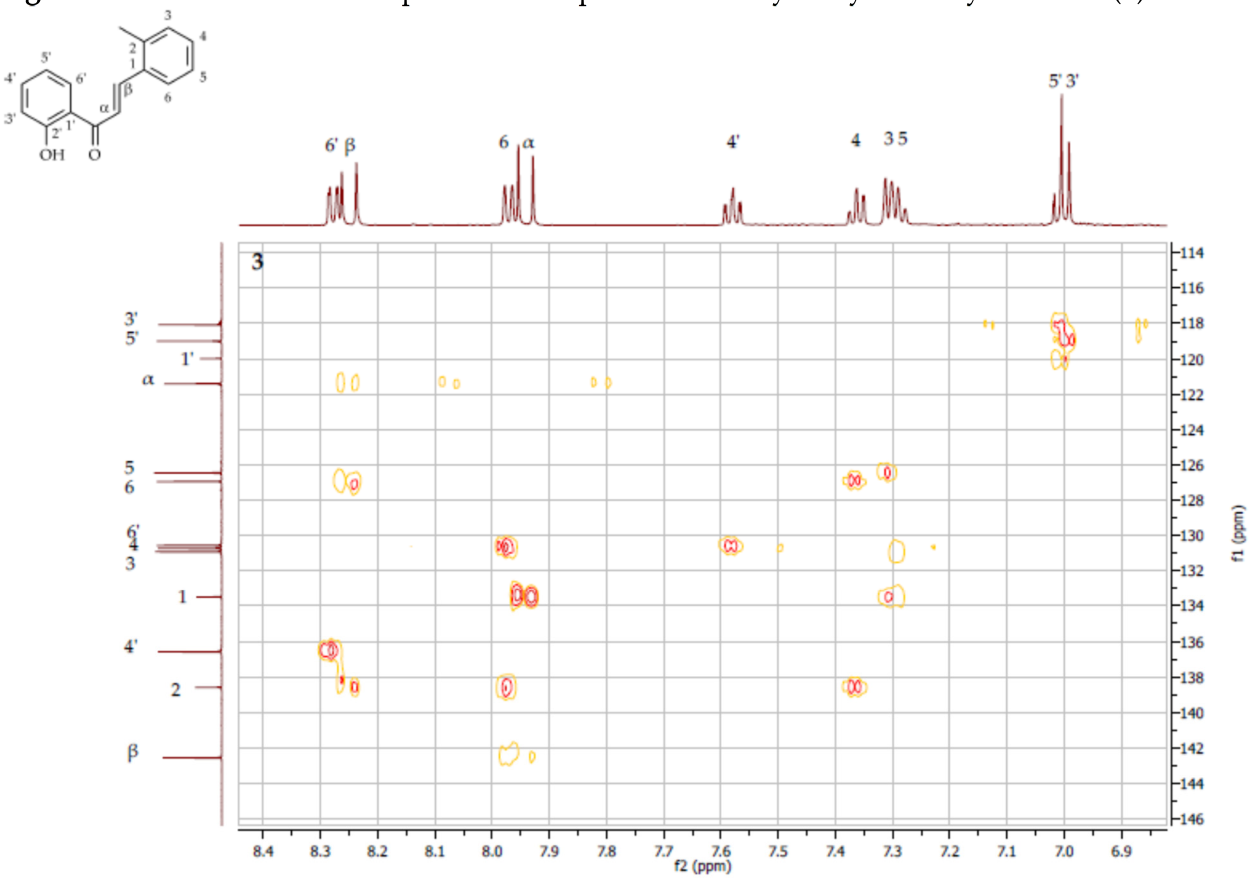

Figure S16. HMBC contour map– ^1H x ^{13}C expansion of 2′-hydroxy-2-methylchalcone (**3**)

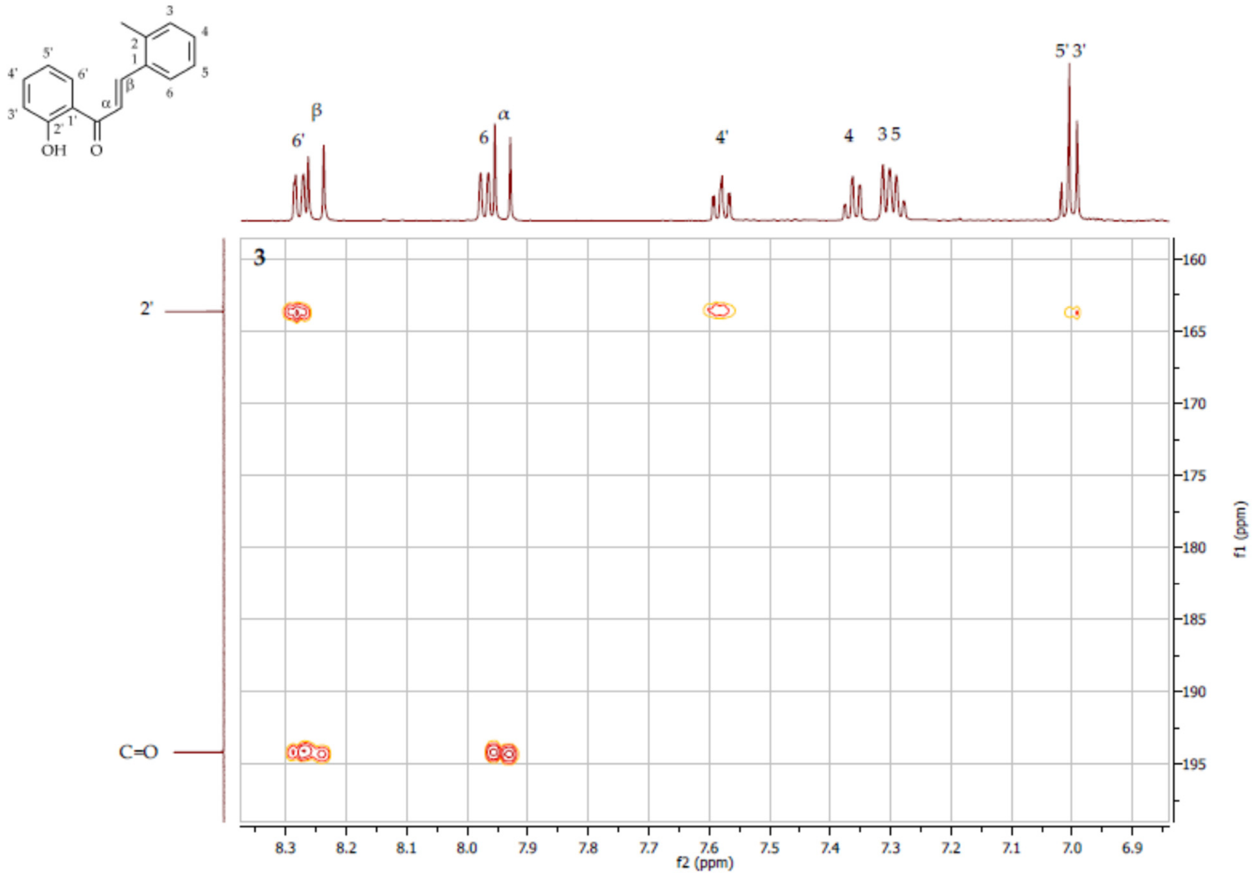

Figure S17. HMBC contour map– ^{1}H x ^{13}C expansion of 2′-hydroxy-2-methylchalcone (**3**)

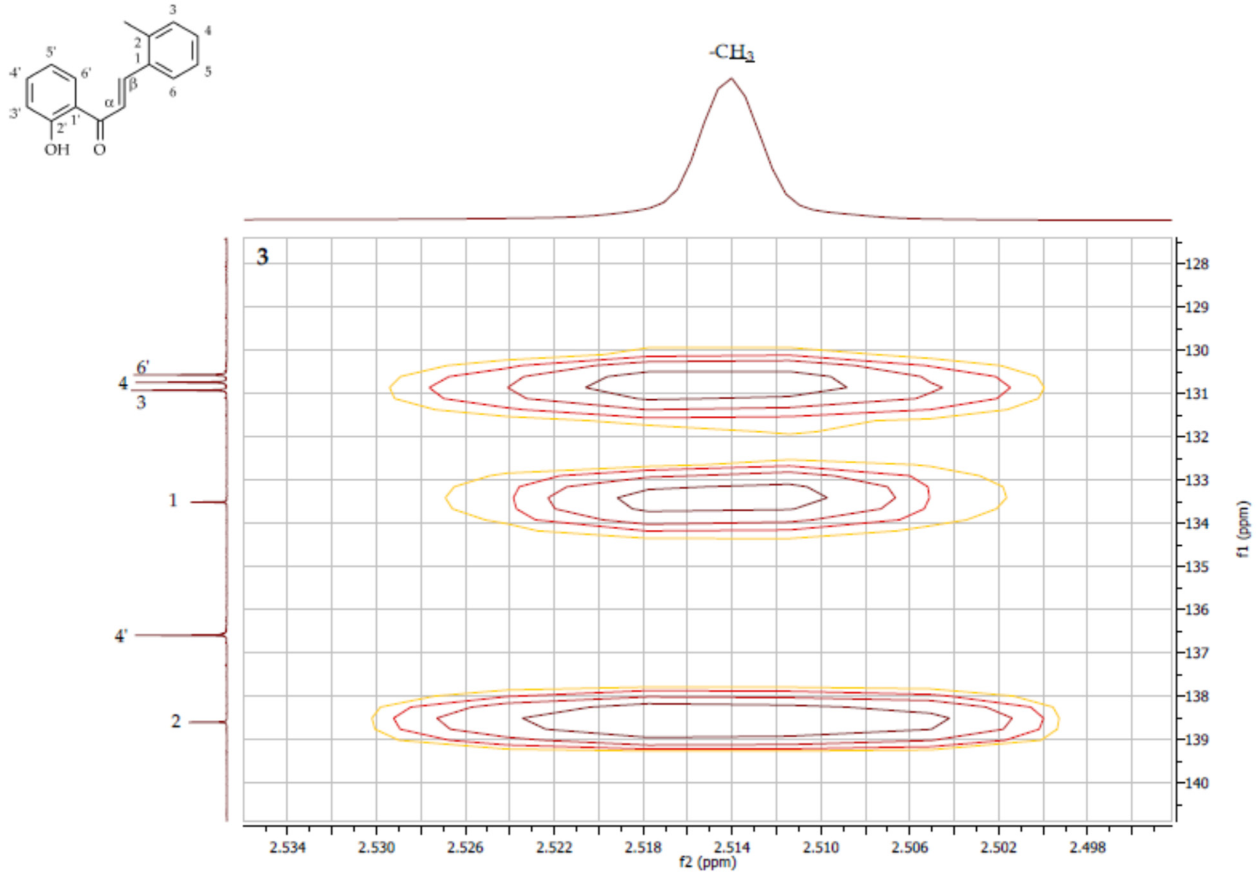

Figure S18. HMBC contour map– ^{1}H x ^{13}C expansion of 2′-hydroxy-2-methylchalcone (**3**)

Molecular formula: $C_{23}H_{28}O_8$
Formula weight: 432.18
Ionization mode: negative
Precursor: $[M-H]^-$ 431.30
431.3000>255.1000 CE: 26.0
431.3000>108.0000 CE: 48.0
431.3000>235.0500 CE: 47.0

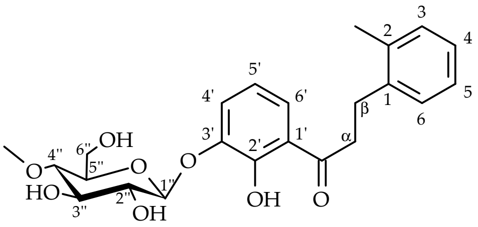

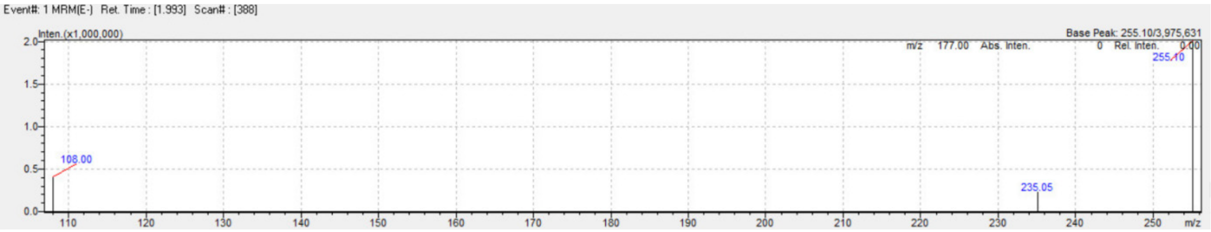

Figure S19. MS analysis of 2′-hydroxy-2-methyldihydrochalcone 3′-*O*-*β*-D-(4′′-*O*-methyl)-glucopyranoside (**3a**)

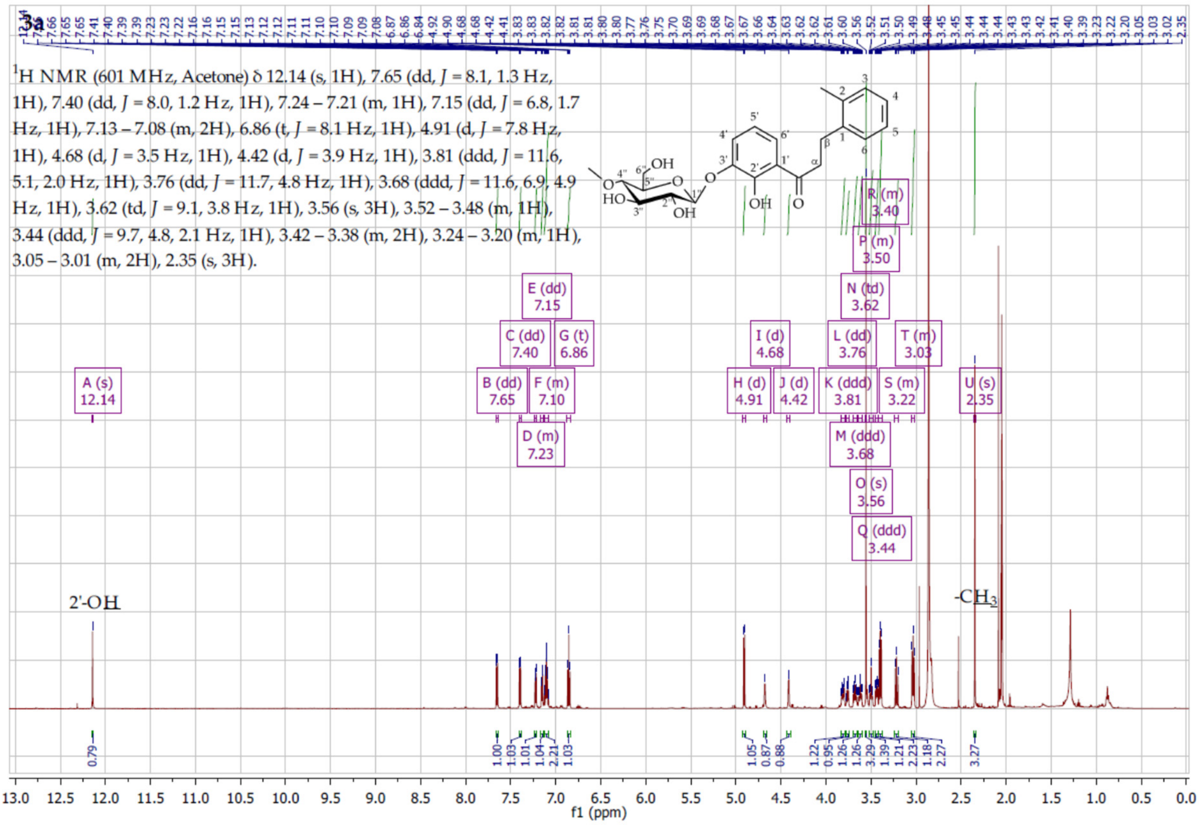

Figure S20. ^{1}H NMR spectrum (*δ*, acetone-d6, 600 MHz) of 2′-hydroxy-2-methyldihydrochalcone 3′-*O*-*β*-D-(4′′-*O*-methyl)-glucopyranoside (**3a**)

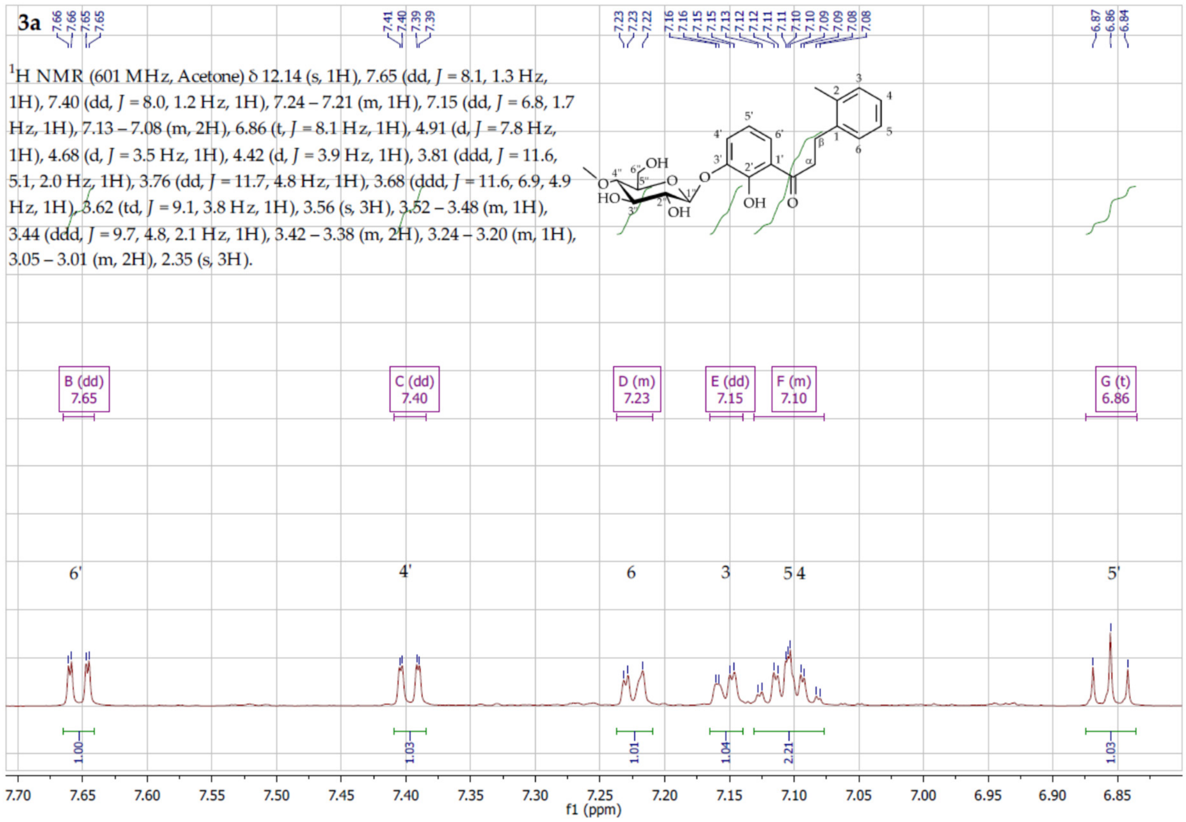

Figure S21. ^{1}H NMR spectrum expansion (*δ*, acetone-d6, 600 MHz) of 2′-hydroxy-2-methyldihydrochalcone 3′-*O*-*β*-D-(4′′-*O*-methyl)-glucopyranoside (**3a**)

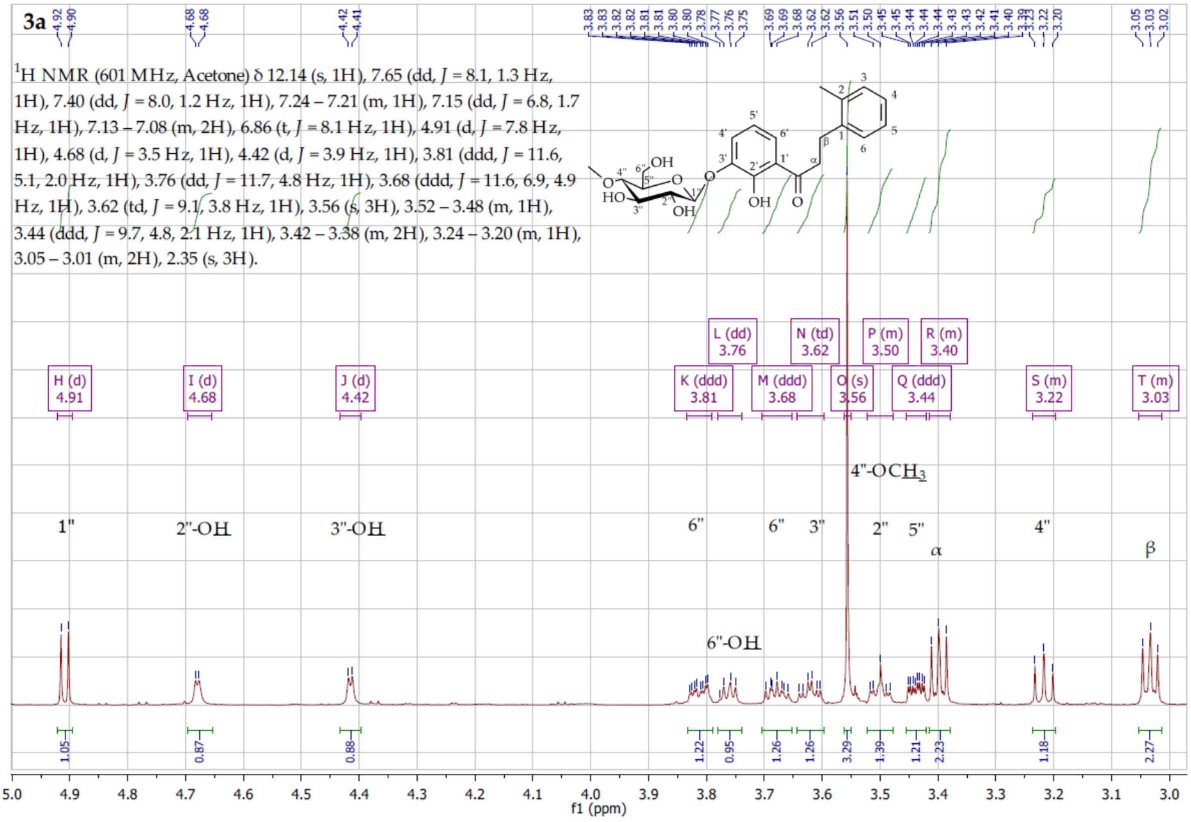

Figure S22. ^{1}H NMR spectrum expansion (*δ*, acetone-d6, 600 MHz) of 2′-hydroxy-2-methyldihydrochalcone 3′-*O*-*β*-D-(4′′-*O*-methyl)-glucopyranoside (**3a**)

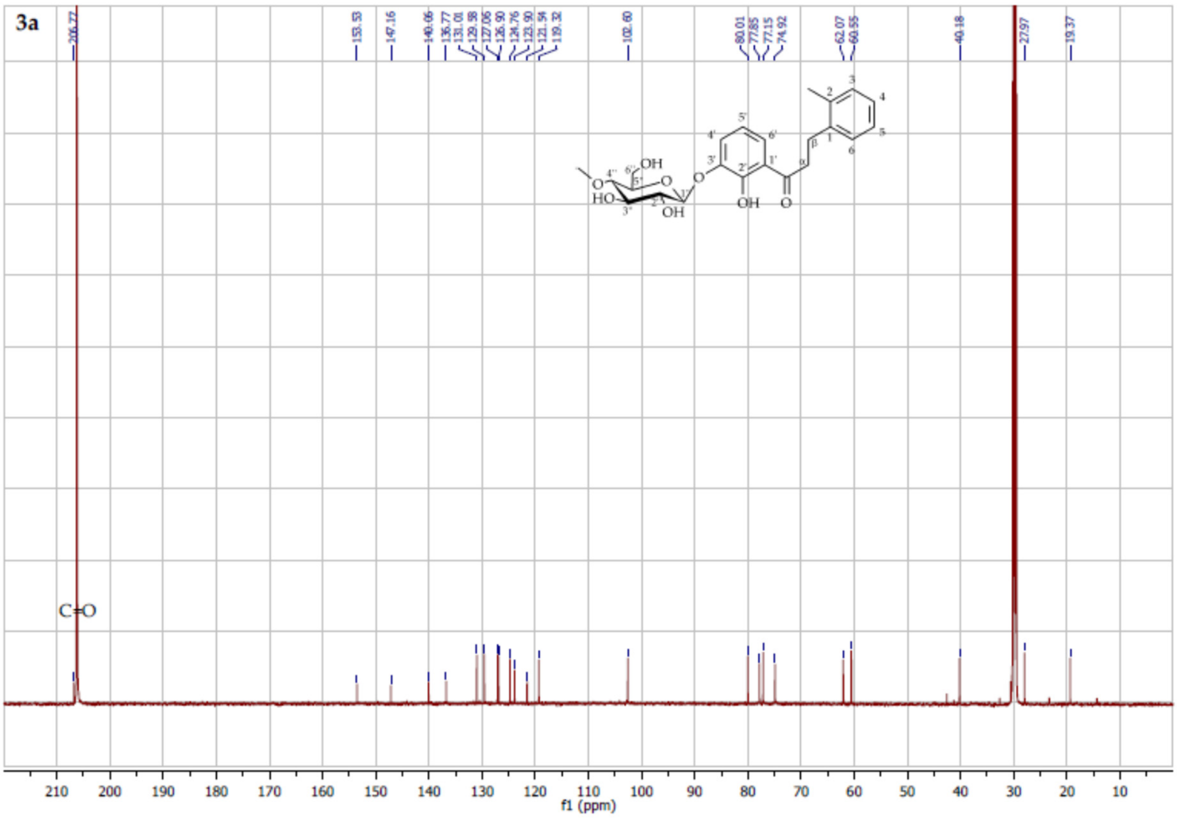

Figure S23. ^{13}C NMR spectrum (*δ*, acetone-d6, 151 MHz) of 2′-hydroxy-2-methyldihydrochalcone 3′-*O*-*β*-D-(4′′-*O*-methyl)-glucopyranoside (**3a**)

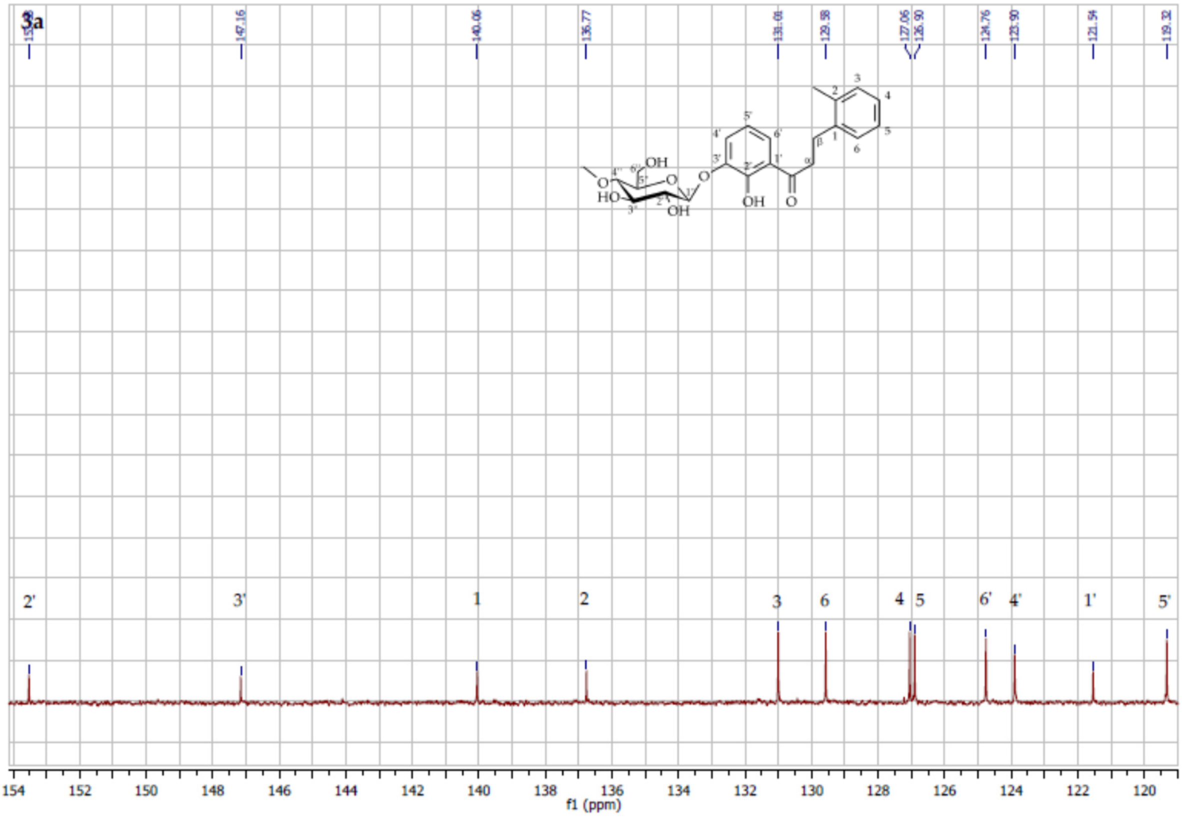

Figure S24. ^{13}C NMR spectrum expansion (*δ*, acetone-d6, 151 MHz) of 2′-hydroxy-2-methyldihydrochalcone 3′-*O*-*β*-D-(4′′-*O*-methyl)-glucopyranoside (**3a**)

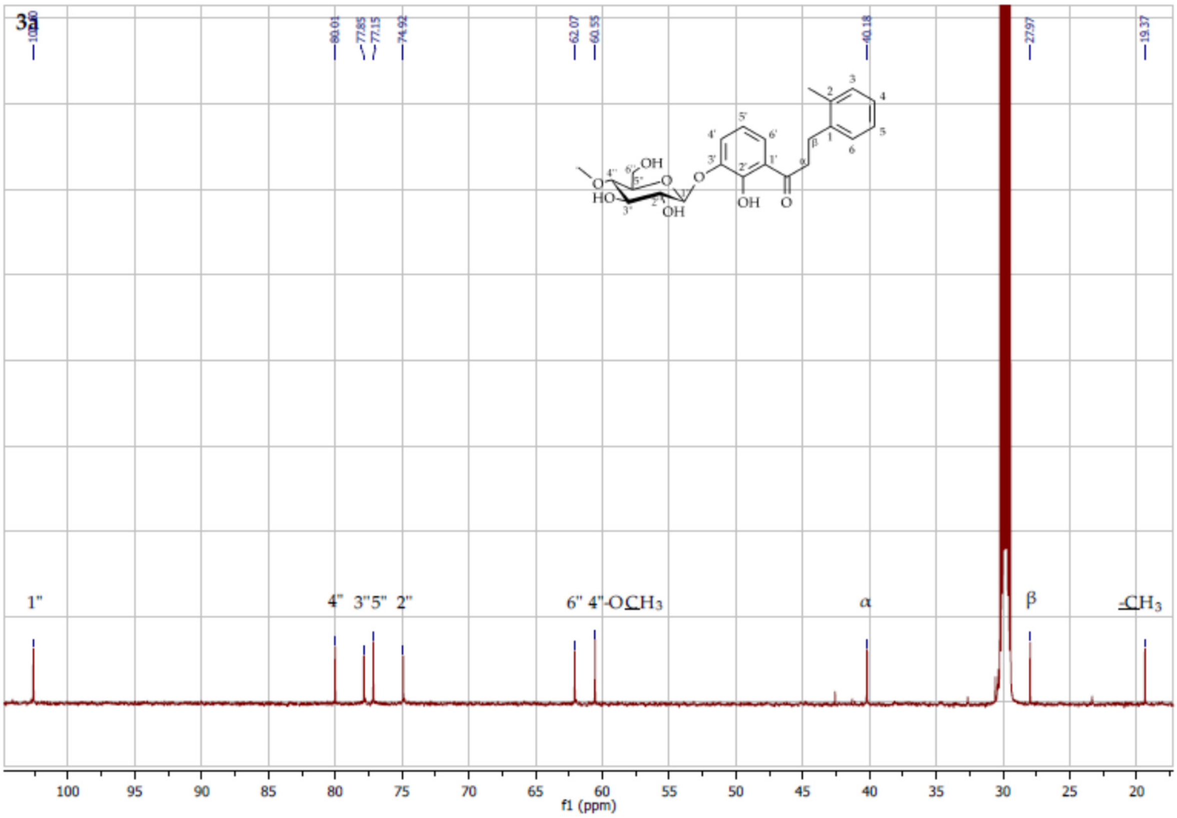

Figure S25. ^{13}C NMR spectrum expansion (*δ*, acetone-d6, 600 MHz) of 2′-hydroxy-2-methyldihydrochalcone 3′-*O*-*β*-D-(4′′-*O*-methyl)-glucopyranoside (**3a**)

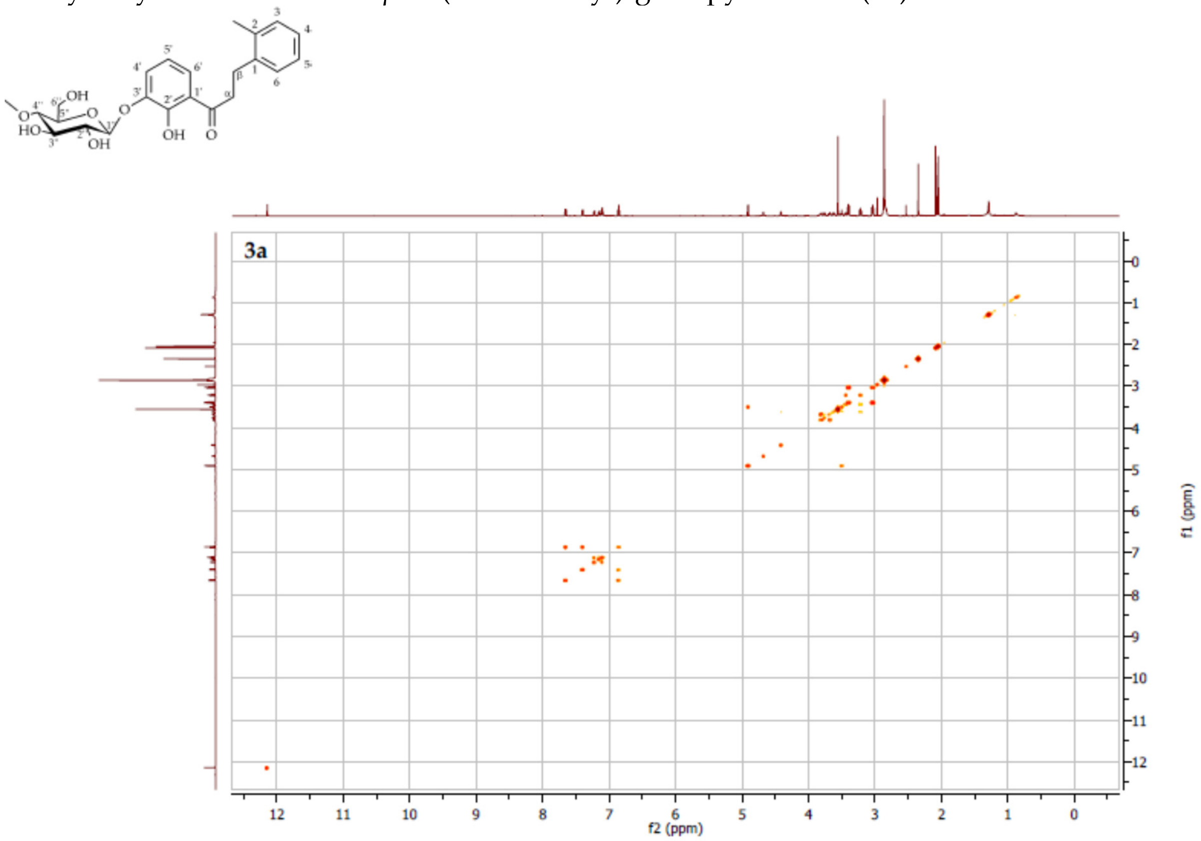

Figure S26. COSY contour map – ^{1}H x $^{1}H$of 2′-hydroxy-2-methyldihydrochalcone 3′-*O*-*β*-D-(4′′-*O*-methyl)-glucopyranoside (**3a**)

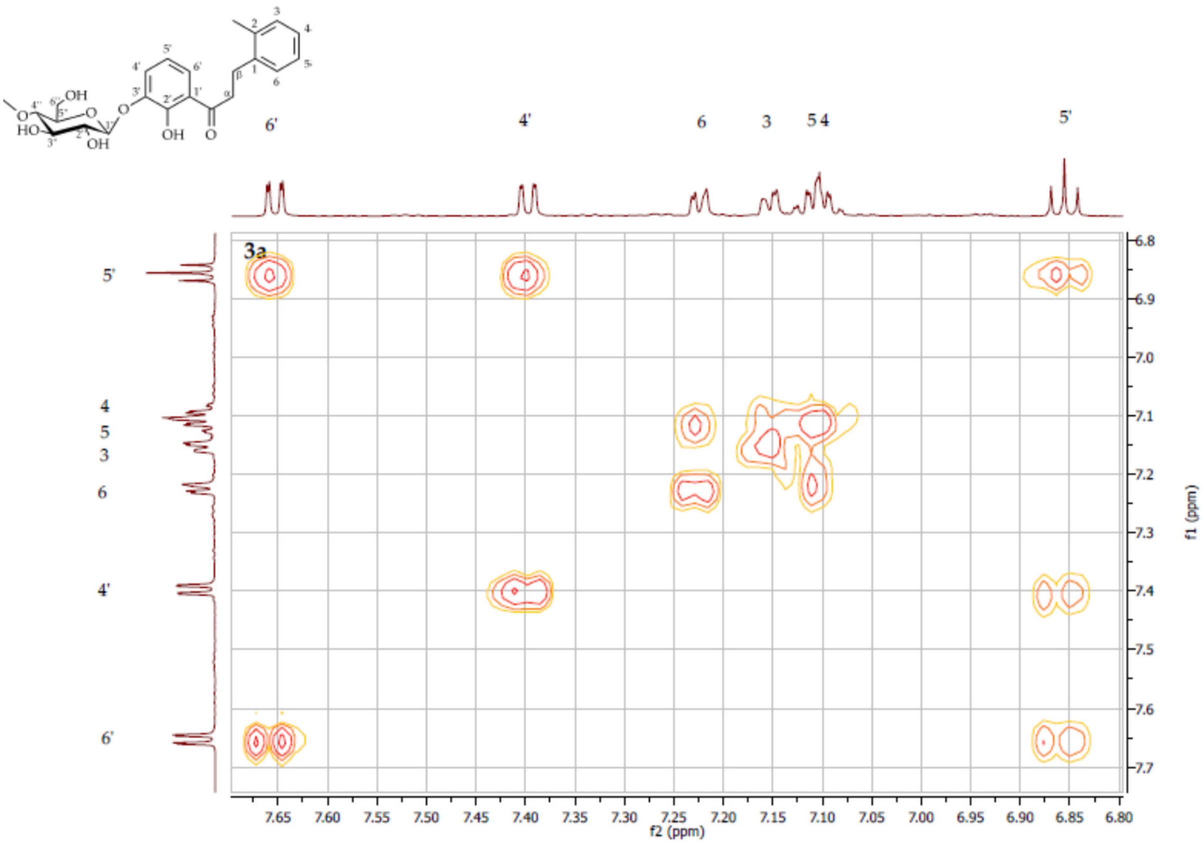

Figure S27. COSY contour map – $^1H \times {}^1H$ expansion of 2′-hydroxy-2-methyldihydrochalcone 3′-*O*-*β*-D-(4′′-*O*-methyl)-glucopyranoside (**3a**)

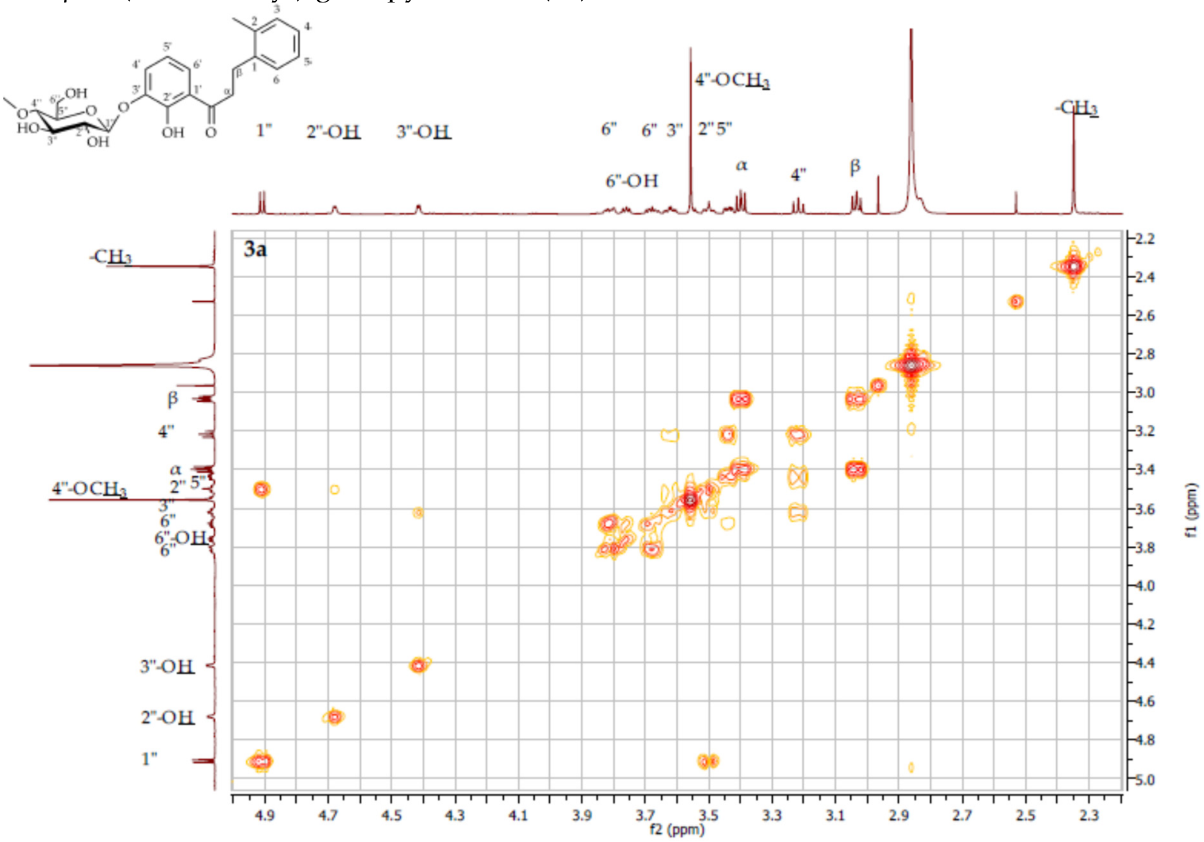

Figure S28. COSY contour map – $^1H \times {}^1H$ expansion of 2′-hydroxy-2-methyldihydrochalcone 3′-*O*-*β*-D-(4′′-*O*-methyl)-glucopyranoside (**3a**)

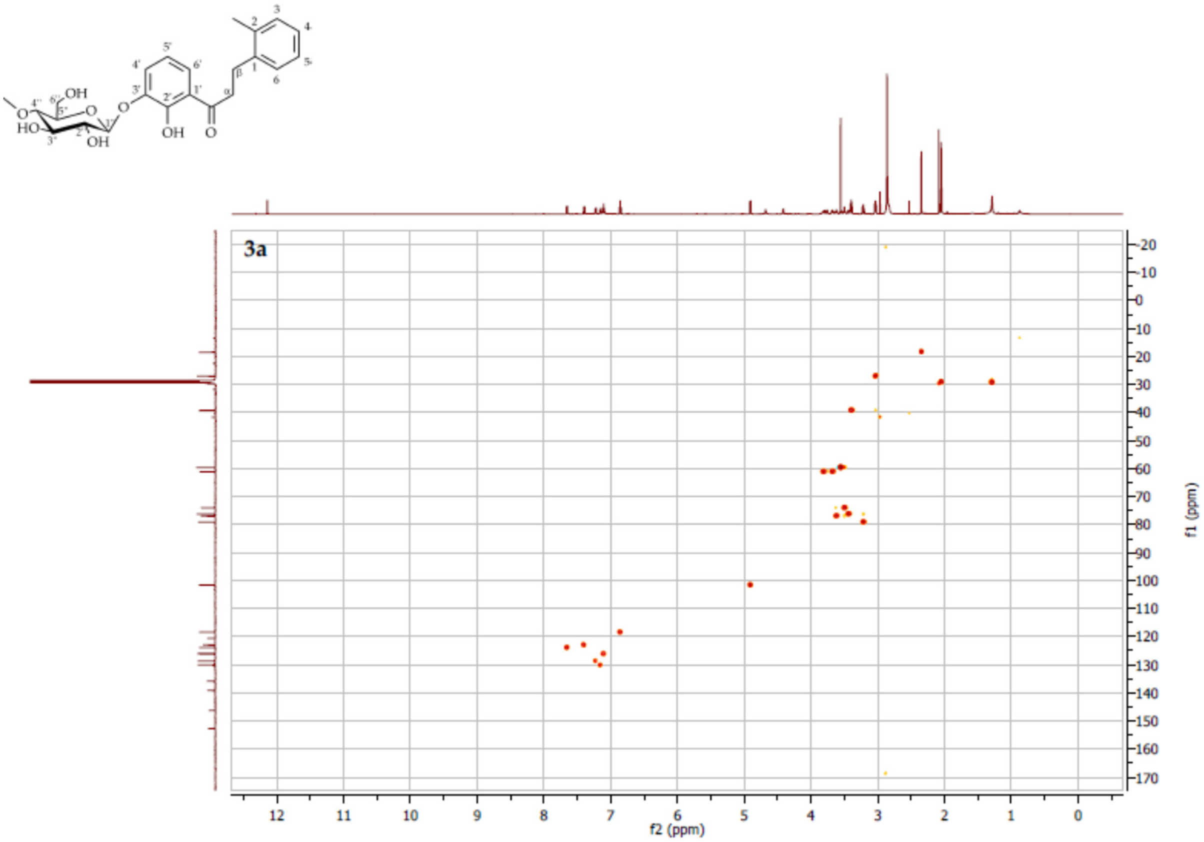

Figure S29. HSQC contour map– ^{1}H x ^{13}C of 2′-hydroxy-2-methyldihydrochalcone 3′-*O*-*β*-D-(4′′-*O*-methyl)-glucopyranoside (**3a**)

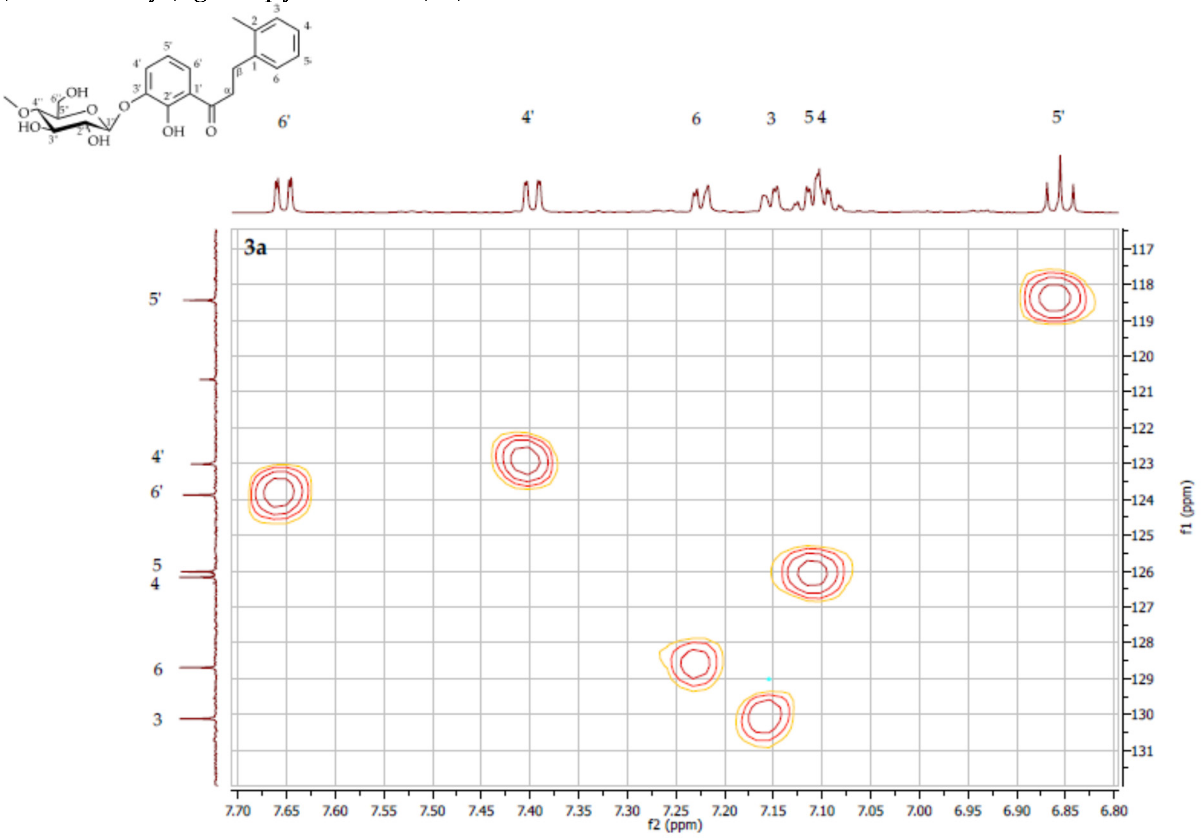

Figure S30. HSQC contour map– ^{1}H x ^{13}C expansion of 2′-hydroxy-2-methyldihydrochalcone 3′-*O*-*β*-D-(4′′-*O*-methyl)-glucopyranoside (**3a**)

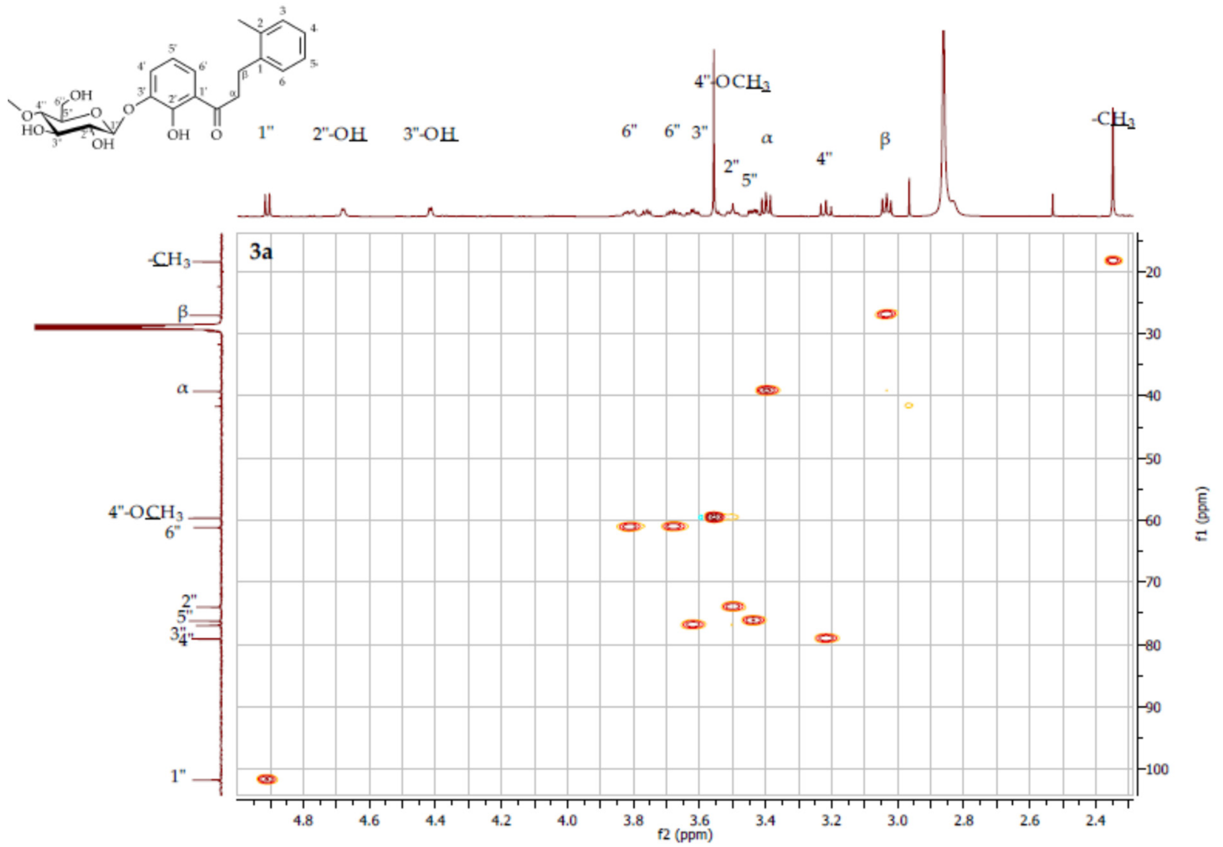

Figure S31. HSQC contour map– ^{1}H x ^{13}C expansion of 2′-hydroxy-2-methyldihydrochalcone 3′-*O*-*β*-D-(4′′-*O*-methyl)-glucopyranoside (**3a**)

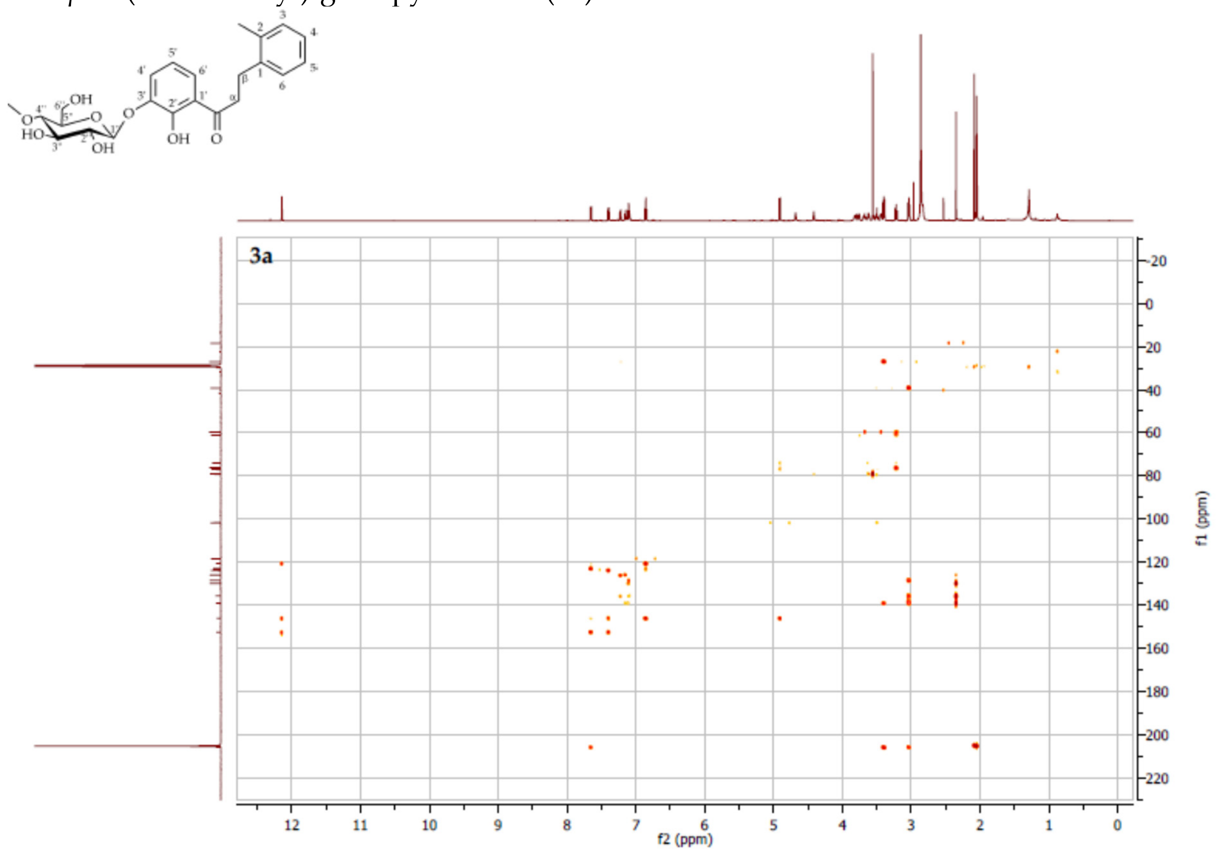

Figure S32. HMBC contour map– ^{1}H x ^{13}C of 2′-hydroxy-2-methyldihydrochalcone 3′-*O*-*β*-D-(4′′-*O*-methyl)-glucopyranoside (**3a**)

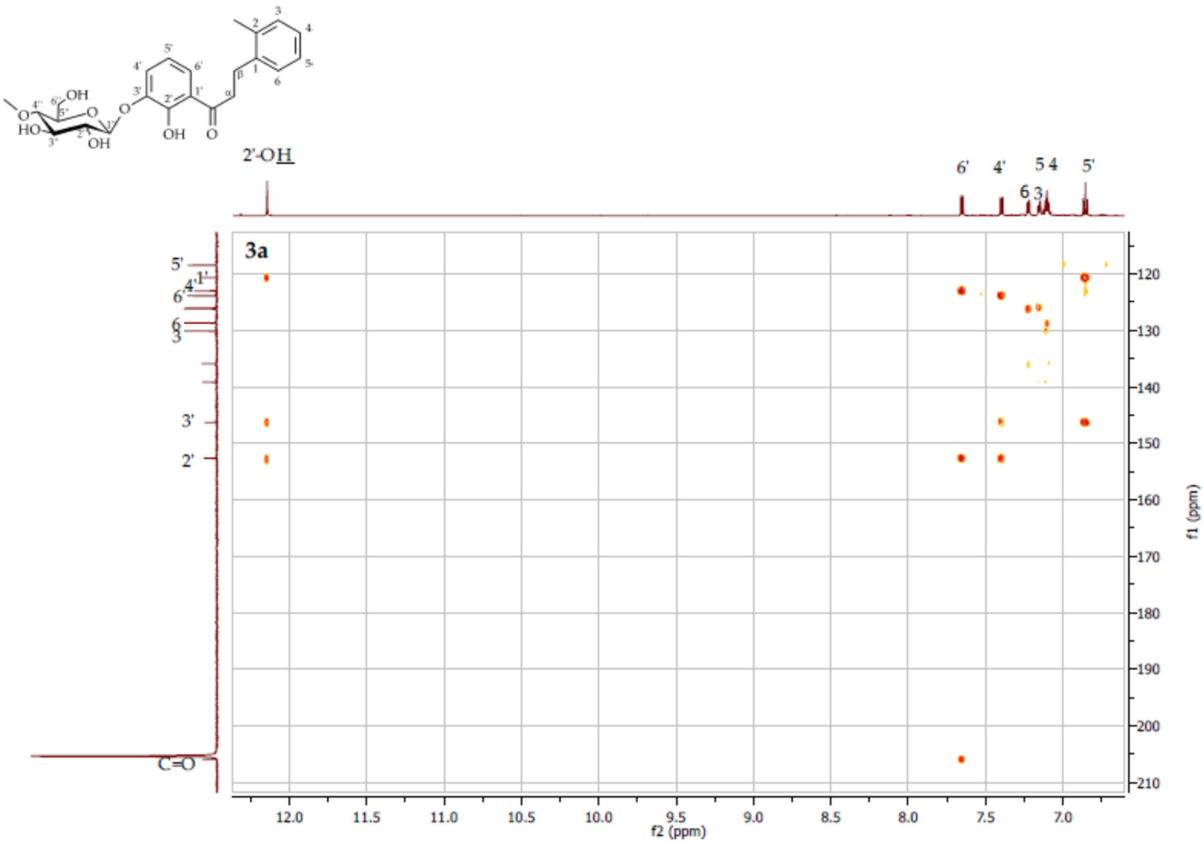

Figure S33. HMBC contour map– ^{1}H x ^{13}C expansion of 2′-hydroxy-2-methyldihydrochalcone 3′-*O*-*β*-D-(4′′-*O*-methyl)-glucopyranoside (**3a**)

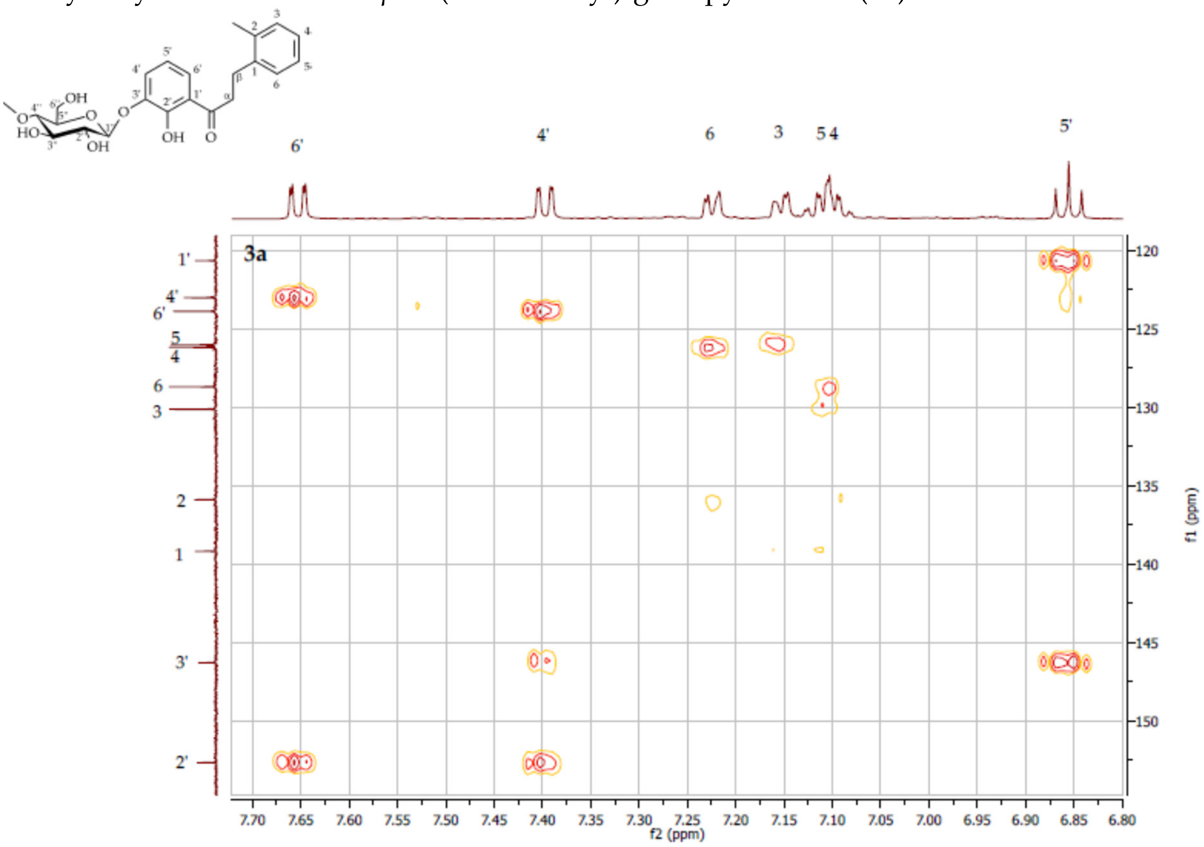

Figure S34. HMBC contour map– ^{1}H x ^{13}C expansion of 2′-hydroxy-2-methyldihydrochalcone 3′-*O*-*β*-D-(4′′-*O*-methyl)-glucopyranoside (**3a**)

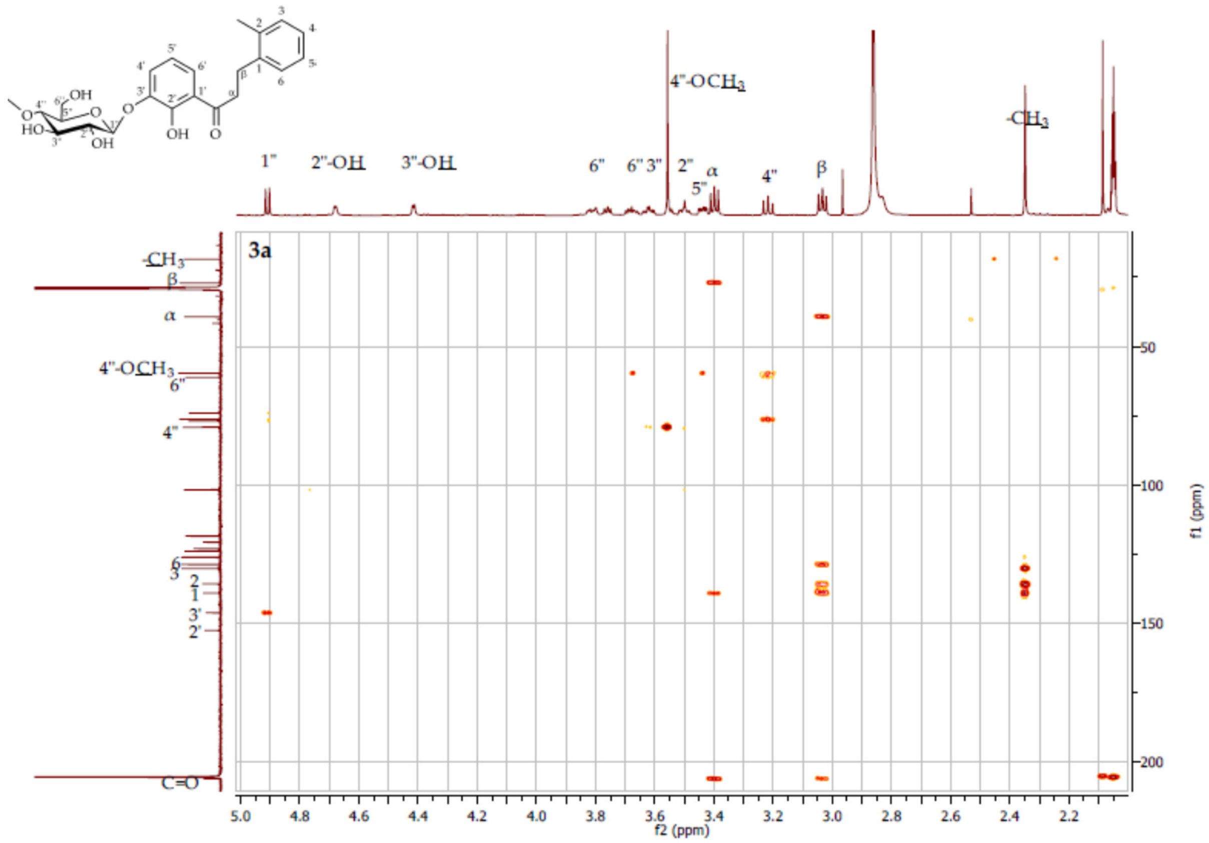

Figure S35. HMBC contour map– ^{1}H x ^{13}C expansion of 2′-hydroxy-2-methyldihydrochalcone 3′-*O*-*β*-D-(4′′-*O*-methyl)-glucopyranoside (**3a**)

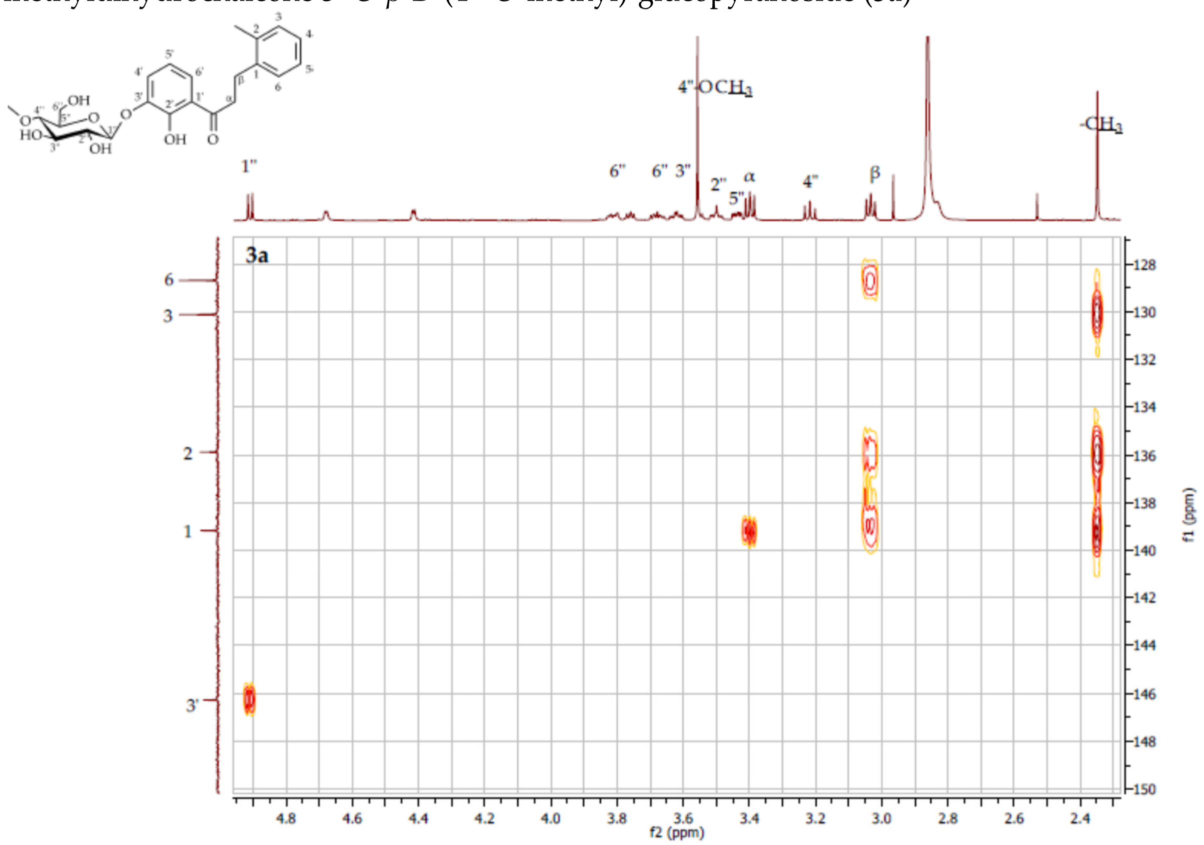

Figure S36. HMBC contour map– ^{1}H x ^{13}C expansion of 2′-hydroxy-2-methyldihydrochalcone 3′-*O*-*β*-D-(4′′-*O*-methyl)-glucopyranoside (**3a**)

Molecular formula: $C_{23}H_{28}O_9$
Formula weight:448.17
Ionization mode:negative
Precursor: $[M-H]^-$ 447.30
447.3000>253.1000 CE: 29.0
447.3000>238.0500 CE: 47.0
447.3000>271.0000 CE: 21.0

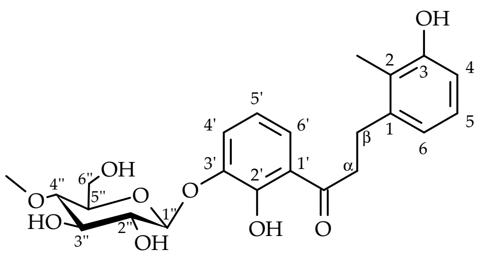

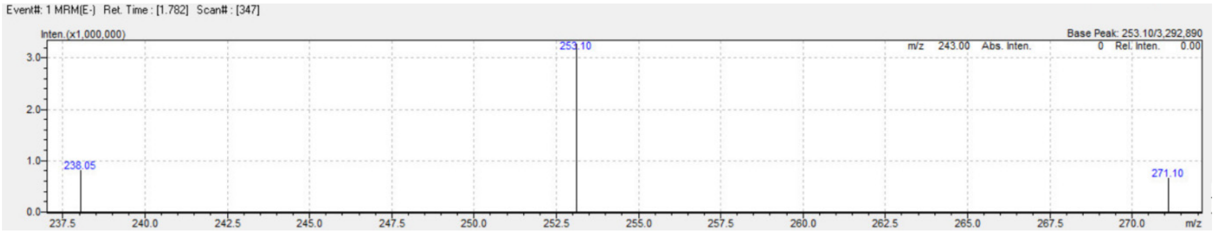

Figure S37.MS analysis of 2′, 3-dihydroxy-2-methyldihydrochalcone 3′-*O*-*β*-D-(4′′-*O*-methyl)-glucopyranoside (**3b**)

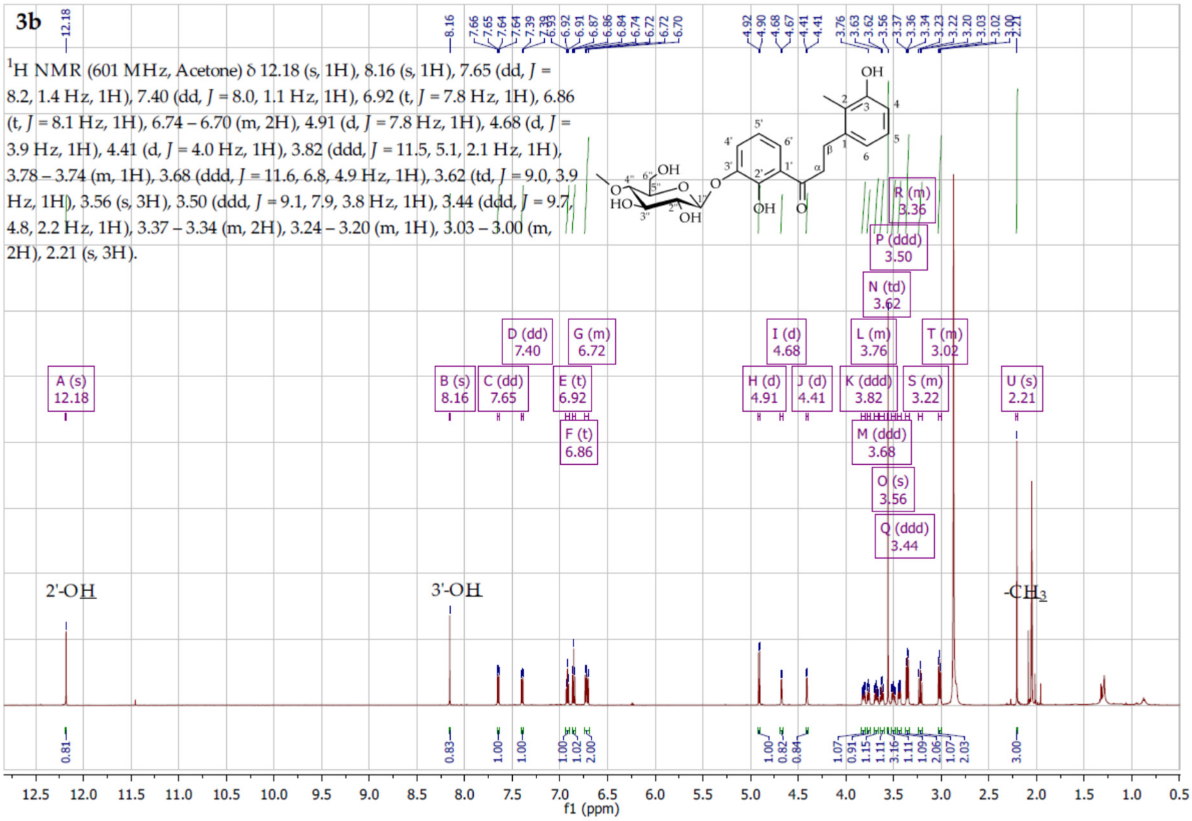

Figure S38. ^{1}H NMR spectrum (*δ*, acetone-d6, 600 MHz) of 2′, 3-dihydroxy-2-methyldihydrochalcone 3′-*O*-*β*-D-(4′′-*O*-methyl)-glucopyranoside (**3b**)

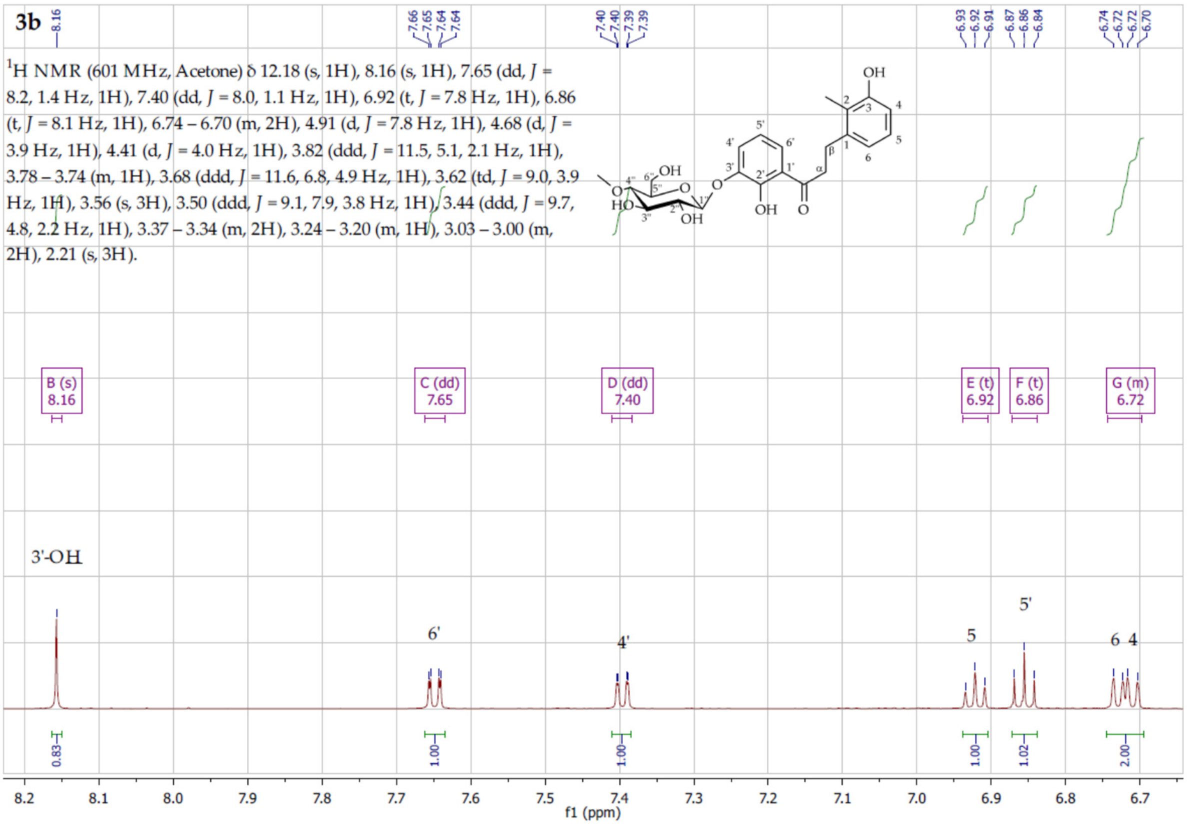

Figure S39. ^{1}H NMR spectrum expansion (*δ*, acetone-d6, 600 MHz) of 2′, 3-dihydroxy-2-methyldihydrochalcone 3′-*O*-*β*-D-(4′′-*O*-methyl)-glucopyranoside (**3b**)

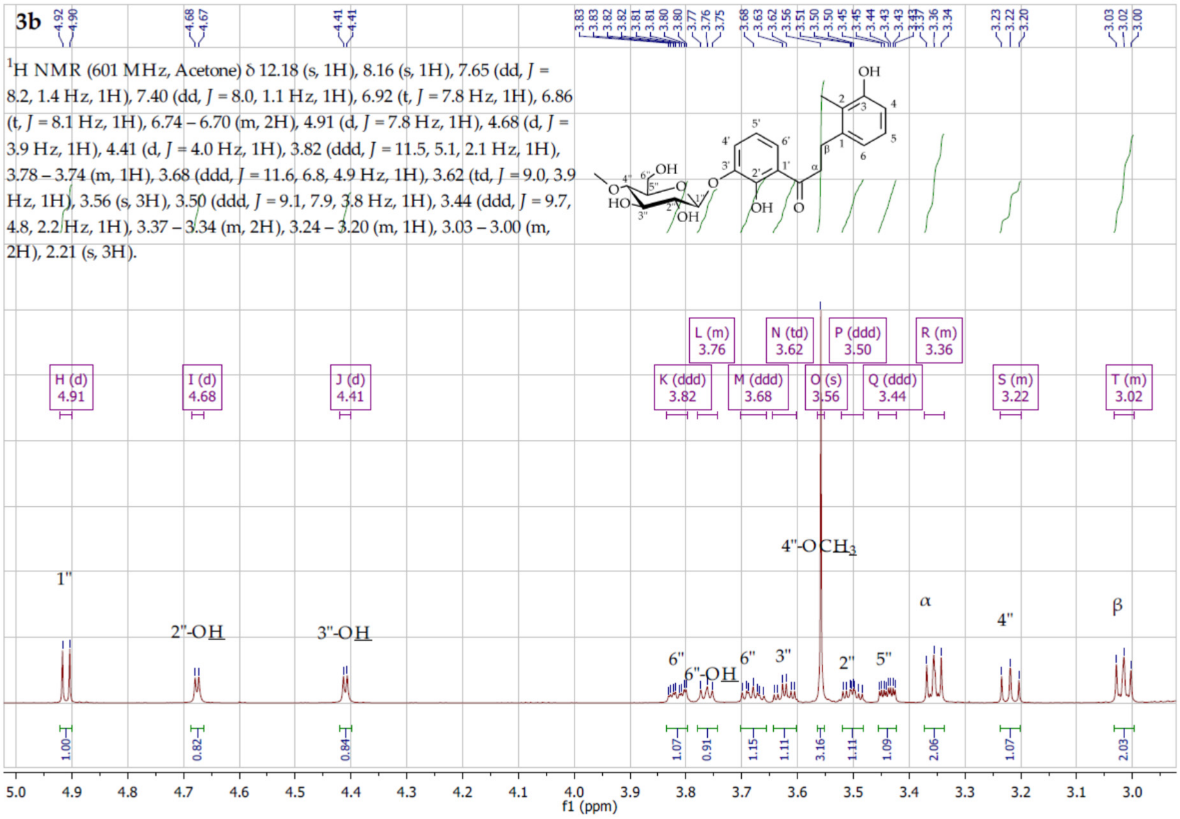

Figure S40. ^{1}H NMR spectrum expansion (*δ*, acetone-d6, 600 MHz) of 2′, 3-dihydroxy-2-methyldihydrochalcone 3′-*O*-*β*-D-(4′′-*O*-methyl)-glucopyranoside (**3b**)

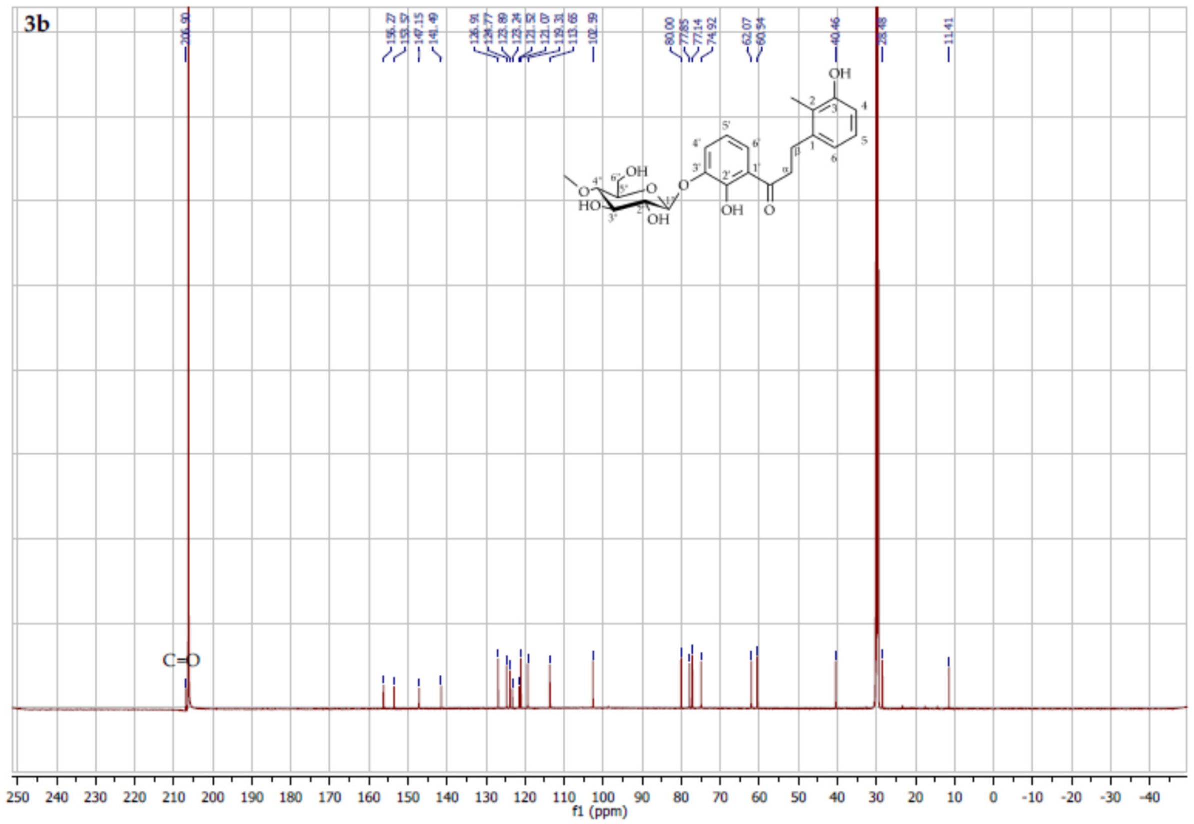

Figure S41. ^{13}C NMR spectrum (*δ*, acetone-d6, 151 MHz) of 2′, 3-dihydroxy-2-methyldihydrochalcone 3′-*O*-*β*-D-(4′′-*O*-methyl)-glucopyranoside (**3b**)

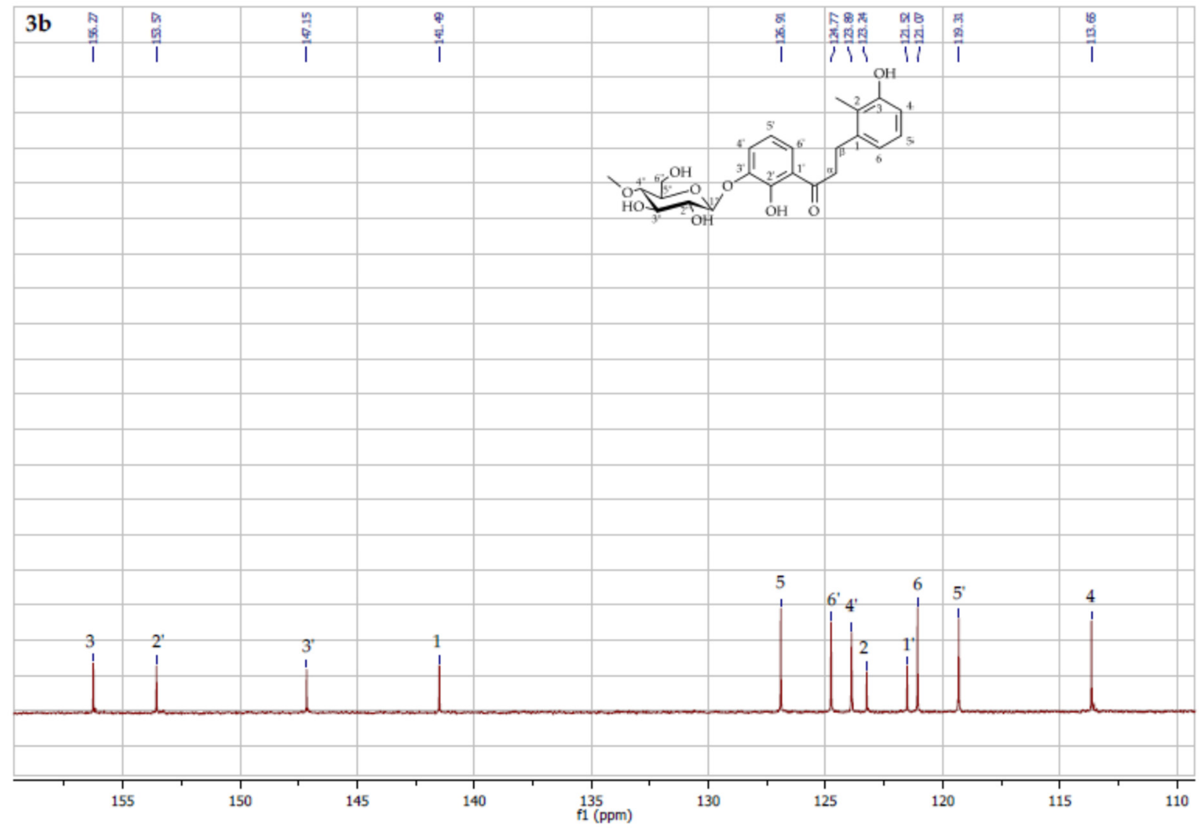

Figure S42. ^{13}C NMR spectrum expansion (*δ*, acetone-d6, 151 MHz) of 2′, 3-dihydroxy-2-methyldihydrochalcone 3′-*O*-*β*-D-(4′′-*O*-methyl)-glucopyranoside (**3b**)

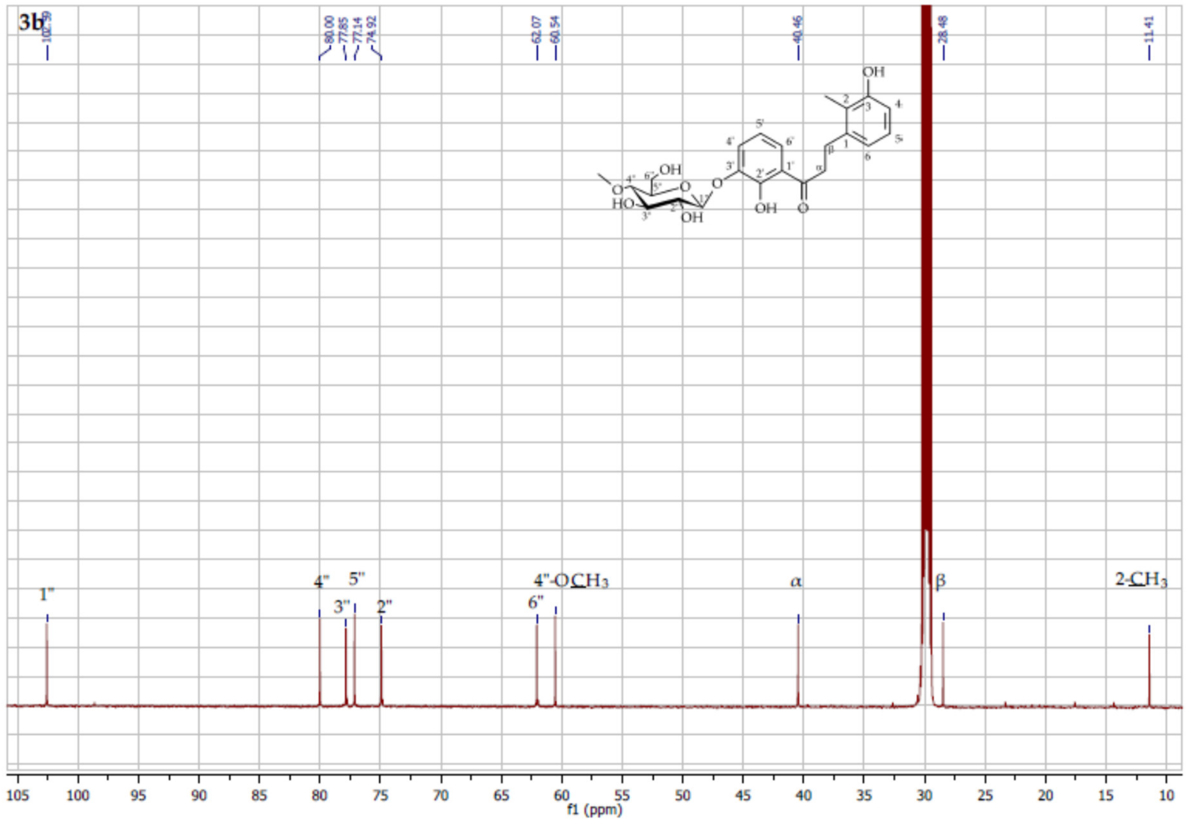

Figure S43. ^{13}C NMR spectrum expansion (δ, acetone-d6, 151 MHz) of 2′, 3-dihydroxy-2-methyldihydrochalcone 3′-*O*-*β*-D-(4′′-*O*-methyl)-glucopyranoside (**3b**)

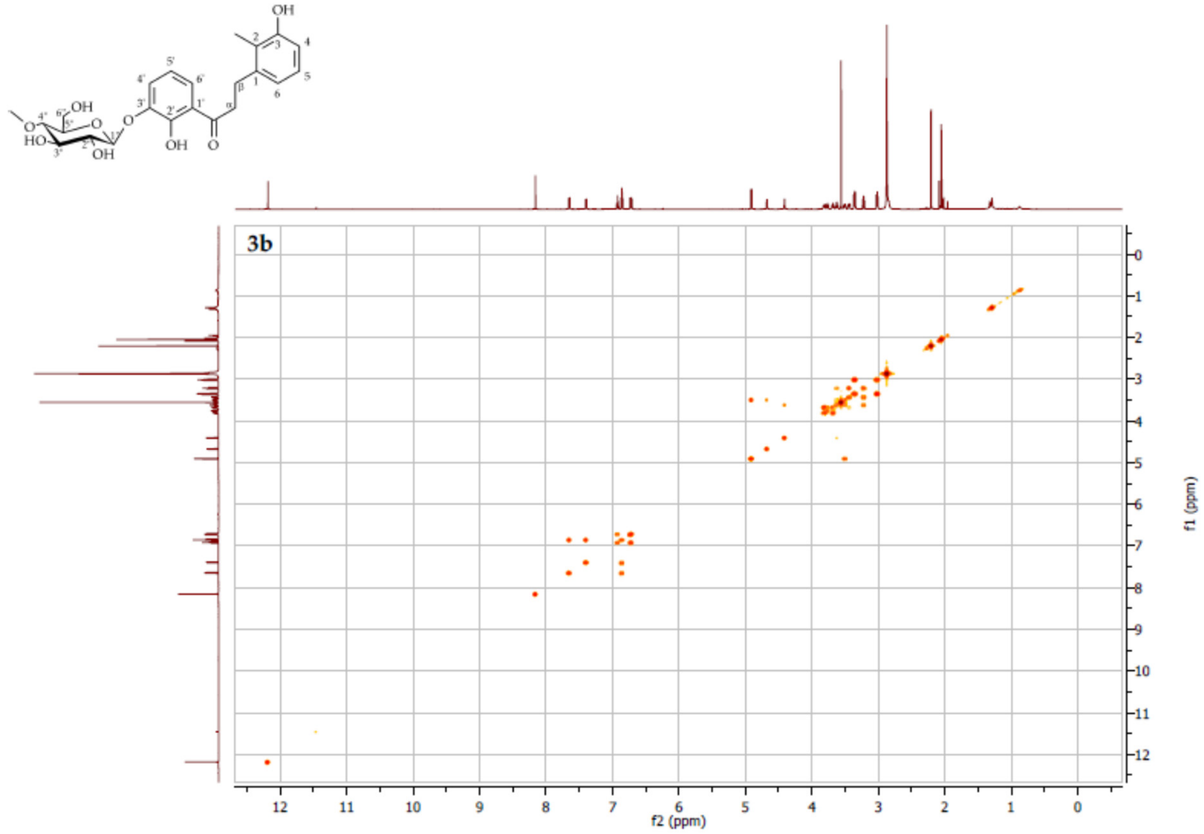

Figure S44. COSY contour map – ^{1}H x $^{1}H$of 2′, 3-dihydroxy-2-methyldihydrochalcone 3′-*O*-*β*-D-(4′′-*O*-methyl)-glucopyranoside (**3b**)

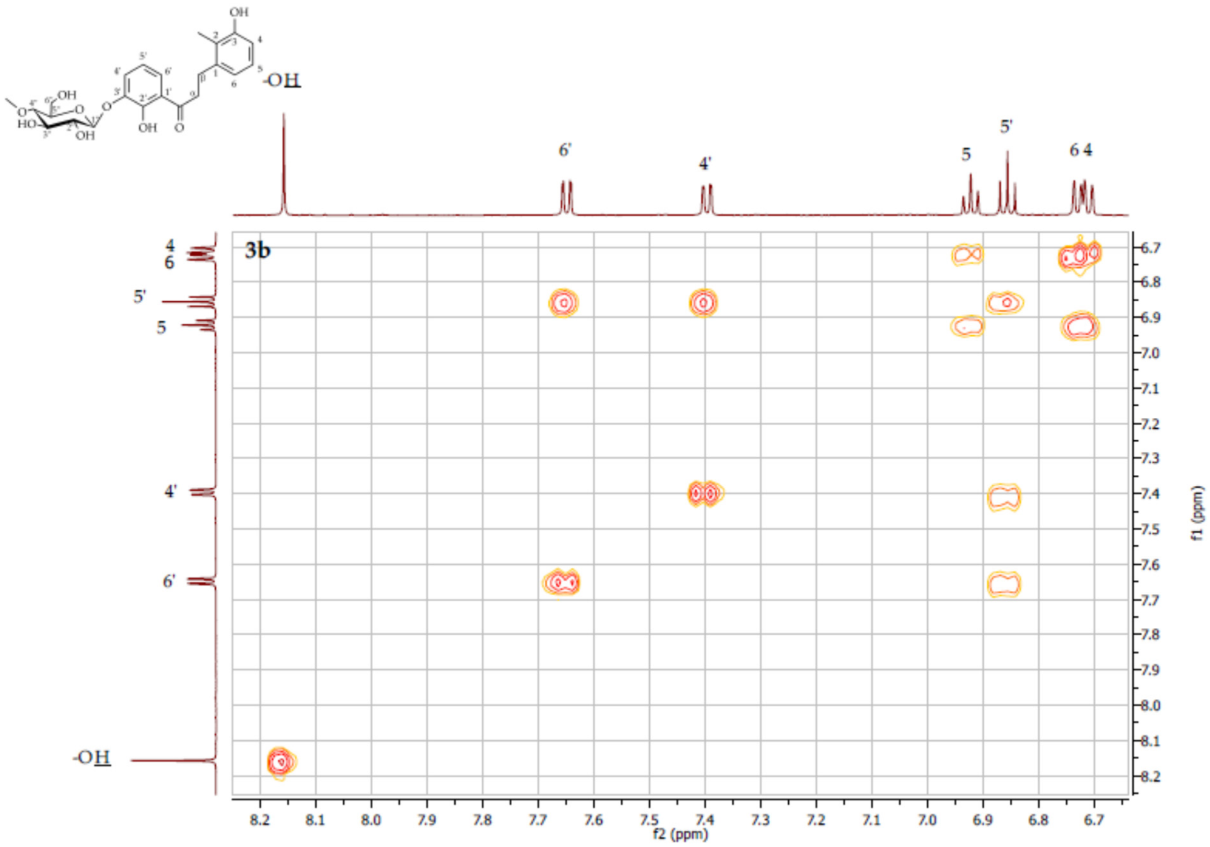

Figure S45.COSY contour map – ¹H x ¹Hexpansion of 2′, 3-dihydroxy-2-methyldihydrochalcone 3′-*O*-*β*-D-(4′′-*O*-methyl)-glucopyranoside (**3b**)

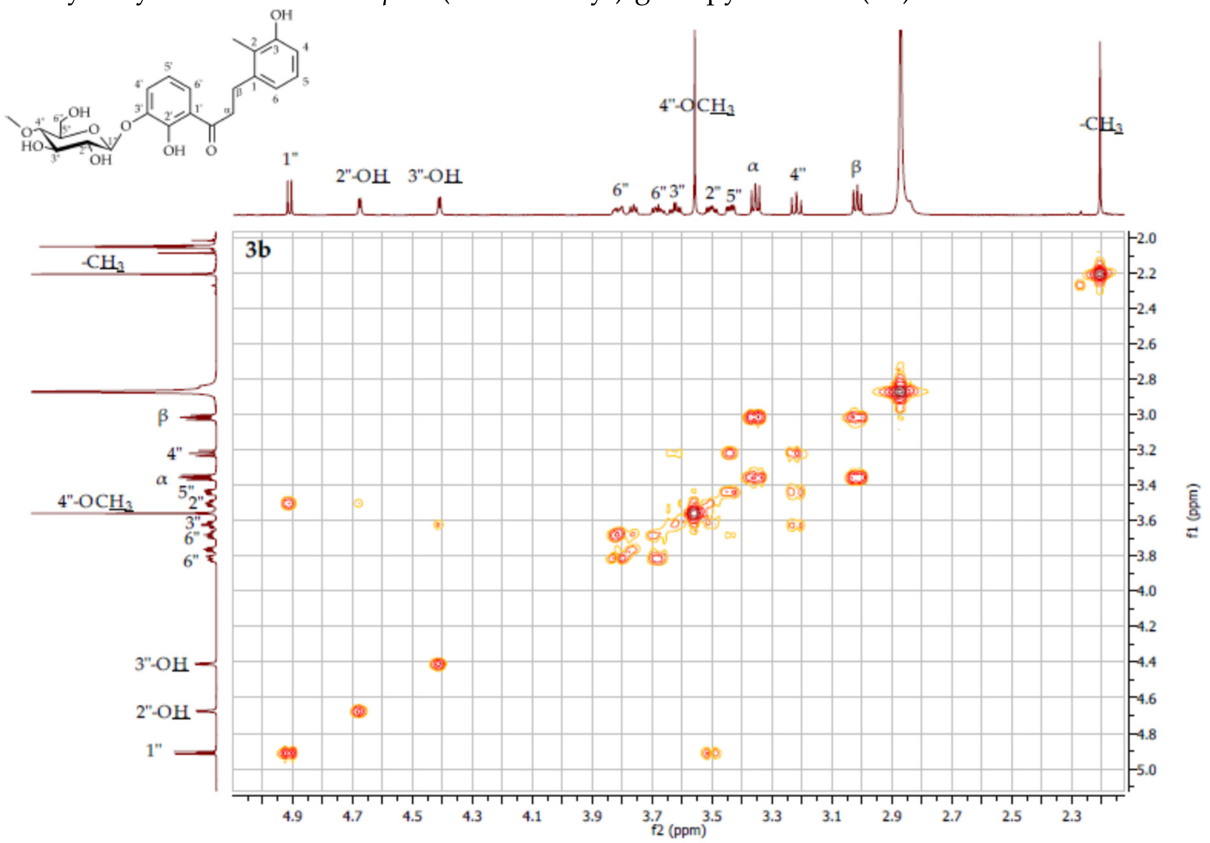

Figure S46. COSY contour map – ¹H x ¹Hexpansion of 2′, 3-dihydroxy-2-methyldihydrochalcone 3′-*O*-*β*-D-(4′′-*O*-methyl)-glucopyranoside (**3b**)

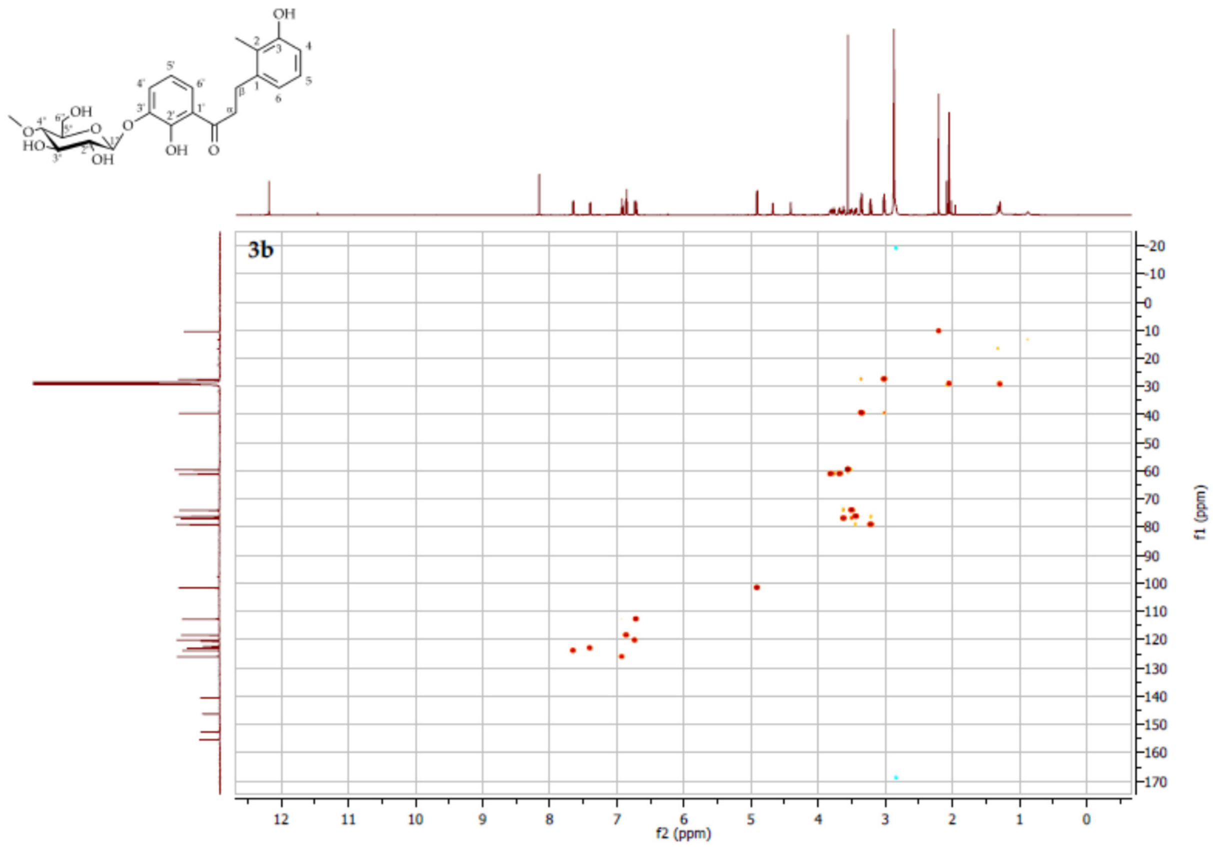

Figure S47. HSQC contour map– ¹H x ¹³C of 2′, 3-dihydroxy-2-methyldihydrochalcone 3′-*O*-*β*-D-(4′′-*O*-methyl)-glucopyranoside (**3b**)

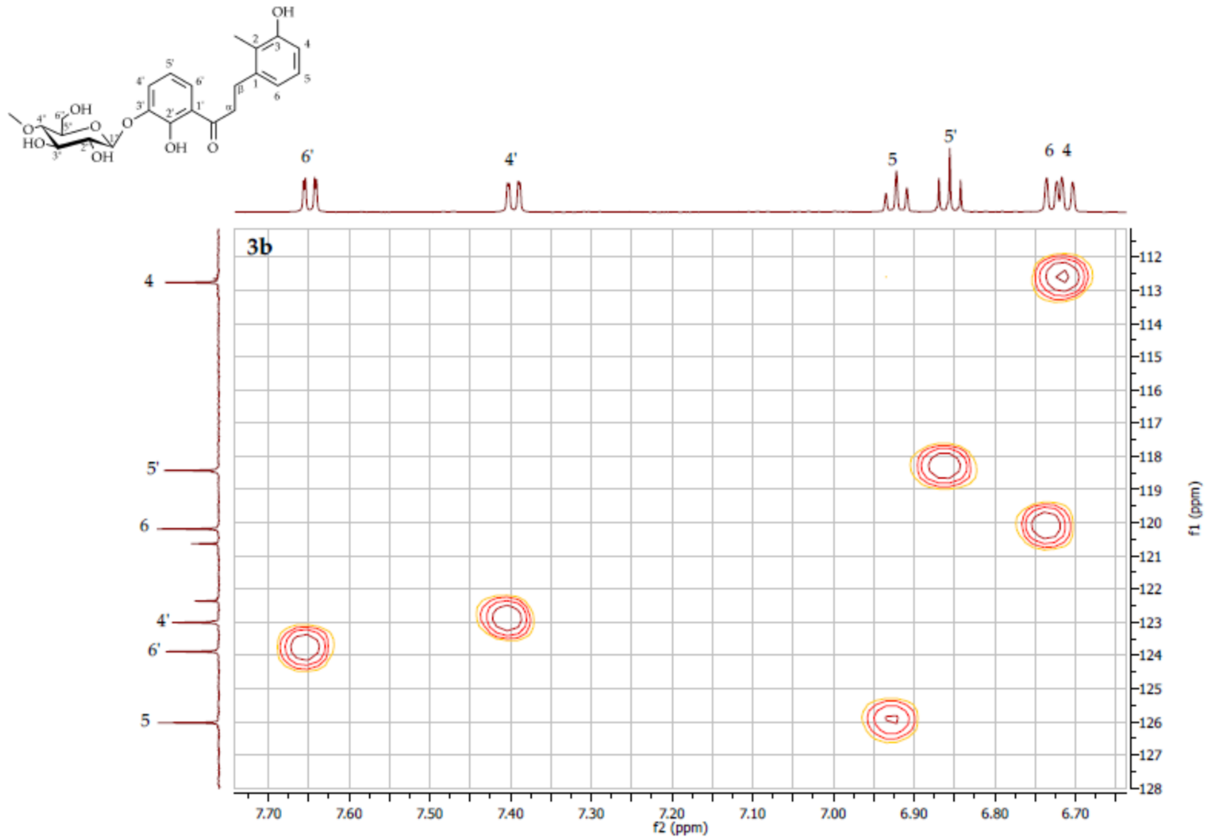

Figure S48. HSQC contour map– ¹H x ¹³C expansion of 2′, 3-dihydroxy-2-methyldihydrochalcone 3′-*O*-*β*-D-(4′′-*O*-methyl)-glucopyranoside (**3b**)

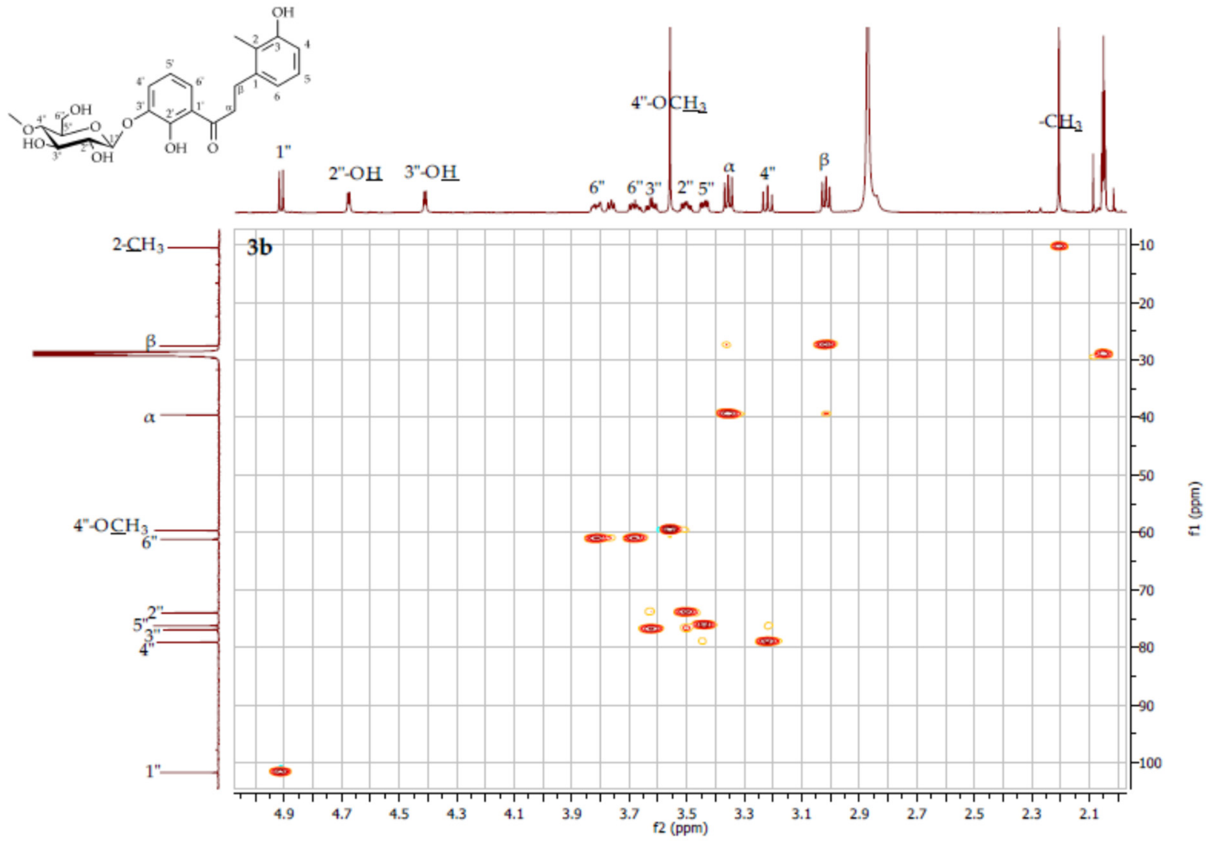

Figure S49. HSQC contour map– ^{1}H x ^{13}C expansion of 2′, 3-dihydroxy-2-methyldihydrochalcone 3′-*O*-*β*-D-(4′′-*O*-methyl)-glucopyranoside (**3b**)

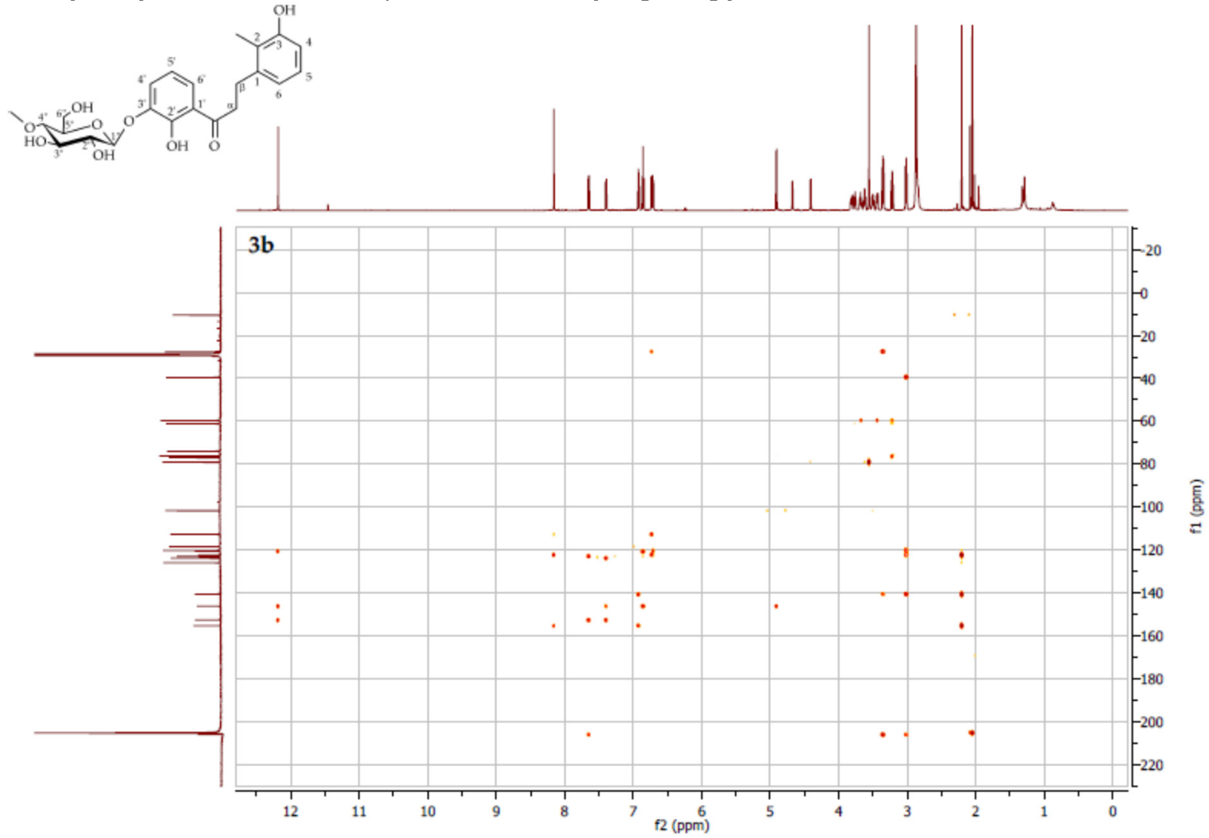

Figure S50. HMBC contour map– ^{1}H x ^{13}C of 2′, 3-dihydroxy-2-methyldihydrochalcone 3′-*O*-*β*-D-(4′′-*O*-methyl)-glucopyranoside (**3b**)

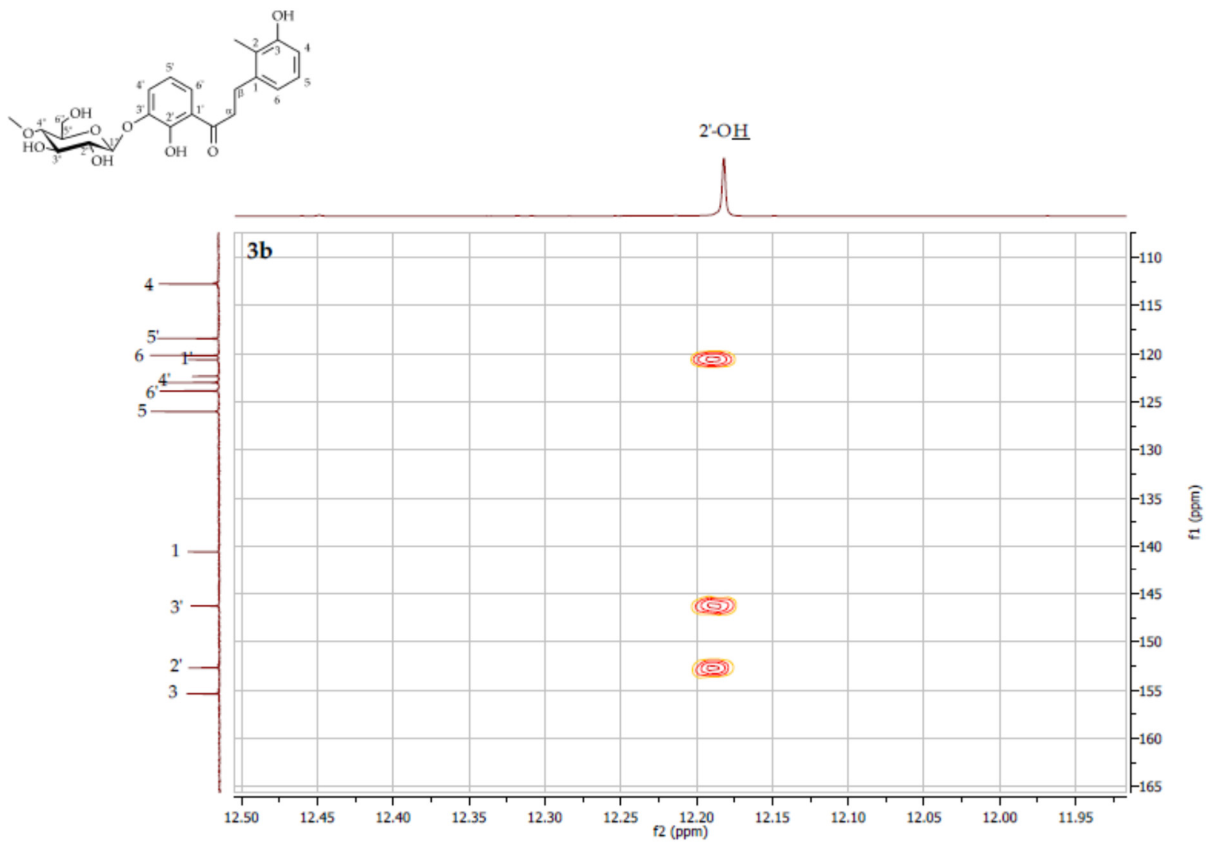

Figure S51. HMBC contour map– ^{1}H x ^{13}C expansion of 2′, 3-dihydroxy-2-methyldihydrochalcone 3′-*O*-*β*-D-(4′′-*O*-methyl)-glucopyranoside (**3b**)

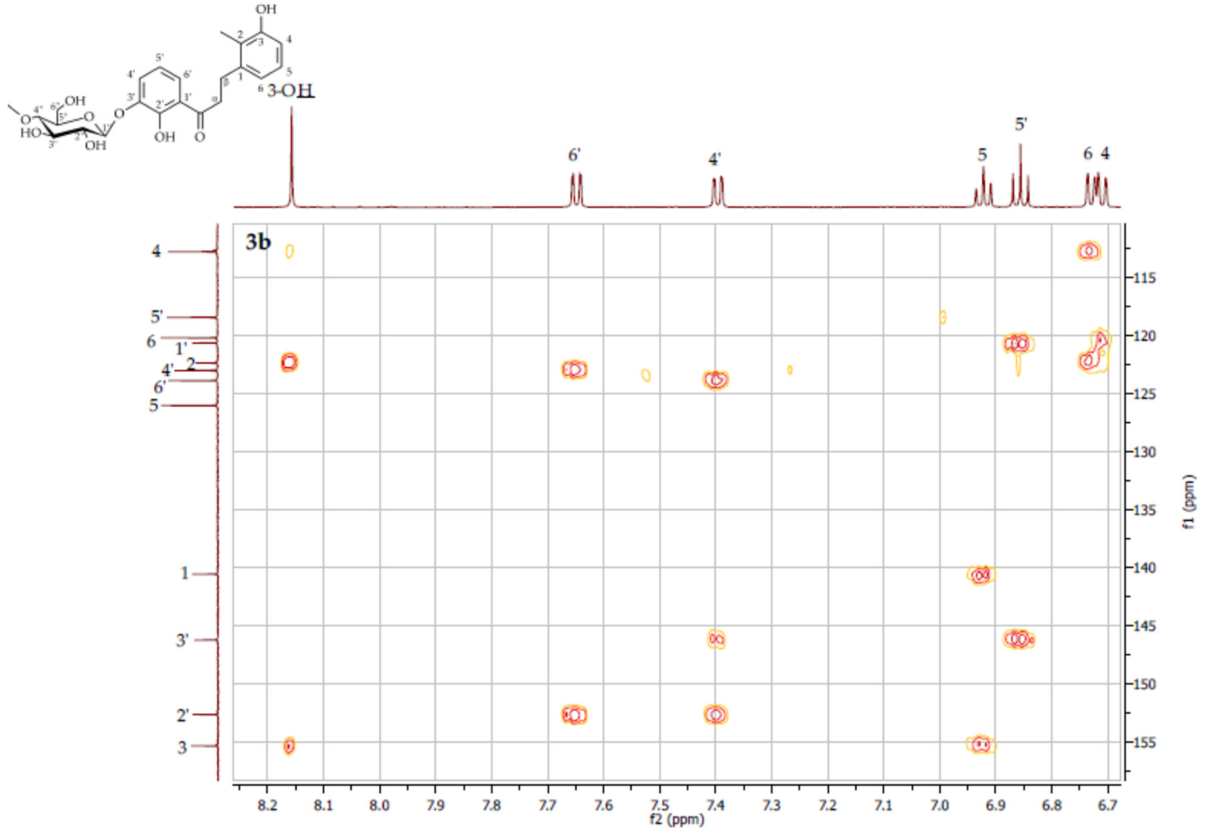

Figure S52. HMBC contour map– ^{1}H x ^{13}C expansion of 2′, 3-dihydroxy-2-methyldihydrochalcone 3′-*O*-*β*-D-(4′′-*O*-methyl)-glucopyranoside (**3b**)

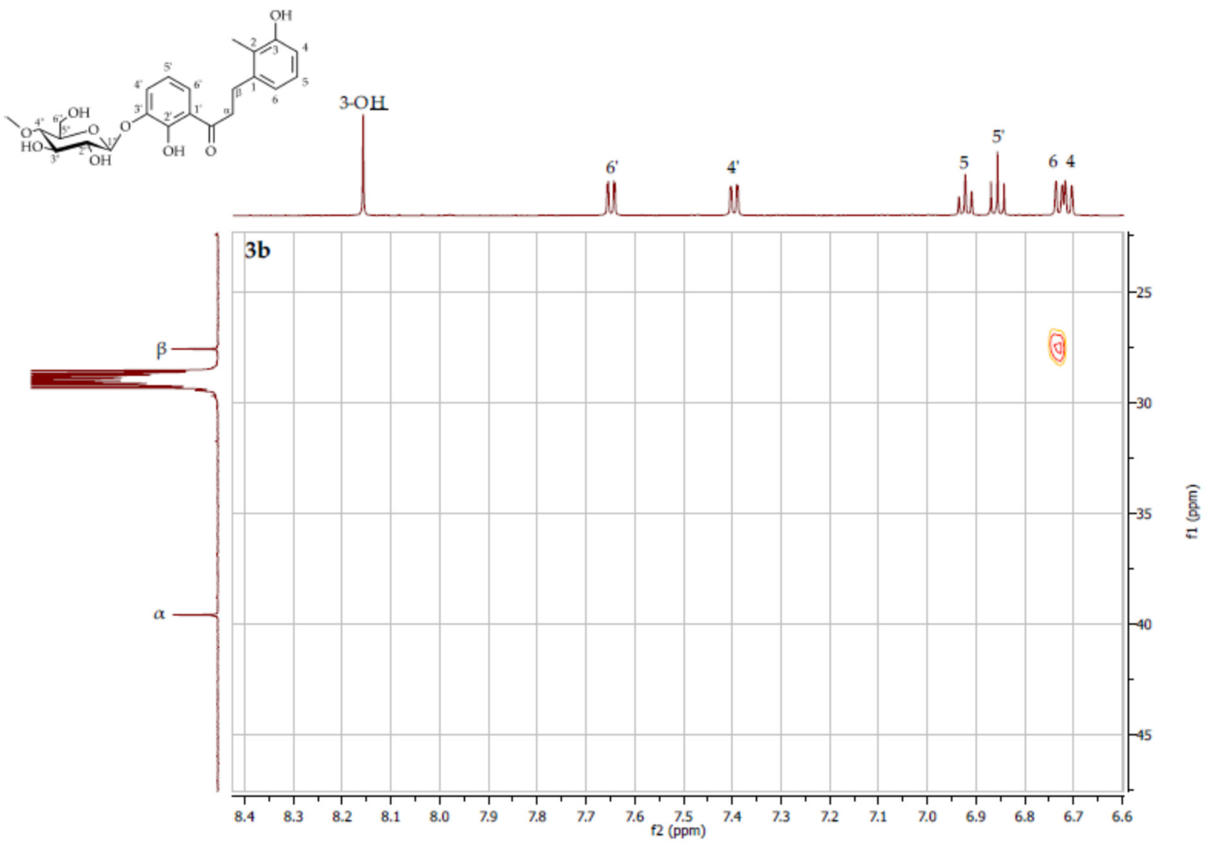

Figure S53. HMBC contour map– ^{1}H x ^{13}C expansion of 2′, 3-dihydroxy-2-methyldihydrochalcone 3′-*O*-*β*-D-(4′′-*O*-methyl)-glucopyranoside (**3b**)

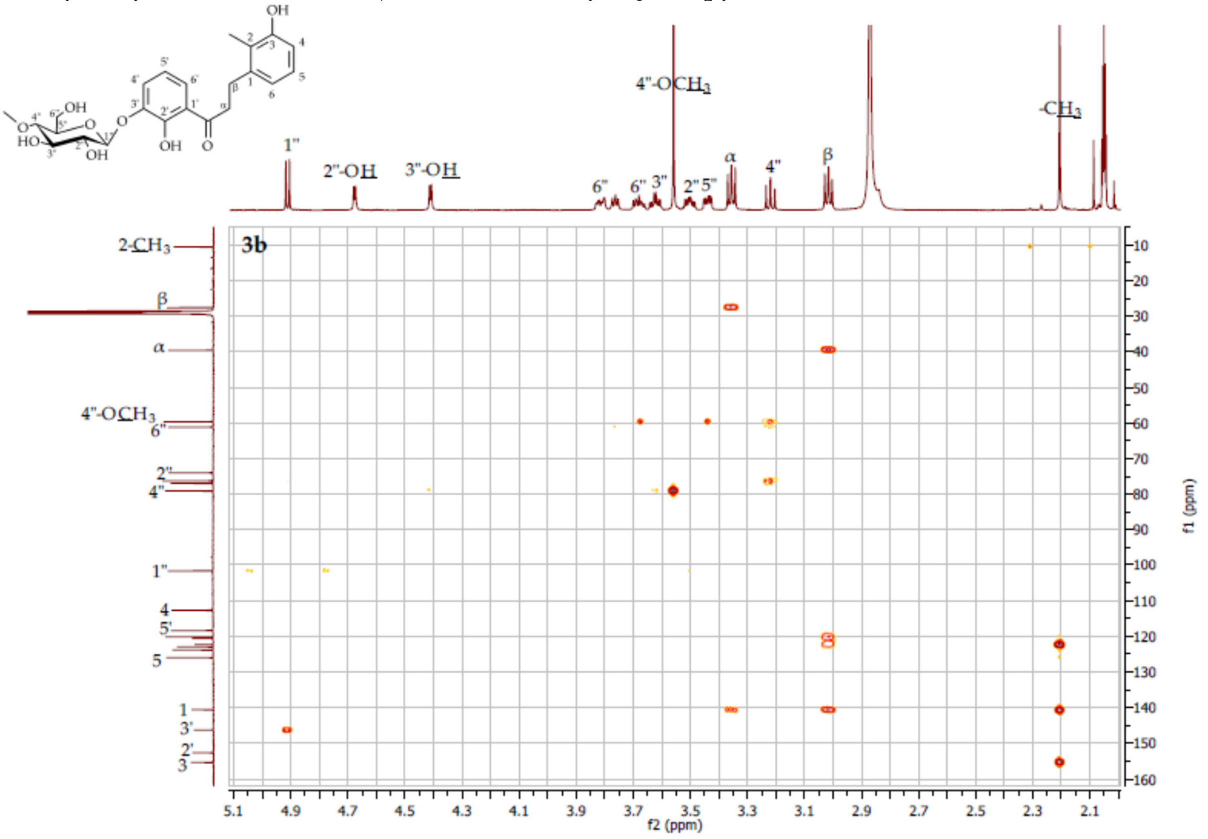

Figure S54. HMBC contour map– ^{1}H x ^{13}C expansion of 2′, 3-dihydroxy-2-methyldihydrochalcone 3′-*O*-*β*-D-(4′′-*O*-methyl)-glucopyranoside (**3b**)

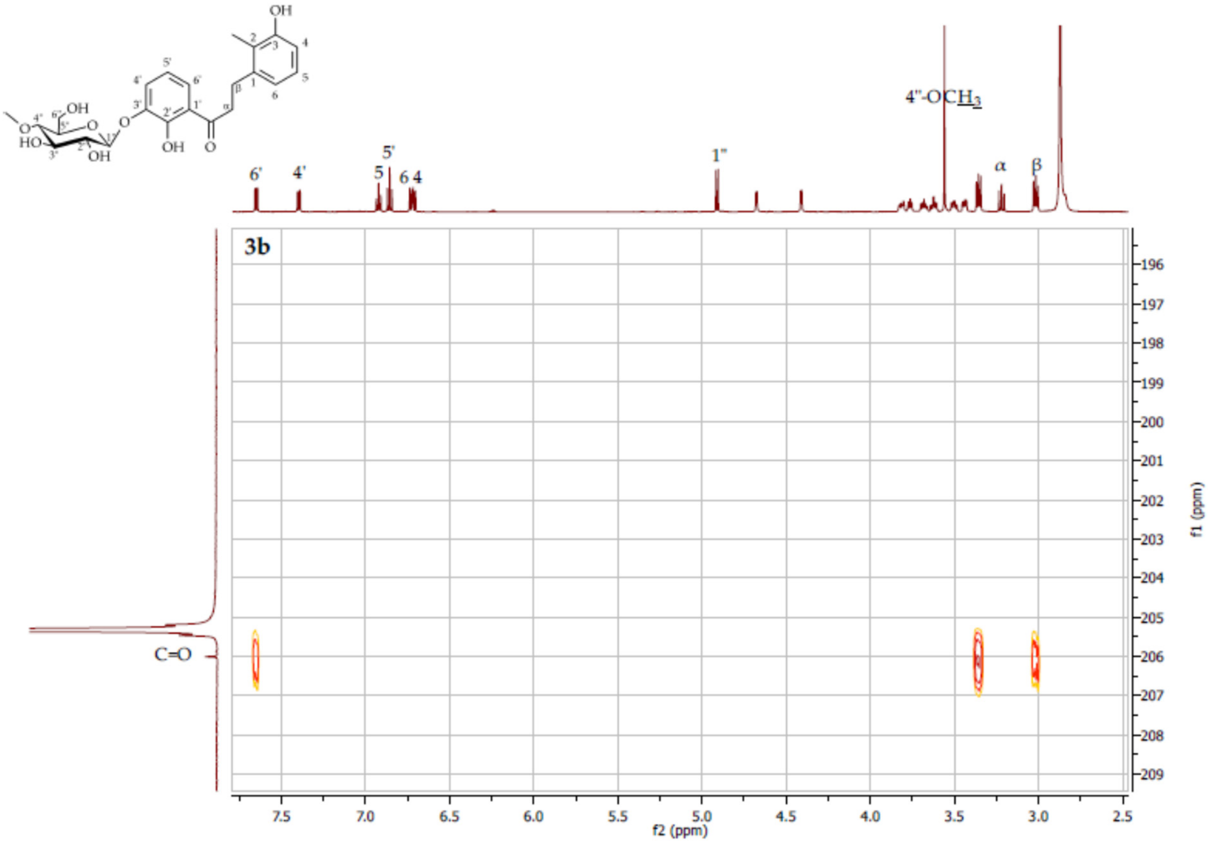

Figure S55. HMBC contour map– $^{1}H \times ^{13}C$ expansion of 2′, 3-dihydroxy-2-methyldihydrochalcone 3′-*O*-*β*-D-(4′′-*O*-methyl)-glucopyranoside (**3b**)

Molecular formula: $C_{23}H_{28}O_9$
Formula weight: 448.17
Ionization mode: negative
Precursor: $[M-H]^-$ 447.30
447.3000>271.1000 CE: 27.0
447.3000>108.0500 CE: 50.0
447.3000>235.0500 CE: 43.0

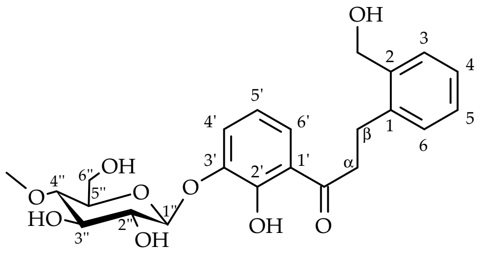

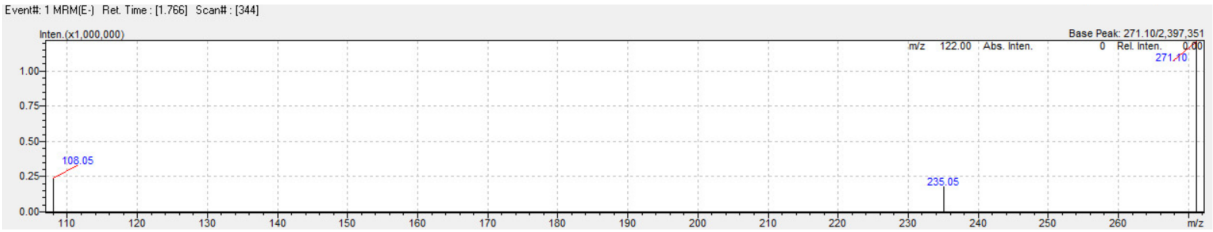

Figure S56. MS analysis of 2′-hydroxy-2-hydroxymethyldihydrochalcone 3′-*O*-*β*-D-(4′′-*O*-methyl)-glucopyranoside (**3c**)

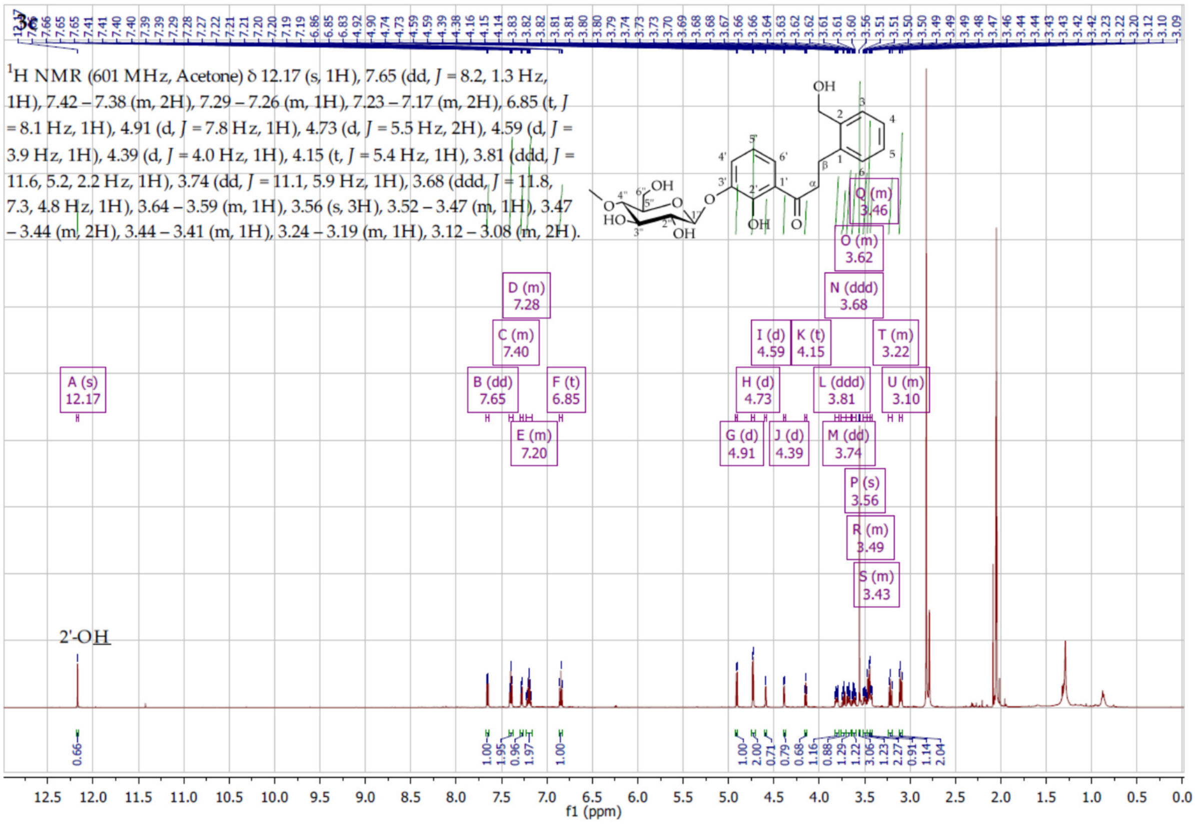

Figure S57. ¹H NMR spectrum (*δ*, acetone-d6, 600 MHz) of 2′-hydroxy-2-hydroxymethyldihydrochalcone 3′-*O*-*β*-D-(4′′-*O*-methyl)-glucopyranoside (**3c**)

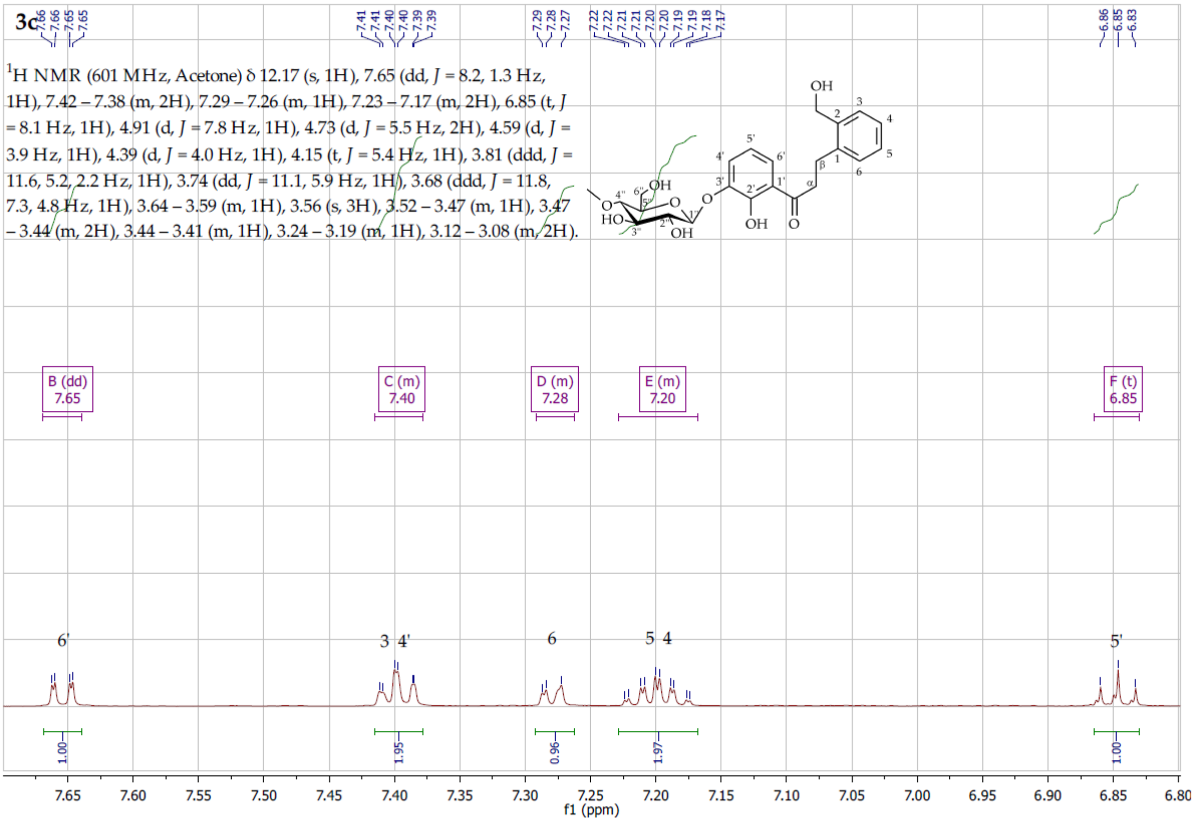

Figure S58. ¹H NMR spectrum expansion (*δ*, acetone-d6, 600 MHz) of 2′-hydroxy-2-hydroxymethyldihydrochalcone 3′-*O*-*β*-D-(4′′-*O*-methyl)-glucopyranoside (**3c**)

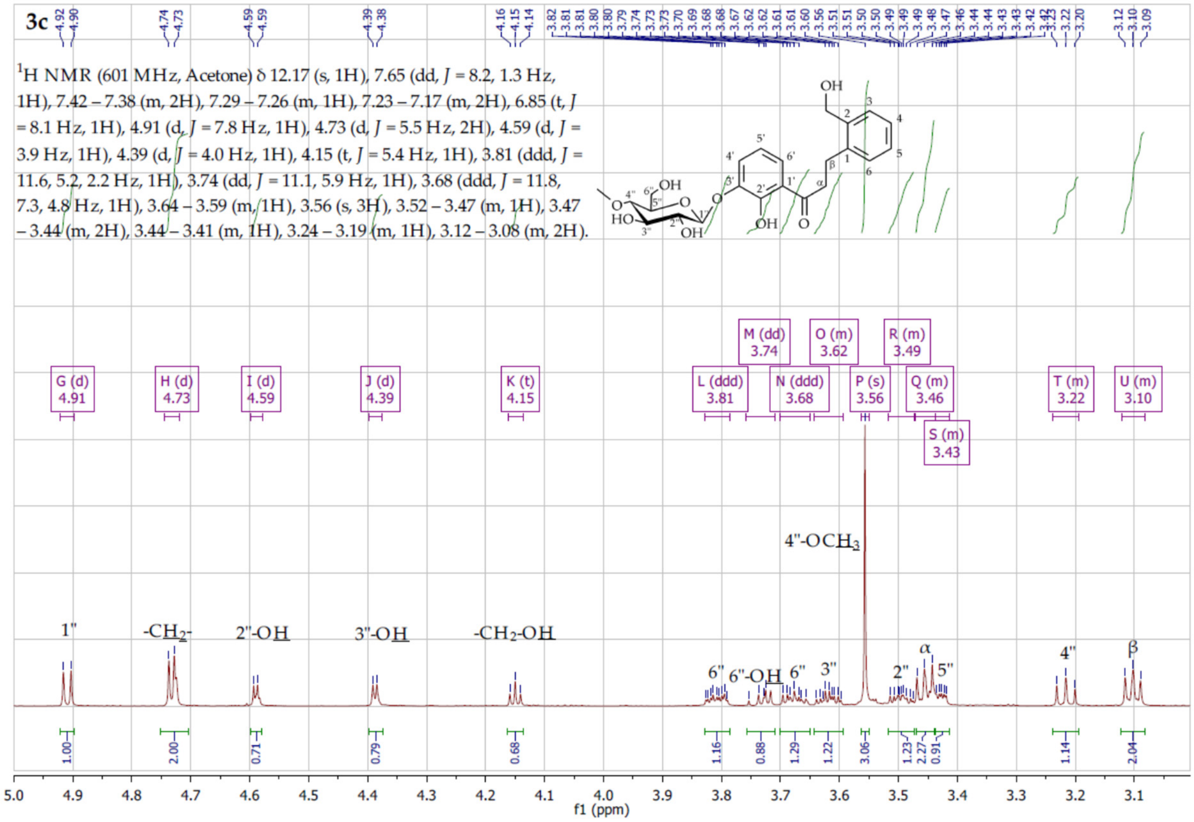

Figure S59. ^{1}H NMR spectrum expansion (*δ,* acetone-d6, 600 MHz) of 2′-hydroxy-2-hydroxymethyldihydrochalcone 3′-*O*-*β*-D-(4′′-*O*-methyl)-glucopyranoside (**3c**)

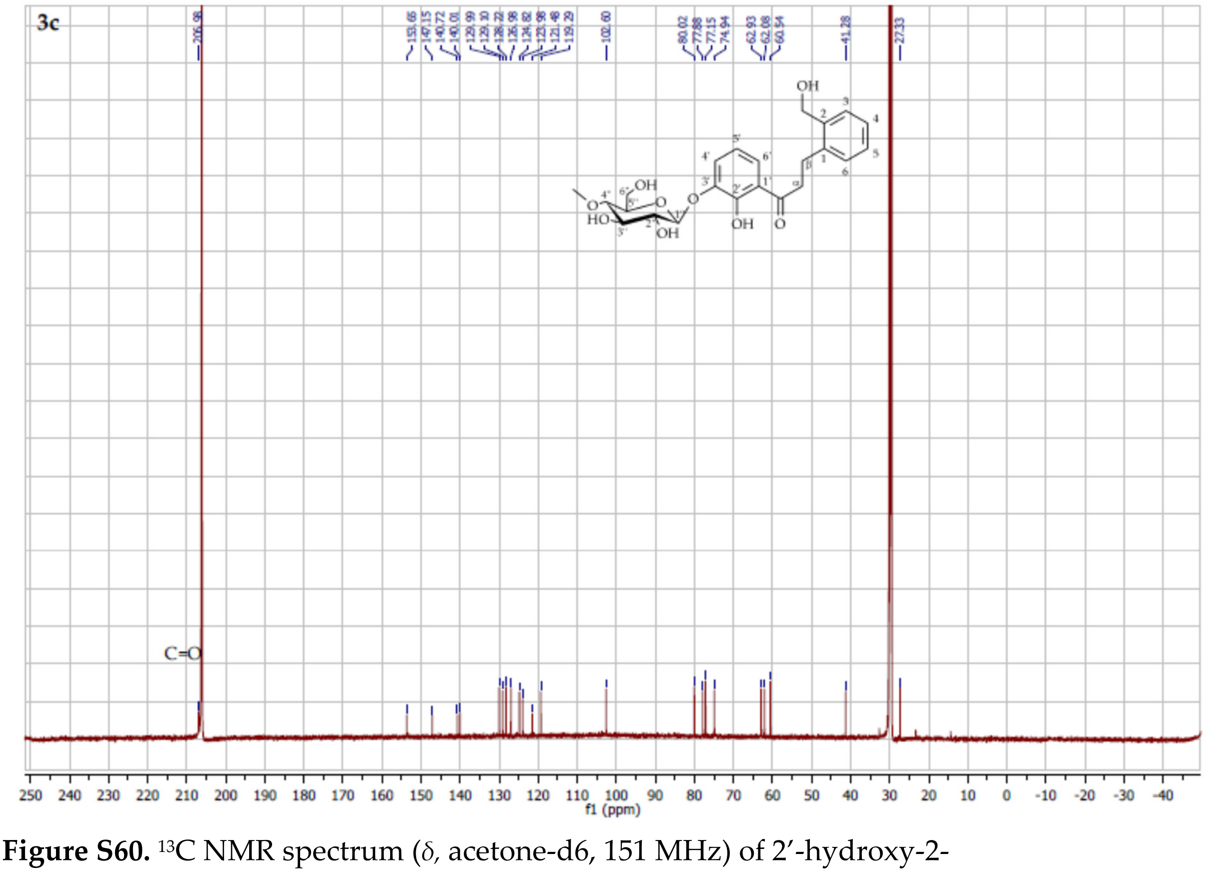

Figure S60. ^{13}C NMR spectrum (*δ,* acetone-d6, 151 MHz) of 2′-hydroxy-2-hydroxymethyldihydrochalcone 3′-*O*-*β*-D-(4′′-*O*-methyl)-glucopyranoside (**3c**)

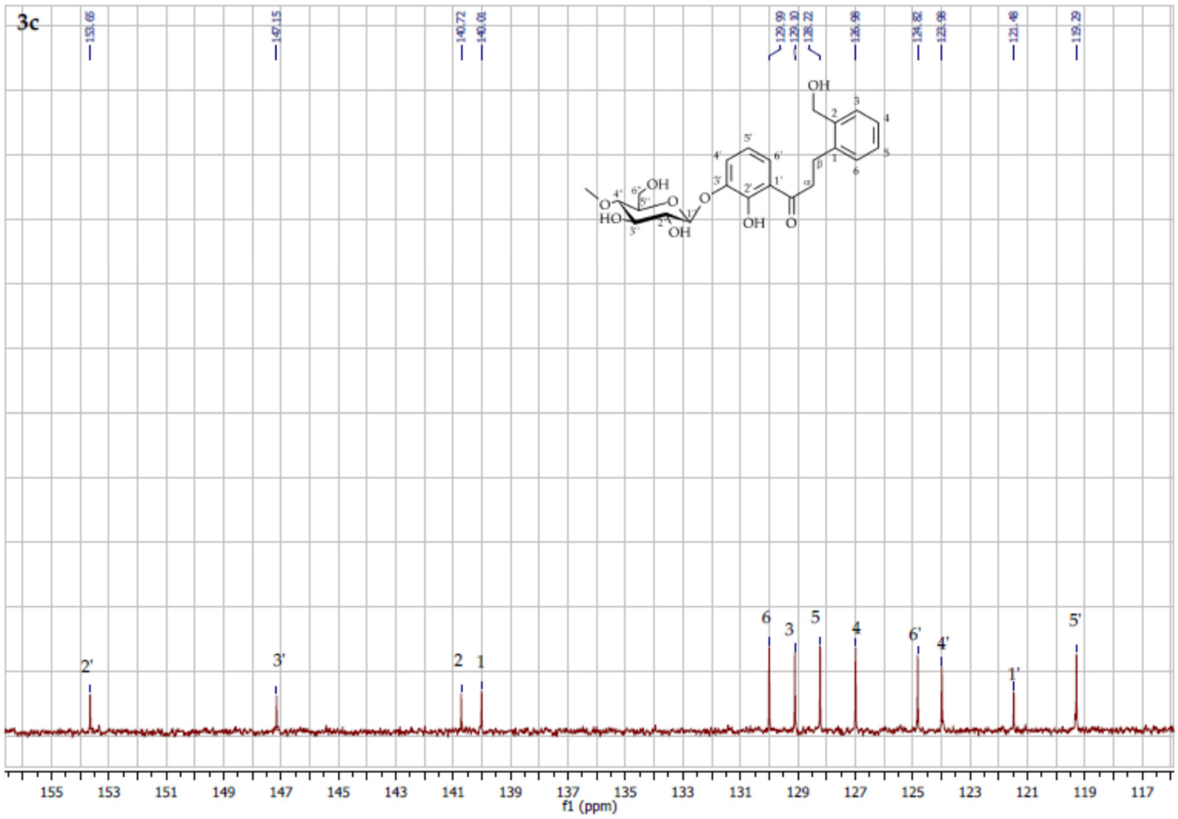

Figure S61. ^{13}C NMR spectrum expansion (*δ*, acetone-d6, 151 MHz) of 2′-hydroxy-2-hydroxymethyldihydrochalcone 3′-*O*-*β*-D-(4′′-*O*-methyl)-glucopyranoside (**3c**)

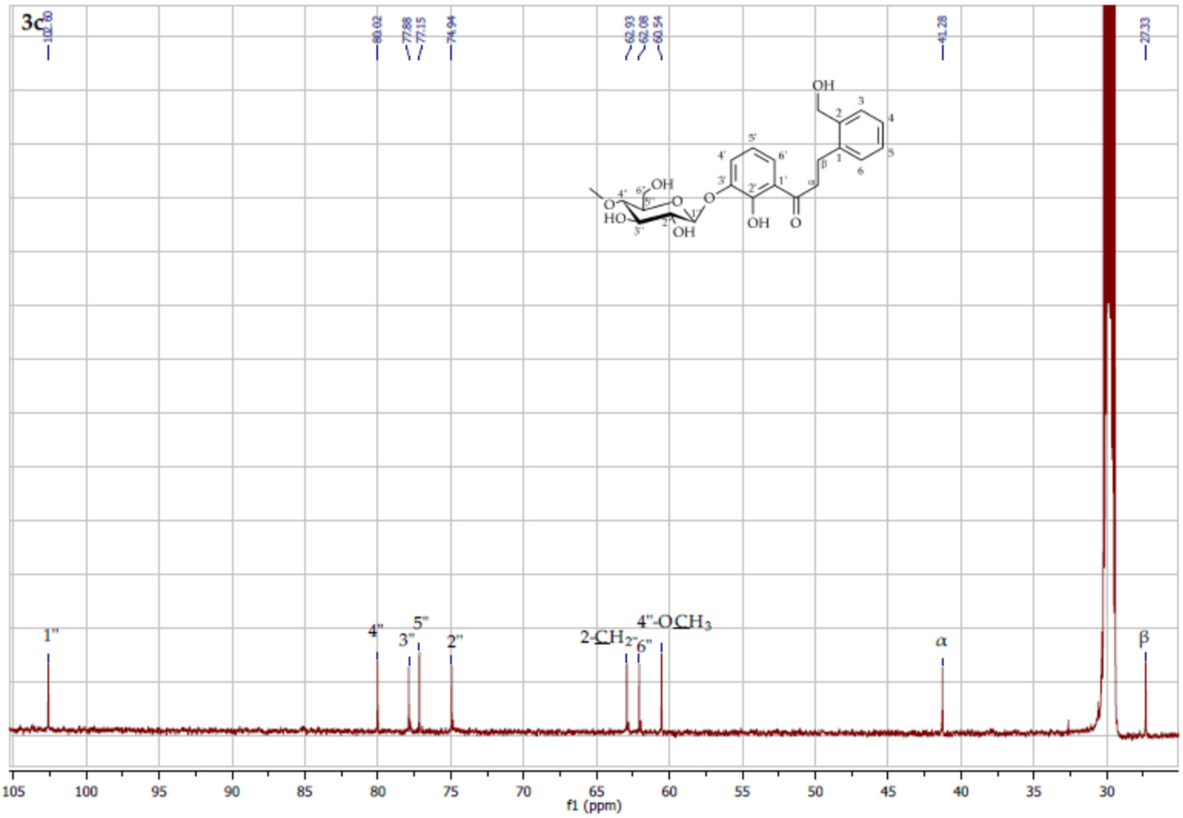

Figure S62. ^{13}C NMR spectrum expansion (*δ*, acetone-d6, 151 MHz) of 2′-hydroxy-2-hydroxymethyldihydrochalcone 3′-*O*-*β*-D-(4′′-*O*-methyl)-glucopyranoside (**3c**)

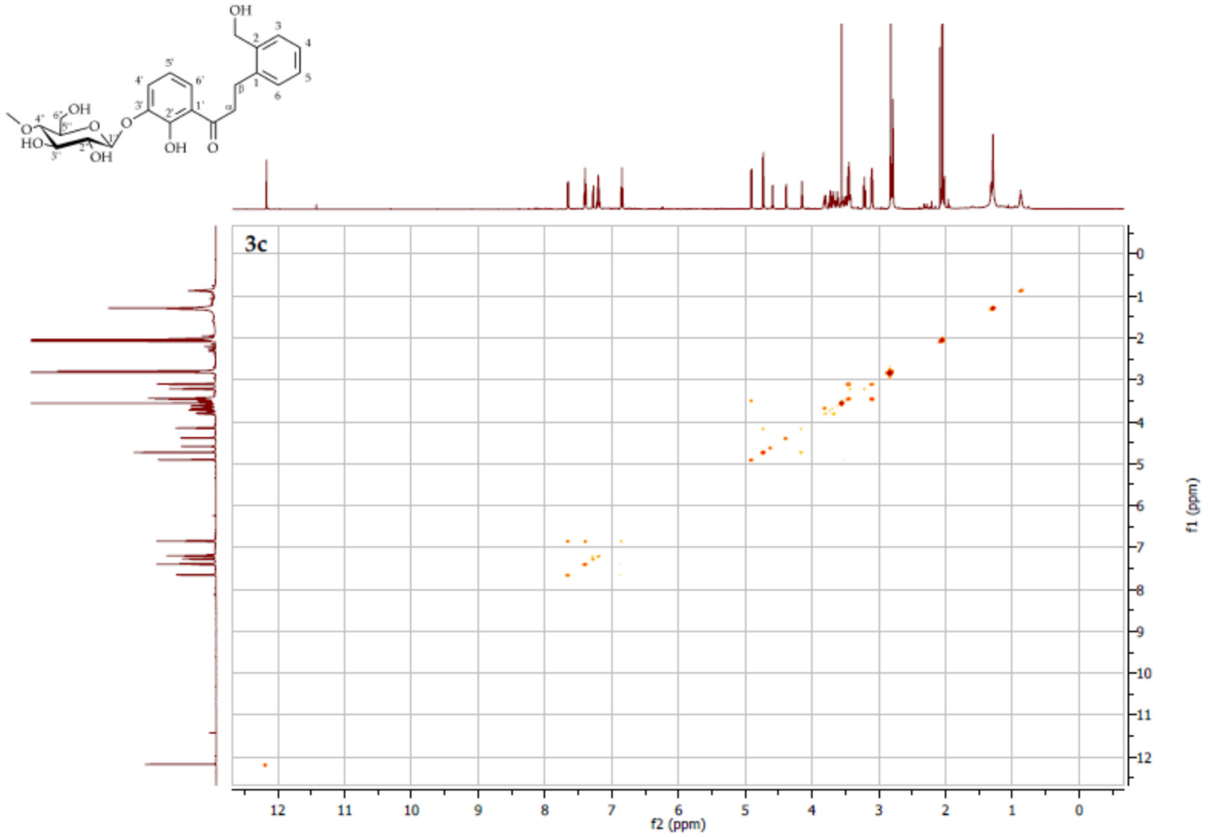

Figure S63. COSY contour map – ^1H x $^1H$of 2′-hydroxy-2-hydroxymethyldihydrochalcone 3′-*O*-*β*-D-(4′′-*O*-methyl)-glucopyranoside (**3c**)

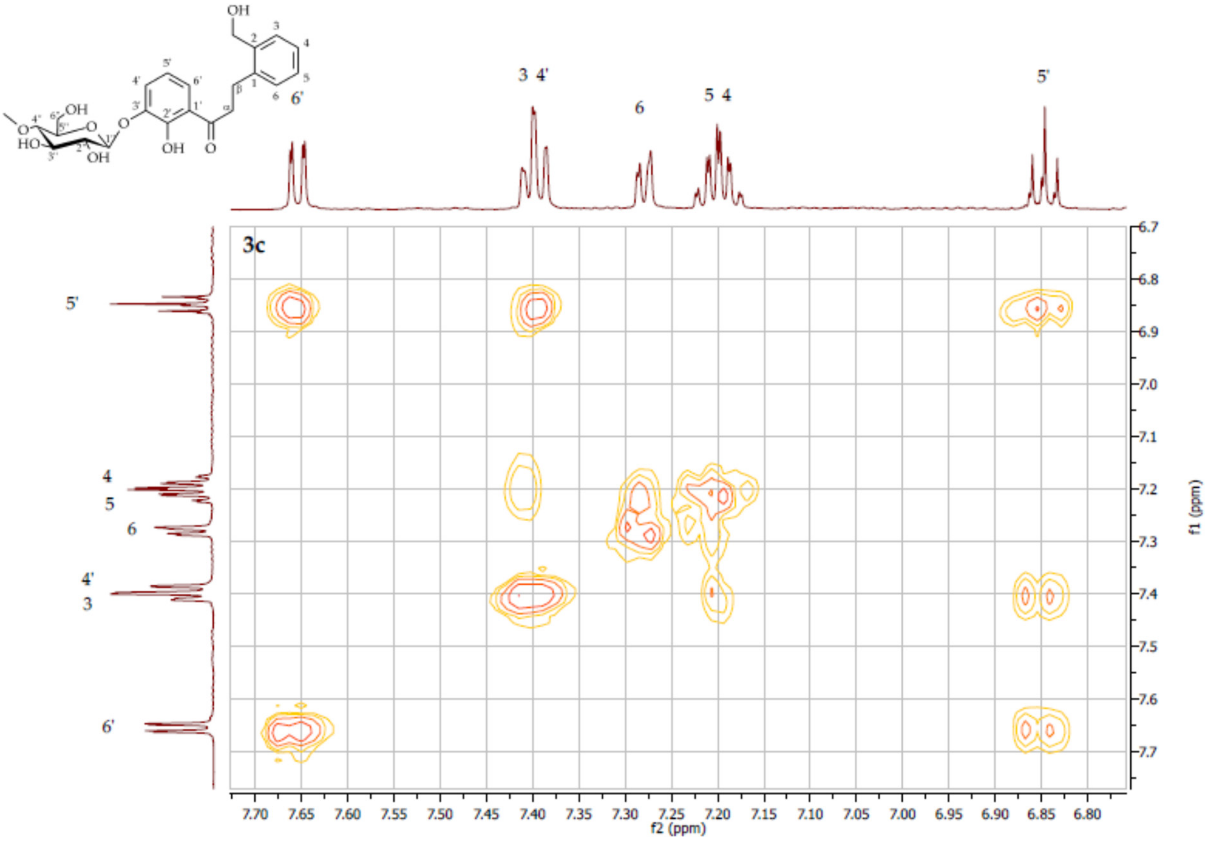

Figure S64. COSY contour map – ^1H x ^1Hexpansion of 2′-hydroxy-2-hydroxymethyldihydrochalcone 3′-*O*-*β*-D-(4′′-*O*-methyl)-glucopyranoside (**3c**)

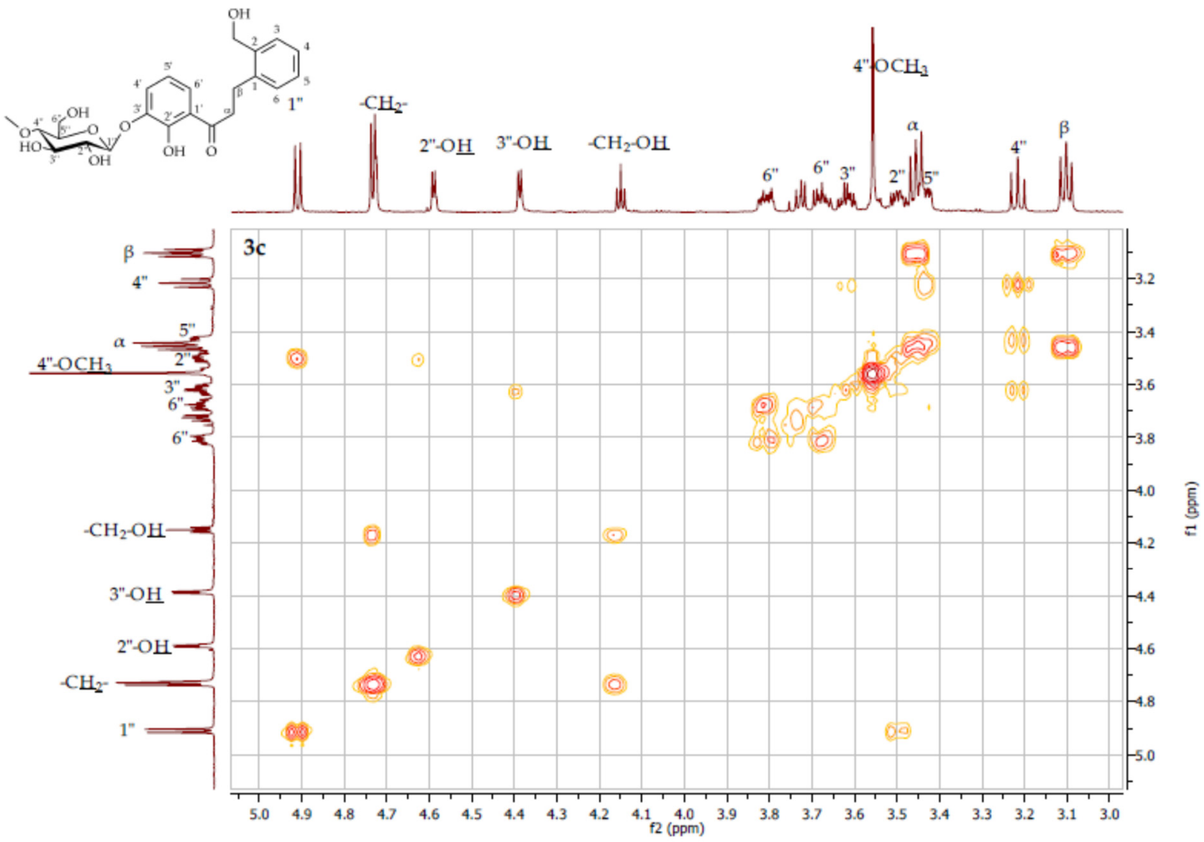

Figure S65. COSY contour map – $^{1}H \times {}^{1}H$expansion of 2′-hydroxy-2-hydroxymethyldihydrochalcone 3′-*O*-*β*-D-(4′′-*O*-methyl)-glucopyranoside (**3c**)

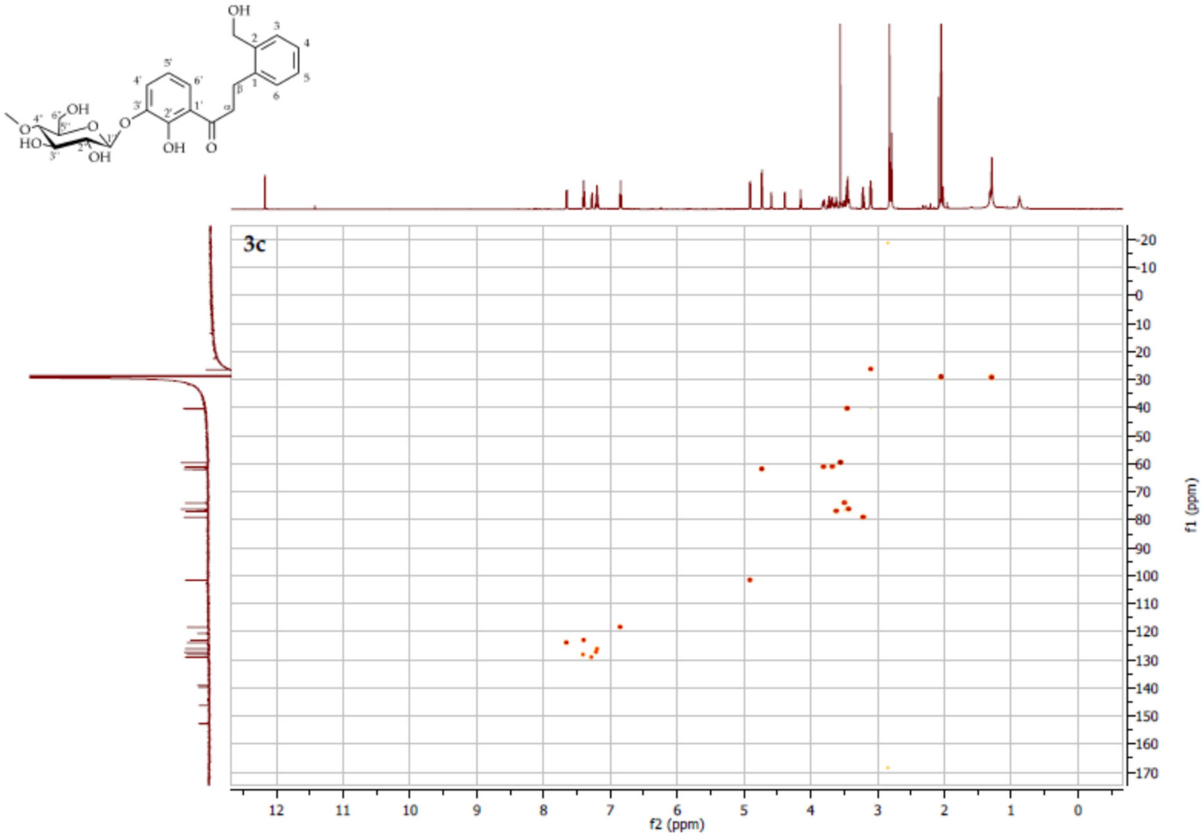

Figure S66. HSQC contour map– $^{1}H \times {}^{13}C$ of 2′-hydroxy-2-hydroxymethyldihydrochalcone 3′-*O*-*β*-D-(4′′-*O*-methyl)-glucopyranoside (**3c**)

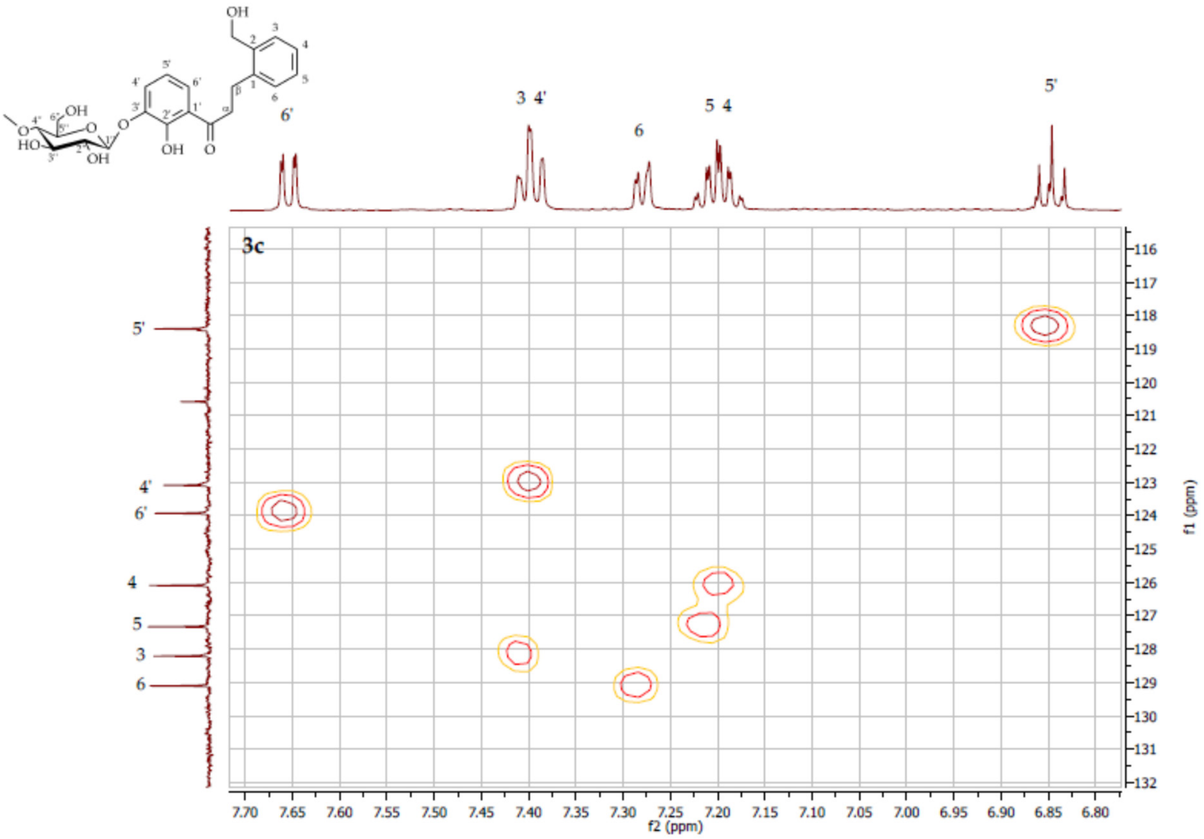

Figure S67. HSQC contour map– ^{1}H x ^{13}C expansion of 2′-hydroxy-2-hydroxymethyldihydrochalcone 3′-*O*-*β*-D-(4′′-*O*-methyl)-glucopyranoside (**3c**)

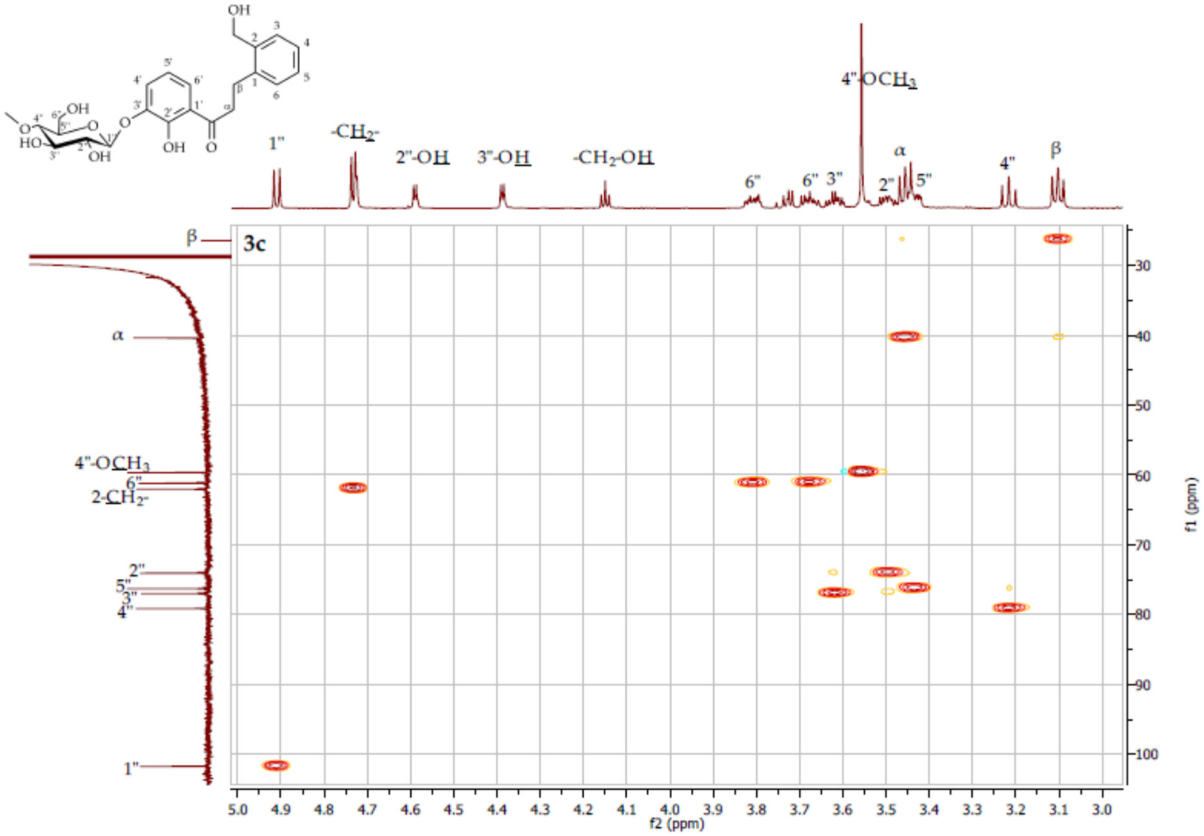

Figure S68. HSQC contour map– ^{1}H x ^{13}C expansion of 2′-hydroxy-2-hydroxymethyldihydrochalcone 3′-*O*-*β*-D-(4′′-*O*-methyl)-glucopyranoside (**3c**)

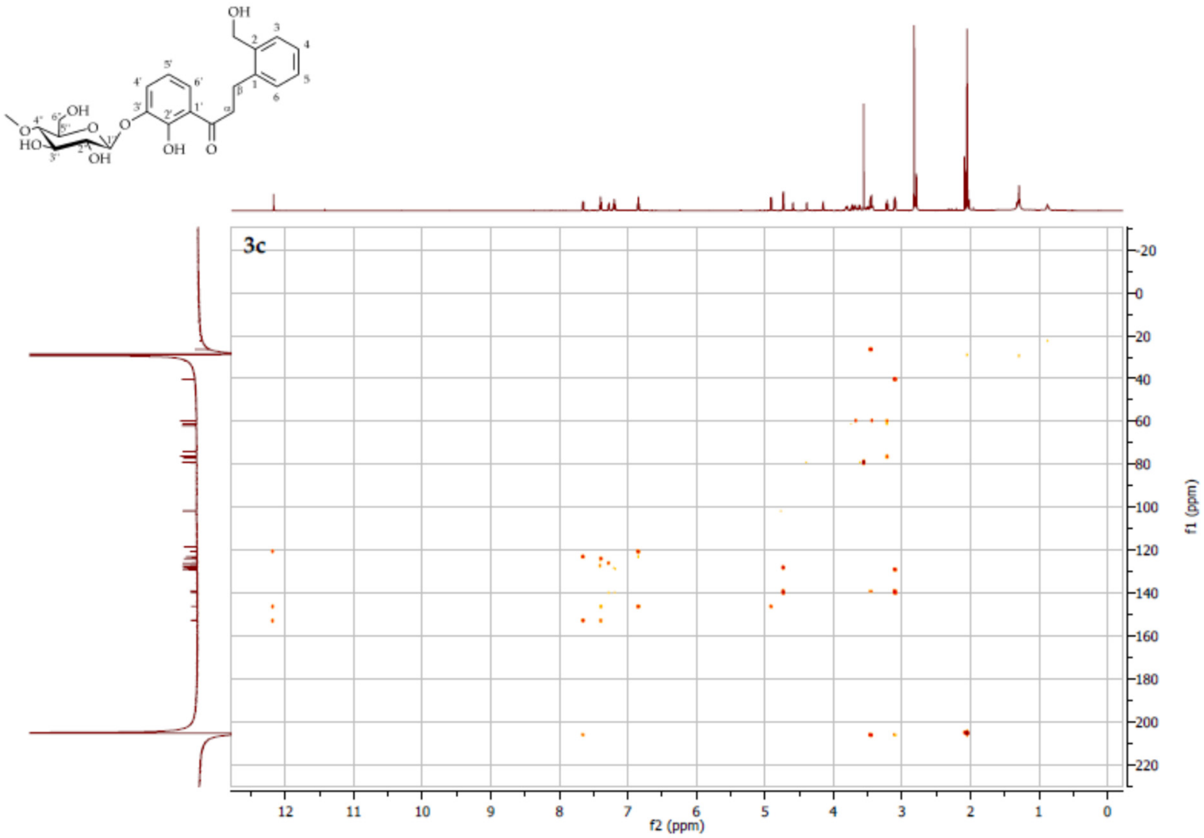

Figure S69. HMBC contour map– ^{1}H x ^{13}C of 2′-hydroxy-2-hydroxymethyldihydrochalcone 3′-*O*-*β*-D-(4′′-*O*-methyl)-glucopyranoside (**3c**)

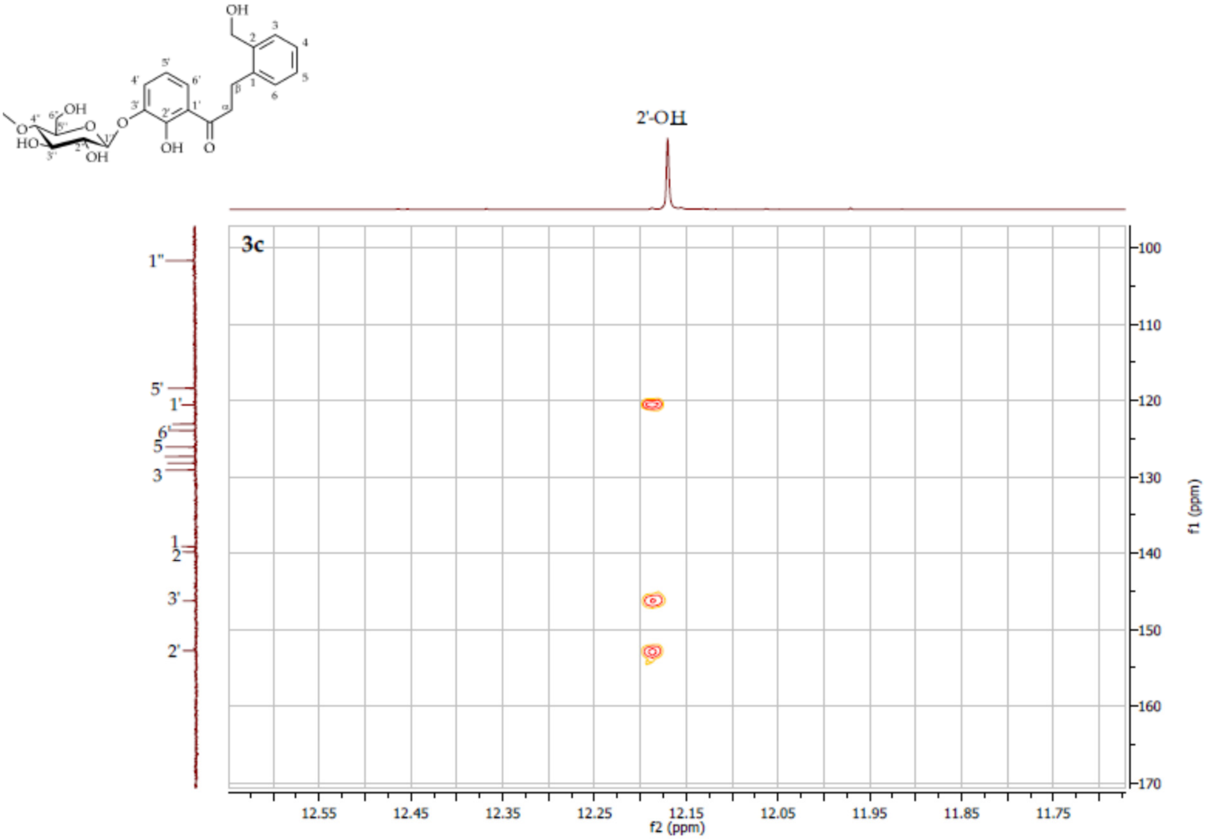

Figure S70. HMBC contour map– ^{1}H x ^{13}C expansion of 2′-hydroxy-2-hydroxymethyldihydrochalcone 3′-*O*-*β*-D-(4′′-*O*-methyl)-glucopyranoside (**3c**)

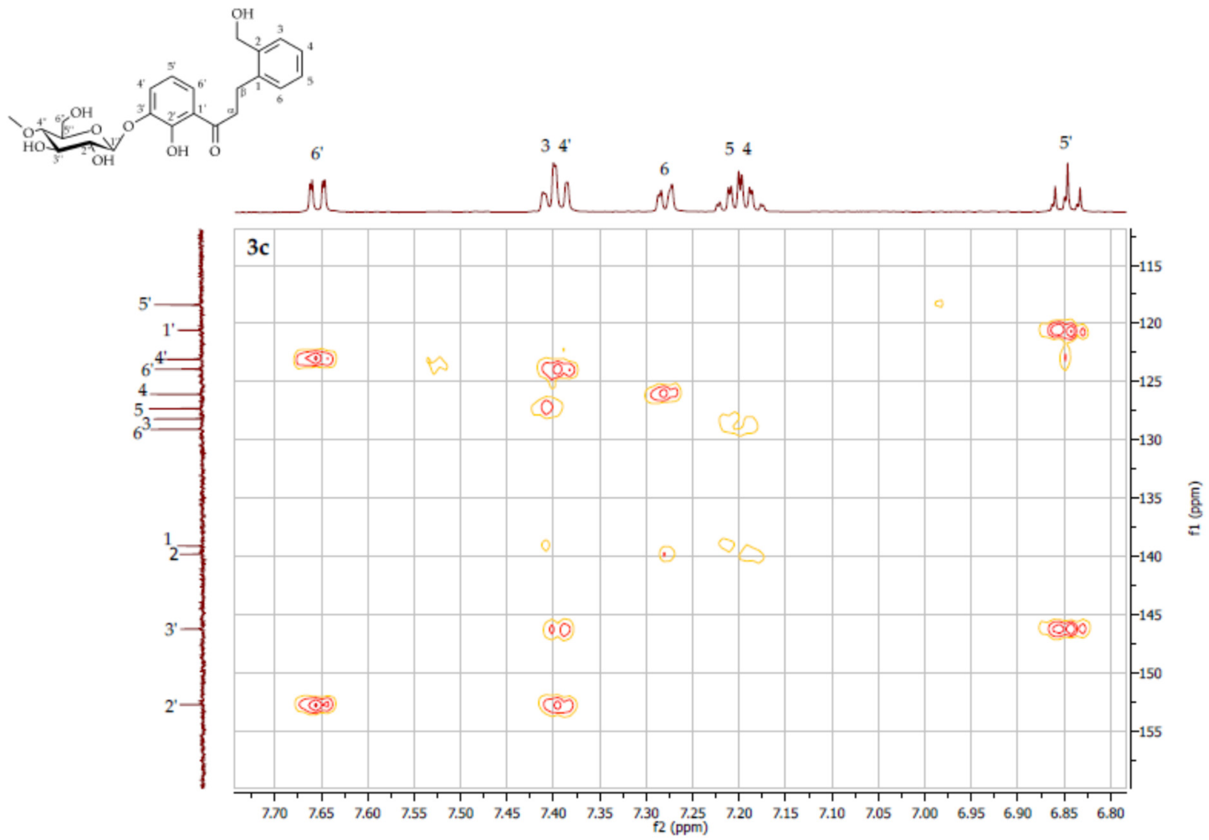

Figure S71. HMBC contour map– ^{1}H x ^{13}C expansion of 2′-hydroxy-2-hydroxymethyldihydrochalcone 3′-*O*-*β*-D-(4′′-*O*-methyl)-glucopyranoside (**3c**)

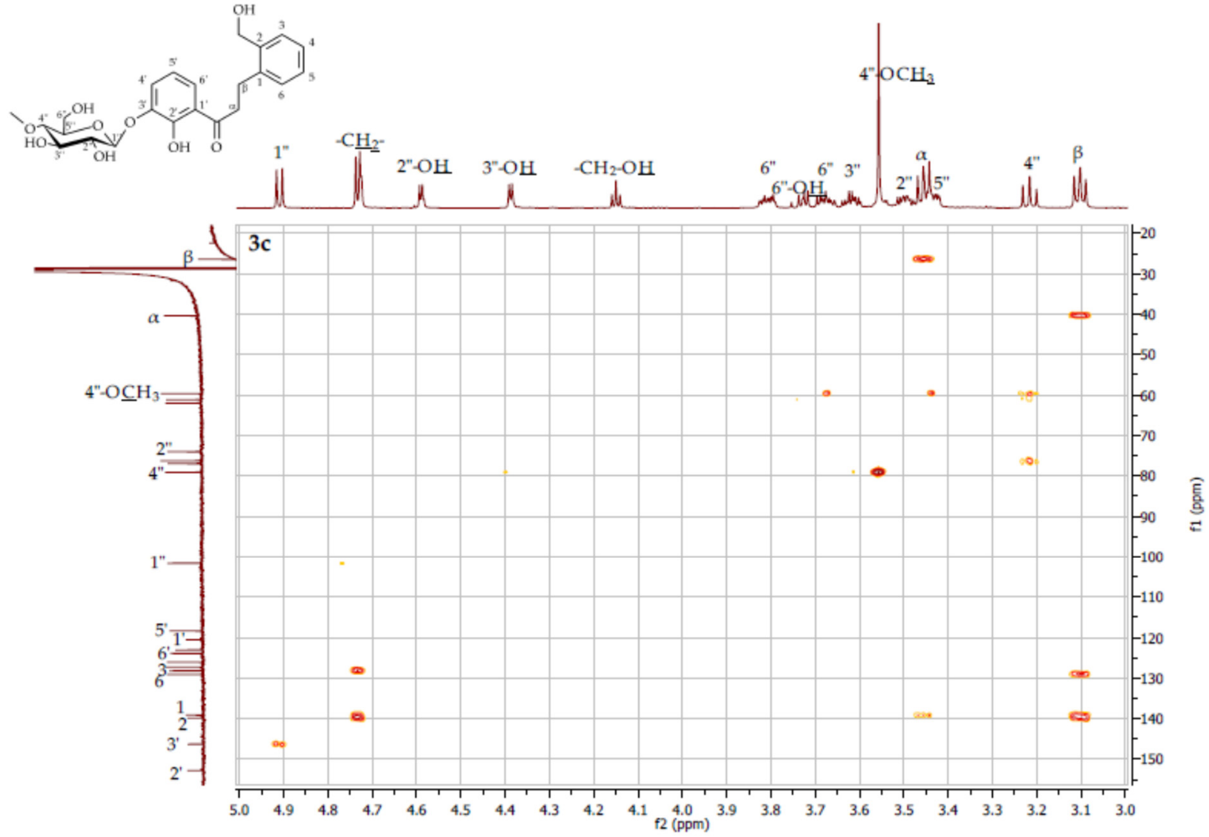

Figure S72. HMBC contour map– ^{1}H x ^{13}C expansion of 2′-hydroxy-2-hydroxymethyldihydrochalcone 3′-*O*-*β*-D-(4′′-*O*-methyl)-glucopyranoside (**3c**)

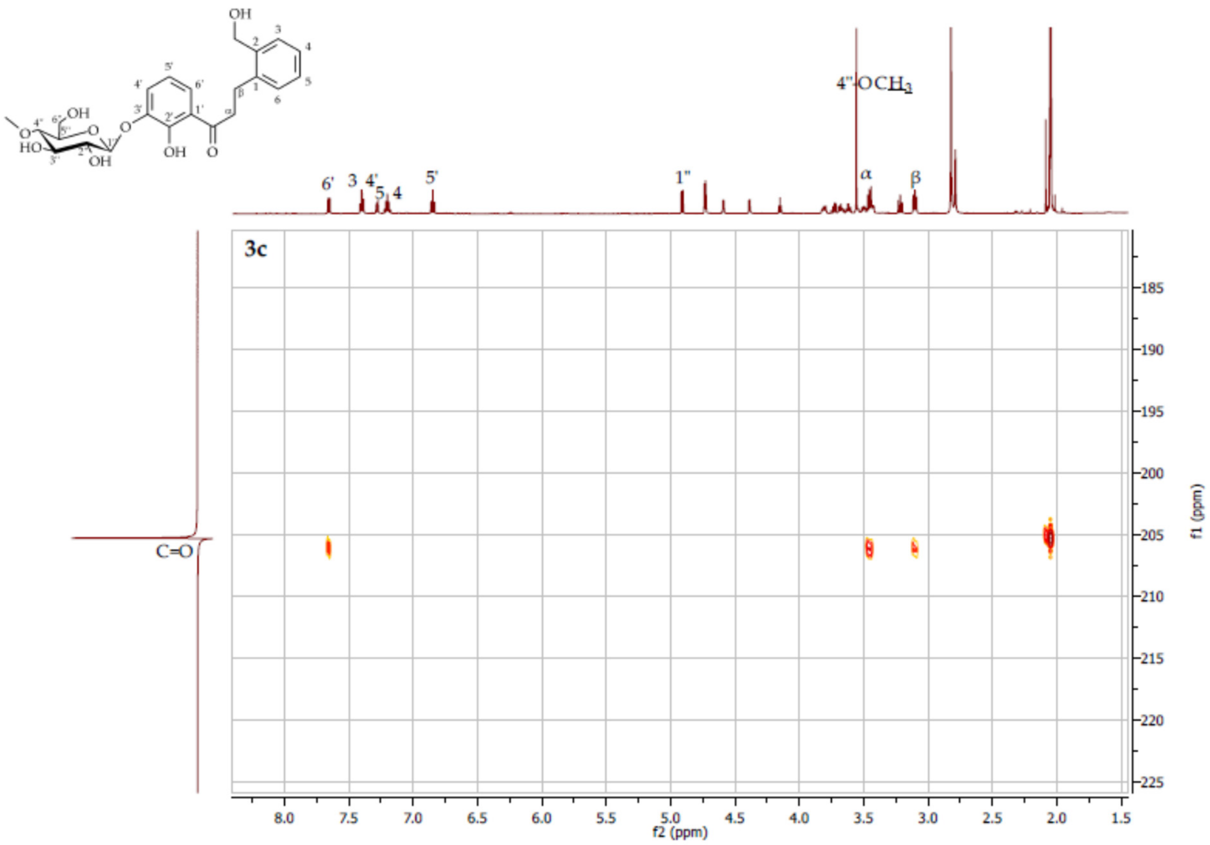

Figure S73. HMBC contour map– ^{1}H x ^{13}C expansion of 2′-hydroxy-2-hydroxymethyldihydrochalcone 3′-*O*-*β*-D-(4′′-*O*-methyl)-glucopyranoside (**3c**)

Molecular formula:$C_{23}H_{28}O_9$
Formula weight:448.17
Ionization mode:negative
Precursor: $[M - H]^-$ 447.30
447.3000>151.1000 CE: 32.0
447.3000>327.1000 CE: 20.0
447.3000>271.0000 CE: 19.0

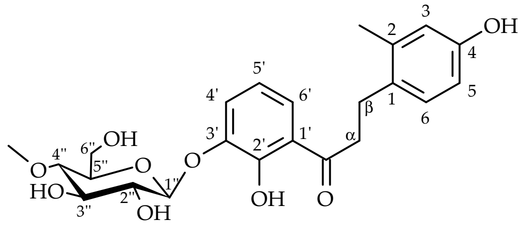

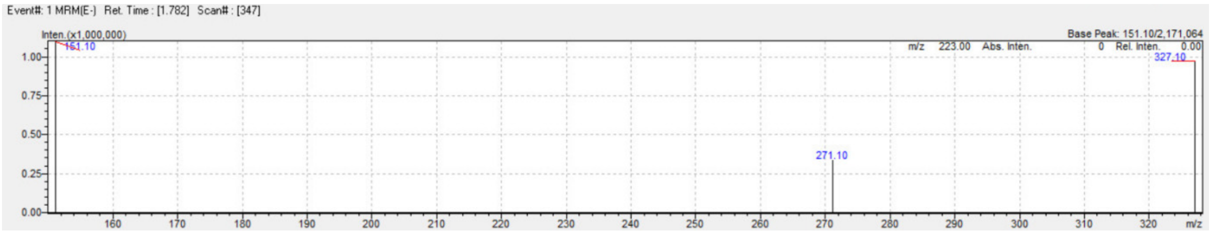

Figure S74.MS analysis of 2′,4-dihydroxy-2-methyldihydrochalcone 3′-*O*-*β*-D-(4′′-*O*-methyl)-glucopyranoside (**3d**)

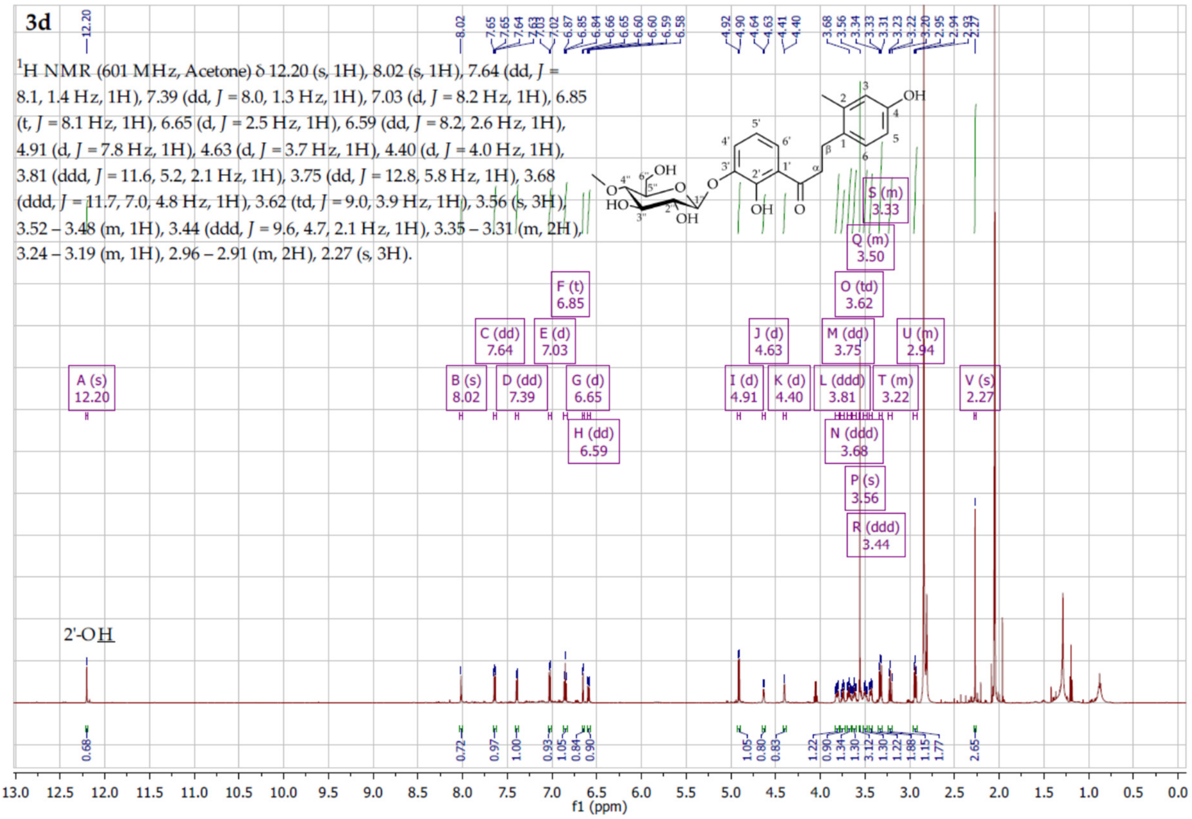

Figure S75. ^{1}H NMR spectrum (*δ,* acetone-d6, 600 MHz) of 2′,4-dihydroxy-2-methyldihydrochalcone 3′-*O*-*β*-D-(4′′-*O*-methyl)-glucopyranoside (**3d**)

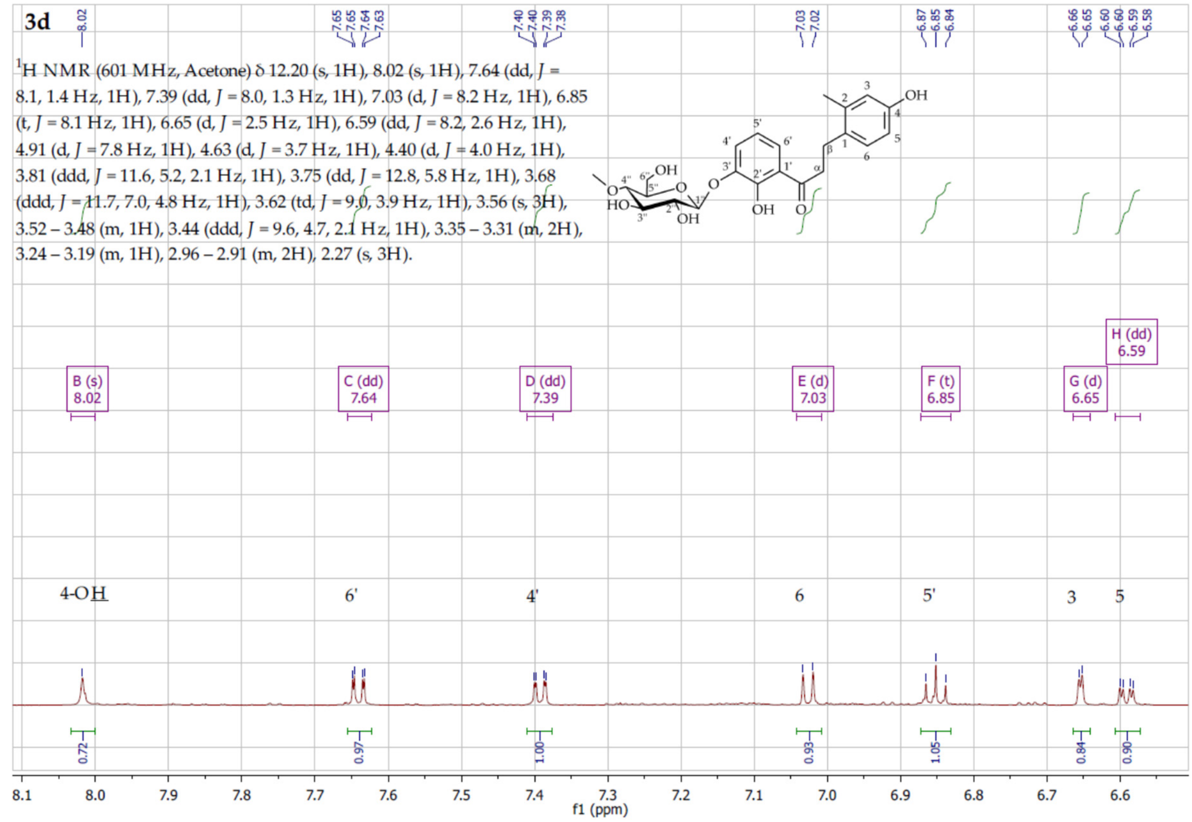

Figure S76. ^{1}H NMR spectrum expansion (*δ,* acetone-d6, 600 MHz) of 2′,4-dihydroxy-2-methyldihydrochalcone 3′-*O*-*β*-D-(4′′-*O*-methyl)-glucopyranoside (**3d**)

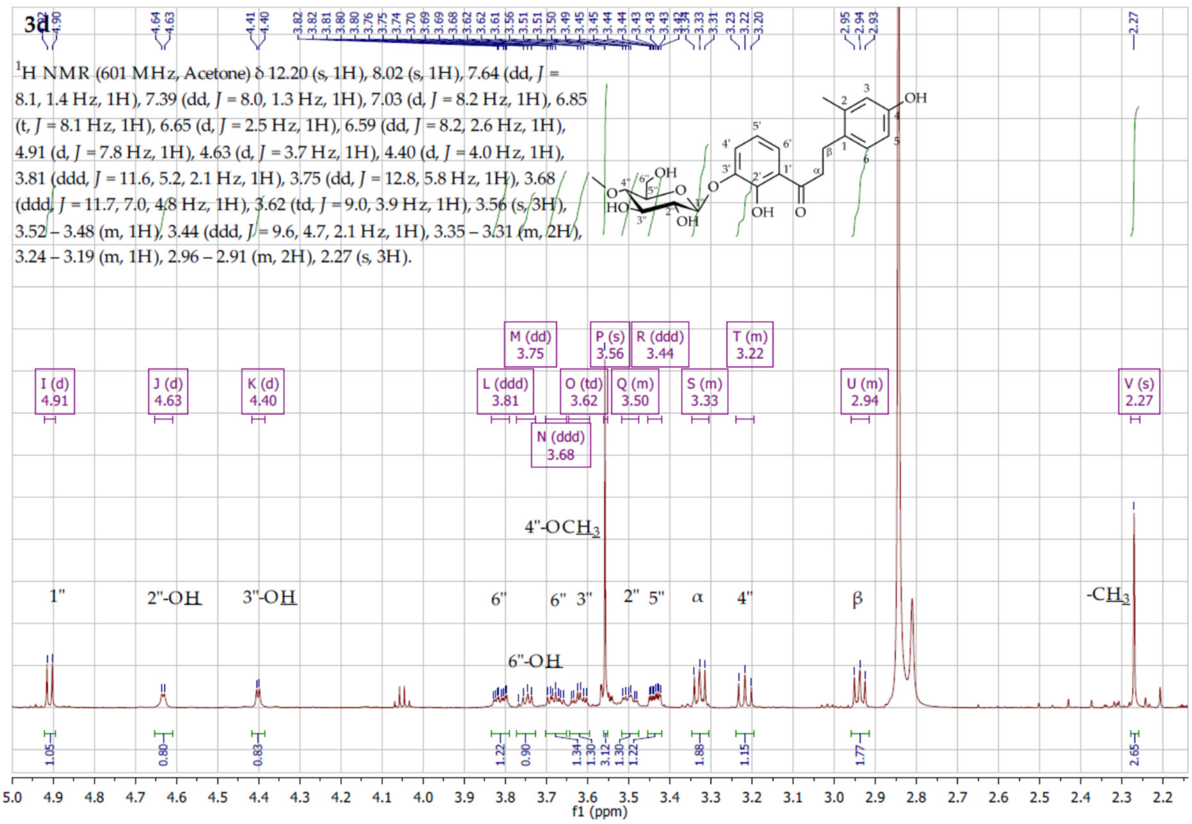

Figure S77. ^{1}H NMR spectrum expansion (*δ*, acetone-d6, 600 MHz) of 2′,4-dihydroxy-2-methyldihydrochalcone 3′-*O*-*β*-D-(4′′-*O*-methyl)-glucopyranoside (**3d**)

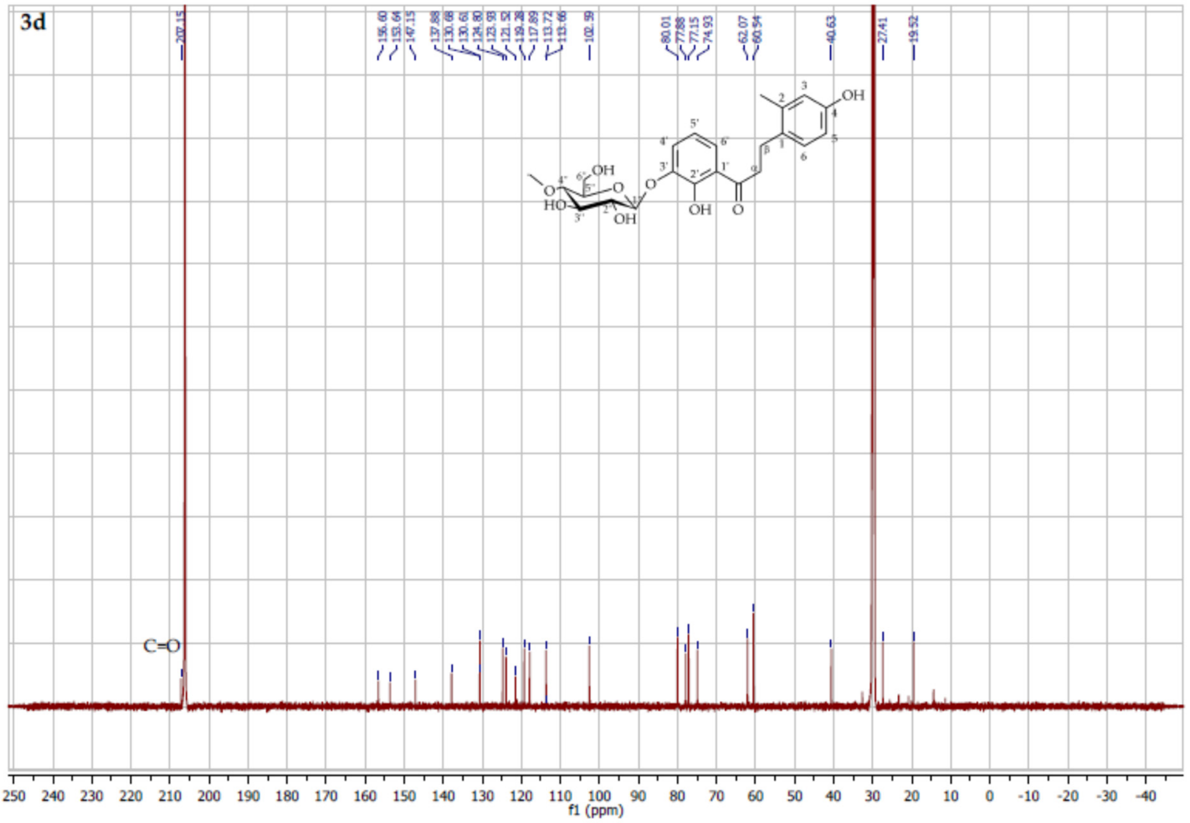

Figure S78. ^{13}C NMR spectrum (*δ*, acetone-d6, 151 MHz) of 2′,4-dihydroxy-2-methyldihydrochalcone 3′-*O*-*β*-D-(4′′-*O*-methyl)-glucopyranoside (**3d**)

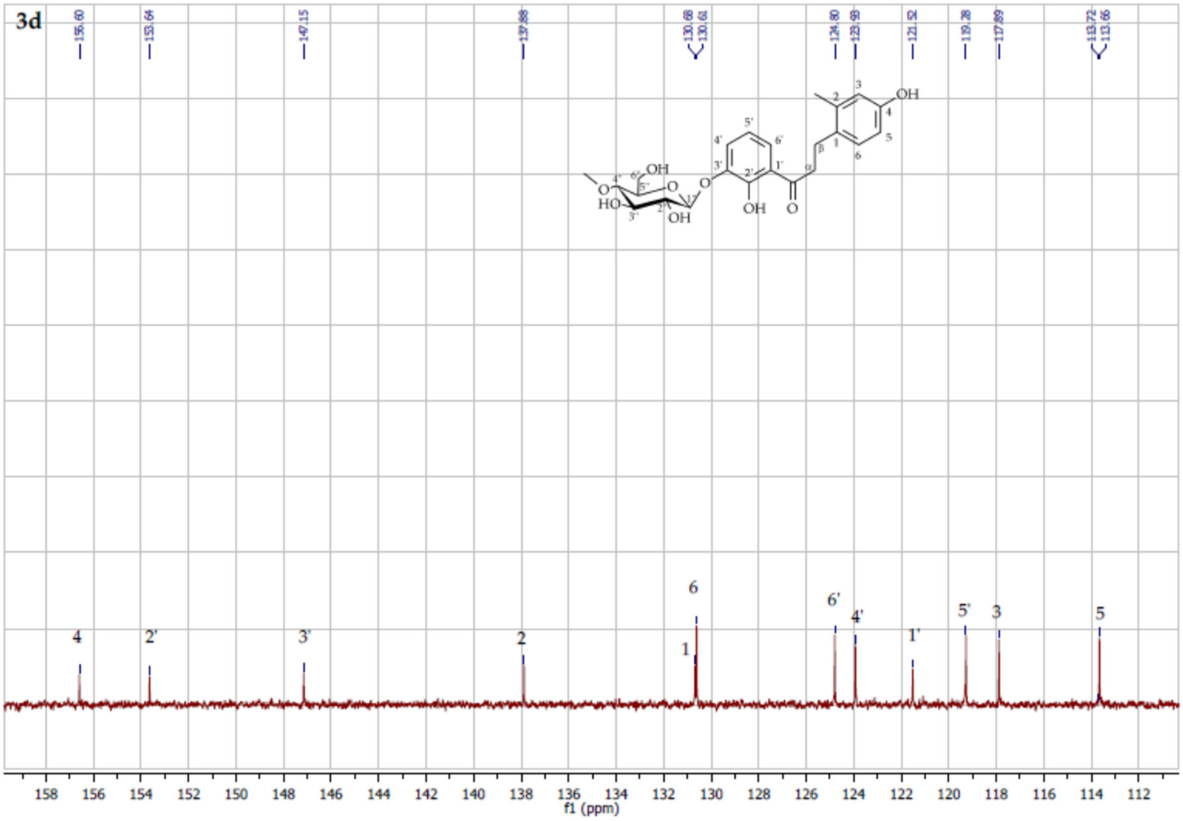

Figure S79. ^{13}C NMR spectrum expansion (*δ*, acetone-d6, 151 MHz) of 2′,4-dihydroxy-2-methyldihydrochalcone 3′-*O*-*β*-D-(4′′-*O*-methyl)-glucopyranoside (**3d**)

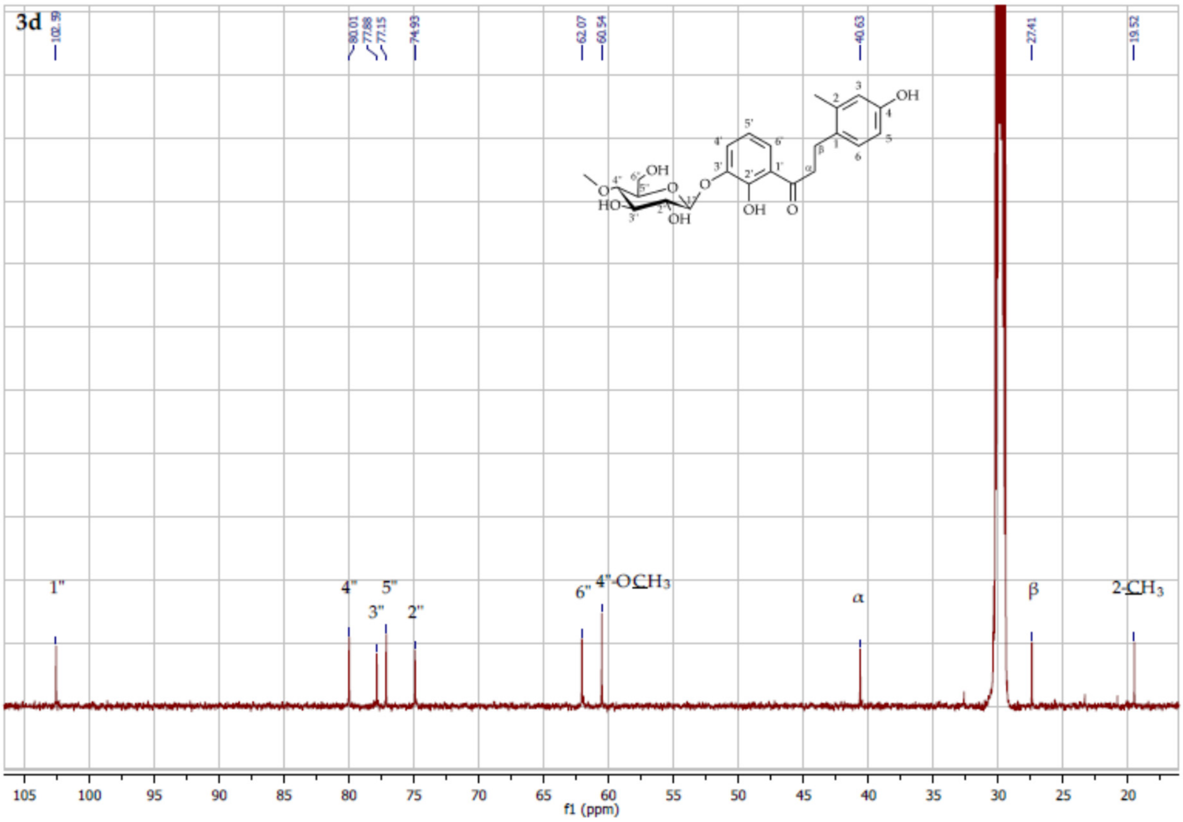

Figure S80. ^{13}C NMR spectrum expansion (*δ*, acetone-d6, 151 MHz) of 2′,4-dihydroxy-2-methyldihydrochalcone 3′-*O*-*β*-D-(4′′-*O*-methyl)-glucopyranoside (**3d**)

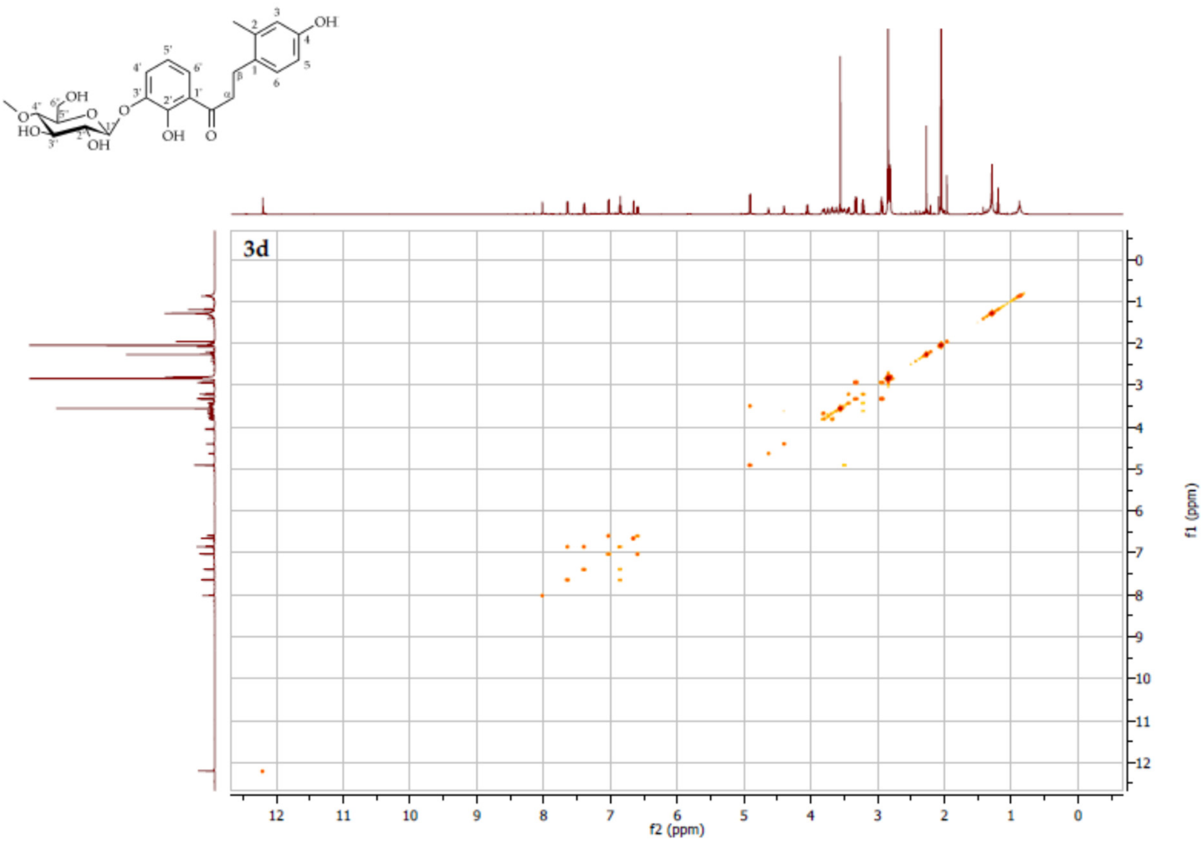

Figure S81. COSY contour map – ¹H x ¹Hof 2′,4-dihydroxy-2-methyldihydrochalcone 3′-*O*-*β*-D-(4′′-*O*-methyl)-glucopyranoside (**3d**)

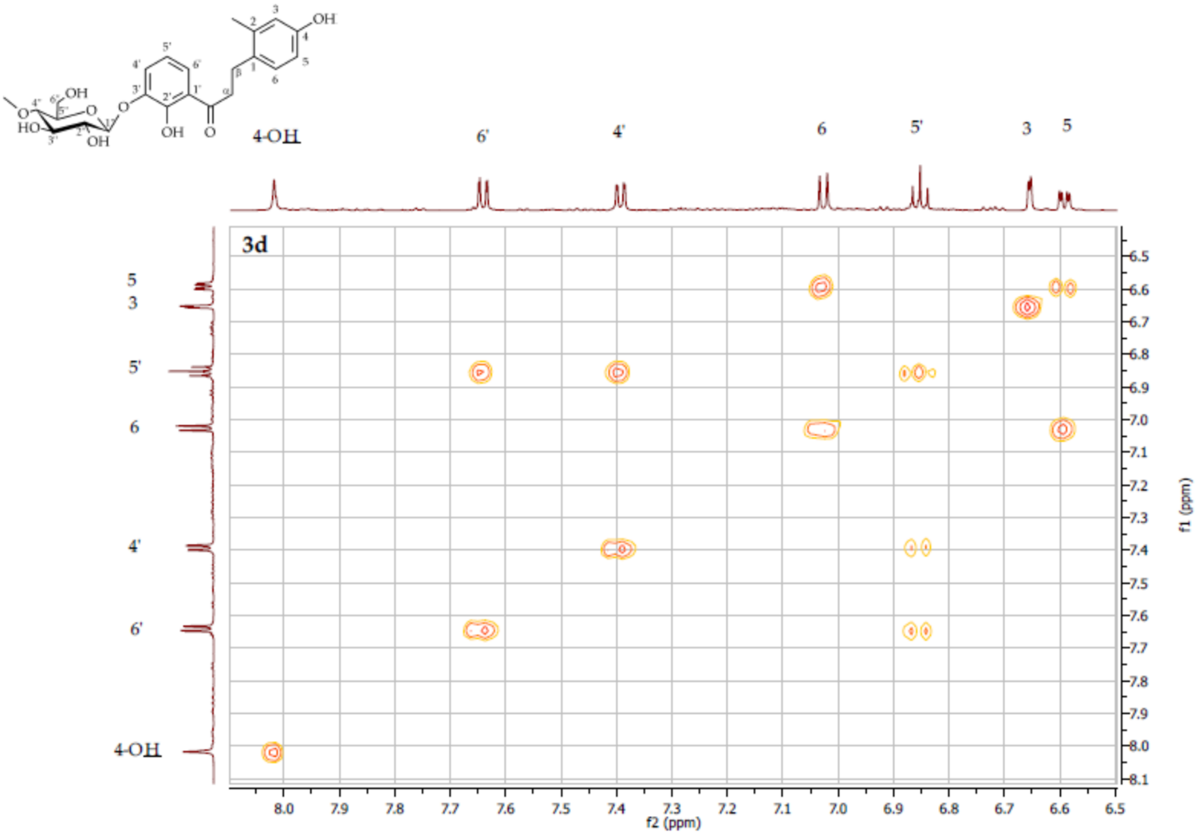

Figure S82. COSY contour map – ¹H x ¹Hexpansion of 2′,4-dihydroxy-2-methyldihydrochalcone 3′-*O*-*β*-D-(4′′-*O*-methyl)-glucopyranoside (**3d**)

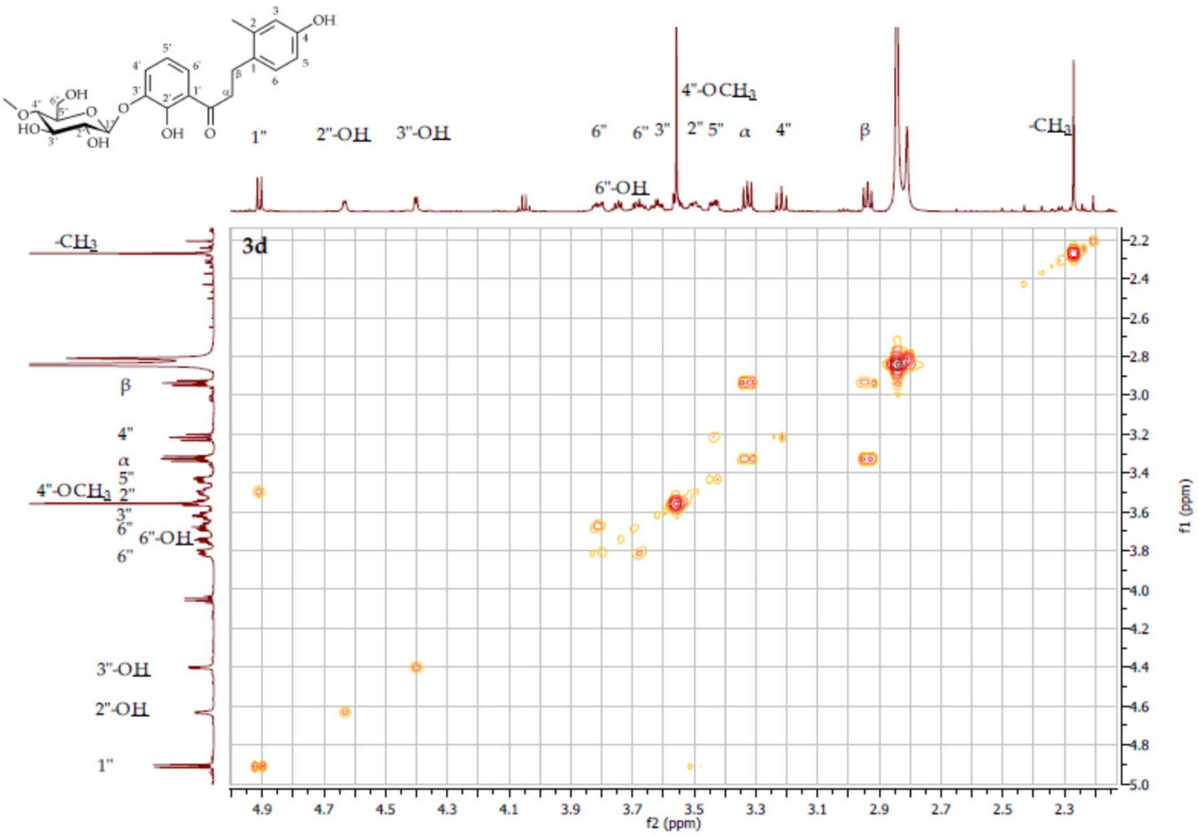

Figure S83. COSY contour map – ¹H x ¹Hexpansion of 2′,4-dihydroxy-2-methyldihydrochalcone 3′-*O*-*β*-D-(4′′-*O*-methyl)-glucopyranoside (**3d**)

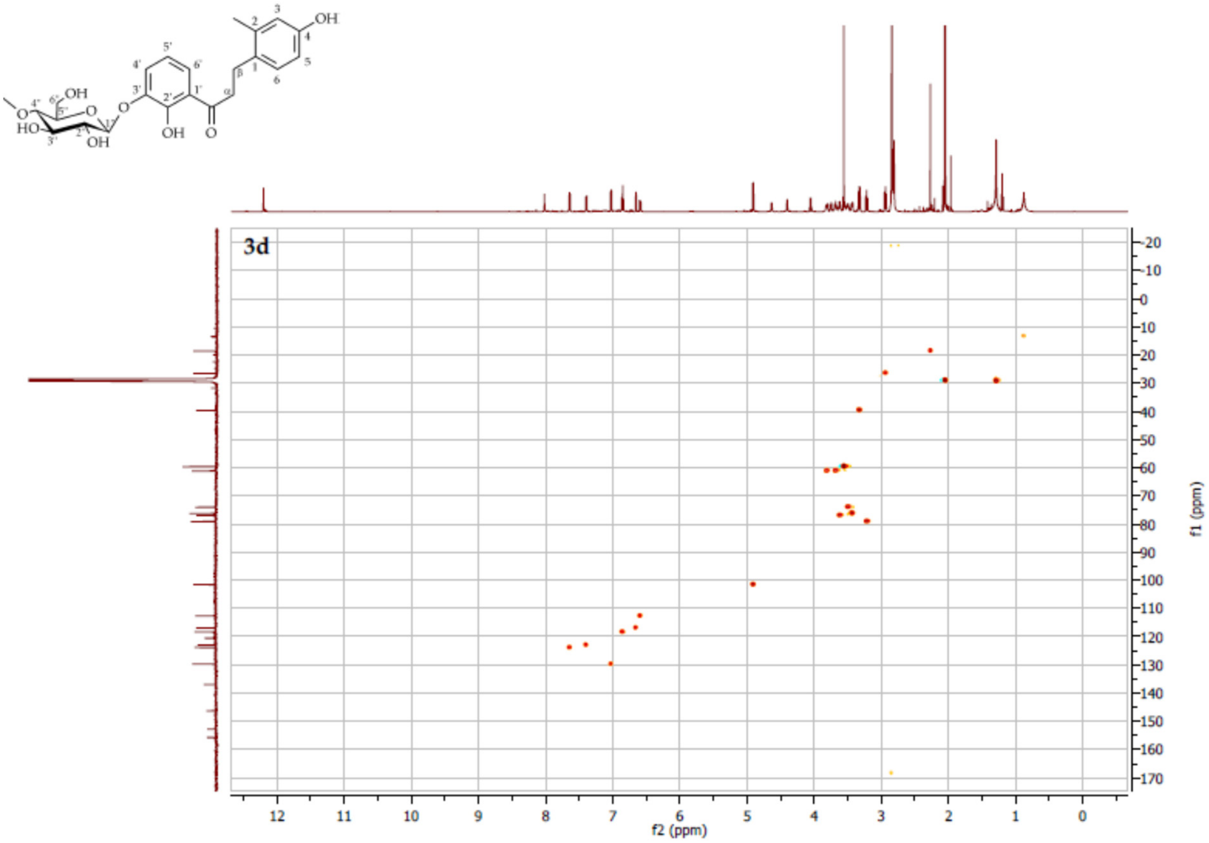

Figure S84. HSQC contour map– ¹H x ¹³C of 2′,4-dihydroxy-2-methyldihydrochalcone 3′-*O*-*β*-D-(4′′-*O*-methyl)-glucopyranoside (**3d**)

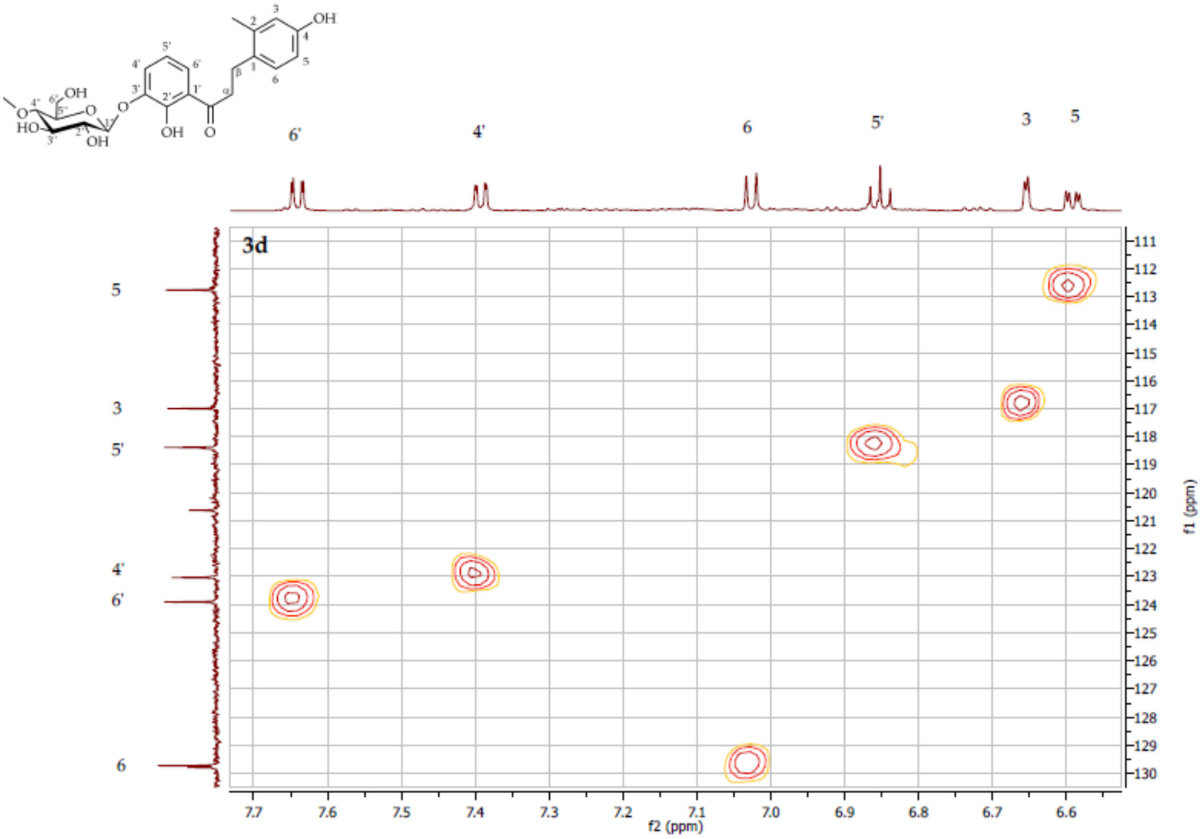

Figure S85. HSQC contour map– ^{1}H x ^{13}C expansion of 2′,4-dihydroxy-2-methyldihydrochalcone 3′-*O*-*β*-D-(4′′-*O*-methyl)-glucopyranoside (**3d**)

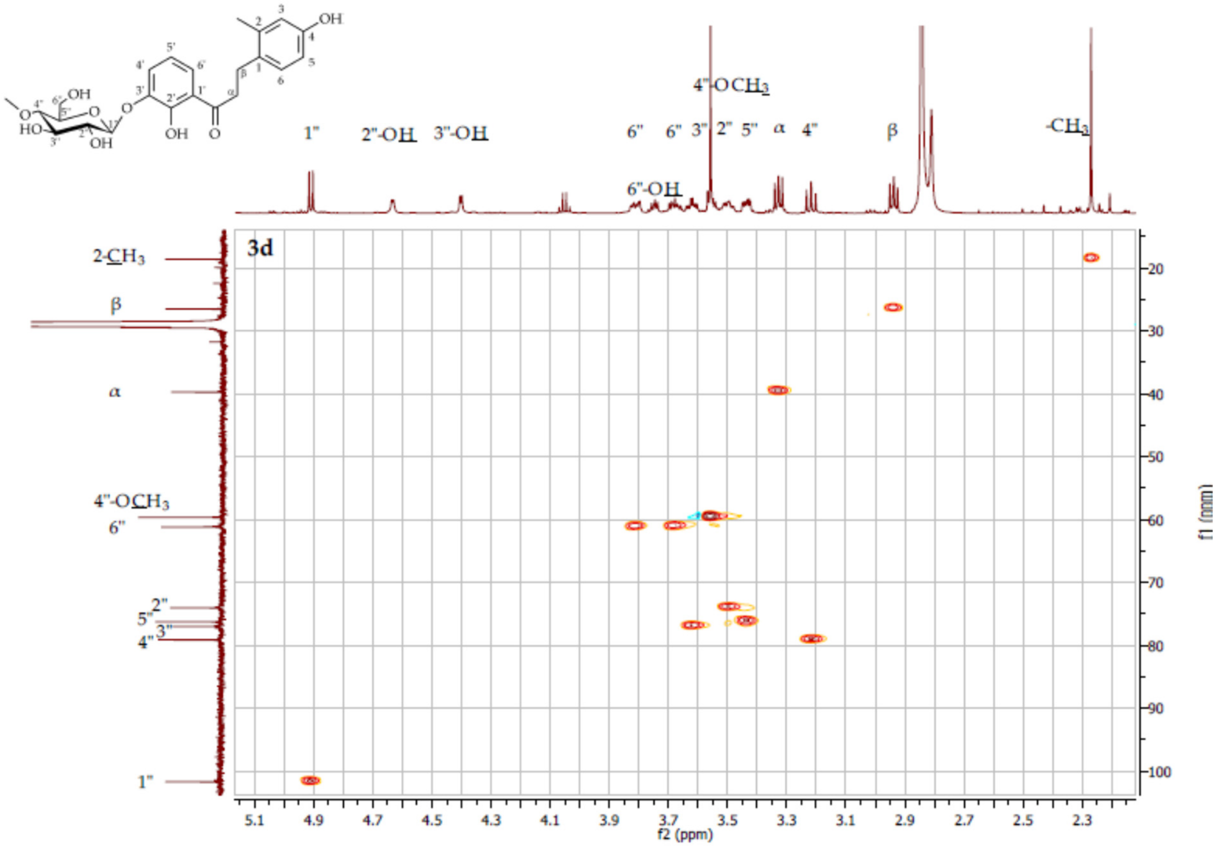

Figure S86. HSQC contour map– ^{1}H x ^{13}C expansion of 2′,4-dihydroxy-2-methyldihydrochalcone 3′-*O*-*β*-D-(4′′-*O*-methyl)-glucopyranoside (**3d**)

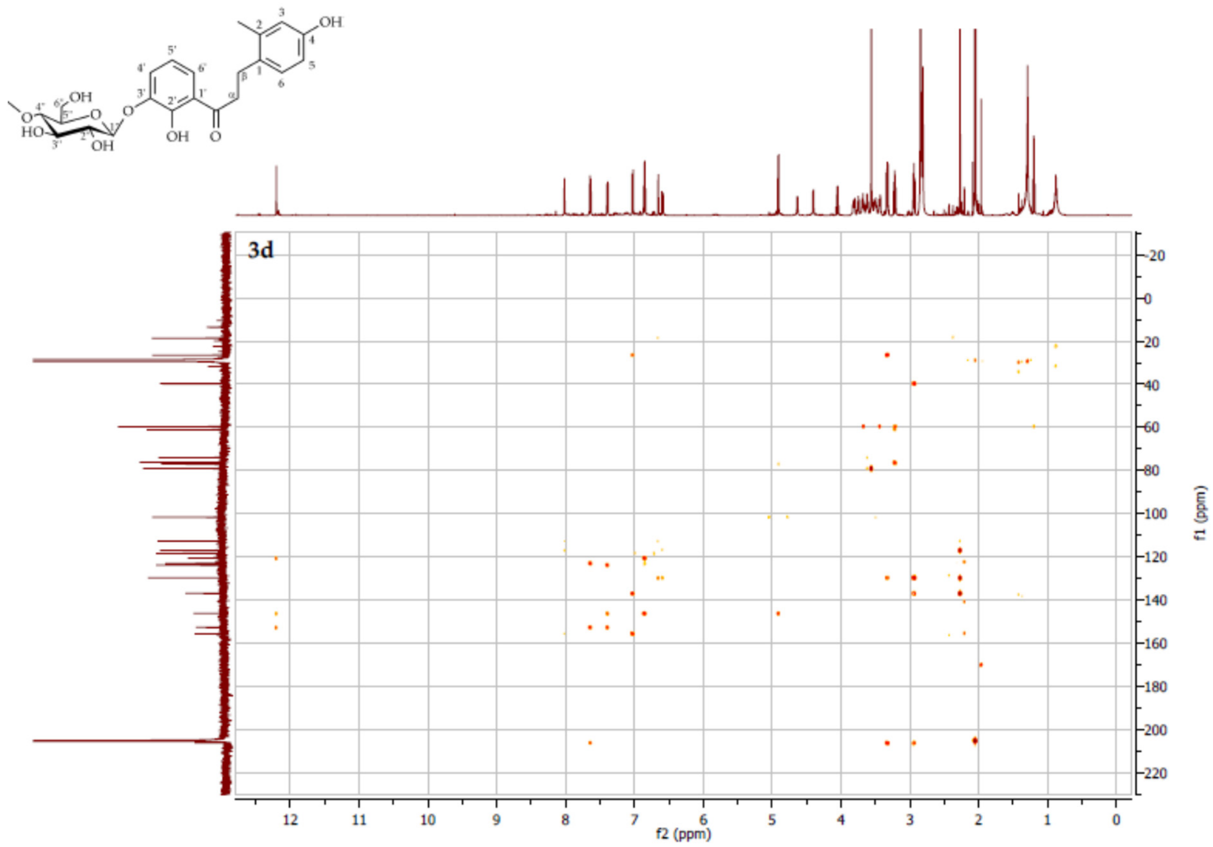

Figure S87. HMBC contour map– ^{1}H x ^{13}C of 2′,4-dihydroxy-2-methyldihydrochalcone 3′-*O*-*β*-D-(4′′-*O*-methyl)-glucopyranoside (**3d**)

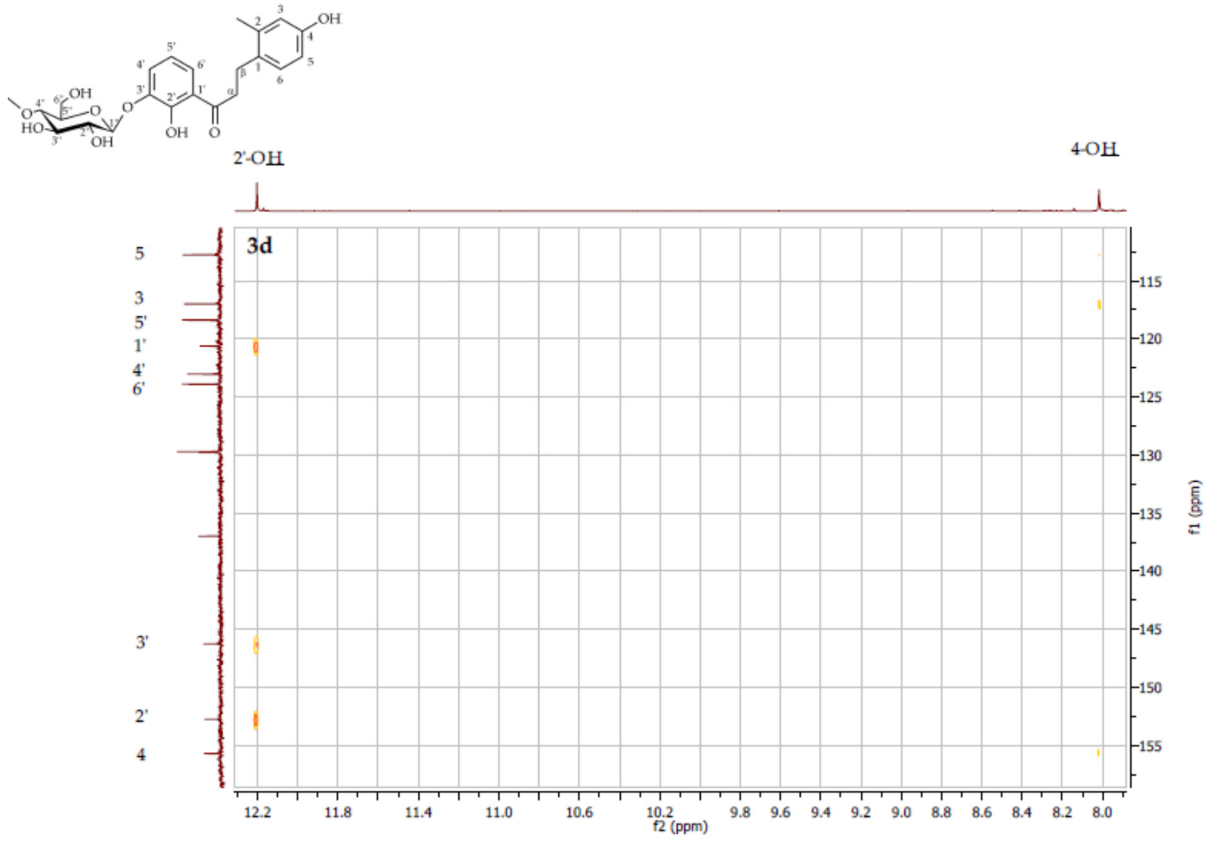

Figure S88. HMBC contour map– ^{1}H x ^{13}C expansion of 2′,4-dihydroxy-2-methyldihydrochalcone 3′-*O*-*β*-D-(4′′-*O*-methyl)-glucopyranoside (**3d**)

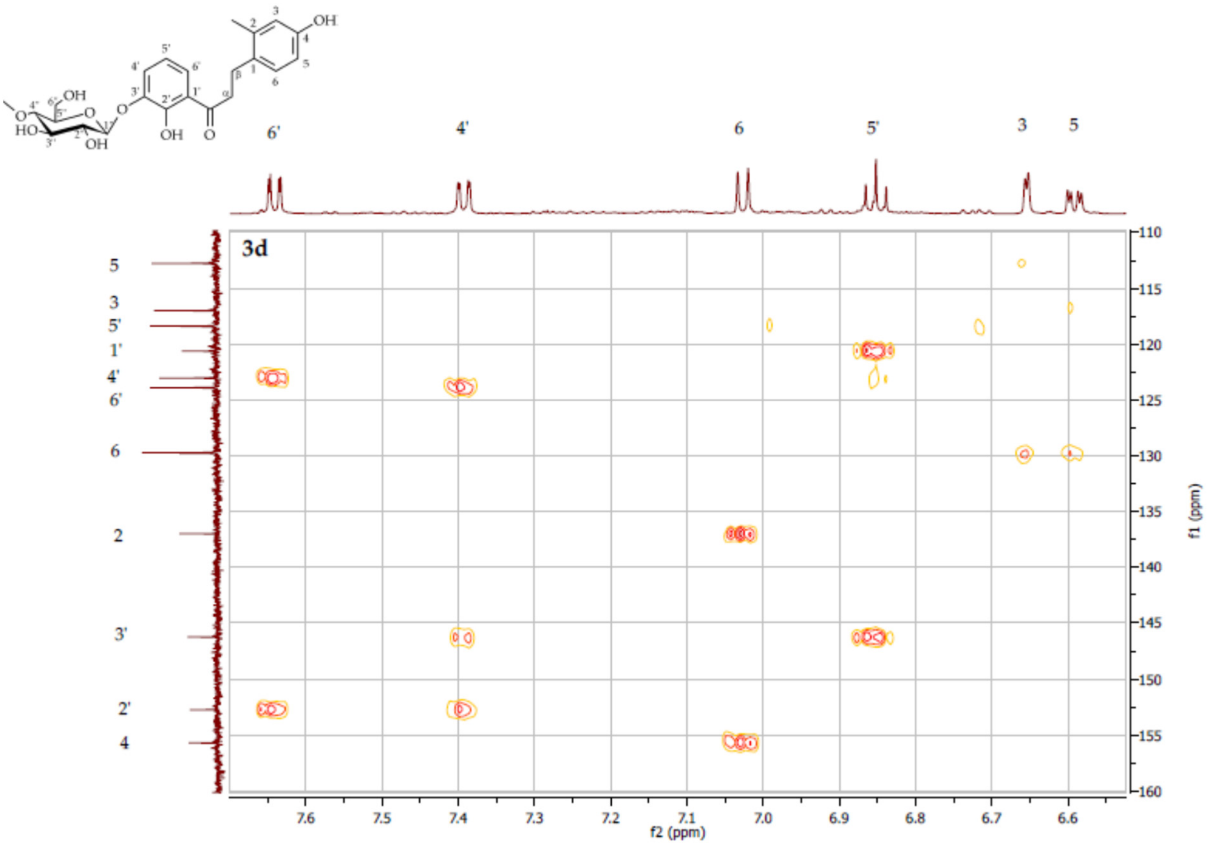

Figure S89. HMBC contour map– ^{1}H x ^{13}C expansion of 2′,4-dihydroxy-2-methyldihydrochalcone 3′-*O*-*β*-D-(4′′-*O*-methyl)-glucopyranoside (**3d**)

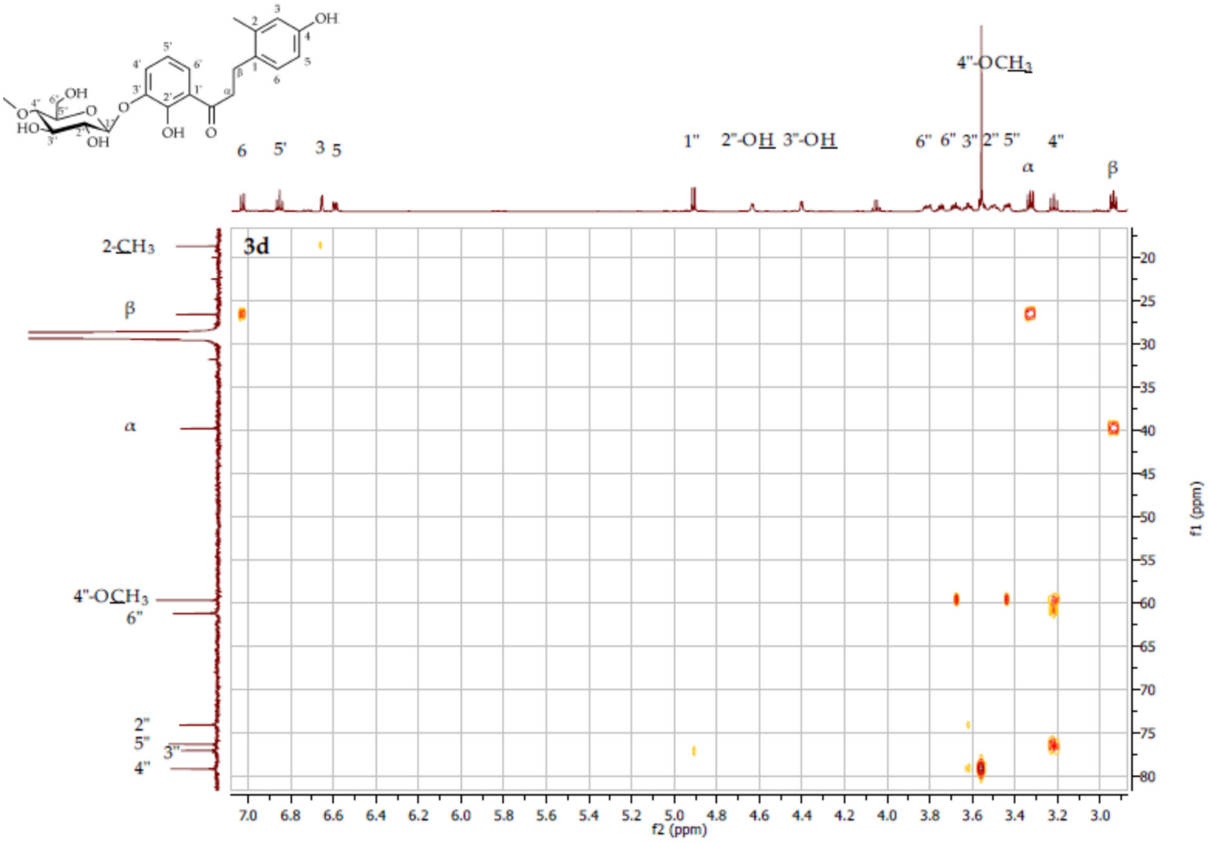

Figure S90. HMBC contour map– ^{1}H x ^{13}C expansion of 2′,4-dihydroxy-2-methyldihydrochalcone 3′-*O*-*β*-D-(4′′-*O*-methyl)-glucopyranoside (**3d**)

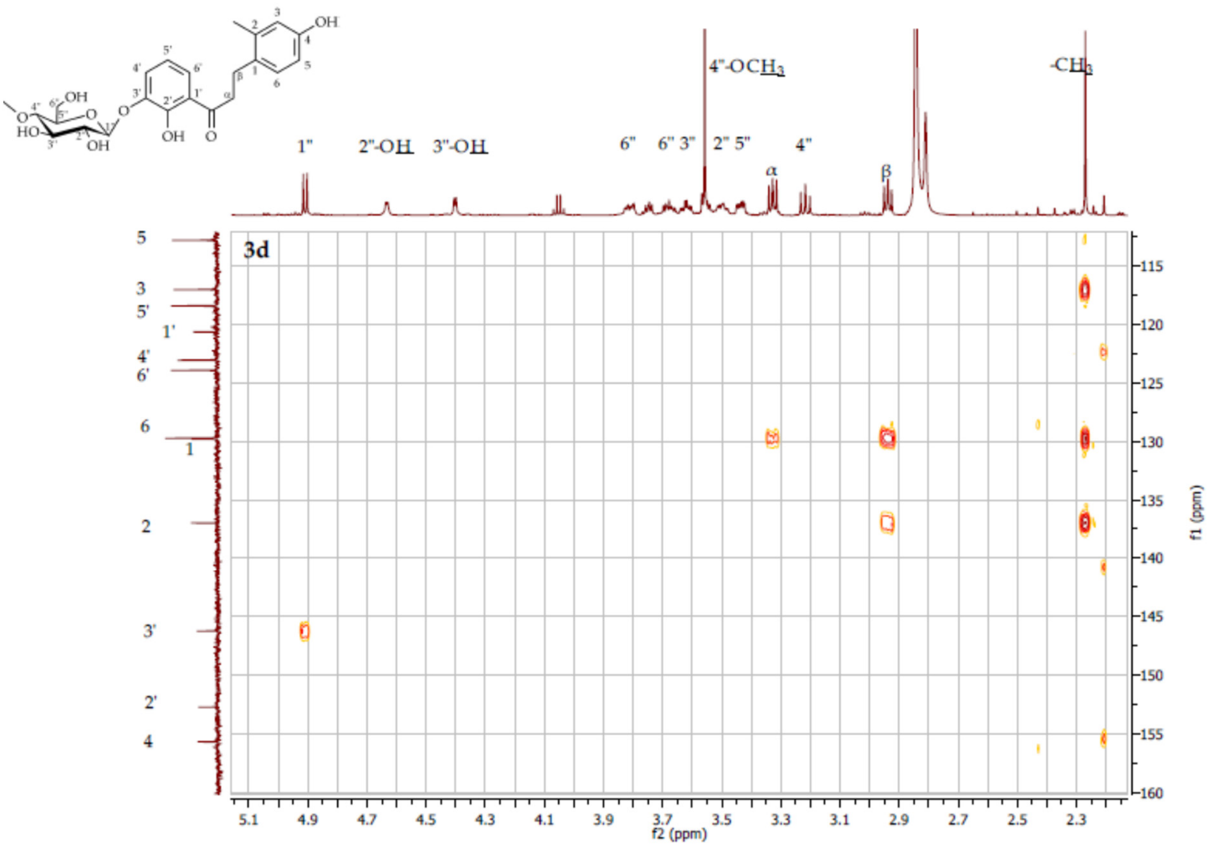

Figure S91. HMBC contour map– ^1H x ^{13}C expansion of 2′,4-dihydroxy-2-methyldihydrochalcone 3′-*O*-*β*-D-(4′′-*O*-methyl)-glucopyranoside (**3d**)

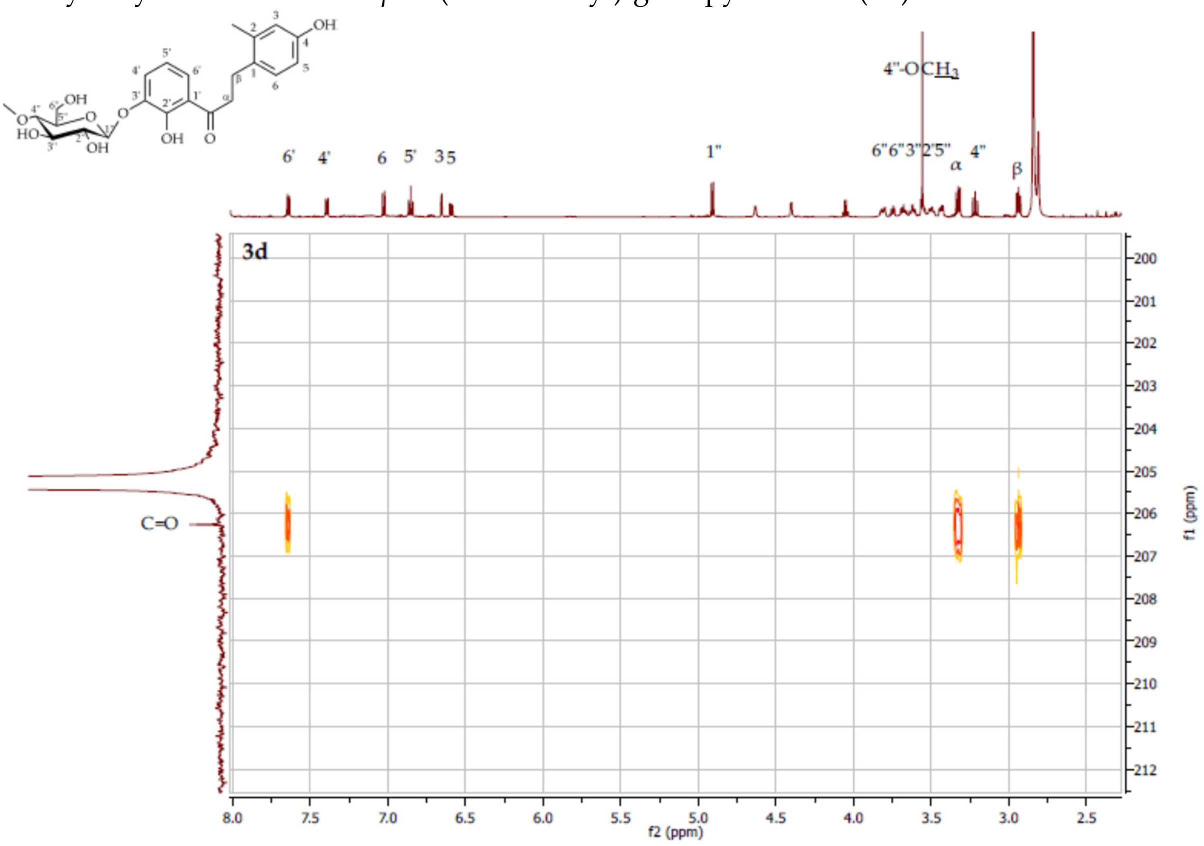

Figure S92. HMBC contour map– ^1H x ^{13}C expansion of 2′,4-dihydroxy-2-methyldihydrochalcone 3′-*O*-*β*-D-(4′′-*O*-methyl)-glucopyranoside (**3d**)

Molecular formula: $C_{23}H_{28}O_8$
Formula weight:432.18
Ionization mode:negative
Precursor: $[M-H]^-$ 431.30
431.3000>237.1000 CE: 24.0
431.3000>255.0500 CE: 13.0
431.3000>222.0500 CE: 42.0

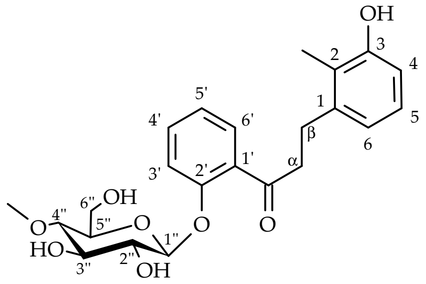

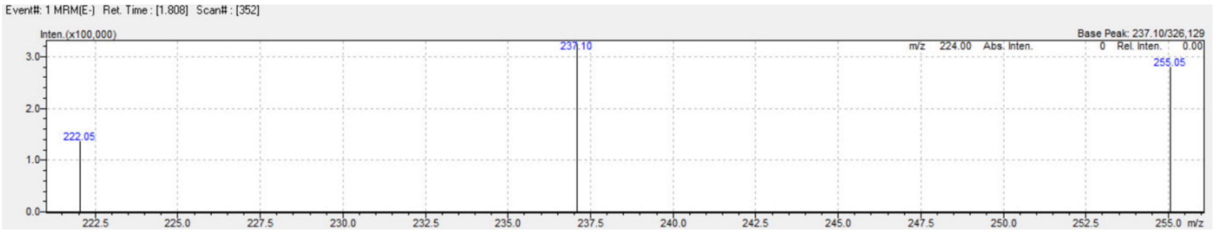

Figure S93.MS analysis of 3-hydroxy-2-methyldihydrochalcone 2′-*O*-*β*-D-(4′′-*O*-methyl)-glucopyranoside (**3e**)

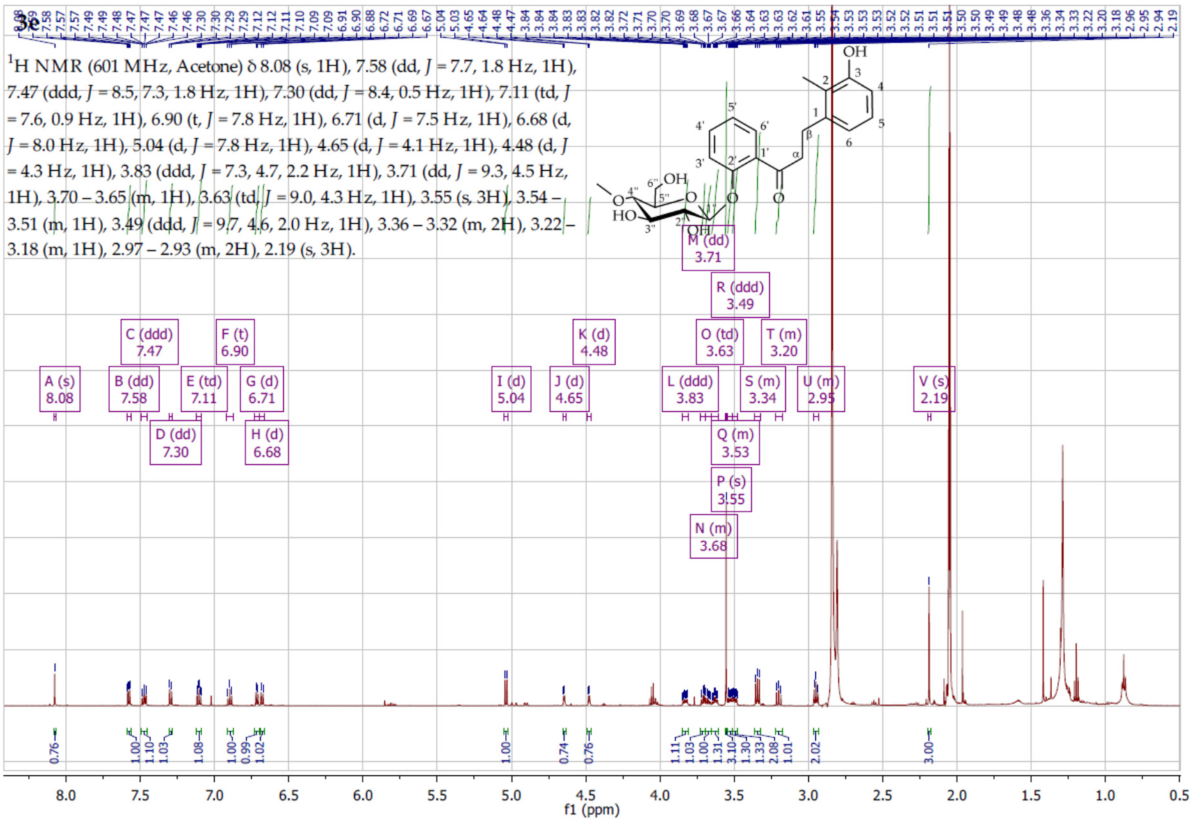

Figure S94. ^{1}H NMR spectrum (*δ*, acetone-d6, 600 MHz) of 3-hydroxy-2-methyldihydrochalcone 2′-*O*-*β*-D-(4′′-*O*-methyl)-glucopyranoside (**3e**)

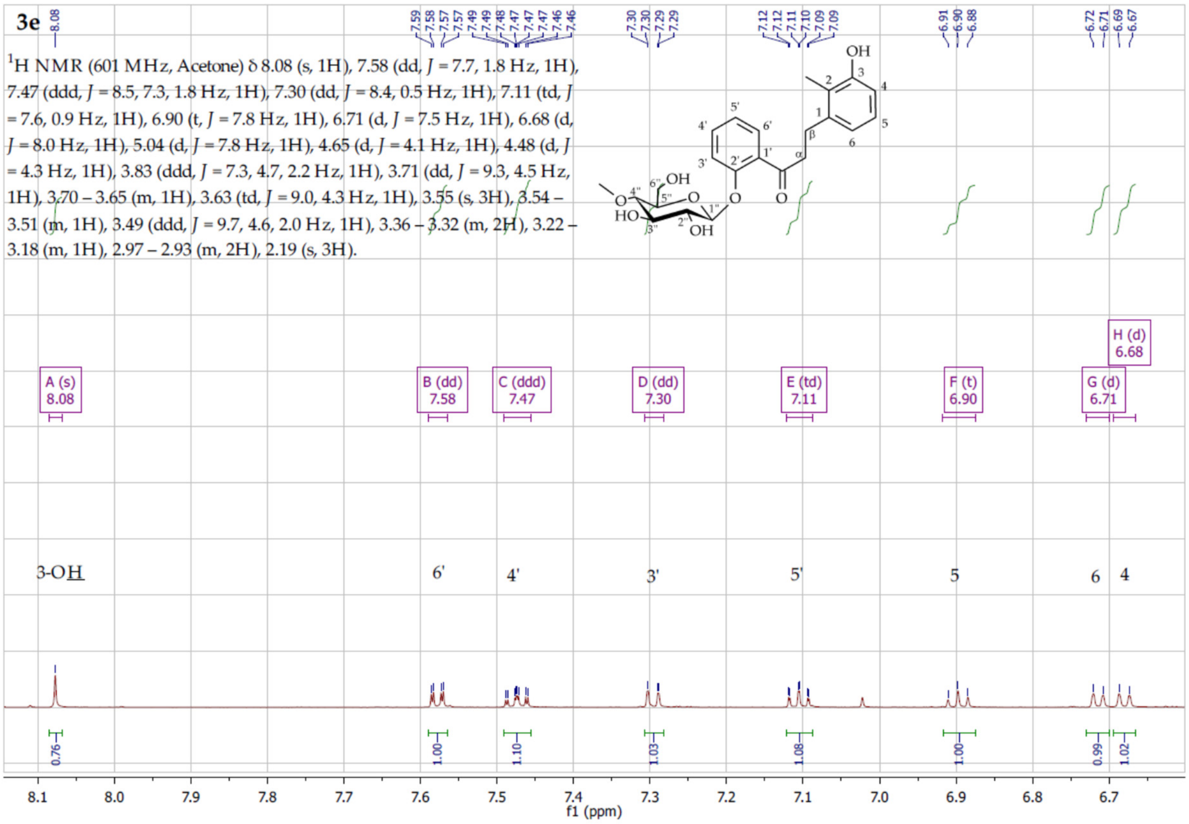

Figure S95. ^{1}H NMR spectrum expansion (*δ*, acetone-d6, 600 MHz) of 3-hydroxy-2-methyldihydrochalcone 2′-*O*-*β*-D-(4′′-*O*-methyl)-glucopyranoside (**3e**)

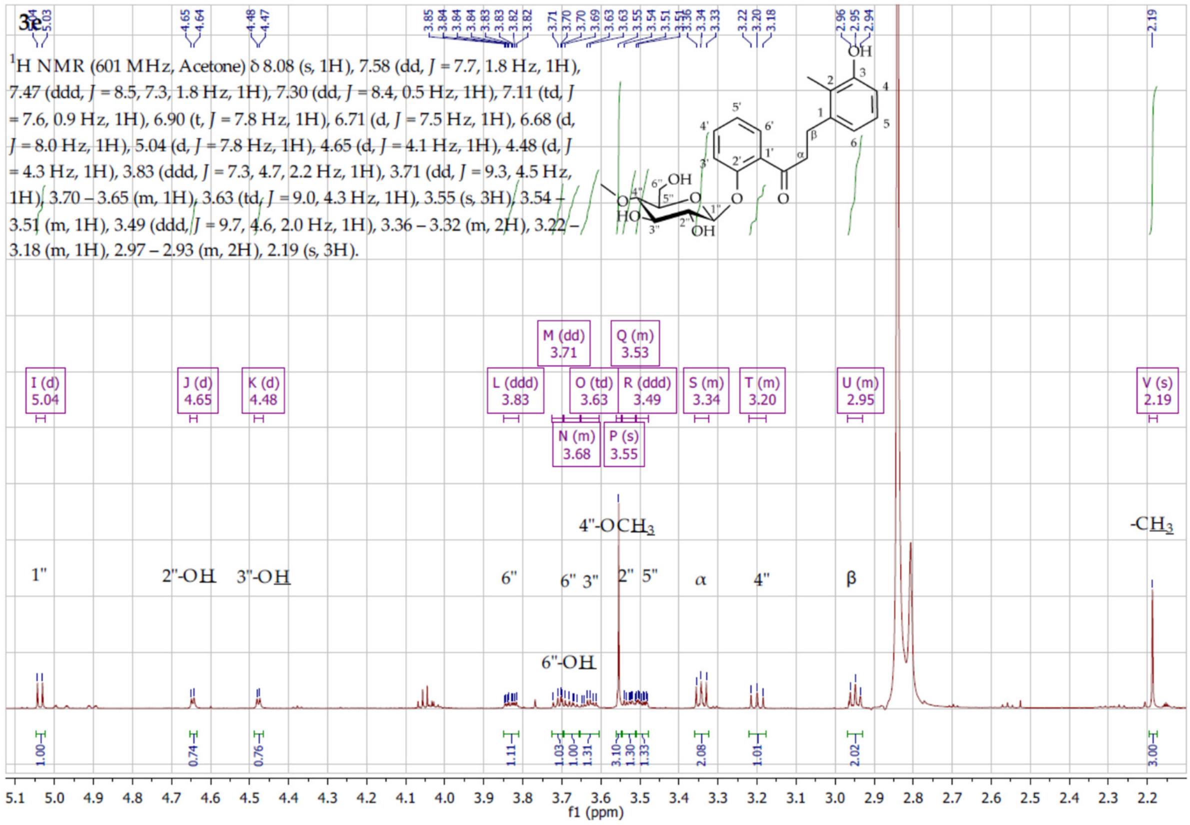

Figure S96. ^{1}H NMR spectrum expansion (*δ,* acetone-d6, 600 MHz) of 3-hydroxy-2-methyldihydrochalcone 2′-*O*-*β*-D-(4′′-*O*-methyl)-glucopyranoside (**3e**)

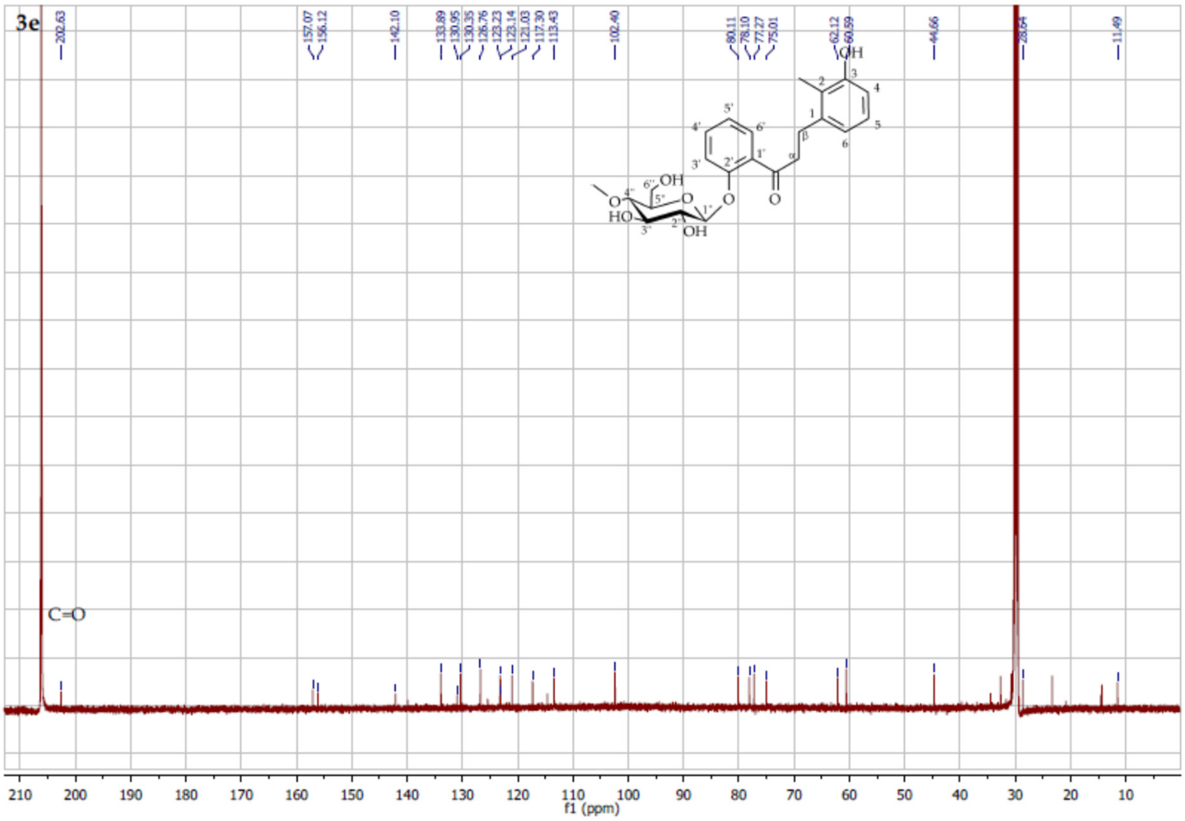

Figure S97. ^{13}C NMR spectrum (*δ*, acetone-d6, 151 MHz) of 3-hydroxy-2-methyldihydrochalcone 2′-*O*-*β*-D-(4′′-*O*-methyl)-glucopyranoside (**3e**)

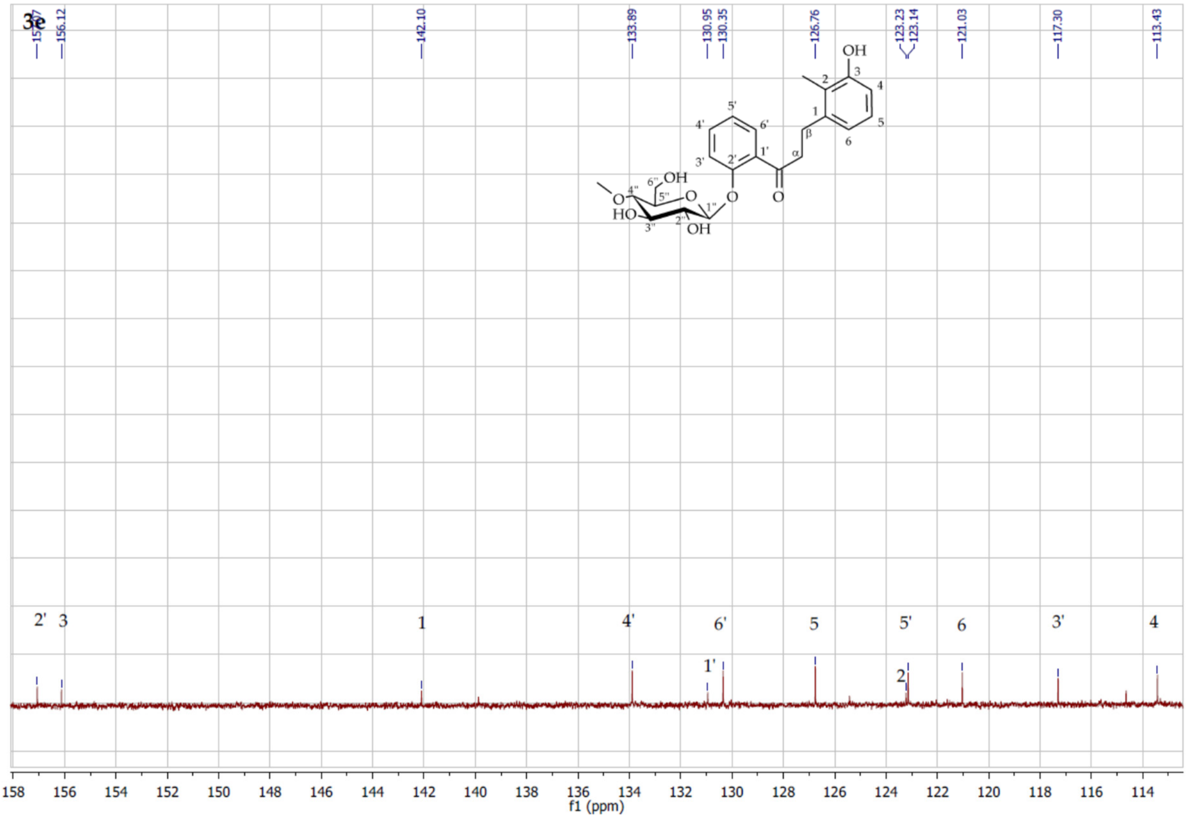

Figure S98. ^{13}C NMR spectrum expansion (*δ*, acetone-d6, 151 MHz) of 3-hydroxy-2-methyldihydrochalcone 2′-*O*-*β*-D-(4′′-*O*-methyl)-glucopyranoside (**3e**)

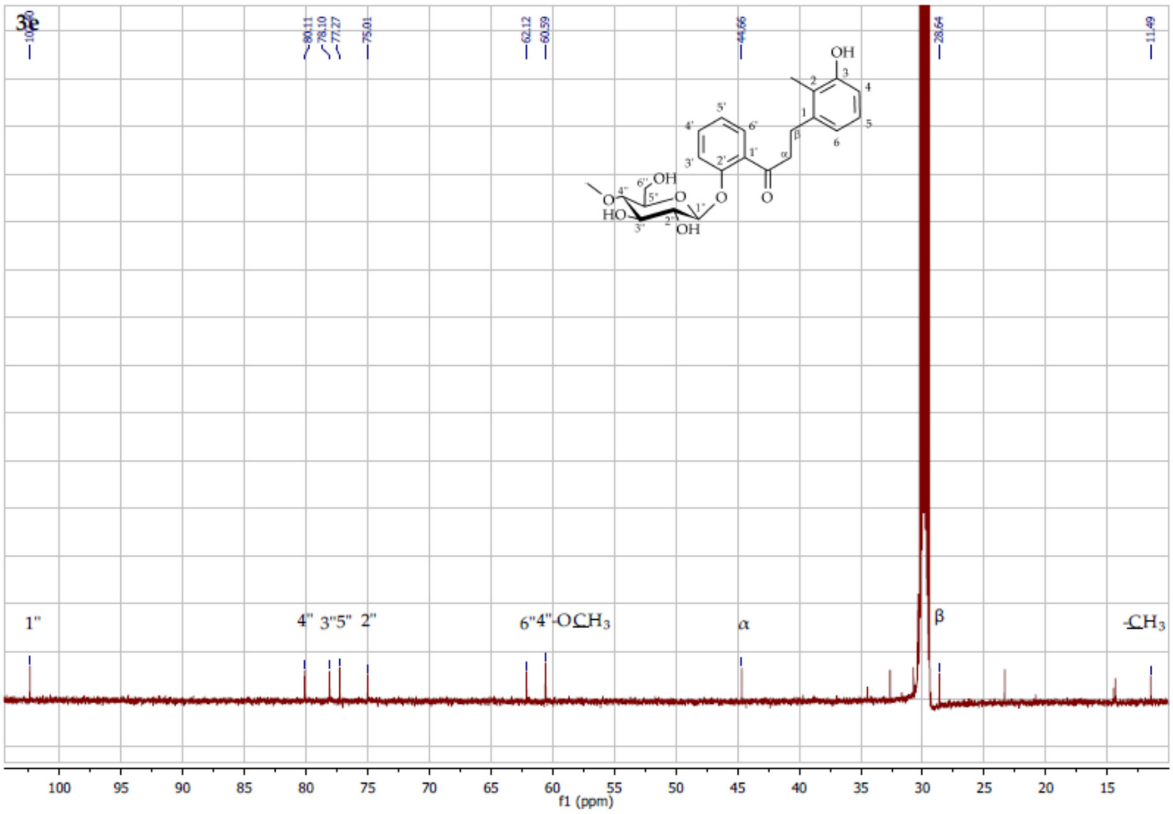

Figure S99. ^{13}C NMR spectrum expansion (δ, acetone-d6, 151 MHz) of 3-hydroxy-2-methyldihydrochalcone 2′-*O*-*β*-D-(4′′-*O*-methyl)-glucopyranoside (**3e**)

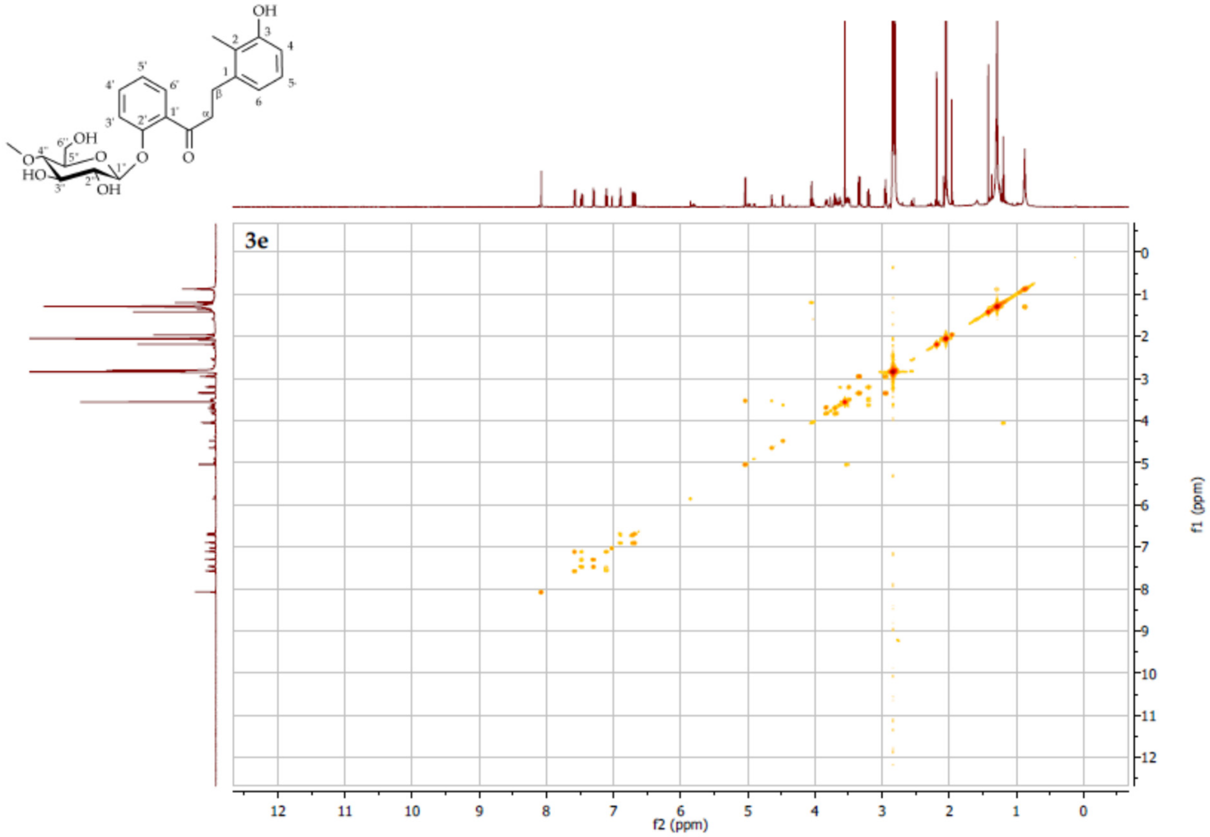

Figure S100. COSY contour map – ^1H x $^1H$of 3-hydroxy-2-methyldihydrochalcone 2′-*O*-*β*-D-(4′′-*O*-methyl)-glucopyranoside (**3e**)

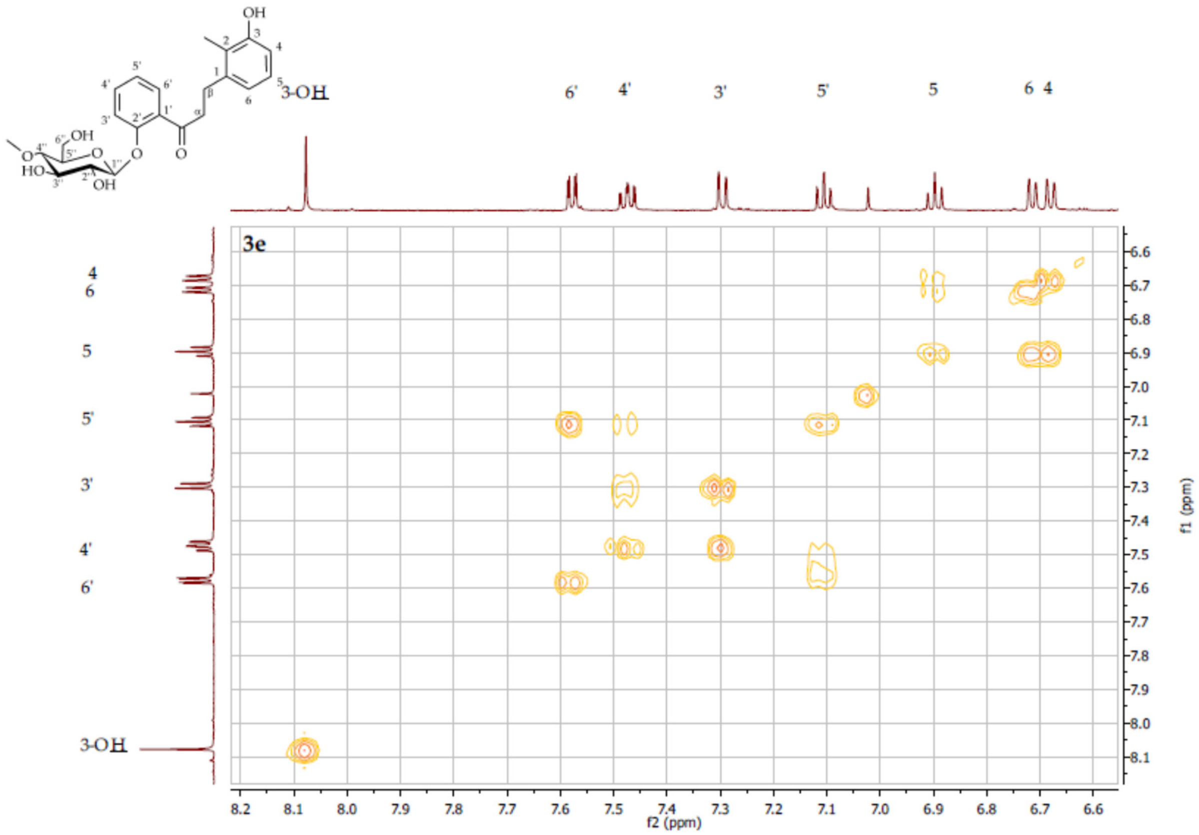

Figure S101. COSY contour map – ¹H x ¹Hexpansion of 3-hydroxy-2-methyldihydrochalcone 2′-*O*-*β*-D-(4′′-*O*-methyl)-glucopyranoside (**3e**)

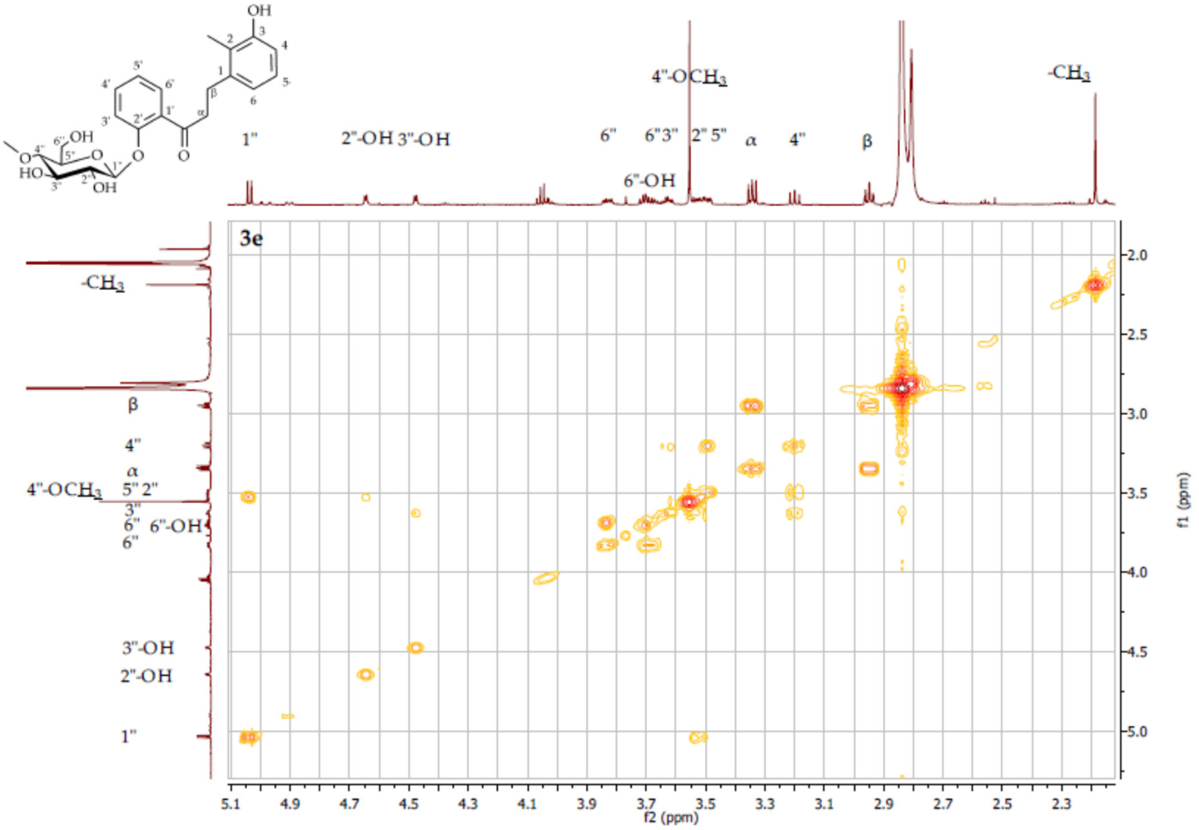

Figure S102. COSY contour map – ¹H x ¹Hexpansion of 3-hydroxy-2-methyldihydrochalcone 2′-*O*-*β*-D-(4′′-*O*-methyl)-glucopyranoside (**3e**)

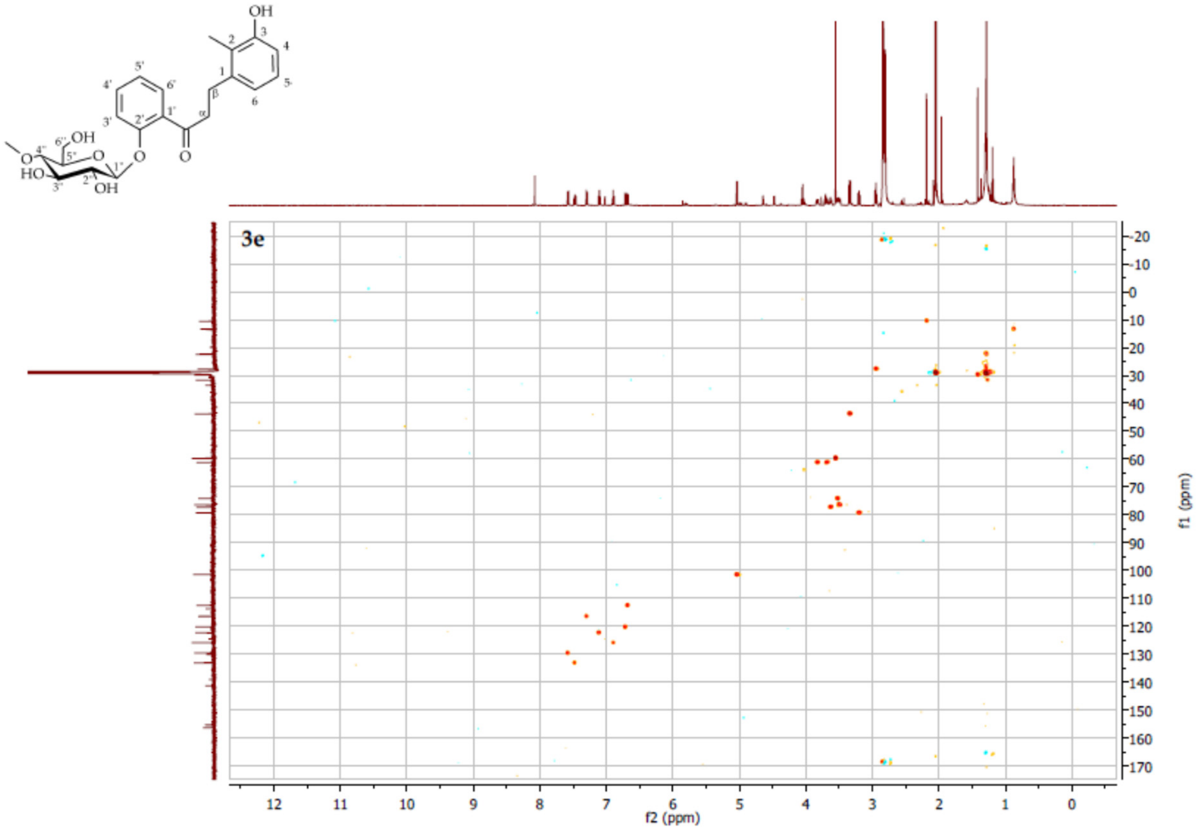

Figure S103. HSQC contour map– ^{1}H x ^{13}C of 3-hydroxy-2-methyldihydrochalcone 2′-*O*-*β*-D-(4′′-*O*-methyl)-glucopyranoside (**3e**)

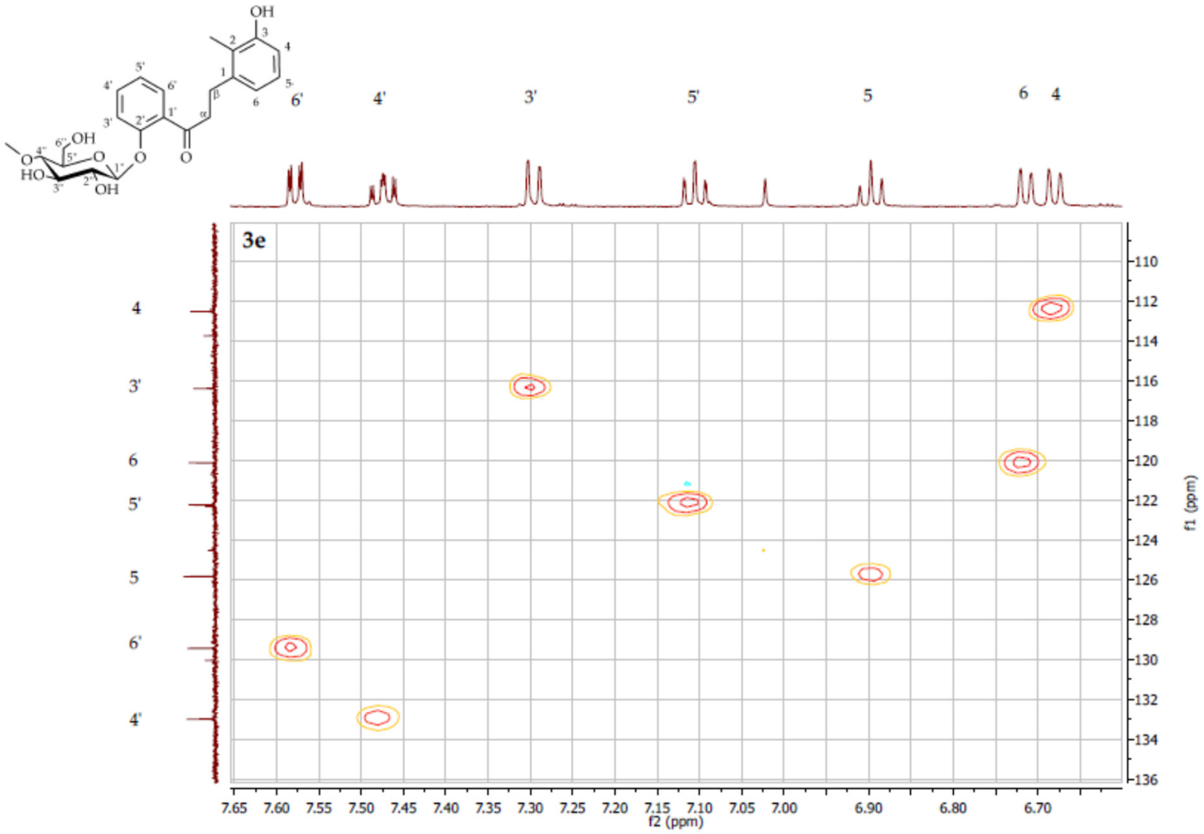

Figure S104. HSQC contour map– ^{1}H x ^{13}C expansion of 3-hydroxy-2-methyldihydrochalcone 2′-*O*-*β*-D-(4′′-*O*-methyl)-glucopyranoside (**3e**)

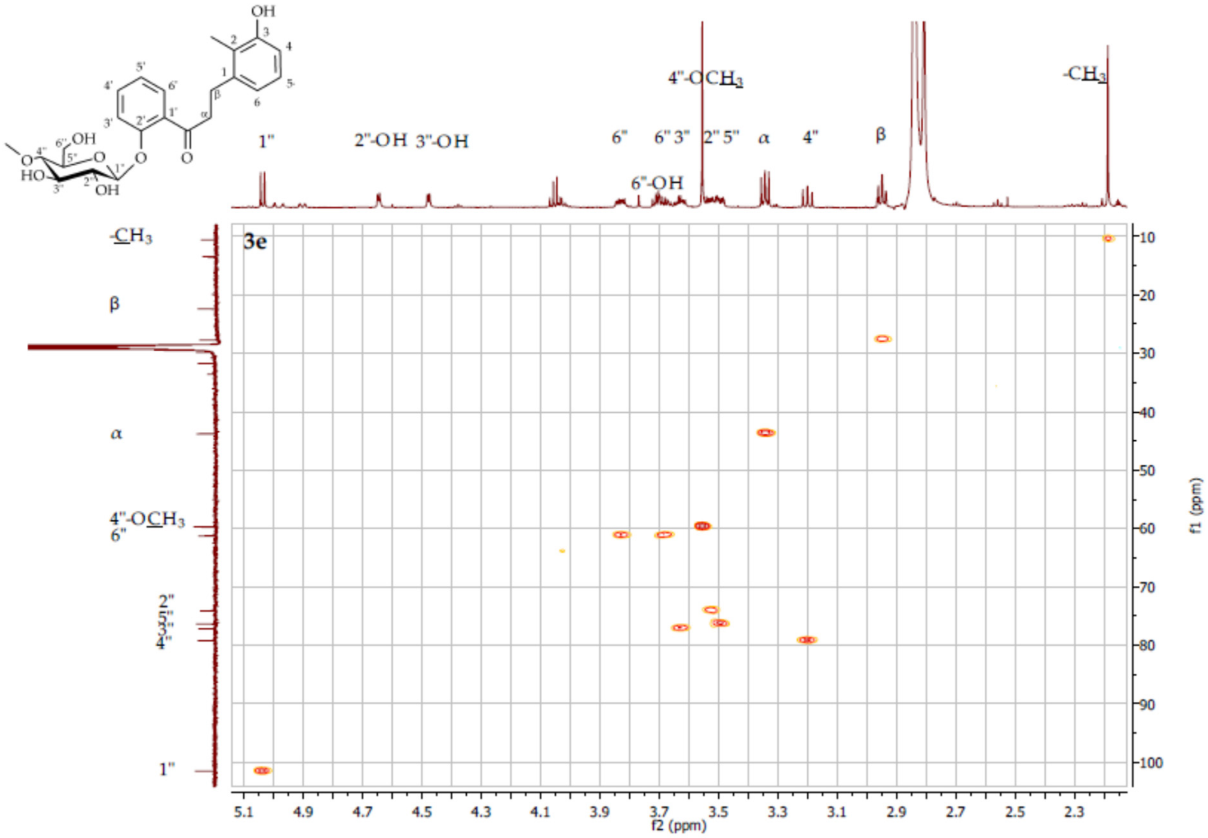

Figure S105. HSQC contour map– ^{1}H x ^{13}C expansion of 3-hydroxy-2-methyldihydrochalcone 2′-*O*-*β*-D-(4′′-*O*-methyl)-glucopyranoside (**3e**)

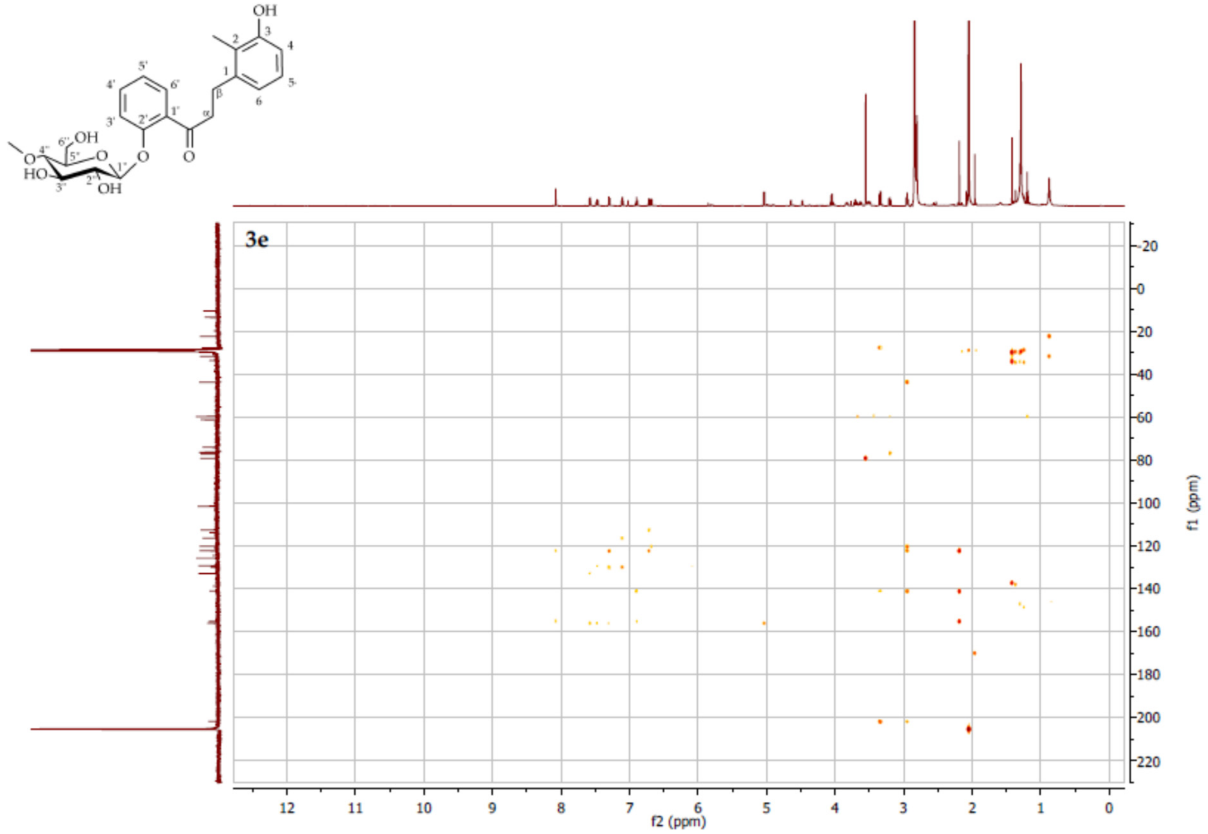

Figure S106. HMBC contour map– ^{1}H x ^{13}C of 3-hydroxy-2-methyldihydrochalcone 2′-*O*-*β*-D-(4′′-*O*-methyl)-glucopyranoside (**3e**)

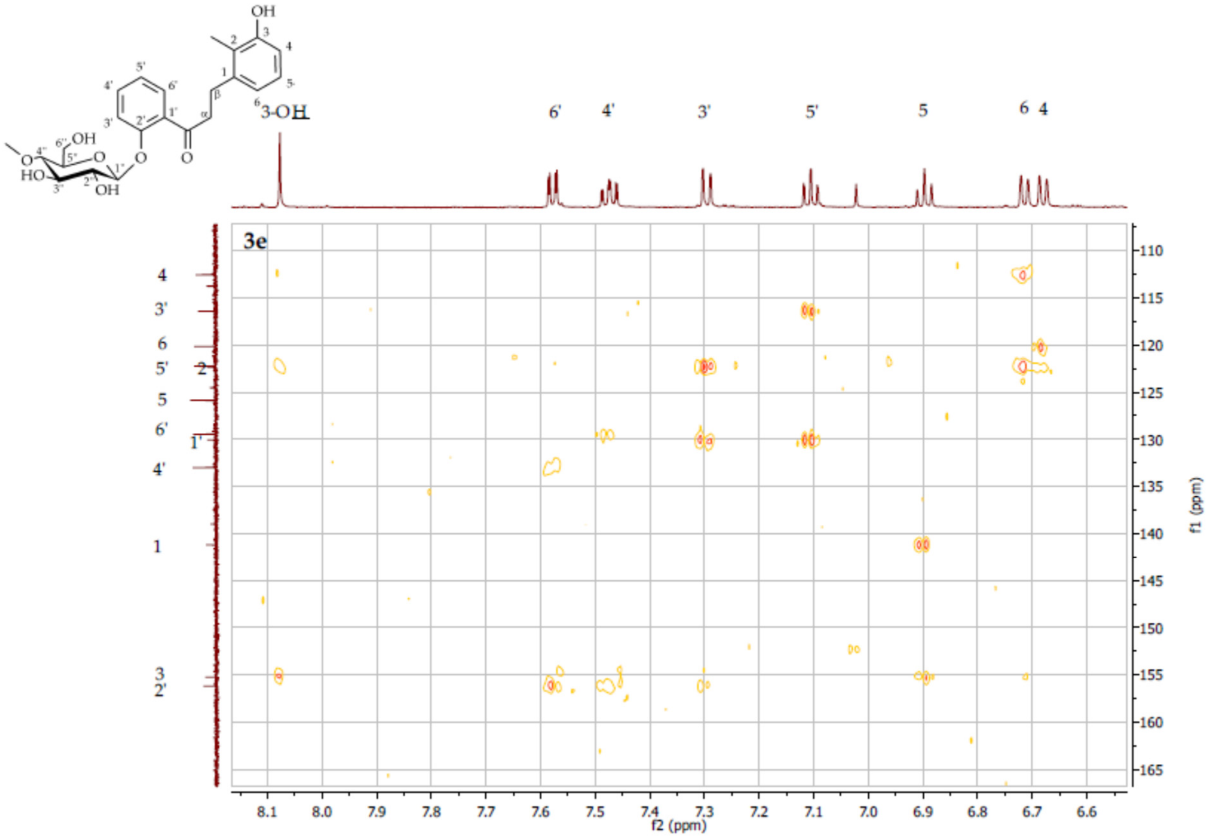

Figure S107. HMBC contour map– ^{1}H x ^{13}C expansion of 3-hydroxy-2-methyldihydrochalcone 2′-*O*-*β*-D-(4′′-*O*-methyl)-glucopyranoside (**3e**)

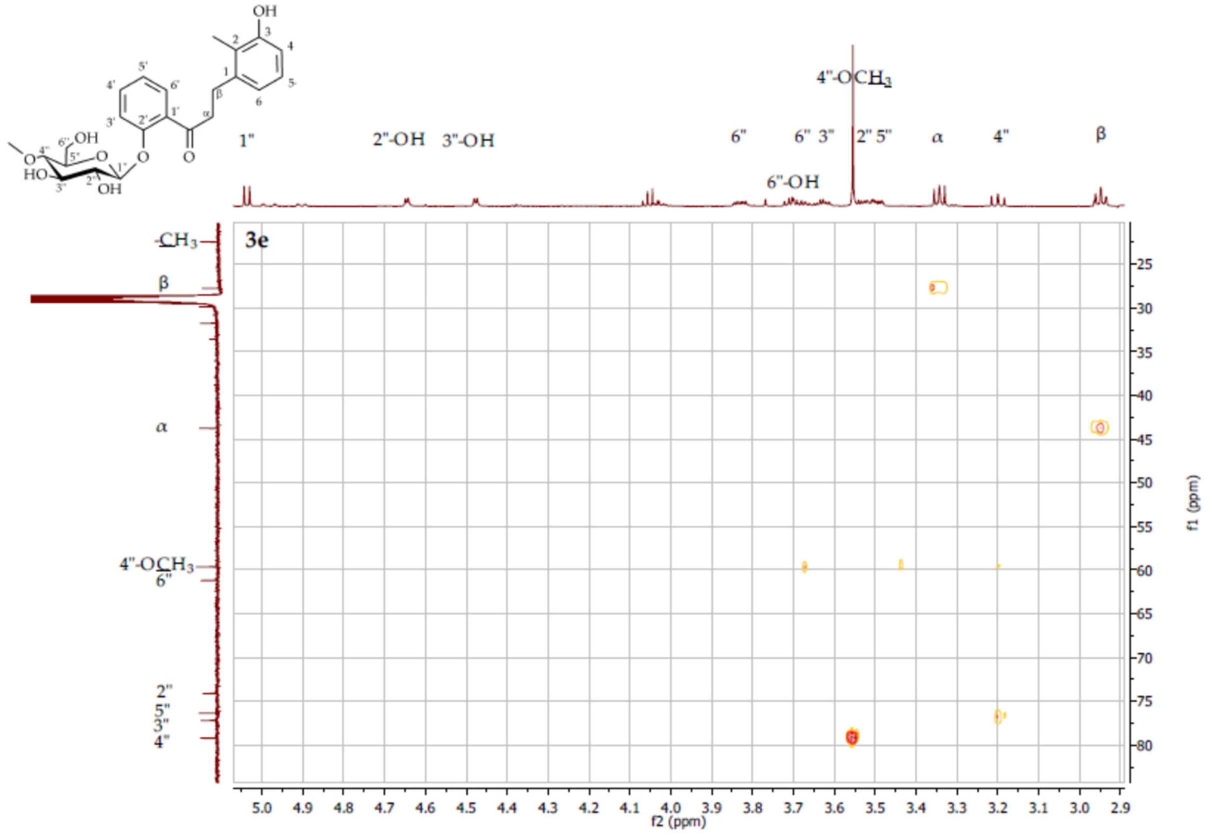

Figure S108. HMBC contour map– ^{1}H x ^{13}C expansion of 3-hydroxy-2-methyldihydrochalcone 2′-*O*-*β*-D-(4′′-*O*-methyl)-glucopyranoside (**3e**)

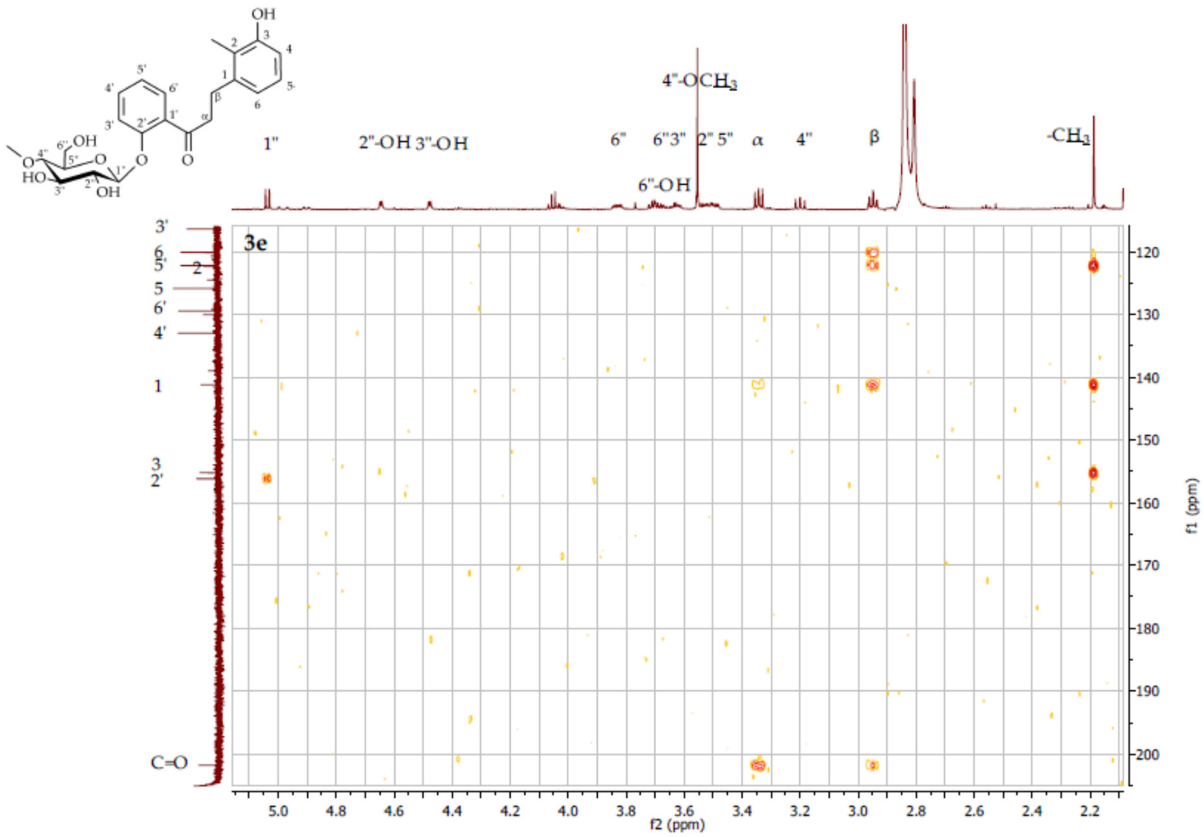

Figure S109. HMBC contour map– $^{1}H \times {}^{13}C$ expansion of 3-hydroxy-2-methyldihydrochalcone 2′-*O*-*β*-D-(4′′-*O*-methyl)-glucopyranoside (**3e**)
